# Haiti has more forest than previously reported: land change 2000–2015

**DOI:** 10.7717/peerj.9919

**Published:** 2020-10-26

**Authors:** Ose Pauleus, T. Mitchell Aide

**Affiliations:** 1 Department of Environmental Sciences, Universidad de Puerto Rico, Recinto de Rio Pidras, San Juan, Puerto Rico, Puerto Rico; 2 Department of Biology, Universidad de Puerto Rico, Recinto de Rio Pidras, San Juan, Puerto Rico, Puerto Rico

**Keywords:** Land use/cover, Forest, Deforestation, Haiti, Landsat imagery, Google Earth Engine

## Abstract

Estimates of forest cover have important political, conservation, and funding implications, but methods vary greatly. Haiti has often been cited as one of the most deforested countries in the world, yet estimates of forest cover range from <1% to 33%. Here, we analyze land change for seven land cover classes (forest, shrub land, agriculture/pasture, plantation, urban/infrastructure, barren land, and water) between 2000 and 2015 using Landsat imagery (30 m resolution) in the Google Earth Engine platform. Forest cover was estimated at 26% in 2000 and 21% in 2015. Although forest cover is declining in Haiti, our quantitative analysis resulted in considerably higher forest cover than what is usually reported by local and international institutions. Our results determined that areas of forest decline were mainly converted to shrubs and mixed agriculture/pasture. An important driver of forest loss and degradation could be the high demand for charcoal, which is the principal source of cooking fuel. Our results differ from other forest cover estimates and forest reports from national and international institutions, most likely due to differences in forest definition, data sources, spatial resolution, and methods. In the case of Haiti, this work demonstrates the need for clear and functional definitions and classification methods to accurately represent land use/cover change. Regardless of how forests are defined, forest cover in Haiti will continue to decline unless corrective actions are taken to protect remaining forest patches. This can serve as a warning of the destructive land use patterns and can help us target efforts for better planning, management, and conservation.

## Introduction

Land change and climate change are the two most important factors affecting the global environment. One of the main impacts of land change is converting forested areas into other land use types, which in turn, affects biodiversity, alters ecosystem services, and has important effects on human well-being (Foley et al., 2005). Satellite imagery has made it possible to monitor land change around the world. Rather than depending on government reports, satellite imagery provides an unbiased data source for estimating land change. Although interpretation of satellite imagery has its challenges, thousands of studies have used Landsat images for local studies. Others have used satellite images to estimate deforestation and forest degradation in the tropics (Souza, 2006; Huang et al., 2008; Cohen, Yang & Kennedy, 2010; Aide et al., 2013; Álvarez-Berríos et al., 2013) to establish long-term patterns of land change and to develop models to predict future land use change using multidecadal time series (Walsh et al., 2001; Carreiras et al., 2017). Recently, Landsat images (30 m resolution) have even been used to map forest cover at the global scale (Hansen et al., 2013; Song et al., 2018).

Despite significant progress in analyzing remotely-sensed data, differences in methods of data collection, data sources, and methods pose issues for land change assessment and forest estimates (Bewket & Abebe, 2013). As such, national and international agencies, government, non-government organizations, and scientists involved in land change assessment and forest monitoring have reported different results due to the use of diverse satellite data sources, various classification methods, and different definitions of land use/cover types (Rindfuss et al., 2004; Bewket & Abebe, 2013). For example, there are significant differences between the Global Forest Watch’s (GFW) and the FAO’s Global Forest Resources Assessment (FRA) results of global forest cover loss. FRA reported a global forest cover loss of −3.3 Mha/yr during 2015 while GFW reported a loss of −19.8 Mha/yr for the same period (Abdollahnejad, Panagiotidis & Surový, 2017). Another important point to consider is whether we are assessing well-preserved forest (very low human intervention) with an interest in biodiversity conservation or forest cover in general with a broader interest that includes biological diversity, ecosystem services, and socio-ecological systems (Barlow et al., 2007; Van Breugel et al., 2013; Lennox et al., 2018; Hedges et al., 2018).

Haiti is a good example of how forest cover estimates can vary greatly. Recently, Hedges et al. (2018) estimated well-preserved forest cover to be only 0.32%, but they did not consider secondary forests which represent most forest patches in the country. The 2010 FRA revised report published by the Food and Agriculture Organization of the United Nations estimated forest cover for Haiti at less than 4% in 2010 (Food & Agriculture Organization, 2010). Although the report has been criticized for using different vegetation classification methods, and incompatible land use classes for the Caribbean with different forest definitions (Evelyn & Camirand, 2003; Niknejad, Zadeh & Heydari, 2014; Keenan et al., 2015), it has been cited widely and used by many institutions for political, economic, and environmental causes (Evelyn & Camirand, 2003). Based on these and other publications and reports, Haiti is often referred to as one of the world’s most deforested and environmentally degraded countries (Posner, Michel & Toussaint, 2010; Maertens & Stork, 2017). For instance, the US National Geographic satellite image (Landsat 5) of 2001 highlighting the stark contrast in forest cover at the Haitian/Dominican Republic border has been used widely to exemplify the status of forests in Haiti (Michel, 2001; Posner, Michel & Toussaint, 2010; Maertens & Stork, 2017). In contrast, other studies have estimated forest cover to be greater than 30% (Churches et al., 2014). These estimates include both well-preserved forest remnants and secondary forests because the latter have been found to be a valuable habitat for a wide array of species. Unfortunately, most studies in Haiti have focused on a forest/no forest dichotomy, while few studies have mapped how land use/cover have changed over the last few decades. This is important because it will help formulate effective policies for better land-use management. Furthermore, it is essential to know the drivers of forest cover loss. Deforestation, for example, has been attributed mostly to charcoal production, because forested lands are under local and distant pressures due to fast growing population that competes for food, firewood, and raw materials (Versluis & Rogan, 2009; Posner, Michel & Toussaint, 2010; White et al., 2013).

In this work, we use Landsat imagery in the Google Earth Engine (GEE) and high-resolution imagery in Google Earth to create a high-precision classifier to produce accurate land use maps of Haiti. Specifically, we produced detailed maps with seven land cover classes for 2000 and 2015. With these data, we address the following questions: (1) How has forest cover changed from 2000 to 2015? (2) What are the important transitions among the different land use types? (3) Where are important changes happening? (4) How do our results compare with forest estimates from previous studies? (5) What can be considered as forest in Haiti? and (6) What are the implications of our results for conservation, management, and development for Haiti? The answers to these questions will serve as a basis for understanding land change dynamics in Haiti and provide information for future natural resources management.

## Materials and Methods

### Study area

**Figure 1:**
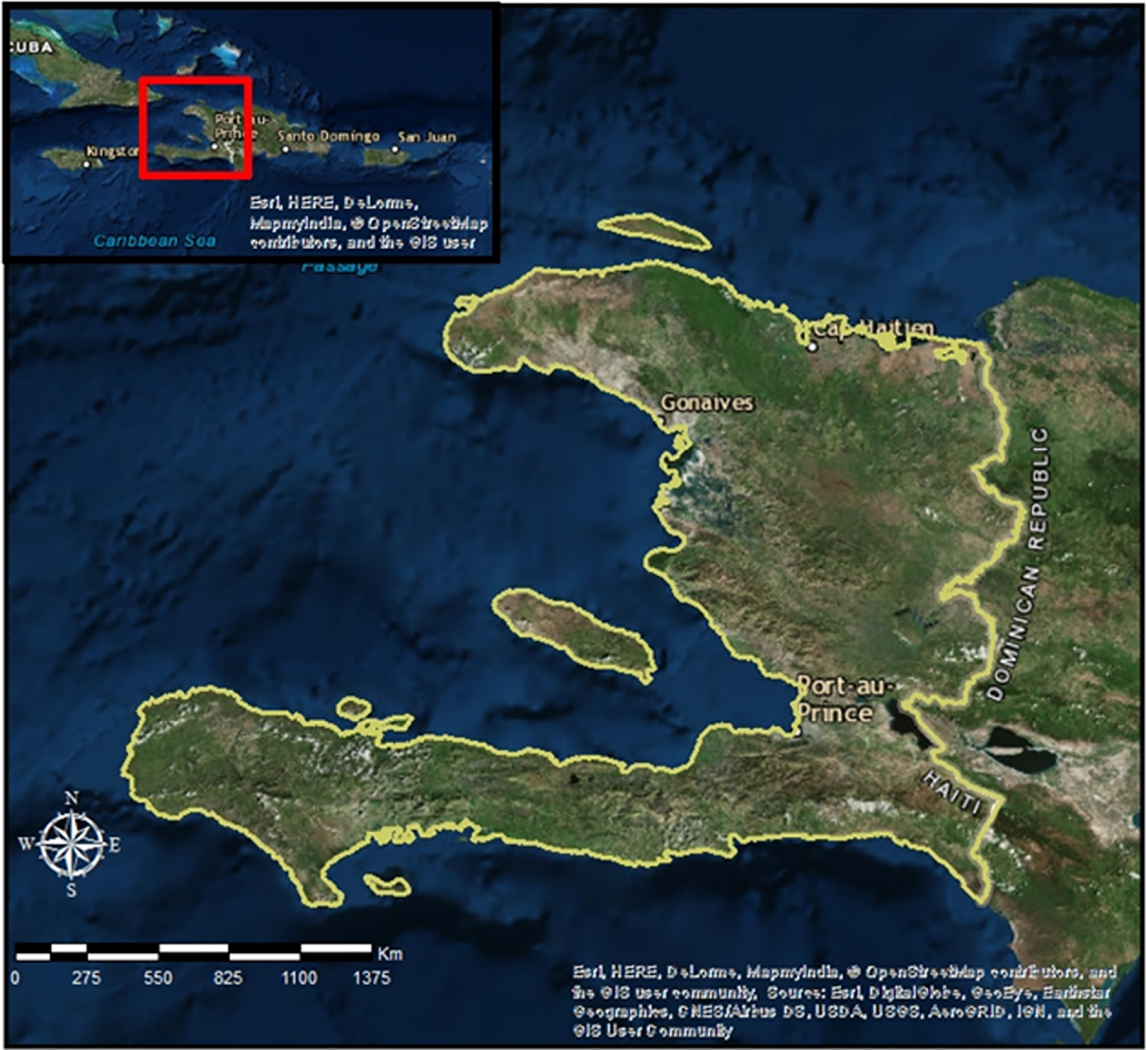
The location of the study: Haiti in the Greater Antilles and the western side of Hispaniola delimited in light green Haiti in the Greater Antilles and the western side of Hispaniola delimited in light green, and the insertion of part of the Caribbean isla. Source: Landsat 8 OLI images. USGS/NSAS Lansdat (https://landsat.usgs.gov/sites/default/files/documents/Landsat_Data_Policy.pdf).

The Republic of Haiti (27,750 km^2^) occupies the western side of Hispaniola, which is the second largest island of the Greater Antilles (Fig. 1). The country is politically divided into 10 departments (provinces), 42 “arrondissments” (districts), 145 “communes” (municipalities; Table S4; Fig. S1), and 571 communal sections (Central Intelligence Agency, 2017), and the capital is Port-au-Prince. Temperatures range from 25 °C to 35 °C between June and September and from 15 °C to 25 °C between late December and early March. November through January are considered the driest months. The rainy season extends from February to May, and the hurricane season occurs between June and November. The average annual rainfall is 1,397 to 2,006 mm, with more rainfall in the southern peninsula and in the northern plains (Huntington et al., 2017). The country’s topography is reflected in its name: “Ayti” derived from the indigenous Arawak that means “mountainous land” and altitude ranges from sea level to Massif de la Selle in the South at 2,680 m (Central Intelligence Agency, 2017). About two-thirds of the country is covered in mountains composed mainly of limestone. Volcanic soils occur in the Massif du Nord. Karstic features, such as limestone caves, grottoes, and subterranean rivers can also be found in many parts of the country. The main forest types in Haiti include tropical and subtropical moist forests, tropical montane cloud forests, dry limestone forest, tropical and subtropical coniferous forests and mangroves. Despite extensive environmental degradation, Haiti has some of the highest levels of biodiversity in the Caribbean because of diverse microclimates created by the mountainous landscape. Much of the biodiversity is concentrated in key biodiversity areas (mostly protected zones; Fig. S1). In addition, more than 75% of the Haitian fauna is endemic (Central Intelligence Agency, 2017).

Despite being the first black republic in the world, Haiti has high levels of political violence, government instability, and environmental catastrophes, and is considered to be the poorest country economically (gross domestic product (GDP), purchasing-power-parity (PPP) per capita) in the western hemisphere. In 2017, the population was estimated to be ~11,085,919 with a yearly growth rate of 1.71% and unemployment estimated at 13.2% (Central Intelligence Agency, 2017). Though vulnerable, agriculture plays a major role in the Haitian economy by employing more than half of the total workers. Charcoal production also plays an important economic role in rural areas. Charcoal and firewood are considered the primary sources of fuel for more than 80% of the people living in cities. The economic and political instability of the country, along with high population growth, high unemployment rate, high firewood/charcoal consumption, and lack of effective environmental regulations make the country vulnerable to economic and environmental disturbances.

### Satellite imagery

Google Earth Engine provides pre-processed Landsat image collections through its platform for fast data analysis. The pre-processing methods include the selection of pre-orthorectified, radiographically, atmospherically corrected images, cloud removal, and missing value filling (Gorelick et al., 2017). The satellite data includes multi-spectral image collections with cloud mask confidence between 0 (none) and 1 (<12%) with 30 m spectral resolution from Landsat 7 ETM+ and Landsat 8 OLI/TIR for 2000 and 2015, respectively. To produce the composites, median values were calculated for all cloud-free pixel from images for each year (Huang et al., 2010). The data used for this study were collections of multi-temporal Landsat satellite images for a 12-month time period (from the end of December of 1 year to the beginning of January of the next year) at a 30 m spatial resolution. For this study, we used data from 2000 and 2015.

### Training data

Training data were created from freely available high-spatial-resolution imagery using GEE platform (Gorelick et al., 2017; Gómez-Chova et al., 2017). We inspected high-spatial-resolution satellite images from 2000 and 2015 to identify pixels of seven landcover classes (i.e., forest, shrub land, agriculture/pasture, plantation, urban/infrastructure, barren land, and water; Table 1). Training samples were obtained by drawing a polygon around approximately homogenized area of a single landcover class. Pixels within the polygon were selected randomly for the training data. The polygons were chosen using a stratified approach in which we selected polygons for each cover class in the four major regions of the country (north, south, central, and west) including a few of the larger islands of Haiti. Approximately 355 training points (i.e., pixels) were used for each of the seven land use/cover types (Table 1).

**Table 1:** Criteria of land use/cover types used in selecting the training data for creating the classification models. All training data were collected using high resolution imagery in Google Earth.

Land use/cover types	Criteria
Forest	Areas of natural or semi natural tall woody vegetation cover (≥5 m) generally with more moderate canopy (≥80% or more cells in the training polygons are trees that means ≥80% forest canopy in a pixel).
Shrub land	Natural or semi natural shrub vegetation generally with a more opened canopy and short woody vegetation (<5 m), where 80% or more cells in the training polygons were shrubs.
Agriculture/Pasture	Areas cover with annual or biannual crops and/or fallow lands mixed with grasslands Often with clear signs of cultivation (i.e., presence of linear till rows).
Plantation	Stand of trees or shrubs cultivated in rows for commercial purposes. This class includes eucalyptus, coffee, fruit trees palm and coconut trees.
Urban/infrastructure	Area with human-made structures including Residential, commercial, industrial, transportation, roads, and mixed urban where more than 95% of cells in training polygons were infrastructure.
Barren land	Areas of exposed soil and barren area with less than 20% vegetation cover
Water	River, open water, lakes, ponds, reservoirs.

To avoid biased evaluation in the accuracy results, the sampling strategy was designed to avoid overlap or partial overlap between the training and testing data. We separated Regions of Interests (ROIs) into training and testing polygons by using the randomColumn and sampleRegions functions in GEE. Through the randomColumn function, a random number between 0 and 1 was assigned to each polygon. Values less than ≤0.70 were designated to training data while values greater than 0.70 were assigned to testing data (Liang et al., 2017). We undertook the same process at the pixel level, where pixels for training data were assigned a value less than ≤0.70. In addition, the polygons for the training and testing data for different classes were separated by at least one km to avoid spatial autocorrelation (Zhen et al., 2013).

### Image classification in GEE

For image classification, we used the cloud-computing technology in GEE platform (https://earthengine.google.org/). We used GEE’s image collections which covered Haiti for 2000 and 2015. To classify the images, we created a random forest decision tree classification algorithm in GEE based on Landsat spectral bands (B0 to B8). The classification of each pixel was based on a majority vote of 30 trees. Random forest has been widely used to map land use/cover classification (Liu et al., 2018; Mandianpari et al., 2017; Tian et al., 2016; Rodriguez-Galiano et al., 2012; Guo et al., 2011) and land cover dynamics (Johansen, Phinn & Taylor, 2015; Huang et al., 2017).

### Estimate classification error with independent validation data

To validate the random forest classification model, 70% of pixels from each class were randomly selected to create the model, and 30% of the pixels served as a validation dataset. We estimated the overall, producer, consumer, kappa accuracy scores, and we created a confusion matrix (Story & Congalton, 1986; Fielding & Bell, 1997). Two land use types, agriculture and pasture, were united to reduce errors. Farmers in Haiti usually use a mixed-farming system where crops are grown and harvested, and residues serve as pasture for grazing. Sometimes farmers leave their lands in fallow (i.e., pasture), which can create confusion because these areas are clearly part of an agricultural landscape (Laganière, Angers & Paré, 2010).

To further verify classification error, we conducted a post-classification validation of forest and non-forest areas using high-resolution images in Google Earth (GE; Table S2). The post classification evaluation was based on visually classifying 50 randomly selected polygons of forest or non-forest for each year, and the results were compared with the 2000 and 2015 maps. In addition, we also extracted statistics data (percent tree cover) from Hansen et al. (2013) to compare with our data.

### Post-classification

To refine the class assignment of a pixel after its initial classification using random forest, we used a post-classification smoothing function in the spatial analysis component of ArcGIS (Tilton, Swain & Vardeman, 1981; Campbell & Wynne, 1987). This step is important because it allows us to reduce noise due to spectral similarity among different classes in our land use/cover maps. We divided the post-classification correction technique into several steps. First, the majority filter tool removed isolated and/or small groups of pixels for each class from the image. Then the boundary clean tool smoothed out rough edges and clustered the classes. Lastly, the region group, set null, and nibble tools from ArcGIS spatial analysis tools were used to reclassify the isolated and small groups of pixels to the nearest associated classes to form maps.

### Change detection

To determine changes in land use/cover between 2000 and 2015, both at the national and municipality level, a post-classification comparison method was applied using the overlay procedure in ArcGIS (Tilton, Swain & Vardeman, 1981; Campbell & Wynne, 1987). Specifically, the Global Administrative Areas (GADM) shape file (municipality level) was superimposed on the post-classified corrected 2000 and 2015 land use/cover maps. The spatial analyst tool (raster calculator) in ArcGIS was used to quantify the land cover transitions between 2000 and 2015. The methods provided “from-to” change information that can be mapped and calculated.

## Results

### Classification accuracy assessment

The classification models from GEE had overall accuracies of 94% (Kappa = 0.92) and 92% (Kappa = 0.93), for 2000 and 2015 respectively (Table S1). Similarly, our manual post-classification assessment of forest and non-forest areas showed accuracies of 91% and 93% for 2000 and 2015, respectively (Table S2). Although the accuracies of the agriculture/pasture class in the classification model were high (2000: 90%, 2015: 87%), this class was occasionally confused with the shrub land and plantation classes (Table S1), reflecting the complex dynamics of the agriculture/pasture system in Haiti. Agricultural lands are frequently surrounded by or mixed with trees, shrubs, fruit trees, and pasture. They can also be left in fallow, and this often leads to colonization of shrubs, which can cause confusion among the different land use classes.

### Land use/cover areas in Haiti between 2000 and 2015

**Figure 2:**
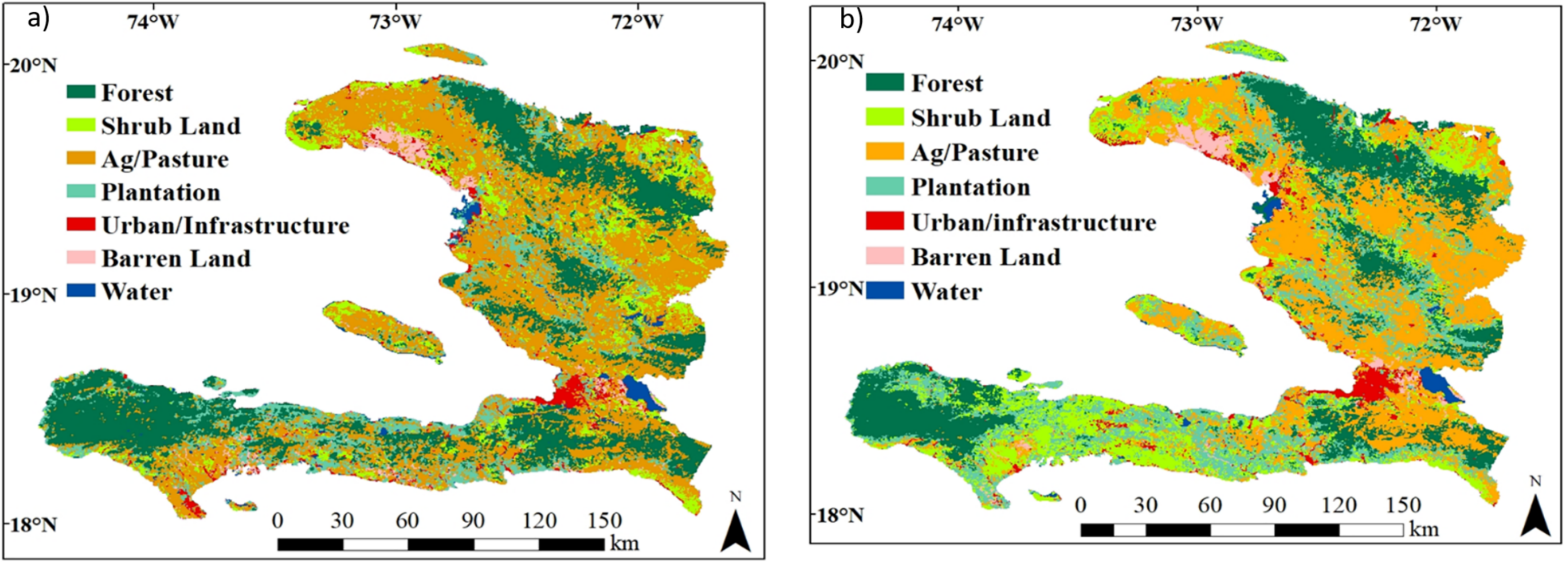
Maps displaying the land use/cover classification across Haiti for (A) 2000 and (B) 2015.

**Figure 3:**
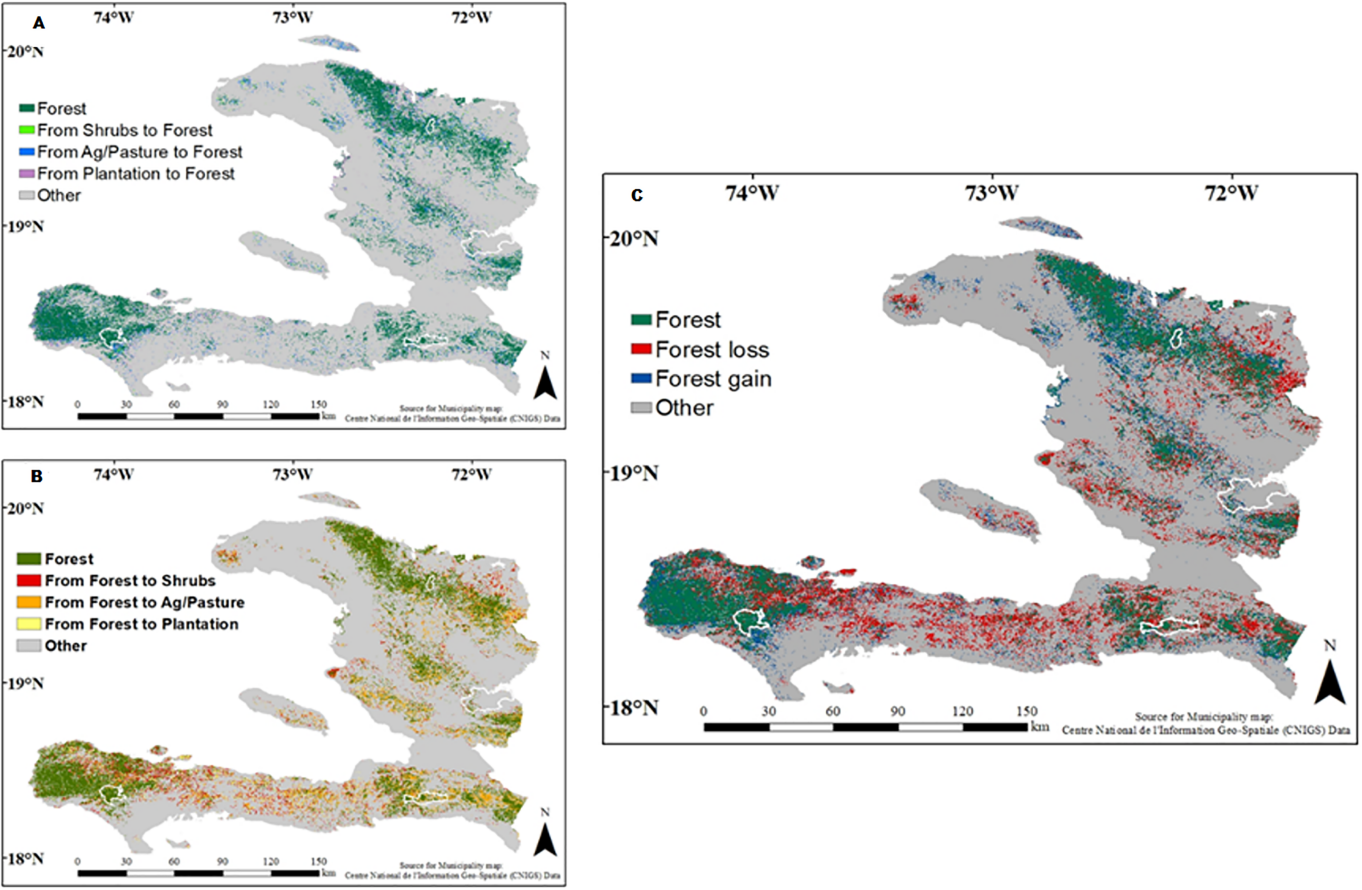
Maps displaying the land use conversion across Haiti for the period of 2000 to 2015. (A) Forest cover gain from shrubs, mixed agriculture pasture and plantation; (B) forest loss from conversion to shrubs, agriculture/pasture and plantation; (C) a combination of (A) and (B). (Other) includes the other land use classes’ conversion. The areas of four protected areas are outline in white (UNEP-WCMC, 2018; https://www.unep-wcmc.org/policies/data-policy#data_policy).

The distribution of land use/cover classes varied between 2000 and 2015 (Table 2 and Fig. 2). The agriculture/pasture class covers the greatest area in Haiti, and it increased from 39.7% in 2000 to 47.9% in 2015. The forest class was the second most important, but this class declined from 26.5% in 2000 to 21.3% in 2015. Forest loss mainly occurred in the south near the protected areas of Pic Macaya National Park of the Massif de la Hotte and La Visite National Park of the Massif de la Selle and in the northeast near the Three Bays National Park (Fig. 3C; Table S3). The next two most important classes were plantations and shrublands, which together covered approximately 25% of Haiti. The plantation class declined from 16.29% to 9.79% between 2000 and 2015, while the shrubland class increased from 11.38% in 2000 to 15.09% in 2015. The urban/infrastructure, water, and barren land classes accounted for 5–7% of the total area (Table 2).

**Table 2:** Forest cover for the years 2000 and 2015 in Haiti. Areas are provided in square kilometers (km^2^) and percentage (%).

Land cover type	2000		2015	
	Area (km^2^)	(%)	Area (km^2^)	(%)
Forest	7,625.07	26.46	6,149.69	21.34
Shrub land	3,279.31	11.38	4,350.12	15.09
Ag/Pasture	11,444.14	39.72	13,816.27	47.95
Plantation	4,695.20	16.29	2,820.36	9.79
Urban/infrastructure	703.06	2.44	661.65	2.29
Barren land	540.55	1.87	713.33	2.47
Water	522.43	1.81	300.00	1.04
Total	28,811.76	100.00	28,811.76	100.00

#### Land use/cover conversion

The cross-tabulation matrix (Table 3) was used to detect the nature of the change between the different land use/cover classes. The loss of forest cover between 2000 and 2015 was mainly due to its transformation into mixed agriculture/pasture (1,555 km^2^), shrublands (1,045 km^2^), and plantations (501 km^2^). The area of plantations also strongly declined, and these areas were mainly transformed into mixed agriculture/pasture (1,984 km^2^) and shrublands (1,147 km^2^). Between 2000 and 2015, mixed agriculture/pastureland was the class with the largest increase in area and this was mainly due to replacing plantations (1,677 km^2^), forest (1,555 km^2^) and shrub lands (1,315 km^2^; Fig. 3). Most of the changes in the other land classes had a much smaller impact (Table 3).

**Table 3:** Transition matrix of land use/cover classes in Haiti in 2000 and 2015 (km^2^). Diagonal values in bold represent the percentage area that was not converted from one land use/cover type to another during the study period.

2015 (km^2^)							
2000	Trees	Shrub	Ag/Pasture	Plantation	Urban/infrast	Barren	Water
Trees	**4,503.50**	1,045.55	1,555.01	501.24	6.08	5.47	8.22
Shrub land	175.32	**715.45**	2,024.86	213.87	117.99	27.44	4.38
Ag/Pasture	637.23	1,226.24	**7,792.27**	1,338.45	143.53	299.92	8.50
Plantation	759.27	1,147.21	1,984.44	**689.61**	72.43	32.38	9.92
Urban/infrast	4.93	89.64	165.14	31.30	**283.97**	117.68	10.40
Barren land	17.64	66.53	189.42	32.87	24.65	**205.58**	3.86
Water	51.80	59.50	105.13	13.02	13.00	24.86	**255.12**

## Discussion

Our analyses of land use in Haiti estimated that forest cover declined from 26% in 2000 to 21% in 2015, which has had negative consequences for biodiversity and other ecosystems services (Mladenoff et al., 1993) These results are well above the 0.32% of well-preserved forest cover reported by Hedges et al. (2018), the 16.17% tree cover for 2000 by Hansen et al. (2013), and the 4% forest cover estimated by the Forest Resources Assessment (FRA) of Food and Agriculture Organization (FAO) and several other forest estimates and agency reports on forest cover in Haiti (Table 4; Michel, 2001; Food & Agriculture Organization, 2010). Our results are comparable with the 24% forest cover reported by the GFW (Global Forest Watch, 2018) for 2010, but they are lower than the 29–32% forest cover in 2010 reported by Churches et al. (2014). An important difference between our study and Churches et al. (2014) is that they include mangrove trees in their tree class, while we included mangroves in the shrub class (Table 4; Churches et al., 2014). If we combine the areas of our tree and plantation classes, we will have a “forest” cover area of 31% within the range reported by Churches et al. (2014) for 2010. The main cause of forest loss during this period has been attributed to forest clearing for agricultural expansion, which might also include the illegal logging for charcoal production fueled by population growth (Stevenson, 1989; Cho, 2011; Ferguson et al., 2018). As the Haitian population grows, the demand for natural resources (e.g., food, wood for cooking) increases proportionally (Cho, 2011; Pomeroy & Ragone, 2015). In fact, Haiti is one of the most densely populated countries in the Caribbean, with an estimated population of more than 11 million, a population growth rate of 1.71%, and an estimated projection of 14.8 million people for 2050. After the 2010 earthquake, which displaced millions of people from the capital to rural areas, both illegal logging and forest clearing for agriculture would have most certainly increased. For example, the population of the island of La Gonave increased by 20%, significantly increasing the demand for food and shelter (Kushner, 2014). Furthermore, the absence of a national forest protection program and weak law enforcement throughout the country allows for easy access to forest areas; it is not surprising that illegal logging for charcoal production is a common driver of forest loss in Haiti (Cho, 2011). For instance, 1.3 million tons of wood are harvested each year for charcoal production (Pomeroy & Ragone, 2015).

**Table 4:** Comparing Haiti forest cover estimates based on satellite data with previous studies on the period 1972–2015. We compared our estimates of forest cover with the estimates from seven previous studies. Forest cover areas are provided in percentage.

Sources	Location	Forest cover (%)	Methods	Agency	Date	Forest definition
Lanly (1982)	Tropics (include Haiti)	1.73	Landsat 1 & 2	FAO	1972 & 1978	Broadleaved woody vegetation with more than 10% canopy cover.
Ortiz-Chour, Davis & Pugliese (2000)	Tropics (include Haiti)	3.2	Compilation of National Statistics.	FAO	2000	Woody vegetation with >10% crown cover including plantation; excludes trees for agricultural production.
Food and Agriculture Organization of the United Nations (2010)	Global Forest	4	National forest inventories compiled by the FAO	FAO	2010	Woody vegetation with >10% of tree crown cover >5-m tall. Closed forests with tree crown coverage >60%.
Álvarez-Berríos et al. (2013)	Great Antilles	8	MODIS Vegetation Index image; random forest classifier	NA	2000 & 2010	Trees and shrubs with >80% cover.
Hansen et al. (2013)	Global Forest	16.17	Wall to wall 30 m resolution Landsat images.	University of Maryland	2013	>25% canopy (2013), >5 m height, tree cover.
Churches et al. (2014)	Haiti	29.4 & 32.4	Modified normalization method using pseudo-invariant polygons, Landsat 5 image classification.	NA	2010	Vegetation greater than 5 m in height with a canopy cover of ≥10%. Includes mangroves. Exclude fruit plantations.
Hedges et al. (2018)	Haiti	0.32	Landsat 5, 7 & 8	N /A	1984–2016	Old growth with closed forest cover canopy (≥70% tree cover) with little to no human intervention.
This study	Haiti	26.4 & 21.3	Wall to wall 30 m resolution Landsat images using GEE	NA	2000 & 2015	Dominant tree cover class with moderate canopy (≥10%; 80% or more cells in the training polygons are trees).

In addition to forest loss, we also observed a decline in the plantation class (Fig. 3). Several factors may have contributed to this decline. First, the capital, Port-au-Prince, was in reconstruction after the devastation caused by the 2010 earthquake. This could have increased the demand for wood, which was likely sourced from areas that we classified as plantations. Second, following the 2010 earthquake, international donors increased funding for crop production, and this may have incentivized farmers to convert plantations into agricultural lands (Versluis & Rogan, 2009; White et al., 2013).

Forest and plantation were mainly replaced by the agriculture/pasture class, which expanded from 39.7% in 2000 to 47.9% in 2015; and the shrub class, which expanded from 11.38% in 2000 to 15.09% in 2015 (Table 2). These findings are consistent with USAID and other international agencies’ reports on agriculture (World Bank, 2013; Inter-American Development Bank) in Haiti. Following the 2010 earthquake, international agencies (e.g., USAID, World Bank, 2013) increased funding to the agricultural sector, and this resulted in an increase in agricultural production (Fuller-Wimbush & Fils-Aimé, 2014; United States Agency for International Development, 2017). For example, the Inter-American Development Bank (IDB) approved a $15 million grant to reform agriculture in Haiti (United States Agency for International Development, 2017) and USAID spent $126 million on a 5-year project to help rebuild Haitian agricultural infrastructure and production.

Along with the increase in the agricultural/pasture class, we also observed an increase in the shrubland class. Other studies have also reported increase in shrubland in the island of La Gonave and Pic Macaya National Park in the southwest region of Haiti’s mainland (Vital, 2008; White et al., 2013). The increase in shrubland in our study may also be related to the availability of funds from international donors. Reforestation projects to counter the deforestation in Haiti are popular projects for international funding. Consequently, many NGOs have established reforestation programs and have transplanted many seedlings/saplings across Haiti, especially near areas of degraded forest (Lall, 2013; Hincha-Ownby, 2013). Since the 2010 earthquake that killed more than 300,000 people and displaced more than a million people (DesRoches et al., 2011), more than 5 million seedlings/saplings have been planted (Lall, 2013; Hincha-Ownby, 2013). A severe drought in 2013 (Food & Agriculture Organization, 2016) might have slowed growth of these plants and might be why we classified more areas as shrublands.

Given the international interest in the Haitian environment (i.e., forest cover) why do forest estimates vary so widely? Forests are defined, assessed, valued, and managed through different lenses depending on the interests of each study or actor. Forests may be valued for their biomass, biodiversity, ecosystem services, or all of these aspects (Lennox et al., 2018). For example, Hedges et al. (2018) in their forest assessment from 1988 to 2016 valued forests mostly for biodiversity. The present study specifically included secondary and degraded forest to capture all potential value (e.g., biomass, biodiversity, ecosystem services). Most of Haiti’s forests were cleared during the period of Spanish, French, and American colonization (Tarter, Freeman & Sander, 2016), and they have continued to decline following Haiti’s independence (Cho, 2011). Given this long history of land use and frequent hurricanes, it is not surprising that most forest cover in Haiti consists of secondary forest. Consequently, any forest management or forest cover estimate in Haiti should consider both remnant, well-preserved forest along with secondary forests. Both are crucial not only to biodiversity, but also because they comprise more land area than well-preserved forests and provide several ecosystem services. Although secondary forests usually have lower species richness in comparison with well-preserved forests, they can provide important habitat for various species, especially in highly degraded and fragmented landscapes (Barlow et al., 2007; Aerts & Honnay, 2011; Edwards et al., 2011; Van Breugel et al., 2013; Lennox et al., 2018). For example, in the year 1940, Puerto Rico had less than 10% forest cover (Lugo, 2004), but following extensive socio-economic changes, forest cover has increased continuously and was estimated to be ~55% in 2014 (Marcano-Vega, 2017), providing habitat for diverse native, naturalized, and introduced species.

Consequently, our forest cover estimates included both well-preserved and secondary forests. We consider our forest cover estimates of 26% in 2000 and 21% in 2015 to be conservative, because we classified shrubs and plantation as separate classes, and we used a medium canopy cover threshold. Even with our conservative approach, we estimated higher forest cover than Hansen et al. (2013) for 2000 and approximately 10 to 20 times more forest than recently published forest cover estimates for Haiti (Hedges et al., 2018) and FAO, respectively (Food & Agriculture Organization, 2010). Our forest cover estimates differ greatly with the results of Hedges et al. (2018) because they restricted their assessment to well-preserved forests with high biodiversity value, and they defined forest as well-preserved (old growth) with closed forest cover canopy (≥70% tree cover) that has not been deforested or degraded (Table 4). FAO defines forest cover as natural forests and forest plantations with an extent more than 0.5 ha and more than 10% tree canopy cover (Table 4; Food & Agriculture Organization, 2010), but they depend on national forest estimates and countries often using different spatiotemporal scales (Evelyn & Camirand, 2003; Food & Agriculture Organization, 2010, 2016; O’Connor, 2016). For Hansen et al. (2013), their definition of forest includes “all vegetation taller than 5 m in height with >25% canopy” that includes all plantation (trees, palm, and coconut tree), while Churches et al. (2014) define forest as vegetation greater than 5 m in height with a canopy of ≥10% (Table 4). In contrast, we defined forest as areas with 80% or more of natural or semi−natural tall woody vegetation (≥5 m) with closed canopy, that is, 80% of the pixels are composed of woody vegetation (Table 1). Possibly the most significant difference between our study and the other studies is that we included other land use classes, which helps to provide more details on land use dynamics in general.

The land change trends that we observed were characterized by the loss of forests and plantations and a gain in agricultural/pasture and shrubs lands (Figs. 3A–3C ). These trends have significant implications for conservation, management, and development for the country. In Haiti, conservation efforts have focused on deforestation caused by charcoal production, while in light of this study and others, the main cause of deforestation is still closely related with agricultural expansion (Chidumayo, 1993; Chidumayo & Gumbo, 2013; Mwampamba et al., 2015; Masoero, 2016). Targeting a misconstrued proximate driver of deforestation can have important implications for forest management planning. First, as Haiti is a mountainous country, the loss of forest cover and the rise in agriculture increase run-off greatly, cause the loss of the topsoil through erosion and increase the probability of deadly floods. For instance, the country had six major floods in 2 months (April and May) in 2016, causing severe economic and environmental damages (Curtis & Hodell, 1993). The increase in flood activities further increases loss of topsoil and subsequent loss of flora and fauna (Tarter, Freeman & Sander, 2016). This phenomenon has tremendous implications for conservation, as the loss of the topsoil associated with prolong drought can promote the establishment of new plant communities (often invasive species) and reduced biodiversity and ecosystem services (Curtis & Hodell, 1993; Tarter, Freeman & Sander, 2016). As stated before, Haiti is one of the most biodiverse countries in the Caribbean (Churches et al., 2014). and if no immediate action is taken to protect this biodiversity, the nation will continue to lose countless rare and endemic species. In addition, the continuous degradation will affect development and the well-being of its people by making the country more vulnerable to environmental hazards and natural disasters (Tarter, Freeman & Sander, 2016).

Haiti needs improved land management and environmental policies at the national level, and local-based and appropriate conservation actions and land use policies and enforcement that involve the local community to insure the protection of remaining natural resources and land preservation. There is also a need for well-planned reforestation projects, environmental education, and research to better manage the rate, extent, and intensity of human-caused deforestation to mitigate forest loss and decrease pressure on biodiversity. Both national and international institutions, government, and NGOs should promote the use of real-time high-spatial-resolution imaging, Rapideye and Sentinel data (1 & 2), and reliable data for more accurate results. For this, it is essential to have a clear local definition of forest cover with a defined national vegetation classification, and a well-organized system to collect, analyze, and distribute vegetation data. Moreover, it is vital to establish long-term land cover change monitoring programs at the national level through the formation of a functional national land use institute. Hence, we can better understand the dynamics of Haiti’s landscape to identify the main driving forces of land change.

One potential limitation of this study could be a slight overestimation of the GEE accuracy assessment due to potential autocorrelation created by misclassified pixels between the training and testing data. This occurs when the independent assumptions between training and testing samples are lost and pixel overlapping or partial overlapping occur (Friedl et al., 2000; Millard & Richardson, 2015). This can improve classification accuracy (Zhen et al., 2013). Strategies to reduce the autocorrelation were applied as described in the methods. As mentioned in the results, we observed misclassified pixels between agriculture and pasture that might be related to Haitian farmers’ systems. This error might also be related to autocorrelation. These types of errors integrated in testing data impact area estimations and amplify the tendency for overestimation of the class (Foody, 2013). Even with slightly possible error, the overestimation of the accuracy assessment is likely to be small. According to Zhu et al. (2000), stratified sampling tends to improve precision and reduce bias, and this usually results in a low percentage of overestimation of the accuracy assessment (Millard & Richardson, 2015). Most importantly, the independent post-classification evaluation of forest/no forest cover provides strong support for the overall classification and conclusion of this study.

## Conclusions

This current study assessed land use/cover change and change detection in Haiti for the years 2000 and 2015 using GEE, a powerful tool for remote sensing data analysis and classification. We confirmed that Haiti is losing forest cover resulting from agriculture and shrubs expansion. These changes are happening mainly in the southern area near protected areas with key biodiversity conservation potential, such as Pic Macaya National Park of the Massif de la Hotte and La Visite National Park of the Massif de la Selle, which include remnants of well-preserved forests. However, we also showed that forest cover is about 10 to 20 times more than previously cited, mainly due to differences in classification methods, definition of forest, and politics. These land change patterns have important implications for soil erosion, biodiversity loss, and ecosystem services, which will increase the vulnerability of the country to hazards and natural events such as hurricanes and floods. Current and future efforts should focus on land management, and environmental policy actions and enforcement to reduce the rate, extent, and intensity of deforestation of both well-preserved and secondary forests. Funding should be allocated to investigate the main drivers of land use change in Haiti, study the structure and composition of the remaining forest patches, and increase environmental education programs. In addition, national and international institutions should develop a functional land use institute and establish a long-term land cover change monitoring program at the national level to better understand the dynamics of Haiti’s landscape.

## Supplemental Information

10.7717/peerj.9919/supp-1 Supplemental Information 1 Haiti Municipalities. The numbers represent the names of the municipalities found in Table S4. The polygon shapefile is from CNIGS, 2018. Open source Haitian Centre for Geospatial Information. http://haitidata.org/people/profile/cnigs/?limit=20&offset=40

10.7717/peerj.9919/supp-2 Supplemental Information 2 Accuracy assessment achieved by GEE classifier for 2000 and 2015 image classifications. An average of 156 points per class was distributed randomly according to the validated and classified training point’s information by GEE to generate the error matrix with producer’s and user’s accuracy. (A) is for 2000 and (B) for 2015.

10.7717/peerj.9919/supp-3 Supplemental Information 3 Classification validation of forest and non-forest areas. The accuracy of the classified 2000 and 2015 classified images were evaluated by exporting them into google earth and by choosing 50 polygons for forest and non-forest areas randomly. Error Matrix was calculated according to [38-39] in the main reference of the article. (A) is for 2000 and (B) for 2015.

10.7717/peerj.9919/supp-4 Supplemental Information 4 Forest Cover Areas in km^2^ by municipalities for the years 2000 and 2015. This data was extracted from the land use/cover maps using the municipality layer of Haiti through ArcGIS. Empty box represents no data.

10.7717/peerj.9919/supp-5 Supplemental Information 5 Haiti departments and municipalities.

10.7717/peerj.9919/supp-6 Supplemental Information 6 Training point for land use/cover random forest models.

10.7717/peerj.9919/supp-7 Supplemental Information 7 Code used in data analysis for land use/cover map 2000. The code used JavaScript programing language within the Google Earth Engine code editing environment. They include scripts for image compositing and for image pre-processing, classification, and accuracy assessment.

10.7717/peerj.9919/supp-8 Supplemental Information 8 Code used in data analysis for land use/cover map 2015. The code used JavaScript programing language within the Google Earth Engine code editing environment. They include scripts for image compositing and for image pre-processing, classification, and accuracy assessment.

10.7717/peerj.9919/supp-9 Supplemental Information 9 Code used to extract Hansen forest cover percentage for the year 2000. The code used JavaScript programing language within the Google Earth Engine code editing environment. They include scripts for threshold to determine forest or no forest, tree cover 2000 distribution, forest gain or loss.

## Additional Information and Declarations

### Data Availability

The following information was supplied regarding data availability: The data and codes/scripts used for the land use analyses are openly accessible in the Google Earth Engine. Data generated for this analysis and code are supplied in the article and the Supplemental Files, respectively.
