# Supplementary figures and images for: Haiti has more forest than previously reported: land change 2000–2015
Source: PeerJ. 2020 Oct 26;8:e9919. doi: 10.7717/peerj.9919 (PMC7594639; doi:10.7717/peerj.9919)

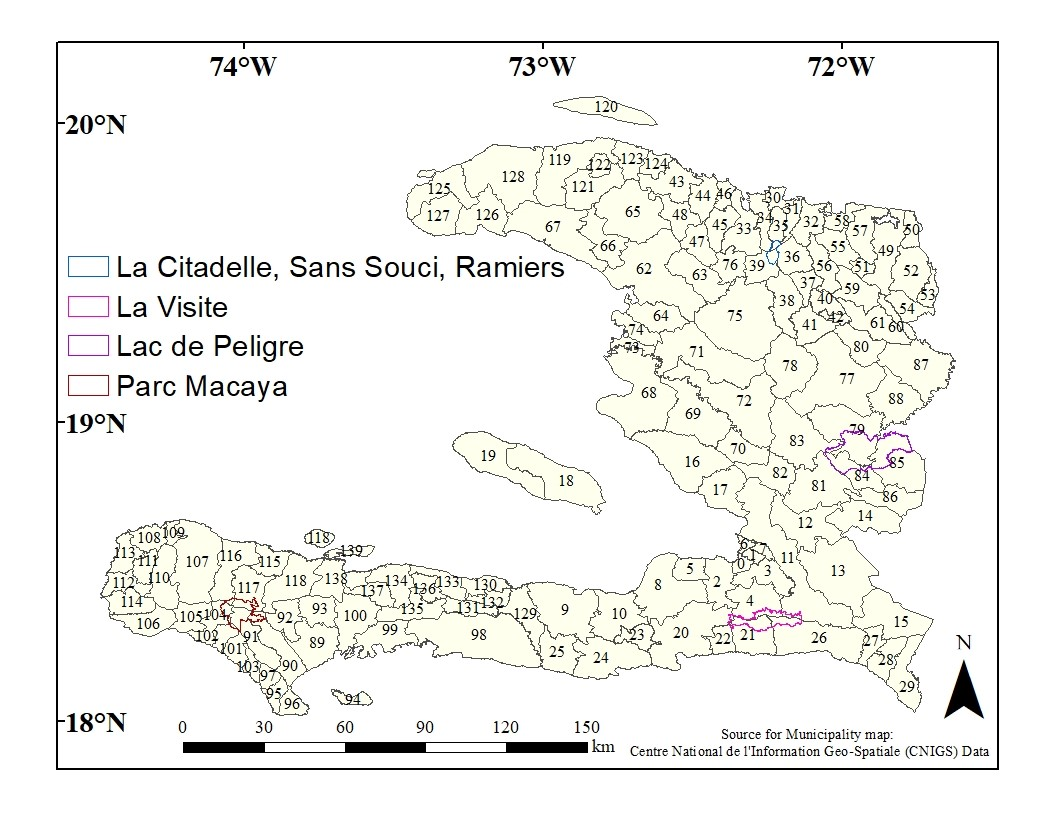

Supplement: Supplemental Information 1 — The polygon shapefile is from CNIGS, 2018. Open source Haitian Centre for Geospatial Information. http://haitidata.org/people/profile/cnigs/?limit=20&offset=40 [file peerj-08-9919-s001.png]
