# Supplementary material for: Haiti has more forest than previously reported: land change 2000–2015
Source: PeerJ. 2020 Oct 26;8:e9919. doi: 10.7717/peerj.9919 (PMC7594639; doi:10.7717/peerj.9919)
Supplement: Supplemental Information 2 — An average of 156 points per class was distributed randomly according to the validated and classified training point’s information by GEE to generate the error matrix with producer’s and user’s accuracy. (A) is for 2000 and (B) for 2015. [file peerj-08-9919-s002.docx]

**Table S1:**  Accuracy assessment achieved by GEE classifier for 2000 and 2015 image classifications. An average of 156 points per class was distributed randomly according to the validated and classified training point’s information by GEE to generate the error matrix with producer’s and user’s accuracy. A) is for 2000 and B for 2015.

| **A) 2000**  **Classified Data** | **Reference Data** | | | | | | | |
| --- | --- | --- | --- | --- | --- | --- | --- | --- |
|  | **Forest** | **Shrubs** | **Ag/Pasture** | **Plantation** | **Urban/infrast** | **Barren** | **Water** | **Producer's accuracy** |
| Forest | 154 | 0 | 0 | 2 | 0 | 0 | 0 | 0.99 |
| Shrubs | 0 | 138 | 6 | 1 | 0 | 0 | 0 | 0.95 |
| Ag/Pasture | 0 | 11 | 150 | 9 | 4 | 1 | 2 | 0.90 |
| Plantation | 1 | 0 | 6 | 139 | 0 | 0 | 0 | 0.95 |
| Urban/infrast | 0 | 3 | 9 | 2 | 159 | 4 | 0 | 0.90 |
| Barren land | 0 | 0 | 4 | 0 | 1 | 140 | 1 | 0.96 |
| Water | 0 | 0 | 3 | 1 | 1 | 2 | 139 | 0.95 |
| User's accuracy | 0.99 | 0.91 | 0.90 | 0.90 | 0.98 | 0.94 | 0.99 |  |
| **Overall accuracy: 0.94** | | | | | | | | |
| **B) 2015**  **Classified Data** | **Reference Data** | | | | | | | |
|  | **Forest** | **Shrubs** | **Ag/Pasture** | **Plant** | **Urban/infrast** | **Barren** | **Water** | **Producer's accuracy** |
| Forest | 152 | 6 | 3 | 1 | 0 | 0 | 1 | 0.93 |
| Shrubs | 5 | 121 | 11 | 5 | 5 | 1 | 0 | 0.82 |
| Ag/Pasture | 2 | 0 | 155 | 7 | 0 | 0 | 0 | 0.94 |
| Plantation | 7 | 5 | 11 | 143 | 1 | 1 | 0 | 0.85 |
| Urban/infrast | 0 | 1 | 2 | 0 | 154 | 2 | 0 | 0.97 |
| Barren land | 0 | 1 | 2 | 0 | 5 | 139 | 0 | 0.95 |
| Water | 0 | 2 | 0 | 2 | 0 | 0 | 153 | 0.99 |
| User's accuracy | 0.91 | 0.89 | 0.87 | 0.9 | 0.9 | 0.97 | 0.99 |  |
| **Overall accuracy: 0.92** | | | | | | | | |
