# Supplementary material for: Haiti has more forest than previously reported: land change 2000–2015
Source: PeerJ. 2020 Oct 26;8:e9919. doi: 10.7717/peerj.9919 (PMC7594639; doi:10.7717/peerj.9919)
Supplement: Supplemental Information 3 — The accuracy of the classified 2000 and 2015 classified images were evaluated by exporting them into google earth and by choosing 50 polygons for forest and non-forest areas randomly. Error Matrix was calculated according to [38-39] in the main reference of the article. (A) is for 2000 and (B) for 2015. [file peerj-08-9919-s003.docx]

**Table S2:** classification validation of forest and non-forest areas. The accuracy of the classified 2000 and 2015 classified images were evaluated by exporting them into google earth and by choosing 50 polygons for forest and non-forest areas randomly. Error Matrix was calculated according to [38-39] in the main reference of the article. A) is for 2000 and B for 2015.

| **A) 2000** | | |
| --- | --- | --- |
| **Classified data** | **Reference Data** | |
|  | **Forest** | **Non-Forest** |
| Forest | 45 | 5 |
| Non-Forest | 4 | 46 |
| Accuracy: 0.91 | | |
| **B) 2015** | | |
| **Classified data** | **Reference data** | |
|  | **Forest** | **Non-Forest** |
| Forest | 47 | 3 |
| Non-Forest | 4 | 46 |
| Accuracy 0.93 | | |
