# Supplementary material for: Haiti has more forest than previously reported: land change 2000–2015
Source: PeerJ. 2020 Oct 26;8:e9919. doi: 10.7717/peerj.9919 (PMC7594639; doi:10.7717/peerj.9919)
Supplement: Supplemental Information 4 — This data was extracted from the land use/cover maps using the municipality layer of Haiti through ArcGIS. Empty box represents no data. [file peerj-08-9919-s004.docx]

**Table S3:** Forest Cover Areas in km^2^ by municipalities for the years 2000 and 2015. This data was extracted from the land use/cover maps using the municipality layer of Haiti through ArcGIS. Empty box represents no data.

| Municipalities | Forest (2000) | Forest (2015) |
| --- | --- | --- |
| Abricots | 72.47 | 62.58 |
| Acul-du-Nord | 95.33 | 123.41 |
| Anse A Foleur | 58.86 | 61.15 |
| Anse A Veau | 29.09 | 0.84 |
| Anse d'Hainault | 80.56 | 90.57 |
| Anse Rouge | 7.27 | 8.64 |
| Anse-Ã -Galet | 21.12 | 6.58 |
| Anse-a-Pitres | 45.42 | 41.71 |
| Aquin | 78.30 | 1.97 |
| Arcahaie | 146.69 | 38.11 |
| Arnaud | 15.46 |  |
| Arniquet | 0.03 | 0.81 |
| Bahon | 40.63 | 45.22 |
| Baie-de-Henne | 0.23 | 0.24 |
| Bainet | 15.15 | 4.80 |
| Baraderes | 124.03 | 16.88 |
| Bas Limbe | 8.88 | 15.70 |
| Bassin-Bleu | 9.61 | 14.74 |
| Beaumont | 161.31 | 120.47 |
| Belladere | 89.43 | 71.58 |
| Belle-Anse | 73.10 | 42.02 |
| Bombardopolis | 42.61 | 13.76 |
| Bonbon | 32.69 | 12.81 |
| Borgne | 168.07 | 167.20 |
| Boucan-Carre | 63.54 | 38.19 |
| Cabaret | 22.76 | 14.15 |
| Camp Perrin | 41.55 | 35.78 |
| Cap-Haitien | 22.55 | 21.24 |
| Capotille | 10.93 | 0.55 |
| Caracol | 16.33 | 15.75 |
| Carice | 30.79 | 22.65 |
| Carrefour | 89.98 | 52.37 |
| Cavaillon | 53.84 | 2.26 |
| Cayes-Jacmel | 47.86 | 42.34 |
| Cerca Carvajal | 23.28 | 20.90 |
| Cerca-la-Source | 37.05 | 13.66 |
| Chambellan | 63.89 | 66.18 |
| Chamsolme | 6.64 | 5.23 |
| Chantal | 57.04 | 92.35 |
| Chardonnieres | 64.69 | 62.71 |
| Cite Soleil | 0.27 | 0.28 |
| Corail | 97.08 | 78.04 |
| Cornillon/Grd Bois | 79.70 | 51.36 |
| Coteaux | 17.38 | 26.97 |
| Cotes-de-Fer | 21.36 | 0.38 |
| Croix-Des-Bouquets | 160.48 | 84.98 |
| Dame-Marie | 71.39 | 86.76 |
| Delmas |  |  |
| Desdunes | 4.84 | 22.62 |
| Dessalines/Marchandes | 69.37 | 78.22 |
| Dondon | 96.28 | 94.36 |
| Ennery | 22.55 | 89.90 |
| Ferrier | 5.07 | 0.63 |
| Fonds Verrettes | 194.10 | 109.57 |
| Fonds-des-Negres | 15.09 | 0.80 |
| Fort Liberte | 74.16 | 50.51 |
| Ganthier | 46.17 | 16.90 |
| Gonaives | 15.26 | 43.07 |
| Grand Boucan | 17.64 | 1.70 |
| Grande Saline | 0.92 | 3.34 |
| Grande-Riviere-du-Nord | 98.15 | 107.71 |
| Grand-Goave | 70.42 | 10.21 |
| Grand-Gosier | 19.29 | 18.22 |
| Gressier | 23.67 | 17.56 |
| Gros Morne | 55.01 | 93.01 |
| Hinche | 54.07 | 57.14 |
| Ile-a-Vache | 2.36 | 0.88 |
| Jacmel | 151.69 | 100.54 |
| Jean-Rabel | 3.74 | 18.68 |
| Jeremie | 326.55 | 226.22 |
| Kenscoff | 125.38 | 82.47 |
| La Chapelle | 24.98 | 20.60 |
| La Tortue | 3.01 | 12.01 |
| La Vallee | 12.66 | 1.61 |
| La Victoire | 7.48 | 2.76 |
| Lascahobas | 52.39 | 58.25 |
| L'Asile | 49.21 | 4.90 |
| Leogane | 119.08 | 66.34 |
| Les Anglais | 72.83 | 78.65 |
| Les Cayes | 0.23 | 0.26 |
| Les Irois | 108.31 | 116.10 |
| L'Estere | 2.17 | 8.50 |
| Limbe | 60.12 | 86.16 |
| Limonade | 45.80 | 37.13 |
| Maissade | 40.25 | 70.68 |
| Maniche | 44.10 | 19.07 |
| Marigot | 89.49 | 66.38 |
| Marmelade | 71.04 | 77.13 |
| Milot | 20.32 | 23.05 |
| Miragoane | 48.98 | 0.14 |
| Mirebalais | 32.28 | 16.81 |
| Mole-Saint-Nicolas | 12.12 | 8.09 |
| Mombin Crochu | 124.98 | 94.76 |
| Mont Organise | 94.59 | 35.90 |
| Moron | 165.85 | 156.25 |
| Ouanaminthe | 30.20 | 21.69 |
| Paillant | 32.76 | 5.23 |
| Perches | 10.15 | 8.66 |
| Pestel | 202.04 | 118.53 |
| Petion-Ville | 41.07 | 15.33 |
| Petite Riviere de l'Art | 155.76 | 141.88 |
| Petite-Riviere-de-Nippes | 23.67 | 3.42 |
| Petit-Goave | 144.19 | 22.78 |
| Petit-Trou-de-Nippes | 43.93 | 8.05 |
| Pignon | 23.29 | 20.81 |
| Pilate | 113.41 | 128.37 |
| Plaine-du-Nord | 30.17 | 38.17 |
| Plaisance | 95.10 | 118.42 |
| Plaisance du Sud | 36.45 | 0.70 |
| Pointe A Raquette | 9.43 | 5.58 |
| Port Margot | 70.50 | 100.75 |
| Port-a-Piment | 23.64 | 18.27 |
| Port-au-Prince | 9.07 | 5.48 |
| Port-de-Paix | 23.13 | 24.78 |
| Port-Salut | 0.20 | 0.20 |
| Quartier Morin | 5.48 | 5.23 |
| Ranquitte | 34.58 | 30.10 |
| Roche-a-Bateau | 3.37 | 5.90 |
| Roseaux | 187.12 | 141.15 |
| Saint Raphael | 28.72 | 27.46 |
| Sainte Suzanne | 80.61 | 83.07 |
| Saint-Louis-du-Nord | 80.04 | 85.45 |
| Saint-Louis-du-Sud | 34.27 | 8.18 |
| Saint-Marc | 72.13 | 31.70 |
| Saint-Michel de l'Attal | 97.89 | 146.54 |
| Saut d'Eau | 45.42 | 26.28 |
| Savanette | 106.01 | 92.80 |
| St. Jean du Sud | 0.44 | 0.46 |
| Tabarre |  |  |
| Terre Neuve | 17.82 | 48.87 |
| Terrier Rouge | 13.82 | 0.91 |
| Thiotte | 48.70 | 53.69 |
| Thomassique | 0.70 | 0.98 |
| Thomazeau | 14.93 | 5.94 |
| Thomonde | 23.38 | 32.62 |
| Tiburon | 56.72 | 67.58 |
| Torbeck | 24.27 | 36.25 |
| Trou du Nord | 32.64 | 16.81 |
| Valliere | 152.13 | 135.10 |
| Verrettes | 26.60 | 9.85 |
| Grand Total | 7620.07 | 6005.43 |
