# Supplementary material for: Haiti has more forest than previously reported: land change 2000–2015
Source: PeerJ. 2020 Oct 26;8:e9919. doi: 10.7717/peerj.9919 (PMC7594639; doi:10.7717/peerj.9919)
Supplement: Supplemental Information 5 [file peerj-08-9919-s005.docx]

**Table S4:** Haiti departments and municipalities.

| **Numbers** | **Departments** | **Municipalities** |
| --- | --- | --- |
| 0 | West | Port-au-Prince |
| 1 | West | Delmas |
| 2 | West | Carrefour |
| 3 | West | Petion-Ville |
| 4 | West | Kenscoff |
| 5 | West | Gressier |
| 6 | West | Cite Soleil |
| 7 | West | Tabarre |
| 8 | West | Leogane |
| 9 | West | Petit-Goave |
| 10 | West | Grand-Goave |
| 11 | West | Croix-Des-Bouquets |
| 12 | West | Thomazeau |
| 13 | West | Ganthier |
| 14 | West | Cornillon / Grand Bois |
| 15 | West | Fonds-Verrettes |
| 16 | West | Arcahaie |
| 17 | West | Cabaret |
| 18 | West | Anse a Galets |
| 19 | West | Pointe a Raquette |
| 20 | South-East | Jacmel |
| 21 | South-East | Marigot |
| 22 | South-East | Cayes-Jacmel |
| 23 | South-East | La Vallee |
| 24 | South-East | Bainet |
| 25 | South-East | Cotes de Fer |
| 26 | South-East | Belle Anse |
| 27 | South-East | Grand Gosier |
| 28 | South-East | Thiotte |
| 29 | South-East | Anse-a-Pitre |
| 30 | North | Cap-Haitien |
| 31 | North | Quartier Morin |
| 32 | North | Limonade |
| 33 | North | Acul du Nord |
| 34 | North | Plaine du Nord |
| 35 | North | Milot |
| 36 | North | Grande Riviere Du Nord |
| 37 | North | Bahon |
| 38 | North | Saint-Raphael |
| 39 | North | Dondon |
| 40 | North | Ranquitte |
| 41 | North | Pignon |
| 42 | North | La Victoire |
| 43 | North | Borgne |
| 44 | North | Port-Margot |
| 45 | North | Limbe |
| 46 | North | Bas Limbe |
| 47 | North | Plaisance |
| 48 | North | Pilate |
| 49 | North-East | Fort-Liberte |
| 50 | North-East | Ferrier |
| 51 | North-East | Perches |
| 52 | North-East | Ouanaminthe |
| 53 | North-East | Capotille |
| 54 | North-East | Mont-Organise |
| 55 | North-East | Trou du Nord |
| 56 | North-East | Sainte Suzanne |
| 57 | North-East | Terrier Rouge |
| 58 | North-East | Caracol |
| 59 | North-East | Vallieres |
| 60 | North-East | Carice |
| 61 | North-East | Mombin Crochu |
| 62 | Artibonite | Gonaives |
| 63 | Artibonite | Ennery |
| 64 | Artibonite | L'Estere |
| 65 | Artibonite | Gros Morne |
| 66 | Artibonite | Terre Neuve |
| 67 | Artibonite | Anse Rouge |
| 68 | Artibonite | Saint-Marc |
| 69 | Artibonite | Verrettes |
| 70 | Artibonite | La Chapelle |
| 71 | Artibonite | Dessalines |
| 72 | Artibonite | Petite Riviere de l'Artibonite |
| 73 | Artibonite | Grande Saline |
| 74 | Artibonite | Desdunes |
| 75 | Artibonite | Saint-Michel de l'Attalaye |
| 76 | Artibonite | Marmelade |
| 77 | Centre | Hinche |
| 78 | Centre | Maissade |
| 79 | Centre | Thomonde |
| 80 | Centre | Cerca Carvajal |
| 81 | Centre | Mirebalais |
| 82 | Centre | Saut d'Eau |
| 83 | Centre | Boucan Carre |
| 84 | Centre | Lascahobas |
| 85 | Centre | Belladere |
| 86 | Centre | Savanette |
| 87 | Centre | Cerca La Source |
| 88 | Centre | Thomassique |
| 89 | South | Les Cayes |
| 90 | South | Torbeck |
| 91 | South | Chantal |
| 92 | South | Camp-Perrin |
| 93 | South | Maniche |
| 94 | South | Ile a Vache |
| 95 | South | Port-Salut |
| 96 | South | Saint Jean du Sud |
| 97 | South | Arniquet |
| 98 | South | Aquin |
| 99 | South | Saint louis du Sud |
| 100 | South | Cavaillon |
| 101 | South | Coteaux |
| 102 | South | Port-a-Piment |
| 103 | South | Roche a Bateau |
| 104 | South | Chardonnieres |
| 105 | South | Les Anglais |
| 106 | South | Tiburon |
| 107 | Grande'Anse | Jeremie |
| 108 | Grande'Anse | Abricots |
| 109 | Grande'Anse | Bonbon |
| 110 | Grande'Anse | Moron |
| 111 | Grande'Anse | Chambellan |
| 112 | Grande'Anse | Anse d'Hainault |
| 113 | Grande'Anse | Dame Marie |
| 114 | Grande'Anse | Les Irois |
| 115 | Grande'Anse | Corail |
| 116 | Grande'Anse | Roseaux |
| 117 | Grande'Anse | Beaumont |
| 118 | Grande'Anse | Pestel |
| 119 | North-West | Port-de-Paix |
| 120 | North-West | La Tortue |
| 121 | North-West | Bassin Bleu |
| 122 | North-West | Chamsolme |
| 123 | North-West | Saint-Louis du Nord |
| 124 | North-West | Anse-a-Foleur |
| 125 | North-West | Mole Saint Nicolas |
| 126 | North-West | Baie de Henne |
| 127 | North-West | Bombardopolis |
| 128 | North-West | Jean Rabel |
| 129 | Nippes | Miragoane |
| 130 | Nippes | Petite Riviere de Nippes |
| 131 | Nippes | Fonds des Negres |
| 132 | Nippes | Paillant |
| 133 | Nippes | Anse-a-Veau |
| 134 | Nippes | Petit Trou de Nippes |
| 135 | Nippes | L'Asile |
| 136 | Nippes | Arnaud |
| 137 | Nippes | Plaisance du Sud |
| 138 | Nippes | Baraderes |
| 139 | Nippes | Grand-Boucan |
