# Supplementary material for: Haiti has more forest than previously reported: land change 2000–2015
Source: PeerJ. 2020 Oct 26;8:e9919. doi: 10.7717/peerj.9919 (PMC7594639; doi:10.7717/peerj.9919)
Supplement: Supplemental Information 6 [file peerj-08-9919-s006.docx]

| Long | Lat | B1 | B2 | B3 | B4 | B5 | B6 | B7 | B8 | class | random |
| --- | --- | --- | --- | --- | --- | --- | --- | --- | --- | --- | --- |
| -72.62003215 | 19.83655319 | 0.01 | 0.02 | 0.04 | 0.02 | 0.41 | 0.16 | 0.06 | 0.30 | 0.00 | 0.49 |
| -72.07700056 | 18.27564055 | 0.02 | 0.03 | 0.05 | 0.03 | 0.35 | 0.17 | 0.08 | 0.30 | 0.00 | 0.00 |
| -72.01097438 | 18.33870228 | 0.02 | 0.03 | 0.05 | 0.05 | 0.19 | 0.19 | 0.12 | 0.29 | 0.00 | 0.45 |
| -73.27086157 | 18.25569795 | 0.05 | 0.07 | 0.11 | 0.11 | 0.34 | 0.32 | 0.19 | 0.30 | 0.00 | 0.35 |
| -72.01717276 | 18.34436167 | 0.01 | 0.02 | 0.05 | 0.05 | 0.20 | 0.16 | 0.09 | 0.29 | 0.00 | 0.07 |
| -72.26052637 | 19.50642232 | 0.01 | 0.01 | 0.04 | 0.02 | 0.32 | 0.14 | 0.05 | 0.30 | 0.00 | 0.01 |
| -72.97280056 | 18.45350698 | 0.02 | 0.02 | 0.04 | 0.03 | 0.31 | 0.14 | 0.07 | 0.30 | 0.00 | 0.27 |
| -71.86275236 | 18.23925878 | 0.01 | 0.02 | 0.04 | 0.03 | 0.33 | 0.15 | 0.07 | 0.30 | 0.00 | 0.10 |
| -72.70357547 | 19.87050951 | 0.01 | 0.02 | 0.04 | 0.02 | 0.37 | 0.16 | 0.06 | 0.30 | 0.00 | 0.03 |
| -72.98331085 | 19.74169109 | 0.03 | 0.03 | 0.05 | 0.05 | 0.28 | 0.20 | 0.10 | 0.30 | 0.00 | 0.11 |
| -72.07834803 | 19.59239109 | 0.01 | 0.02 | 0.05 | 0.04 | 0.34 | 0.19 | 0.09 | 0.30 | 0.00 | 0.57 |
| -72.01313034 | 18.33951077 | 0.01 | 0.02 | 0.05 | 0.04 | 0.20 | 0.14 | 0.08 | 0.29 | 0.00 | 0.08 |
| -72.92024911 | 18.38074344 | 0.01 | 0.02 | 0.04 | 0.03 | 0.33 | 0.12 | 0.05 | 0.30 | 0.00 | 0.33 |
| -72.25863991 | 19.77403044 | 0.02 | 0.02 | 0.05 | 0.04 | 0.31 | 0.14 | 0.07 | 0.30 | 0.00 | 0.50 |
| -73.26843612 | 18.25596745 | 0.03 | 0.04 | 0.07 | 0.06 | 0.36 | 0.23 | 0.11 | 0.30 | 0.00 | 0.45 |
| -72.70276698 | 19.87212647 | 0.01 | 0.01 | 0.03 | 0.02 | 0.30 | 0.12 | 0.05 | 0.30 | 0.00 | 0.25 |
| -73.27032258 | 18.25677593 | 0.02 | 0.03 | 0.06 | 0.06 | 0.31 | 0.21 | 0.11 | 0.30 | 0.00 | 0.12 |
| -72.01124388 | 18.34220571 | 0.01 | 0.02 | 0.05 | 0.04 | 0.19 | 0.14 | 0.08 | 0.29 | 0.00 | 0.51 |
| -73.06388973 | 19.771066 | 0.01 | 0.02 | 0.04 | 0.03 | 0.29 | 0.15 | 0.07 | 0.30 | 0.00 | 0.63 |
| -74.0356872 | 18.33034795 | 0.01 | 0.01 | 0.04 | 0.03 | 0.27 | 0.14 | 0.06 | 0.29 | 0.00 | 0.10 |
| -73.07709496 | 19.7462725 | 0.04 | 0.05 | 0.08 | 0.08 | 0.32 | 0.27 | 0.14 | 0.30 | 0.00 | 0.07 |
| -72.62353558 | 19.83736167 | 0.01 | 0.02 | 0.03 | 0.02 | 0.29 | 0.12 | 0.05 | 0.30 | 0.00 | 0.47 |
| -72.01259135 | 18.34247521 | 0.01 | 0.02 | 0.04 | 0.04 | 0.17 | 0.14 | 0.08 | 0.29 | 0.00 | 0.37 |
| -72.61895417 | 19.83951763 | 0.02 | 0.02 | 0.06 | 0.04 | 0.38 | 0.18 | 0.08 | 0.30 | 0.00 | 0.35 |
| -71.85844045 | 18.24545716 | 0.01 | 0.02 | 0.05 | 0.03 | 0.38 | 0.17 | 0.08 | 0.30 | 0.00 | 0.40 |
| -71.86329135 | 18.24626564 | 0.02 | 0.02 | 0.05 | 0.03 | 0.39 | 0.19 | 0.08 | 0.30 | 0.00 | 0.07 |
| -74.03649569 | 18.32873098 | 0.00 | 0.01 | 0.04 | 0.02 | 0.30 | 0.13 | 0.05 | 0.29 | 0.00 | 0.51 |
| -74.03784316 | 18.39044524 | 0.01 | 0.00 | 0.02 | 0.01 | 0.16 | 0.07 | 0.03 | 0.29 | 0.00 | 0.10 |
| -71.85790146 | 18.24357069 | 0.02 | 0.03 | 0.06 | 0.05 | 0.37 | 0.20 | 0.10 | 0.30 | 0.00 | 0.25 |
| -71.85978792 | 18.24195373 | 0.02 | 0.02 | 0.05 | 0.04 | 0.35 | 0.20 | 0.10 | 0.30 | 0.00 | 0.11 |
| -74.03703468 | 18.39367918 | 0.00 | 0.01 | 0.03 | 0.02 | 0.21 | 0.11 | 0.05 | 0.29 | 0.00 | 0.69 |
| -71.86194388 | 18.24384019 | 0.02 | 0.02 | 0.05 | 0.04 | 0.36 | 0.19 | 0.09 | 0.30 | 0.00 | 0.19 |
| -71.86140489 | 18.2481521 | 0.01 | 0.02 | 0.04 | 0.03 | 0.38 | 0.17 | 0.07 | 0.30 | 0.00 | 0.14 |
| -72.0104354 | 18.34058875 | 0.01 | 0.02 | 0.04 | 0.04 | 0.17 | 0.16 | 0.09 | 0.29 | 0.00 | 0.19 |
| -72.70168901 | 19.87185698 | 0.01 | 0.02 | 0.03 | 0.02 | 0.31 | 0.13 | 0.05 | 0.30 | 0.00 | 0.15 |
| -72.2791215 | 18.34948207 | 0.01 | 0.01 | 0.03 | 0.03 | 0.20 | 0.11 | 0.05 | 0.29 | 0.00 | 0.31 |
| -73.37192204 | 19.72282647 | 0.03 | 0.04 | 0.05 | 0.05 | 0.28 | 0.20 | 0.10 | 0.30 | 0.00 | 0.33 |
| -73.42393449 | 18.34921257 | 0.02 | 0.02 | 0.05 | 0.03 | 0.29 | 0.13 | 0.07 | 0.29 | 0.00 | 0.02 |
| -72.56289929 | 19.72363496 | 0.01 | 0.02 | 0.04 | 0.02 | 0.41 | 0.17 | 0.07 | 0.30 | 0.00 | 0.23 |
| -71.85924893 | 18.24410968 | 0.02 | 0.03 | 0.06 | 0.04 | 0.38 | 0.20 | 0.10 | 0.30 | 0.00 | 0.41 |
| -72.28181644 | 18.34517015 | 0.01 | 0.01 | 0.04 | 0.03 | 0.21 | 0.10 | 0.05 | 0.29 | 0.00 | 0.14 |
| -72.01070489 | 18.34004976 | 0.01 | 0.02 | 0.04 | 0.04 | 0.18 | 0.13 | 0.08 | 0.29 | 0.00 | 0.58 |
| -72.01474731 | 18.33978026 | 0.02 | 0.03 | 0.05 | 0.05 | 0.19 | 0.17 | 0.11 | 0.29 | 0.00 | 0.17 |
| -72.62461355 | 19.83736167 | 0.01 | 0.02 | 0.04 | 0.02 | 0.39 | 0.15 | 0.06 | 0.30 | 0.00 | 0.47 |
| -71.86032691 | 18.25165553 | 0.02 | 0.03 | 0.05 | 0.04 | 0.34 | 0.20 | 0.10 | 0.30 | 0.00 | 0.29 |
| -71.85709298 | 18.24033676 | 0.01 | 0.02 | 0.04 | 0.03 | 0.35 | 0.16 | 0.07 | 0.30 | 0.00 | 0.31 |
| -74.03622619 | 18.33169542 | 0.00 | 0.01 | 0.03 | 0.02 | 0.28 | 0.11 | 0.04 | 0.29 | 0.00 | 0.51 |
| -74.03730417 | 18.38909777 | 0.01 | 0.00 | 0.01 | 0.01 | 0.14 | 0.06 | 0.03 | 0.29 | 0.00 | 0.04 |
| -71.86302186 | 18.25111654 | 0.02 | 0.02 | 0.05 | 0.03 | 0.36 | 0.18 | 0.09 | 0.30 | 0.00 | 0.03 |
| -74.42241193 | 18.48503784 | 0.01 | 0.01 | 0.03 | 0.01 | 0.29 | 0.11 | 0.04 | 0.30 | 0.00 | 0.60 |
| -72.61572023 | 19.83924813 | 0.01 | 0.01 | 0.03 | 0.02 | 0.31 | 0.12 | 0.05 | 0.30 | 0.00 | 0.02 |
| -72.01690327 | 18.34382268 | 0.01 | 0.02 | 0.05 | 0.05 | 0.20 | 0.17 | 0.10 | 0.29 | 0.00 | 0.66 |
| -72.61598973 | 19.83843965 | 0.01 | 0.01 | 0.03 | 0.02 | 0.29 | 0.12 | 0.05 | 0.30 | 0.00 | 0.30 |
| -72.27804352 | 18.34678712 | 0.01 | 0.02 | 0.04 | 0.03 | 0.23 | 0.12 | 0.06 | 0.29 | 0.00 | 0.18 |
| -72.61949316 | 19.83628369 | 0.01 | 0.02 | 0.04 | 0.02 | 0.48 | 0.18 | 0.07 | 0.30 | 0.00 | 0.23 |
| -74.03676518 | 18.32846149 | 0.00 | 0.01 | 0.03 | 0.02 | 0.31 | 0.13 | 0.05 | 0.29 | 0.00 | 0.13 |
| -72.02148467 | 18.34139723 | 0.01 | 0.02 | 0.05 | 0.05 | 0.21 | 0.15 | 0.09 | 0.29 | 0.00 | 0.16 |
| -72.27696554 | 18.34517015 | 0.01 | 0.02 | 0.04 | 0.03 | 0.21 | 0.14 | 0.07 | 0.29 | 0.00 | 0.14 |
| -72.62030164 | 19.84059561 | 0.01 | 0.02 | 0.04 | 0.02 | 0.33 | 0.12 | 0.05 | 0.30 | 0.00 | 0.70 |
| -73.77050453 | 18.37562304 | 0.02 | 0.03 | 0.05 | 0.03 | 0.36 | 0.16 | 0.07 | 0.30 | 0.00 | 0.23 |
| -72.02471861 | 18.34004976 | 0.01 | 0.02 | 0.05 | 0.04 | 0.22 | 0.13 | 0.07 | 0.29 | 0.00 | 0.69 |
| -72.48393738 | 19.70530933 | 0.01 | 0.02 | 0.06 | 0.03 | 0.36 | 0.16 | 0.07 | 0.30 | 0.00 | 0.46 |
| -72.61787619 | 19.83817016 | 0.02 | 0.02 | 0.05 | 0.04 | 0.38 | 0.17 | 0.08 | 0.30 | 0.00 | 0.65 |
| -71.85790146 | 18.24869109 | 0.04 | 0.05 | 0.10 | 0.10 | 0.37 | 0.29 | 0.17 | 0.30 | 0.00 | 0.25 |
| -72.27696554 | 18.34409218 | 0.01 | 0.01 | 0.03 | 0.03 | 0.22 | 0.11 | 0.06 | 0.29 | 0.00 | 0.10 |
| -72.01286085 | 18.33951077 | 0.01 | 0.02 | 0.05 | 0.04 | 0.20 | 0.15 | 0.09 | 0.29 | 0.00 | 0.00 |
| -72.48366789 | 19.70692629 | 0.02 | 0.02 | 0.04 | 0.02 | 0.33 | 0.15 | 0.06 | 0.30 | 0.00 | 0.50 |
| -72.01366933 | 18.34031925 | 0.01 | 0.03 | 0.05 | 0.05 | 0.21 | 0.16 | 0.10 | 0.30 | 0.00 | 0.03 |
| -72.27642655 | 18.3430142 | 0.01 | 0.02 | 0.04 | 0.04 | 0.24 | 0.14 | 0.07 | 0.29 | 0.00 | 0.61 |
| -72.2343854 | 19.61044723 | 0.01 | 0.01 | 0.03 | 0.02 | 0.27 | 0.11 | 0.05 | 0.30 | 0.00 | 0.52 |
| -71.85844045 | 18.24949958 | 0.02 | 0.03 | 0.07 | 0.06 | 0.38 | 0.23 | 0.12 | 0.30 | 0.00 | 0.41 |
| -72.07861753 | 19.59454705 | 0.01 | 0.02 | 0.05 | 0.03 | 0.38 | 0.19 | 0.08 | 0.30 | 0.00 | 0.27 |
| -74.03407024 | 18.33034795 | 0.00 | 0.01 | 0.03 | 0.02 | 0.26 | 0.10 | 0.04 | 0.29 | 0.00 | 0.60 |
| -72.02417962 | 18.34031925 | 0.01 | 0.02 | 0.05 | 0.04 | 0.22 | 0.14 | 0.08 | 0.29 | 0.00 | 0.33 |
| -71.86275236 | 18.25030806 | 0.02 | 0.03 | 0.05 | 0.04 | 0.40 | 0.22 | 0.10 | 0.30 | 0.00 | 0.28 |
| -72.04627818 | 18.27617954 | 0.03 | 0.03 | 0.05 | 0.04 | 0.39 | 0.19 | 0.08 | 0.30 | 0.00 | 0.23 |
| -71.86059641 | 18.24788261 | 0.03 | 0.04 | 0.07 | 0.06 | 0.40 | 0.23 | 0.12 | 0.30 | 0.00 | 0.05 |
| -72.01366933 | 18.34220571 | 0.01 | 0.02 | 0.04 | 0.04 | 0.18 | 0.14 | 0.08 | 0.29 | 0.00 | 0.32 |
| -73.48268431 | 18.34840409 | 0.01 | 0.01 | 0.03 | 0.02 | 0.27 | 0.11 | 0.04 | 0.29 | 0.00 | 0.50 |
| -72.23249893 | 19.6120642 | 0.01 | 0.01 | 0.03 | 0.02 | 0.33 | 0.12 | 0.04 | 0.30 | 0.00 | 0.58 |
| -72.01609478 | 18.34085824 | 0.01 | 0.02 | 0.05 | 0.04 | 0.20 | 0.14 | 0.08 | 0.29 | 0.00 | 0.45 |
| -71.85736247 | 18.24545716 | 0.02 | 0.02 | 0.05 | 0.03 | 0.37 | 0.17 | 0.08 | 0.30 | 0.00 | 0.27 |
| -74.0359567 | 18.38990625 | 0.01 | 0.00 | 0.01 | 0.01 | 0.09 | 0.05 | 0.02 | 0.29 | 0.00 | 0.60 |
| -72.62164911 | 19.83628369 | 0.01 | 0.01 | 0.04 | 0.02 | 0.33 | 0.13 | 0.06 | 0.30 | 0.00 | 0.60 |
| -73.42339551 | 18.34894308 | 0.02 | 0.03 | 0.05 | 0.04 | 0.30 | 0.16 | 0.08 | 0.29 | 0.00 | 0.30 |
| -72.56182132 | 19.72498243 | 0.02 | 0.02 | 0.05 | 0.03 | 0.43 | 0.19 | 0.07 | 0.30 | 0.00 | 0.27 |
| -73.42339551 | 18.34705662 | 0.03 | 0.03 | 0.06 | 0.05 | 0.27 | 0.16 | 0.09 | 0.29 | 0.00 | 0.06 |
| -73.27167005 | 18.25488947 | 0.01 | 0.02 | 0.05 | 0.03 | 0.30 | 0.14 | 0.06 | 0.30 | 0.00 | 0.57 |
| -72.62488305 | 19.8357447 | 0.01 | 0.02 | 0.04 | 0.02 | 0.35 | 0.16 | 0.06 | 0.30 | 0.00 | 0.24 |
| -71.85897944 | 18.24491817 | 0.01 | 0.02 | 0.06 | 0.04 | 0.37 | 0.18 | 0.09 | 0.30 | 0.00 | 0.29 |
| -72.62407457 | 19.83763117 | 0.02 | 0.02 | 0.05 | 0.02 | 0.40 | 0.17 | 0.06 | 0.30 | 0.00 | 0.43 |
| -74.03730417 | 18.3928707 | 0.01 | 0.00 | 0.02 | 0.01 | 0.15 | 0.07 | 0.03 | 0.29 | 0.00 | 0.69 |
| -72.23384641 | 19.61017774 | 0.01 | 0.02 | 0.04 | 0.02 | 0.33 | 0.14 | 0.06 | 0.30 | 0.00 | 0.14 |
| -74.03622619 | 18.39125373 | 0.00 | 0.01 | 0.03 | 0.02 | 0.21 | 0.10 | 0.05 | 0.29 | 0.00 | 0.28 |
| -72.70276698 | 19.86808405 | 0.01 | 0.02 | 0.04 | 0.02 | 0.41 | 0.15 | 0.06 | 0.30 | 0.00 | 0.16 |
| -72.278852 | 18.34651763 | 0.01 | 0.02 | 0.04 | 0.04 | 0.21 | 0.14 | 0.07 | 0.29 | 0.00 | 0.09 |
| -73.26978359 | 18.25461997 | 0.06 | 0.07 | 0.10 | 0.11 | 0.36 | 0.31 | 0.19 | 0.30 | 0.00 | 0.35 |
| -72.02094568 | 18.34139723 | 0.02 | 0.03 | 0.06 | 0.06 | 0.21 | 0.18 | 0.11 | 0.29 | 0.00 | 0.11 |
| -72.27966049 | 18.34840409 | 0.01 | 0.01 | 0.03 | 0.03 | 0.21 | 0.11 | 0.05 | 0.29 | 0.00 | 0.63 |
| -73.37138305 | 19.72228748 | 0.03 | 0.03 | 0.05 | 0.04 | 0.27 | 0.17 | 0.09 | 0.30 | 0.00 | 0.05 |
| -71.85817095 | 18.23952828 | 0.01 | 0.02 | 0.04 | 0.03 | 0.35 | 0.16 | 0.07 | 0.30 | 0.00 | 0.36 |
| -72.70411446 | 19.87131799 | 0.01 | 0.01 | 0.03 | 0.01 | 0.25 | 0.09 | 0.04 | 0.30 | 0.00 | 0.31 |
| -72.62137962 | 19.83547521 | 0.01 | 0.02 | 0.05 | 0.03 | 0.35 | 0.15 | 0.06 | 0.30 | 0.00 | 0.26 |
| -72.01420832 | 18.3430142 | 0.01 | 0.02 | 0.05 | 0.05 | 0.18 | 0.16 | 0.09 | 0.30 | 0.00 | 0.43 |
| -71.85924893 | 18.25192503 | 0.02 | 0.02 | 0.04 | 0.03 | 0.36 | 0.16 | 0.07 | 0.30 | 0.00 | 0.38 |
| -72.23384641 | 19.60856077 | 0.01 | 0.01 | 0.04 | 0.02 | 0.33 | 0.13 | 0.05 | 0.30 | 0.00 | 0.33 |
| -71.86329135 | 18.25273351 | 0.02 | 0.02 | 0.04 | 0.03 | 0.34 | 0.16 | 0.07 | 0.30 | 0.00 | 0.55 |
| -72.01636428 | 18.34490066 | 0.01 | 0.02 | 0.05 | 0.04 | 0.20 | 0.15 | 0.09 | 0.29 | 0.00 | 0.43 |
| -72.28154695 | 18.34490066 | 0.01 | 0.01 | 0.03 | 0.03 | 0.21 | 0.10 | 0.05 | 0.29 | 0.00 | 0.58 |
| -74.03676518 | 18.39394867 | 0.00 | 0.01 | 0.04 | 0.03 | 0.27 | 0.13 | 0.06 | 0.29 | 0.00 | 0.01 |
| -72.48528485 | 19.70584831 | 0.01 | 0.02 | 0.03 | 0.02 | 0.34 | 0.14 | 0.06 | 0.30 | 0.00 | 0.34 |
| -73.26870561 | 18.25408098 | 0.03 | 0.04 | 0.08 | 0.07 | 0.33 | 0.26 | 0.13 | 0.30 | 0.00 | 0.67 |
| -72.28100796 | 18.34597864 | 0.01 | 0.01 | 0.03 | 0.02 | 0.20 | 0.10 | 0.05 | 0.29 | 0.00 | 0.66 |
| -71.86005742 | 18.25057755 | 0.02 | 0.02 | 0.05 | 0.03 | 0.36 | 0.19 | 0.09 | 0.30 | 0.00 | 0.29 |
| -73.25792583 | 19.76917954 | 0.03 | 0.04 | 0.08 | 0.08 | 0.35 | 0.27 | 0.15 | 0.30 | 0.00 | 0.21 |
| -73.25900381 | 19.76864055 | 0.03 | 0.05 | 0.09 | 0.10 | 0.38 | 0.32 | 0.19 | 0.30 | 0.00 | 0.51 |
| -74.0356872 | 18.33061745 | 0.00 | 0.01 | 0.03 | 0.02 | 0.26 | 0.12 | 0.05 | 0.29 | 0.00 | 0.66 |
| -72.61572023 | 19.83951763 | 0.01 | 0.01 | 0.03 | 0.02 | 0.31 | 0.12 | 0.05 | 0.30 | 0.00 | 0.40 |
| -73.42231753 | 18.3478651 | 0.01 | 0.02 | 0.04 | 0.03 | 0.25 | 0.11 | 0.06 | 0.29 | 0.00 | 0.12 |
| -73.37138305 | 19.72282647 | 0.03 | 0.03 | 0.05 | 0.04 | 0.30 | 0.18 | 0.08 | 0.30 | 0.00 | 0.41 |
| -72.2589094 | 19.77376095 | 0.01 | 0.02 | 0.04 | 0.03 | 0.26 | 0.12 | 0.05 | 0.30 | 0.00 | 0.65 |
| -71.86059641 | 18.24734362 | 0.02 | 0.03 | 0.07 | 0.07 | 0.38 | 0.21 | 0.12 | 0.30 | 0.00 | 0.26 |
| -72.70303648 | 19.86889254 | 0.01 | 0.02 | 0.04 | 0.02 | 0.35 | 0.15 | 0.06 | 0.30 | 0.00 | 0.23 |
| -72.01852023 | 18.34490066 | 0.01 | 0.02 | 0.05 | 0.04 | 0.19 | 0.14 | 0.08 | 0.29 | 0.00 | 0.59 |
| -72.70438395 | 19.86916203 | 0.01 | 0.02 | 0.04 | 0.02 | 0.38 | 0.17 | 0.07 | 0.30 | 0.00 | 0.60 |
| -72.11014839 | 18.28642033 | 0.02 | 0.03 | 0.05 | 0.03 | 0.41 | 0.18 | 0.08 | 0.30 | 0.00 | 0.31 |
| -72.28100796 | 18.34813459 | 0.01 | 0.01 | 0.03 | 0.03 | 0.21 | 0.11 | 0.05 | 0.29 | 0.00 | 0.58 |
| -72.02202366 | 18.34139723 | 0.01 | 0.02 | 0.05 | 0.05 | 0.21 | 0.16 | 0.09 | 0.29 | 0.00 | 0.13 |
| -72.07888702 | 19.59319958 | 0.01 | 0.02 | 0.05 | 0.02 | 0.41 | 0.18 | 0.07 | 0.30 | 0.00 | 0.03 |
| -72.01609478 | 18.34193622 | 0.02 | 0.03 | 0.05 | 0.05 | 0.19 | 0.17 | 0.11 | 0.29 | 0.00 | 0.65 |
| -72.70168901 | 19.86835355 | 0.01 | 0.02 | 0.03 | 0.02 | 0.29 | 0.12 | 0.05 | 0.30 | 0.00 | 0.41 |
| -72.56182132 | 19.72336546 | 0.01 | 0.01 | 0.04 | 0.02 | 0.34 | 0.15 | 0.06 | 0.29 | 0.00 | 0.63 |
| -72.01366933 | 18.34193622 | 0.01 | 0.02 | 0.05 | 0.04 | 0.19 | 0.15 | 0.09 | 0.29 | 0.00 | 0.43 |
| -72.01582529 | 18.34463116 | 0.02 | 0.03 | 0.05 | 0.05 | 0.20 | 0.18 | 0.11 | 0.30 | 0.00 | 0.64 |
| -72.10448901 | 18.26998116 | 0.02 | 0.03 | 0.06 | 0.04 | 0.37 | 0.18 | 0.08 | 0.30 | 0.00 | 0.18 |
| -73.07655597 | 19.74546402 | 0.03 | 0.05 | 0.07 | 0.08 | 0.32 | 0.25 | 0.13 | 0.30 | 0.00 | 0.08 |
| -72.27750453 | 18.34517015 | 0.01 | 0.02 | 0.04 | 0.04 | 0.22 | 0.16 | 0.09 | 0.29 | 0.00 | 0.62 |
| -72.23115146 | 19.60829127 | 0.01 | 0.01 | 0.03 | 0.02 | 0.36 | 0.15 | 0.06 | 0.30 | 0.00 | 0.49 |
| -72.27966049 | 18.34543965 | 0.01 | 0.02 | 0.04 | 0.03 | 0.22 | 0.12 | 0.06 | 0.29 | 0.00 | 0.33 |
| -72.61625922 | 19.83790066 | 0.01 | 0.02 | 0.04 | 0.02 | 0.41 | 0.16 | 0.06 | 0.30 | 0.00 | 0.10 |
| -72.86042132 | 19.68994813 | 0.02 | 0.02 | 0.04 | 0.03 | 0.24 | 0.14 | 0.07 | 0.30 | 0.00 | 0.21 |
| -72.02417962 | 18.34058875 | 0.01 | 0.02 | 0.05 | 0.04 | 0.22 | 0.14 | 0.08 | 0.29 | 0.00 | 0.30 |
| -73.42447348 | 18.34732611 | 0.02 | 0.03 | 0.06 | 0.05 | 0.38 | 0.22 | 0.11 | 0.29 | 0.00 | 0.38 |
| -71.86275236 | 18.25138604 | 0.03 | 0.03 | 0.06 | 0.05 | 0.35 | 0.20 | 0.11 | 0.30 | 0.00 | 0.24 |
| -72.01286085 | 18.33924127 | 0.01 | 0.02 | 0.04 | 0.04 | 0.20 | 0.15 | 0.09 | 0.29 | 0.00 | 0.19 |
| -72.70546193 | 19.87050951 | 0.01 | 0.02 | 0.04 | 0.03 | 0.30 | 0.13 | 0.06 | 0.30 | 0.00 | 0.30 |
| -72.48016446 | 19.57406546 | 0.02 | 0.02 | 0.04 | 0.02 | 0.34 | 0.15 | 0.06 | 0.30 | 0.00 | 0.68 |
| -72.61787619 | 19.83951763 | 0.01 | 0.02 | 0.03 | 0.02 | 0.34 | 0.14 | 0.05 | 0.30 | 0.00 | 0.49 |
| -72.1047585 | 18.26890319 | 0.03 | 0.03 | 0.06 | 0.05 | 0.38 | 0.22 | 0.11 | 0.30 | 0.00 | 0.29 |
| -72.70276698 | 19.87050951 | 0.01 | 0.01 | 0.03 | 0.02 | 0.30 | 0.12 | 0.05 | 0.30 | 0.00 | 0.61 |
| -72.30526247 | 19.09113117 | 0.01 | 0.01 | 0.03 | 0.02 | 0.22 | 0.11 | 0.05 | 0.29 | 0.00 | 0.54 |
| -72.6221881 | 19.83924813 | 0.02 | 0.02 | 0.04 | 0.02 | 0.42 | 0.18 | 0.07 | 0.30 | 0.00 | 0.27 |
| -72.97199208 | 18.45377647 | 0.02 | 0.02 | 0.06 | 0.04 | 0.38 | 0.20 | 0.09 | 0.30 | 0.00 | 0.54 |
| -71.86032691 | 18.25138604 | 0.02 | 0.03 | 0.05 | 0.04 | 0.36 | 0.20 | 0.10 | 0.30 | 0.00 | 0.18 |
| -72.01690327 | 18.34193622 | 0.01 | 0.02 | 0.04 | 0.04 | 0.19 | 0.14 | 0.08 | 0.29 | 0.00 | 0.34 |
| -73.27140056 | 18.25435048 | 0.02 | 0.02 | 0.05 | 0.03 | 0.31 | 0.15 | 0.07 | 0.30 | 0.00 | 0.36 |
| -72.70330597 | 19.87050951 | 0.01 | 0.02 | 0.03 | 0.02 | 0.37 | 0.15 | 0.06 | 0.30 | 0.00 | 0.30 |
| -72.01420832 | 18.34436167 | 0.01 | 0.02 | 0.04 | 0.04 | 0.17 | 0.14 | 0.09 | 0.30 | 0.00 | 0.03 |
| -72.56155182 | 19.72255698 | 0.01 | 0.01 | 0.03 | 0.02 | 0.32 | 0.11 | 0.05 | 0.29 | 0.00 | 0.43 |
| -72.48097294 | 19.57568243 | 0.02 | 0.02 | 0.04 | 0.02 | 0.35 | 0.15 | 0.06 | 0.30 | 0.00 | 0.23 |
| -71.85897944 | 18.24276221 | 0.02 | 0.03 | 0.05 | 0.04 | 0.37 | 0.20 | 0.10 | 0.30 | 0.00 | 0.35 |
| -72.62623052 | 19.8360142 | 0.01 | 0.01 | 0.03 | 0.02 | 0.31 | 0.13 | 0.05 | 0.30 | 0.00 | 0.24 |
| -73.42285652 | 18.3478651 | 0.01 | 0.02 | 0.04 | 0.02 | 0.26 | 0.10 | 0.05 | 0.29 | 0.00 | 0.56 |
| -71.86059641 | 18.23979777 | 0.01 | 0.02 | 0.04 | 0.02 | 0.38 | 0.15 | 0.07 | 0.30 | 0.00 | 0.49 |
| -72.70384496 | 19.86835355 | 0.01 | 0.02 | 0.03 | 0.02 | 0.30 | 0.12 | 0.05 | 0.30 | 0.00 | 0.04 |
| -71.8611354 | 18.24491817 | 0.02 | 0.02 | 0.05 | 0.03 | 0.33 | 0.17 | 0.08 | 0.30 | 0.00 | 0.22 |
| -72.31658124 | 19.7662151 | 0.02 | 0.02 | 0.04 | 0.03 | 0.36 | 0.16 | 0.06 | 0.30 | 0.00 | 0.08 |
| -73.27167005 | 18.25435048 | 0.02 | 0.02 | 0.05 | 0.04 | 0.33 | 0.17 | 0.08 | 0.30 | 0.00 | 0.48 |
| -71.85951843 | 18.24761311 | 0.03 | 0.04 | 0.07 | 0.06 | 0.39 | 0.22 | 0.11 | 0.30 | 0.00 | 0.49 |
| -72.62434406 | 19.83466673 | 0.01 | 0.02 | 0.04 | 0.02 | 0.36 | 0.15 | 0.06 | 0.30 | 0.00 | 0.43 |
| -71.86275236 | 18.24222322 | 0.02 | 0.03 | 0.05 | 0.04 | 0.34 | 0.20 | 0.10 | 0.30 | 0.00 | 0.37 |
| -71.86032691 | 18.24761311 | 0.02 | 0.03 | 0.06 | 0.05 | 0.40 | 0.19 | 0.10 | 0.30 | 0.00 | 0.16 |
| -72.23007348 | 19.60990824 | 0.00 | 0.01 | 0.03 | 0.02 | 0.30 | 0.11 | 0.04 | 0.30 | 0.00 | 0.46 |
| -74.42322042 | 18.48611582 | 0.01 | 0.01 | 0.03 | 0.01 | 0.30 | 0.12 | 0.05 | 0.30 | 0.00 | 0.61 |
| -74.03622619 | 18.3928707 | 0.01 | 0.00 | 0.02 | 0.01 | 0.13 | 0.07 | 0.03 | 0.29 | 0.00 | 0.25 |
| -74.03730417 | 18.39044524 | 0.01 | 0.00 | 0.02 | 0.01 | 0.16 | 0.07 | 0.03 | 0.29 | 0.00 | 0.02 |
| -72.01420832 | 18.34409218 | 0.02 | 0.02 | 0.05 | 0.05 | 0.19 | 0.17 | 0.10 | 0.30 | 0.00 | 0.10 |
| -72.02471861 | 18.34058875 | 0.01 | 0.02 | 0.05 | 0.05 | 0.22 | 0.15 | 0.09 | 0.29 | 0.00 | 0.25 |
| -72.61976265 | 19.8408651 | 0.01 | 0.01 | 0.03 | 0.02 | 0.30 | 0.13 | 0.05 | 0.30 | 0.00 | 0.08 |
| -72.70303648 | 19.87158748 | 0.01 | 0.01 | 0.03 | 0.02 | 0.29 | 0.11 | 0.04 | 0.30 | 0.00 | 0.07 |
| -72.25702294 | 19.77322196 | 0.02 | 0.02 | 0.05 | 0.03 | 0.28 | 0.13 | 0.06 | 0.30 | 0.00 | 0.07 |
| -72.49390868 | 18.5068669 | 0.01 | 0.01 | 0.03 | 0.02 | 0.19 | 0.08 | 0.03 | 0.30 | 0.00 | 0.30 |
| -72.07673106 | 18.27510156 | 0.02 | 0.03 | 0.06 | 0.04 | 0.37 | 0.18 | 0.08 | 0.30 | 0.00 | 0.01 |
| -72.61652872 | 19.83870914 | 0.01 | 0.02 | 0.03 | 0.02 | 0.34 | 0.14 | 0.06 | 0.30 | 0.00 | 0.62 |
| -71.86140489 | 18.24545716 | 0.02 | 0.02 | 0.05 | 0.03 | 0.39 | 0.19 | 0.09 | 0.30 | 0.00 | 0.59 |
| -74.03460922 | 18.32953947 | 0.00 | 0.01 | 0.03 | 0.02 | 0.22 | 0.11 | 0.05 | 0.29 | 0.00 | 0.19 |
| -72.56155182 | 19.72417395 | 0.01 | 0.02 | 0.03 | 0.02 | 0.29 | 0.12 | 0.05 | 0.30 | 0.00 | 0.37 |
| -72.61814568 | 19.83843965 | 0.01 | 0.01 | 0.03 | 0.02 | 0.30 | 0.12 | 0.05 | 0.30 | 0.00 | 0.57 |
| -73.27167005 | 18.25435048 | 0.02 | 0.02 | 0.05 | 0.04 | 0.33 | 0.17 | 0.08 | 0.30 | 0.00 | 0.02 |
| -71.85817095 | 18.24949958 | 0.03 | 0.04 | 0.08 | 0.07 | 0.34 | 0.26 | 0.14 | 0.30 | 0.00 | 0.62 |
| -73.77158251 | 18.37508405 | 0.02 | 0.02 | 0.05 | 0.03 | 0.37 | 0.16 | 0.06 | 0.30 | 0.00 | 0.53 |
| -72.62084063 | 19.83682268 | 0.01 | 0.02 | 0.04 | 0.03 | 0.28 | 0.14 | 0.06 | 0.30 | 0.00 | 0.39 |
| -71.85736247 | 18.24680463 | 0.01 | 0.02 | 0.05 | 0.03 | 0.40 | 0.18 | 0.08 | 0.30 | 0.00 | 0.21 |
| -72.27966049 | 18.34517015 | 0.01 | 0.01 | 0.03 | 0.03 | 0.22 | 0.11 | 0.05 | 0.29 | 0.00 | 0.08 |
| -72.23330742 | 19.61152521 | 0.01 | 0.01 | 0.03 | 0.02 | 0.31 | 0.12 | 0.05 | 0.30 | 0.00 | 0.25 |
| -72.62596103 | 19.83628369 | 0.01 | 0.01 | 0.03 | 0.02 | 0.36 | 0.14 | 0.06 | 0.30 | 0.00 | 0.02 |
| -73.2692446 | 18.25381149 | 0.04 | 0.05 | 0.09 | 0.09 | 0.35 | 0.25 | 0.15 | 0.30 | 0.00 | 0.44 |
| -74.03784316 | 18.39367918 | 0.01 | 0.01 | 0.00 | 0.00 | 0.03 | 0.01 | 0.00 | 0.29 | 0.00 | 0.45 |
| -72.01582529 | 18.34382268 | 0.01 | 0.02 | 0.05 | 0.04 | 0.20 | 0.16 | 0.09 | 0.29 | 0.00 | 0.54 |
| -71.8611354 | 18.24896059 | 0.02 | 0.02 | 0.05 | 0.03 | 0.43 | 0.20 | 0.08 | 0.30 | 0.00 | 0.43 |
| -73.42393449 | 18.34732611 | 0.02 | 0.02 | 0.05 | 0.03 | 0.34 | 0.16 | 0.07 | 0.29 | 0.00 | 0.70 |
| -72.01663377 | 18.34517015 | 0.01 | 0.02 | 0.05 | 0.05 | 0.19 | 0.16 | 0.09 | 0.29 | 0.00 | 0.17 |
| -72.27642655 | 18.34732611 | 0.01 | 0.01 | 0.04 | 0.03 | 0.23 | 0.11 | 0.05 | 0.29 | 0.00 | 0.06 |
| -72.62084063 | 19.8360142 | 0.02 | 0.02 | 0.05 | 0.04 | 0.33 | 0.17 | 0.07 | 0.30 | 0.00 | 0.01 |
| -72.62272709 | 19.83790066 | 0.01 | 0.02 | 0.04 | 0.02 | 0.40 | 0.17 | 0.06 | 0.30 | 0.00 | 0.59 |
| -72.62299659 | 19.8357447 | 0.01 | 0.02 | 0.04 | 0.02 | 0.36 | 0.14 | 0.06 | 0.30 | 0.00 | 0.02 |
| -71.86302186 | 18.24087575 | 0.01 | 0.02 | 0.04 | 0.03 | 0.32 | 0.15 | 0.07 | 0.30 | 0.00 | 0.06 |
| -72.97387854 | 18.45458496 | 0.02 | 0.04 | 0.08 | 0.06 | 0.40 | 0.24 | 0.12 | 0.30 | 0.00 | 0.65 |
| -71.85736247 | 18.2433012 | 0.01 | 0.02 | 0.05 | 0.03 | 0.35 | 0.17 | 0.08 | 0.30 | 0.00 | 0.21 |
| -72.70034153 | 19.87185698 | 0.01 | 0.01 | 0.04 | 0.02 | 0.36 | 0.14 | 0.05 | 0.30 | 0.00 | 0.50 |
| -72.62272709 | 19.83466673 | 0.01 | 0.02 | 0.04 | 0.02 | 0.33 | 0.14 | 0.06 | 0.30 | 0.00 | 0.13 |
| -72.62164911 | 19.8357447 | 0.01 | 0.01 | 0.04 | 0.02 | 0.35 | 0.16 | 0.06 | 0.30 | 0.00 | 0.00 |
| -72.55993485 | 19.72390445 | 0.01 | 0.01 | 0.03 | 0.02 | 0.30 | 0.12 | 0.05 | 0.29 | 0.00 | 0.21 |
| -72.56128233 | 19.72255698 | 0.01 | 0.01 | 0.03 | 0.02 | 0.30 | 0.14 | 0.06 | 0.29 | 0.00 | 0.58 |
| -72.23249893 | 19.60909976 | 0.01 | 0.01 | 0.03 | 0.02 | 0.29 | 0.12 | 0.05 | 0.30 | 0.00 | 0.64 |
| -72.01717276 | 18.34328369 | 0.02 | 0.03 | 0.05 | 0.05 | 0.21 | 0.18 | 0.11 | 0.29 | 0.00 | 0.04 |
| -72.01232186 | 18.34247521 | 0.01 | 0.02 | 0.04 | 0.04 | 0.18 | 0.13 | 0.08 | 0.29 | 0.00 | 0.35 |
| -72.23465489 | 19.60909976 | 0.01 | 0.01 | 0.03 | 0.02 | 0.34 | 0.14 | 0.06 | 0.30 | 0.00 | 0.17 |
| -72.23088197 | 19.61152521 | 0.01 | 0.01 | 0.03 | 0.01 | 0.23 | 0.09 | 0.04 | 0.30 | 0.00 | 0.39 |
| -72.23357691 | 19.61044723 | 0.01 | 0.01 | 0.03 | 0.02 | 0.33 | 0.13 | 0.05 | 0.30 | 0.00 | 0.09 |
| -72.01663377 | 18.3430142 | 0.01 | 0.03 | 0.05 | 0.05 | 0.21 | 0.16 | 0.09 | 0.30 | 0.00 | 0.66 |
| -72.02256265 | 18.34328369 | 0.01 | 0.02 | 0.04 | 0.04 | 0.20 | 0.16 | 0.09 | 0.29 | 0.00 | 0.65 |
| -71.85897944 | 18.24384019 | 0.02 | 0.03 | 0.05 | 0.04 | 0.36 | 0.20 | 0.09 | 0.30 | 0.00 | 0.19 |
| -71.85790146 | 18.24141474 | 0.02 | 0.02 | 0.05 | 0.04 | 0.34 | 0.19 | 0.09 | 0.30 | 0.00 | 0.57 |
| -73.2695141 | 18.25542846 | 0.03 | 0.04 | 0.07 | 0.07 | 0.36 | 0.23 | 0.12 | 0.30 | 0.00 | 0.27 |
| -71.85628449 | 18.23979777 | 0.02 | 0.02 | 0.04 | 0.03 | 0.35 | 0.17 | 0.08 | 0.30 | 0.00 | 0.34 |
| -71.86059641 | 18.25057755 | 0.01 | 0.02 | 0.04 | 0.03 | 0.36 | 0.17 | 0.08 | 0.30 | 0.00 | 0.35 |
| -72.48474587 | 19.70719579 | 0.02 | 0.02 | 0.05 | 0.03 | 0.36 | 0.16 | 0.07 | 0.30 | 0.00 | 0.47 |
| -72.01124388 | 18.34112773 | 0.01 | 0.02 | 0.05 | 0.04 | 0.21 | 0.16 | 0.09 | 0.29 | 0.00 | 0.34 |
| -72.01905922 | 18.34597864 | 0.01 | 0.02 | 0.05 | 0.04 | 0.19 | 0.15 | 0.09 | 0.29 | 0.00 | 0.42 |
| -71.86167438 | 18.25219452 | 0.02 | 0.02 | 0.04 | 0.03 | 0.36 | 0.16 | 0.07 | 0.30 | 0.00 | 0.44 |
| -73.26870561 | 18.25515896 | 0.03 | 0.04 | 0.08 | 0.07 | 0.38 | 0.26 | 0.13 | 0.30 | 0.00 | 0.46 |
| -72.01447781 | 18.34463116 | 0.02 | 0.03 | 0.05 | 0.05 | 0.19 | 0.18 | 0.11 | 0.30 | 0.00 | 0.14 |
| -71.85763197 | 18.24087575 | 0.02 | 0.02 | 0.04 | 0.03 | 0.36 | 0.17 | 0.08 | 0.30 | 0.00 | 0.50 |
| -72.01878973 | 18.34355319 | 0.01 | 0.02 | 0.05 | 0.04 | 0.20 | 0.14 | 0.08 | 0.29 | 0.00 | 0.29 |
| -71.86194388 | 18.24869109 | 0.02 | 0.03 | 0.05 | 0.04 | 0.38 | 0.20 | 0.10 | 0.30 | 0.00 | 0.04 |
| -71.85897944 | 18.24788261 | 0.03 | 0.03 | 0.07 | 0.05 | 0.42 | 0.21 | 0.10 | 0.30 | 0.00 | 0.51 |
| -72.01798124 | 18.34166672 | 0.01 | 0.02 | 0.04 | 0.04 | 0.19 | 0.13 | 0.07 | 0.29 | 0.00 | 0.15 |
| -74.42510688 | 18.48665481 | 0.01 | 0.01 | 0.03 | 0.01 | 0.32 | 0.12 | 0.05 | 0.30 | 0.00 | 0.13 |
| -71.86032691 | 18.24680463 | 0.03 | 0.03 | 0.07 | 0.06 | 0.41 | 0.23 | 0.11 | 0.30 | 0.00 | 0.36 |
| -72.27992998 | 18.34624813 | 0.01 | 0.02 | 0.04 | 0.03 | 0.24 | 0.14 | 0.07 | 0.29 | 0.00 | 0.02 |
| -72.02067619 | 18.3430142 | 0.01 | 0.02 | 0.05 | 0.05 | 0.20 | 0.18 | 0.11 | 0.29 | 0.00 | 0.17 |
| -71.8611354 | 18.24545716 | 0.01 | 0.02 | 0.05 | 0.03 | 0.35 | 0.17 | 0.08 | 0.30 | 0.00 | 0.37 |
| -74.44181554 | 18.47102412 | 0.01 | 0.02 | 0.04 | 0.02 | 0.40 | 0.16 | 0.06 | 0.30 | 0.00 | 0.62 |
| -71.86275236 | 18.24141474 | 0.02 | 0.02 | 0.04 | 0.03 | 0.30 | 0.16 | 0.07 | 0.30 | 0.00 | 0.00 |
| -73.42312601 | 18.34948207 | 0.03 | 0.03 | 0.05 | 0.04 | 0.28 | 0.14 | 0.07 | 0.29 | 0.00 | 0.65 |
| -74.03541771 | 18.32873098 | 0.00 | 0.01 | 0.03 | 0.02 | 0.29 | 0.12 | 0.05 | 0.29 | 0.00 | 0.09 |
| -72.23169045 | 19.6117947 | 0.01 | 0.01 | 0.03 | 0.02 | 0.38 | 0.16 | 0.06 | 0.30 | 0.00 | 0.46 |
| -72.02337114 | 18.34004976 | 0.01 | 0.02 | 0.05 | 0.04 | 0.23 | 0.14 | 0.08 | 0.29 | 0.00 | 0.69 |
| -72.01582529 | 18.33951077 | 0.01 | 0.02 | 0.04 | 0.04 | 0.19 | 0.15 | 0.09 | 0.29 | 0.00 | 0.49 |
| -72.31631175 | 19.76459813 | 0.02 | 0.02 | 0.05 | 0.03 | 0.32 | 0.14 | 0.06 | 0.30 | 0.00 | 0.63 |
| -72.28073846 | 18.34867358 | 0.01 | 0.01 | 0.03 | 0.02 | 0.21 | 0.11 | 0.05 | 0.29 | 0.00 | 0.56 |
| -72.11014839 | 18.28588134 | 0.03 | 0.03 | 0.06 | 0.04 | 0.41 | 0.19 | 0.09 | 0.30 | 0.00 | 0.53 |
| -71.86167438 | 18.24357069 | 0.02 | 0.02 | 0.05 | 0.03 | 0.40 | 0.19 | 0.09 | 0.30 | 0.00 | 0.20 |
| -72.4831289 | 19.70773478 | 0.01 | 0.02 | 0.04 | 0.02 | 0.35 | 0.15 | 0.06 | 0.30 | 0.00 | 0.62 |
| -72.30445399 | 19.09140066 | 0.01 | 0.01 | 0.02 | 0.01 | 0.21 | 0.09 | 0.04 | 0.29 | 0.00 | 0.52 |
| -72.23088197 | 19.60990824 | 0.01 | 0.01 | 0.03 | 0.02 | 0.28 | 0.11 | 0.04 | 0.30 | 0.00 | 0.61 |
| -74.0359567 | 18.32900048 | 0.00 | 0.01 | 0.03 | 0.02 | 0.27 | 0.11 | 0.05 | 0.29 | 0.00 | 0.19 |
| -72.4831289 | 19.7063873 | 0.01 | 0.02 | 0.04 | 0.02 | 0.28 | 0.12 | 0.06 | 0.30 | 0.00 | 0.18 |
| -72.62164911 | 19.83709218 | 0.02 | 0.03 | 0.06 | 0.04 | 0.28 | 0.15 | 0.07 | 0.30 | 0.00 | 0.05 |
| -72.31712023 | 19.76594561 | 0.02 | 0.02 | 0.04 | 0.02 | 0.32 | 0.14 | 0.05 | 0.30 | 0.00 | 0.46 |
| -72.49202222 | 18.50848387 | 0.01 | 0.02 | 0.04 | 0.02 | 0.33 | 0.13 | 0.05 | 0.30 | 0.00 | 0.17 |
| -72.01555579 | 18.34058875 | 0.01 | 0.02 | 0.05 | 0.04 | 0.19 | 0.15 | 0.09 | 0.29 | 0.00 | 0.48 |
| -72.62326608 | 19.83520572 | 0.01 | 0.01 | 0.03 | 0.02 | 0.33 | 0.14 | 0.06 | 0.30 | 0.00 | 0.14 |
| -72.69980254 | 19.870779 | 0.01 | 0.02 | 0.04 | 0.03 | 0.39 | 0.16 | 0.06 | 0.30 | 0.00 | 0.26 |
| -73.42420399 | 18.3478651 | 0.03 | 0.04 | 0.06 | 0.04 | 0.33 | 0.18 | 0.09 | 0.29 | 0.00 | 0.56 |
| -72.49229171 | 18.5068669 | 0.01 | 0.01 | 0.03 | 0.02 | 0.26 | 0.11 | 0.05 | 0.30 | 0.00 | 0.19 |
| -72.01447781 | 18.33978026 | 0.01 | 0.02 | 0.05 | 0.04 | 0.19 | 0.15 | 0.09 | 0.29 | 0.00 | 0.06 |
| -73.77050453 | 18.37535355 | 0.02 | 0.03 | 0.05 | 0.03 | 0.34 | 0.16 | 0.07 | 0.30 | 0.00 | 0.55 |
| -72.31658124 | 19.76594561 | 0.02 | 0.02 | 0.05 | 0.03 | 0.34 | 0.15 | 0.07 | 0.30 | 0.00 | 0.33 |
| -72.70034153 | 19.870779 | 0.01 | 0.02 | 0.04 | 0.02 | 0.40 | 0.16 | 0.06 | 0.30 | 0.00 | 0.27 |
| -71.86329135 | 18.25300301 | 0.02 | 0.02 | 0.04 | 0.03 | 0.34 | 0.16 | 0.07 | 0.30 | 0.00 | 0.15 |
| -74.34910941 | 18.47587503 | 0.01 | 0.01 | 0.04 | 0.02 | 0.41 | 0.16 | 0.06 | 0.30 | 0.00 | 0.09 |
| -73.42258702 | 18.34813459 | 0.01 | 0.02 | 0.04 | 0.03 | 0.25 | 0.11 | 0.06 | 0.29 | 0.00 | 0.22 |
| -72.28046897 | 18.34651763 | 0.01 | 0.02 | 0.04 | 0.03 | 0.23 | 0.13 | 0.06 | 0.29 | 0.00 | 0.45 |
| -72.62111013 | 19.83493622 | 0.02 | 0.02 | 0.05 | 0.03 | 0.52 | 0.19 | 0.07 | 0.30 | 0.00 | 0.41 |
| -74.03676518 | 18.39044524 | 0.00 | 0.01 | 0.03 | 0.02 | 0.19 | 0.09 | 0.04 | 0.29 | 0.00 | 0.68 |
| -72.01259135 | 18.34058875 | 0.01 | 0.02 | 0.04 | 0.04 | 0.19 | 0.15 | 0.09 | 0.29 | 0.00 | 0.54 |
| -72.9059659 | 18.28372539 | 0.02 | 0.02 | 0.04 | 0.03 | 0.34 | 0.15 | 0.07 | 0.30 | 0.00 | 0.25 |
| -72.62461355 | 19.83709218 | 0.01 | 0.02 | 0.04 | 0.02 | 0.39 | 0.16 | 0.06 | 0.30 | 0.00 | 0.43 |
| -72.27777402 | 18.34517015 | 0.01 | 0.02 | 0.04 | 0.04 | 0.23 | 0.15 | 0.08 | 0.29 | 0.00 | 0.13 |
| -74.0356872 | 18.33142593 | 0.00 | 0.01 | 0.03 | 0.02 | 0.31 | 0.12 | 0.05 | 0.29 | 0.00 | 0.02 |
| -72.61787619 | 19.83843965 | 0.01 | 0.02 | 0.03 | 0.02 | 0.34 | 0.14 | 0.05 | 0.30 | 0.00 | 0.40 |
| -72.01393882 | 18.33978026 | 0.01 | 0.02 | 0.04 | 0.04 | 0.19 | 0.14 | 0.08 | 0.29 | 0.00 | 0.47 |
| -72.0101659 | 18.34382268 | 0.01 | 0.02 | 0.05 | 0.05 | 0.18 | 0.15 | 0.09 | 0.30 | 0.00 | 0.10 |
| -74.44181554 | 18.47102412 | 0.01 | 0.02 | 0.04 | 0.02 | 0.40 | 0.16 | 0.06 | 0.30 | 0.00 | 0.08 |
| -72.48366789 | 19.70746528 | 0.01 | 0.02 | 0.04 | 0.02 | 0.38 | 0.15 | 0.06 | 0.30 | 0.00 | 0.63 |
| -72.01474731 | 18.34247521 | 0.02 | 0.03 | 0.05 | 0.05 | 0.19 | 0.18 | 0.11 | 0.30 | 0.00 | 0.27 |
| -72.48366789 | 19.70773478 | 0.02 | 0.02 | 0.05 | 0.03 | 0.41 | 0.17 | 0.08 | 0.30 | 0.00 | 0.55 |
| -72.62326608 | 19.83870914 | 0.02 | 0.03 | 0.05 | 0.03 | 0.35 | 0.18 | 0.08 | 0.30 | 0.00 | 0.47 |
| -72.62353558 | 19.8360142 | 0.01 | 0.02 | 0.04 | 0.02 | 0.38 | 0.16 | 0.06 | 0.30 | 0.00 | 0.04 |
| -72.27858251 | 18.34517015 | 0.01 | 0.02 | 0.04 | 0.03 | 0.23 | 0.13 | 0.06 | 0.29 | 0.00 | 0.25 |
| -71.85844045 | 18.24491817 | 0.01 | 0.02 | 0.05 | 0.03 | 0.37 | 0.19 | 0.09 | 0.30 | 0.00 | 0.55 |
| -72.01771175 | 18.3430142 | 0.01 | 0.02 | 0.05 | 0.05 | 0.20 | 0.15 | 0.09 | 0.29 | 0.00 | 0.21 |
| -72.56236031 | 19.72498243 | 0.02 | 0.02 | 0.05 | 0.04 | 0.41 | 0.18 | 0.08 | 0.30 | 0.00 | 0.63 |
| -72.27831301 | 18.34355319 | 0.00 | 0.01 | 0.04 | 0.03 | 0.23 | 0.10 | 0.05 | 0.29 | 0.00 | 0.40 |
| -71.8608659 | 18.25084705 | 0.02 | 0.02 | 0.05 | 0.03 | 0.36 | 0.19 | 0.09 | 0.30 | 0.00 | 0.49 |
| -72.97226157 | 18.45512394 | 0.02 | 0.03 | 0.05 | 0.04 | 0.33 | 0.18 | 0.09 | 0.30 | 0.00 | 0.22 |
| -71.85924893 | 18.25273351 | 0.04 | 0.05 | 0.08 | 0.07 | 0.37 | 0.28 | 0.15 | 0.30 | 0.00 | 0.21 |
| -72.62623052 | 19.83520572 | 0.01 | 0.02 | 0.04 | 0.02 | 0.36 | 0.15 | 0.06 | 0.30 | 0.00 | 0.20 |
| -72.62084063 | 19.83520572 | 0.01 | 0.02 | 0.05 | 0.03 | 0.49 | 0.18 | 0.07 | 0.30 | 0.00 | 0.08 |
| -72.37694803 | 19.51100373 | 0.01 | 0.02 | 0.05 | 0.03 | 0.36 | 0.15 | 0.06 | 0.30 | 0.00 | 0.08 |
| -74.42537637 | 18.48611582 | 0.01 | 0.01 | 0.03 | 0.01 | 0.28 | 0.11 | 0.04 | 0.30 | 0.00 | 0.66 |
| -72.7019585 | 19.86781456 | 0.01 | 0.02 | 0.03 | 0.02 | 0.28 | 0.11 | 0.05 | 0.30 | 0.00 | 0.62 |
| -71.86167438 | 18.25165553 | 0.02 | 0.02 | 0.05 | 0.04 | 0.35 | 0.18 | 0.09 | 0.30 | 0.00 | 0.46 |
| -72.25729244 | 19.50857828 | 0.01 | 0.02 | 0.04 | 0.02 | 0.31 | 0.15 | 0.06 | 0.30 | 0.00 | 0.33 |
| -71.85978792 | 18.25057755 | 0.02 | 0.03 | 0.06 | 0.05 | 0.35 | 0.22 | 0.11 | 0.30 | 0.00 | 0.59 |
| -72.62003215 | 19.83655319 | 0.01 | 0.02 | 0.04 | 0.02 | 0.41 | 0.16 | 0.06 | 0.30 | 0.00 | 0.16 |
| -72.92051861 | 18.38047394 | 0.01 | 0.01 | 0.03 | 0.02 | 0.23 | 0.10 | 0.04 | 0.30 | 0.00 | 0.64 |
| -72.70330597 | 19.86835355 | 0.01 | 0.01 | 0.03 | 0.02 | 0.29 | 0.10 | 0.04 | 0.30 | 0.00 | 0.51 |
| -73.27059208 | 18.25650643 | 0.01 | 0.02 | 0.05 | 0.04 | 0.36 | 0.17 | 0.08 | 0.30 | 0.00 | 0.26 |
| -72.02471861 | 18.34031925 | 0.01 | 0.02 | 0.05 | 0.04 | 0.22 | 0.13 | 0.07 | 0.29 | 0.00 | 0.44 |
| -72.62057114 | 19.83520572 | 0.02 | 0.02 | 0.05 | 0.03 | 0.48 | 0.18 | 0.07 | 0.30 | 0.00 | 0.16 |
| -72.01932872 | 18.34543965 | 0.01 | 0.02 | 0.04 | 0.05 | 0.18 | 0.16 | 0.09 | 0.29 | 0.00 | 0.43 |
| -72.07780904 | 19.59454705 | 0.01 | 0.01 | 0.04 | 0.03 | 0.37 | 0.17 | 0.07 | 0.30 | 0.00 | 0.32 |
| -72.87793847 | 19.68563622 | 0.03 | 0.03 | 0.06 | 0.05 | 0.27 | 0.20 | 0.11 | 0.30 | 0.00 | 0.67 |
| -74.03514821 | 18.32980896 | 0.01 | 0.01 | 0.03 | 0.02 | 0.23 | 0.11 | 0.05 | 0.29 | 0.00 | 0.57 |
| -72.5626298 | 19.72390445 | 0.02 | 0.02 | 0.04 | 0.02 | 0.36 | 0.15 | 0.06 | 0.30 | 0.00 | 0.25 |
| -72.01744225 | 18.34436167 | 0.01 | 0.02 | 0.05 | 0.05 | 0.21 | 0.17 | 0.09 | 0.29 | 0.00 | 0.66 |
| -72.61922366 | 19.83736167 | 0.01 | 0.02 | 0.04 | 0.02 | 0.30 | 0.13 | 0.05 | 0.30 | 0.00 | 0.62 |
| -72.28181644 | 18.34651763 | 0.01 | 0.01 | 0.03 | 0.03 | 0.21 | 0.11 | 0.05 | 0.29 | 0.00 | 0.53 |
| -72.27669605 | 18.34624813 | 0.01 | 0.01 | 0.03 | 0.03 | 0.21 | 0.11 | 0.05 | 0.29 | 0.00 | 0.66 |
| -72.25863991 | 19.77429994 | 0.01 | 0.02 | 0.05 | 0.03 | 0.28 | 0.13 | 0.05 | 0.30 | 0.00 | 0.39 |
| -72.01555579 | 18.34382268 | 0.02 | 0.03 | 0.05 | 0.05 | 0.20 | 0.18 | 0.10 | 0.30 | 0.00 | 0.23 |
| -72.07753955 | 19.59454705 | 0.01 | 0.02 | 0.04 | 0.03 | 0.33 | 0.16 | 0.06 | 0.30 | 0.00 | 0.22 |
| -72.27966049 | 18.34813459 | 0.01 | 0.01 | 0.04 | 0.03 | 0.22 | 0.11 | 0.05 | 0.29 | 0.00 | 0.65 |
| -74.03622619 | 18.3279225 | 0.00 | 0.01 | 0.04 | 0.02 | 0.32 | 0.13 | 0.05 | 0.29 | 0.00 | 0.13 |
| -72.70168901 | 19.86835355 | 0.01 | 0.02 | 0.03 | 0.02 | 0.29 | 0.12 | 0.05 | 0.30 | 0.00 | 0.69 |
| -71.8608659 | 18.2433012 | 0.02 | 0.03 | 0.06 | 0.05 | 0.35 | 0.21 | 0.11 | 0.30 | 0.00 | 0.67 |
| -73.37219153 | 19.72282647 | 0.04 | 0.04 | 0.05 | 0.05 | 0.25 | 0.19 | 0.10 | 0.30 | 0.00 | 0.50 |
| -73.27113106 | 18.25515896 | 0.02 | 0.03 | 0.06 | 0.04 | 0.30 | 0.18 | 0.09 | 0.30 | 0.00 | 0.21 |
| -72.25756193 | 19.50884777 | 0.01 | 0.02 | 0.04 | 0.02 | 0.34 | 0.15 | 0.05 | 0.30 | 0.00 | 0.35 |
| -73.42258702 | 18.34705662 | 0.01 | 0.02 | 0.04 | 0.02 | 0.24 | 0.10 | 0.05 | 0.29 | 0.00 | 0.65 |
| -74.0356872 | 18.32900048 | 0.00 | 0.01 | 0.03 | 0.02 | 0.28 | 0.11 | 0.05 | 0.29 | 0.00 | 0.61 |
| -72.37748702 | 19.51127322 | 0.01 | 0.02 | 0.04 | 0.03 | 0.33 | 0.17 | 0.07 | 0.30 | 0.00 | 0.15 |
| -71.8608659 | 18.24141474 | 0.02 | 0.02 | 0.05 | 0.04 | 0.36 | 0.19 | 0.09 | 0.30 | 0.00 | 0.17 |
| -74.03514821 | 18.32953947 | 0.00 | 0.01 | 0.03 | 0.02 | 0.20 | 0.10 | 0.04 | 0.29 | 0.00 | 0.34 |
| -71.86302186 | 18.24006727 | 0.01 | 0.02 | 0.04 | 0.03 | 0.33 | 0.15 | 0.06 | 0.30 | 0.00 | 0.66 |
| -71.85978792 | 18.24249272 | 0.02 | 0.03 | 0.05 | 0.05 | 0.35 | 0.22 | 0.12 | 0.30 | 0.00 | 0.68 |
| -71.85870994 | 18.25003856 | 0.02 | 0.03 | 0.06 | 0.05 | 0.35 | 0.21 | 0.10 | 0.30 | 0.00 | 0.62 |
| -73.48376229 | 18.34732611 | 0.01 | 0.01 | 0.03 | 0.01 | 0.19 | 0.07 | 0.03 | 0.29 | 0.00 | 0.42 |
| -74.03460922 | 18.32711402 | 0.01 | 0.01 | 0.04 | 0.02 | 0.38 | 0.15 | 0.06 | 0.29 | 0.00 | 0.31 |
| -72.01339984 | 18.34058875 | 0.01 | 0.03 | 0.05 | 0.05 | 0.20 | 0.16 | 0.09 | 0.30 | 0.00 | 0.51 |
| -72.1098789 | 18.28507286 | 0.02 | 0.03 | 0.05 | 0.03 | 0.34 | 0.17 | 0.08 | 0.30 | 0.00 | 0.04 |
| -74.03622619 | 18.39125373 | 0.00 | 0.01 | 0.03 | 0.02 | 0.21 | 0.10 | 0.05 | 0.29 | 0.00 | 0.23 |
| -72.23115146 | 19.61044723 | 0.01 | 0.01 | 0.03 | 0.02 | 0.28 | 0.10 | 0.04 | 0.30 | 0.00 | 0.13 |
| -72.23169045 | 19.61071673 | 0.01 | 0.01 | 0.04 | 0.02 | 0.36 | 0.14 | 0.05 | 0.30 | 0.00 | 0.19 |
| -72.4931002 | 18.5071364 | 0.01 | 0.01 | 0.03 | 0.02 | 0.22 | 0.10 | 0.04 | 0.30 | 0.00 | 0.32 |
| -72.62326608 | 19.83547521 | 0.01 | 0.01 | 0.03 | 0.02 | 0.26 | 0.11 | 0.05 | 0.30 | 0.00 | 0.09 |
| -72.62542204 | 19.83763117 | 0.01 | 0.02 | 0.05 | 0.03 | 0.34 | 0.16 | 0.07 | 0.30 | 0.00 | 0.21 |
| -72.48474587 | 19.7066568 | 0.01 | 0.02 | 0.03 | 0.02 | 0.33 | 0.13 | 0.05 | 0.30 | 0.00 | 0.39 |
| -71.85978792 | 18.24572665 | 0.02 | 0.02 | 0.04 | 0.03 | 0.37 | 0.18 | 0.08 | 0.30 | 0.00 | 0.49 |
| -73.42797691 | 19.78750517 | 0.05 | 0.07 | 0.11 | 0.12 | 0.28 | 0.29 | 0.18 | 0.30 | 1.00 | 0.26 |
| -73.36114226 | 19.6271559 | 0.06 | 0.08 | 0.11 | 0.13 | 0.25 | 0.24 | 0.17 | 0.30 | 1.00 | 0.59 |
| -72.19989009 | 18.2236281 | 0.02 | 0.04 | 0.06 | 0.05 | 0.28 | 0.19 | 0.10 | 0.30 | 1.00 | 0.11 |
| -73.13045489 | 19.92440842 | 0.05 | 0.07 | 0.10 | 0.12 | 0.28 | 0.30 | 0.19 | 0.30 | 1.00 | 0.09 |
| -73.40264442 | 19.83870914 | 0.04 | 0.05 | 0.08 | 0.09 | 0.26 | 0.25 | 0.15 | 0.30 | 1.00 | 0.30 |
| -72.1993511 | 18.22416708 | 0.02 | 0.03 | 0.06 | 0.05 | 0.28 | 0.19 | 0.10 | 0.30 | 1.00 | 0.23 |
| -73.26035128 | 19.69426005 | 0.03 | 0.04 | 0.06 | 0.06 | 0.26 | 0.21 | 0.12 | 0.30 | 1.00 | 0.19 |
| -73.4039919 | 19.83817016 | 0.02 | 0.03 | 0.07 | 0.07 | 0.24 | 0.24 | 0.14 | 0.30 | 1.00 | 0.43 |
| -73.35359641 | 19.81903604 | 0.02 | 0.03 | 0.05 | 0.04 | 0.24 | 0.19 | 0.10 | 0.30 | 1.00 | 0.30 |
| -73.35548287 | 19.75839976 | 0.04 | 0.05 | 0.07 | 0.07 | 0.28 | 0.28 | 0.16 | 0.30 | 1.00 | 0.41 |
| -72.19962059 | 18.22389759 | 0.02 | 0.03 | 0.06 | 0.05 | 0.27 | 0.19 | 0.09 | 0.30 | 1.00 | 0.53 |
| -73.0056789 | 19.9157846 | 0.05 | 0.07 | 0.10 | 0.12 | 0.25 | 0.29 | 0.20 | 0.30 | 1.00 | 0.18 |
| -73.05203197 | 19.89206907 | 0.02 | 0.05 | 0.09 | 0.09 | 0.22 | 0.23 | 0.15 | 0.30 | 1.00 | 0.18 |
| -73.01645868 | 19.91821005 | 0.04 | 0.06 | 0.09 | 0.11 | 0.26 | 0.30 | 0.18 | 0.30 | 1.00 | 0.35 |
| -72.88737078 | 18.84184867 | 0.09 | 0.11 | 0.16 | 0.20 | 0.38 | 0.38 | 0.27 | 0.30 | 1.00 | 0.51 |
| -73.29322962 | 19.86026871 | 0.04 | 0.05 | 0.07 | 0.07 | 0.24 | 0.21 | 0.12 | 0.30 | 1.00 | 0.61 |
| -73.26277673 | 19.69560752 | 0.04 | 0.04 | 0.06 | 0.06 | 0.27 | 0.22 | 0.12 | 0.30 | 1.00 | 0.42 |
| -73.32556897 | 19.62850337 | 0.05 | 0.06 | 0.07 | 0.08 | 0.21 | 0.15 | 0.09 | 0.30 | 1.00 | 0.69 |
| -73.43336681 | 19.76190319 | 0.04 | 0.05 | 0.07 | 0.07 | 0.26 | 0.22 | 0.13 | 0.30 | 1.00 | 0.36 |
| -72.78577132 | 20.07532539 | 0.02 | 0.03 | 0.06 | 0.05 | 0.34 | 0.21 | 0.10 | 0.30 | 1.00 | 0.61 |
| -72.67797348 | 20.04325554 | 0.02 | 0.03 | 0.06 | 0.06 | 0.27 | 0.19 | 0.11 | 0.30 | 1.00 | 0.41 |
| -73.45061446 | 19.7613642 | 0.03 | 0.05 | 0.07 | 0.08 | 0.23 | 0.16 | 0.10 | 0.30 | 1.00 | 0.02 |
| -73.36518468 | 19.63038983 | 0.03 | 0.05 | 0.07 | 0.08 | 0.25 | 0.18 | 0.12 | 0.30 | 1.00 | 0.62 |
| -73.36168125 | 19.6320068 | 0.04 | 0.05 | 0.08 | 0.08 | 0.27 | 0.19 | 0.12 | 0.30 | 1.00 | 0.52 |
| -73.40749533 | 19.78831366 | 0.03 | 0.04 | 0.07 | 0.07 | 0.25 | 0.23 | 0.12 | 0.30 | 1.00 | 0.45 |
| -73.40291392 | 19.83790066 | 0.01 | 0.02 | 0.06 | 0.06 | 0.23 | 0.20 | 0.11 | 0.30 | 1.00 | 0.69 |
| -72.85071951 | 18.14816961 | 0.03 | 0.04 | 0.08 | 0.08 | 0.34 | 0.21 | 0.11 | 0.30 | 1.00 | 0.59 |
| -73.38027637 | 19.83924813 | 0.02 | 0.04 | 0.07 | 0.08 | 0.23 | 0.23 | 0.14 | 0.30 | 1.00 | 0.63 |
| -73.34093016 | 19.62877286 | 0.06 | 0.06 | 0.09 | 0.09 | 0.22 | 0.18 | 0.12 | 0.30 | 1.00 | 0.25 |
| -73.42770742 | 19.78777467 | 0.04 | 0.06 | 0.09 | 0.10 | 0.26 | 0.28 | 0.17 | 0.30 | 1.00 | 0.67 |
| -72.78577132 | 20.07559489 | 0.03 | 0.03 | 0.06 | 0.05 | 0.33 | 0.23 | 0.11 | 0.30 | 1.00 | 0.44 |
| -73.35494388 | 19.81795806 | 0.02 | 0.02 | 0.05 | 0.05 | 0.24 | 0.19 | 0.11 | 0.30 | 1.00 | 0.12 |
| -73.18543179 | 19.90662178 | 0.07 | 0.08 | 0.11 | 0.13 | 0.28 | 0.33 | 0.21 | 0.30 | 1.00 | 0.22 |
| -73.35332691 | 19.81903604 | 0.02 | 0.02 | 0.04 | 0.04 | 0.21 | 0.17 | 0.09 | 0.30 | 1.00 | 0.25 |
| -72.82188359 | 18.14520517 | 0.03 | 0.03 | 0.06 | 0.05 | 0.33 | 0.21 | 0.11 | 0.30 | 1.00 | 0.43 |
| -73.32583847 | 19.62931185 | 0.05 | 0.05 | 0.07 | 0.07 | 0.19 | 0.15 | 0.10 | 0.30 | 1.00 | 0.09 |
| -73.29269063 | 19.86053821 | 0.03 | 0.04 | 0.07 | 0.07 | 0.24 | 0.19 | 0.11 | 0.30 | 1.00 | 0.10 |
| -72.92429153 | 18.16433929 | 0.02 | 0.02 | 0.05 | 0.03 | 0.37 | 0.17 | 0.07 | 0.30 | 1.00 | 0.38 |
| -72.88521482 | 18.15194254 | 0.03 | 0.04 | 0.07 | 0.06 | 0.30 | 0.25 | 0.13 | 0.30 | 1.00 | 0.29 |
| -72.88790976 | 18.84184867 | 0.08 | 0.10 | 0.15 | 0.16 | 0.37 | 0.34 | 0.24 | 0.30 | 1.00 | 0.05 |
| -73.45438738 | 19.74196059 | 0.03 | 0.04 | 0.06 | 0.06 | 0.20 | 0.11 | 0.07 | 0.30 | 1.00 | 0.33 |
| -72.81487673 | 20.08853063 | 0.03 | 0.05 | 0.09 | 0.09 | 0.33 | 0.26 | 0.15 | 0.30 | 1.00 | 0.06 |
| -73.40210543 | 19.83870914 | 0.05 | 0.07 | 0.10 | 0.10 | 0.27 | 0.26 | 0.16 | 0.30 | 1.00 | 0.32 |
| -73.40237493 | 19.83870914 | 0.03 | 0.05 | 0.08 | 0.09 | 0.25 | 0.24 | 0.14 | 0.30 | 1.00 | 0.32 |
| -73.45438738 | 19.74196059 | 0.03 | 0.04 | 0.06 | 0.06 | 0.20 | 0.11 | 0.07 | 0.30 | 1.00 | 0.02 |
| -73.40264442 | 19.7910086 | 0.03 | 0.04 | 0.07 | 0.08 | 0.26 | 0.25 | 0.14 | 0.30 | 1.00 | 0.29 |
| -73.34119966 | 19.62931185 | 0.06 | 0.07 | 0.09 | 0.09 | 0.23 | 0.18 | 0.12 | 0.30 | 1.00 | 0.04 |
| -73.45061446 | 19.7613642 | 0.03 | 0.05 | 0.07 | 0.08 | 0.23 | 0.16 | 0.10 | 0.30 | 1.00 | 0.40 |
| -73.43390579 | 19.76163369 | 0.05 | 0.05 | 0.08 | 0.08 | 0.27 | 0.25 | 0.15 | 0.30 | 1.00 | 0.62 |
| -73.45007547 | 19.7613642 | 0.02 | 0.03 | 0.06 | 0.06 | 0.24 | 0.14 | 0.08 | 0.30 | 1.00 | 0.25 |
| -73.25981229 | 19.69533803 | 0.03 | 0.04 | 0.06 | 0.05 | 0.25 | 0.21 | 0.12 | 0.30 | 1.00 | 0.61 |
| -73.45007547 | 19.7613642 | 0.02 | 0.03 | 0.06 | 0.06 | 0.24 | 0.14 | 0.08 | 0.30 | 1.00 | 0.21 |
| -72.19773413 | 18.22551456 | 0.02 | 0.02 | 0.05 | 0.03 | 0.27 | 0.15 | 0.07 | 0.30 | 1.00 | 0.01 |
| -72.92483052 | 18.16406979 | 0.02 | 0.02 | 0.05 | 0.03 | 0.36 | 0.16 | 0.07 | 0.30 | 1.00 | 0.60 |
| -72.78792727 | 18.15086456 | 0.02 | 0.03 | 0.05 | 0.04 | 0.31 | 0.18 | 0.08 | 0.30 | 1.00 | 0.35 |
| -72.78738828 | 18.15086456 | 0.02 | 0.02 | 0.05 | 0.03 | 0.32 | 0.16 | 0.07 | 0.30 | 1.00 | 0.63 |
| -73.45411789 | 19.7411521 | 0.04 | 0.05 | 0.07 | 0.07 | 0.21 | 0.15 | 0.09 | 0.30 | 1.00 | 0.51 |
| -73.25981229 | 19.69479904 | 0.04 | 0.04 | 0.07 | 0.07 | 0.27 | 0.24 | 0.14 | 0.30 | 1.00 | 0.06 |
| -72.20042908 | 18.22416708 | 0.03 | 0.04 | 0.07 | 0.06 | 0.34 | 0.24 | 0.13 | 0.30 | 1.00 | 0.21 |
| -73.43390579 | 19.76163369 | 0.05 | 0.05 | 0.08 | 0.08 | 0.27 | 0.25 | 0.15 | 0.30 | 1.00 | 0.14 |
| -72.78550182 | 20.07532539 | 0.03 | 0.04 | 0.07 | 0.06 | 0.36 | 0.25 | 0.12 | 0.30 | 1.00 | 0.45 |
| -72.19962059 | 18.22389759 | 0.02 | 0.03 | 0.06 | 0.05 | 0.27 | 0.19 | 0.09 | 0.30 | 1.00 | 0.61 |
| -73.35467439 | 19.81822756 | 0.02 | 0.03 | 0.05 | 0.05 | 0.24 | 0.20 | 0.11 | 0.30 | 1.00 | 0.07 |
| -72.92456103 | 18.1638003 | 0.02 | 0.02 | 0.05 | 0.03 | 0.35 | 0.16 | 0.07 | 0.30 | 1.00 | 0.29 |
| -73.4085733 | 19.78858315 | 0.03 | 0.04 | 0.07 | 0.07 | 0.26 | 0.25 | 0.13 | 0.30 | 1.00 | 0.23 |
| -72.81460724 | 20.08826113 | 0.02 | 0.04 | 0.08 | 0.08 | 0.34 | 0.25 | 0.13 | 0.30 | 1.00 | 0.56 |
| -72.81514623 | 20.08826113 | 0.04 | 0.06 | 0.09 | 0.10 | 0.36 | 0.29 | 0.17 | 0.30 | 1.00 | 0.27 |
| -73.4088428 | 19.78831366 | 0.03 | 0.04 | 0.07 | 0.07 | 0.27 | 0.24 | 0.13 | 0.30 | 1.00 | 0.67 |
| -72.20042908 | 18.22416708 | 0.03 | 0.04 | 0.07 | 0.06 | 0.34 | 0.24 | 0.13 | 0.30 | 1.00 | 0.67 |
| -72.78550182 | 20.07586438 | 0.03 | 0.04 | 0.06 | 0.05 | 0.34 | 0.21 | 0.10 | 0.30 | 1.00 | 0.63 |
| -73.36141175 | 19.62742539 | 0.04 | 0.05 | 0.08 | 0.08 | 0.24 | 0.18 | 0.11 | 0.30 | 1.00 | 0.09 |
| -72.78577132 | 20.07559489 | 0.03 | 0.03 | 0.06 | 0.05 | 0.33 | 0.23 | 0.11 | 0.30 | 1.00 | 0.20 |
| -72.88764027 | 18.84184867 | 0.07 | 0.09 | 0.15 | 0.16 | 0.38 | 0.34 | 0.23 | 0.30 | 1.00 | 0.19 |
| -73.26277673 | 19.69533803 | 0.03 | 0.04 | 0.07 | 0.07 | 0.27 | 0.27 | 0.15 | 0.30 | 1.00 | 0.66 |
| -73.35332691 | 19.81903604 | 0.02 | 0.02 | 0.04 | 0.04 | 0.21 | 0.17 | 0.09 | 0.30 | 1.00 | 0.60 |
| -73.26277673 | 19.69533803 | 0.03 | 0.04 | 0.07 | 0.07 | 0.27 | 0.27 | 0.15 | 0.30 | 1.00 | 0.42 |
| -72.1993511 | 18.22443658 | 0.02 | 0.02 | 0.05 | 0.03 | 0.25 | 0.15 | 0.07 | 0.30 | 1.00 | 0.58 |
| -72.78577132 | 20.0750559 | 0.02 | 0.03 | 0.06 | 0.05 | 0.37 | 0.22 | 0.10 | 0.30 | 1.00 | 0.50 |
| -73.34066067 | 19.62904236 | 0.06 | 0.06 | 0.08 | 0.09 | 0.18 | 0.16 | 0.11 | 0.30 | 1.00 | 0.31 |
| -72.88764027 | 18.84184867 | 0.07 | 0.09 | 0.15 | 0.16 | 0.38 | 0.34 | 0.23 | 0.30 | 1.00 | 0.57 |
| -73.35467439 | 19.81795806 | 0.01 | 0.02 | 0.04 | 0.04 | 0.23 | 0.17 | 0.09 | 0.30 | 1.00 | 0.64 |
| -73.45438738 | 19.7414216 | 0.03 | 0.04 | 0.06 | 0.06 | 0.22 | 0.13 | 0.08 | 0.30 | 1.00 | 0.46 |
| -73.05257096 | 19.89206907 | 0.03 | 0.05 | 0.10 | 0.12 | 0.24 | 0.28 | 0.19 | 0.30 | 1.00 | 0.20 |
| -73.1051224 | 19.91605409 | 0.04 | 0.05 | 0.08 | 0.10 | 0.26 | 0.28 | 0.17 | 0.30 | 1.00 | 0.64 |
| -73.12964641 | 19.92467792 | 0.07 | 0.10 | 0.15 | 0.18 | 0.31 | 0.35 | 0.24 | 0.30 | 1.00 | 0.62 |
| -73.05203197 | 19.89233857 | 0.03 | 0.04 | 0.08 | 0.09 | 0.22 | 0.22 | 0.14 | 0.30 | 1.00 | 0.24 |
| -73.3191011 | 19.62985084 | 0.06 | 0.07 | 0.08 | 0.09 | 0.22 | 0.20 | 0.13 | 0.30 | 1.00 | 0.41 |
| -73.40237493 | 19.83843965 | 0.02 | 0.03 | 0.08 | 0.08 | 0.25 | 0.24 | 0.14 | 0.30 | 1.00 | 0.20 |
| -73.36060327 | 19.6268864 | 0.06 | 0.07 | 0.10 | 0.11 | 0.26 | 0.22 | 0.15 | 0.30 | 1.00 | 0.23 |
| -72.92483052 | 18.16406979 | 0.02 | 0.02 | 0.05 | 0.03 | 0.36 | 0.16 | 0.07 | 0.30 | 1.00 | 0.55 |
| -73.40830381 | 19.78831366 | 0.03 | 0.04 | 0.07 | 0.07 | 0.27 | 0.24 | 0.14 | 0.30 | 1.00 | 0.09 |
| -73.34066067 | 19.62904236 | 0.06 | 0.06 | 0.08 | 0.09 | 0.18 | 0.16 | 0.11 | 0.30 | 1.00 | 0.09 |
| -73.37704244 | 19.82631239 | 0.02 | 0.03 | 0.06 | 0.06 | 0.24 | 0.24 | 0.14 | 0.30 | 1.00 | 0.60 |
| -72.19800363 | 18.22524506 | 0.02 | 0.02 | 0.04 | 0.02 | 0.27 | 0.14 | 0.06 | 0.30 | 1.00 | 0.27 |
| -73.42743792 | 19.78804416 | 0.05 | 0.07 | 0.10 | 0.12 | 0.27 | 0.29 | 0.18 | 0.30 | 1.00 | 0.60 |
| -72.88521482 | 18.15167304 | 0.03 | 0.03 | 0.06 | 0.05 | 0.30 | 0.21 | 0.11 | 0.30 | 1.00 | 0.28 |
| -73.32556897 | 19.62877286 | 0.05 | 0.05 | 0.07 | 0.07 | 0.20 | 0.14 | 0.09 | 0.30 | 1.00 | 0.36 |
| -72.92456103 | 18.16460878 | 0.02 | 0.02 | 0.05 | 0.03 | 0.38 | 0.16 | 0.06 | 0.30 | 1.00 | 0.50 |
| -72.7868493 | 20.0747864 | 0.03 | 0.03 | 0.06 | 0.06 | 0.37 | 0.23 | 0.11 | 0.30 | 1.00 | 0.08 |
| -73.35332691 | 19.81930553 | 0.02 | 0.03 | 0.06 | 0.06 | 0.26 | 0.22 | 0.13 | 0.30 | 1.00 | 0.13 |
| -72.19854262 | 18.22551456 | 0.03 | 0.03 | 0.07 | 0.05 | 0.34 | 0.22 | 0.11 | 0.30 | 1.00 | 0.44 |
| -73.43336681 | 19.76217268 | 0.04 | 0.05 | 0.07 | 0.07 | 0.27 | 0.25 | 0.14 | 0.30 | 1.00 | 0.47 |
| -73.18597078 | 19.90635229 | 0.06 | 0.08 | 0.11 | 0.13 | 0.29 | 0.33 | 0.21 | 0.30 | 1.00 | 0.61 |
| -72.78496283 | 20.0750559 | 0.03 | 0.03 | 0.06 | 0.05 | 0.35 | 0.21 | 0.10 | 0.30 | 1.00 | 0.24 |
| -73.42824641 | 19.78831366 | 0.05 | 0.07 | 0.11 | 0.13 | 0.28 | 0.30 | 0.19 | 0.30 | 1.00 | 0.18 |
| -72.88710128 | 18.84130968 | 0.07 | 0.09 | 0.14 | 0.16 | 0.35 | 0.34 | 0.24 | 0.30 | 1.00 | 0.12 |
| -73.36491518 | 19.63038983 | 0.03 | 0.04 | 0.07 | 0.07 | 0.24 | 0.18 | 0.11 | 0.30 | 1.00 | 0.54 |
| -73.40210543 | 19.7912781 | 0.03 | 0.04 | 0.07 | 0.08 | 0.25 | 0.24 | 0.14 | 0.30 | 1.00 | 0.02 |
| -72.67797348 | 20.04298604 | 0.02 | 0.03 | 0.06 | 0.05 | 0.31 | 0.19 | 0.10 | 0.30 | 1.00 | 0.07 |
| -73.32583847 | 19.62931185 | 0.05 | 0.05 | 0.07 | 0.07 | 0.19 | 0.15 | 0.10 | 0.30 | 1.00 | 0.06 |
| -73.2595428 | 19.69533803 | 0.04 | 0.04 | 0.07 | 0.07 | 0.26 | 0.23 | 0.13 | 0.30 | 1.00 | 0.12 |
| -73.43282782 | 19.76190319 | 0.04 | 0.05 | 0.07 | 0.07 | 0.28 | 0.25 | 0.13 | 0.30 | 1.00 | 0.25 |
| -73.32556897 | 19.62904236 | 0.05 | 0.05 | 0.07 | 0.07 | 0.19 | 0.14 | 0.09 | 0.30 | 1.00 | 0.45 |
| -73.40776482 | 19.78804416 | 0.03 | 0.04 | 0.07 | 0.07 | 0.24 | 0.24 | 0.14 | 0.30 | 1.00 | 0.07 |
| -73.42716843 | 19.78804416 | 0.05 | 0.07 | 0.10 | 0.11 | 0.26 | 0.29 | 0.19 | 0.30 | 1.00 | 0.07 |
| -72.78577132 | 20.07532539 | 0.02 | 0.03 | 0.06 | 0.05 | 0.34 | 0.21 | 0.10 | 0.30 | 1.00 | 0.52 |
| -73.37731193 | 19.82658189 | 0.02 | 0.03 | 0.07 | 0.07 | 0.25 | 0.24 | 0.14 | 0.30 | 1.00 | 0.60 |
| -73.3541354 | 19.81876655 | 0.01 | 0.03 | 0.05 | 0.05 | 0.24 | 0.20 | 0.11 | 0.30 | 1.00 | 0.17 |
| -73.36114226 | 19.62742539 | 0.05 | 0.07 | 0.10 | 0.12 | 0.25 | 0.22 | 0.15 | 0.30 | 1.00 | 0.68 |
| -72.82188359 | 18.14493568 | 0.03 | 0.03 | 0.06 | 0.05 | 0.35 | 0.21 | 0.10 | 0.30 | 1.00 | 0.58 |
| -72.19962059 | 18.22443658 | 0.02 | 0.02 | 0.04 | 0.02 | 0.26 | 0.14 | 0.06 | 0.30 | 1.00 | 0.61 |
| -73.40830381 | 19.78858315 | 0.03 | 0.04 | 0.07 | 0.07 | 0.27 | 0.24 | 0.14 | 0.30 | 1.00 | 0.44 |
| -73.18624027 | 19.90608279 | 0.06 | 0.07 | 0.11 | 0.12 | 0.28 | 0.32 | 0.20 | 0.30 | 1.00 | 0.38 |
| -72.19827312 | 18.22524506 | 0.02 | 0.02 | 0.04 | 0.03 | 0.27 | 0.15 | 0.06 | 0.30 | 1.00 | 0.67 |
| -73.37677294 | 19.82631239 | 0.01 | 0.03 | 0.06 | 0.06 | 0.23 | 0.23 | 0.13 | 0.30 | 1.00 | 0.43 |
| -73.29269063 | 19.86053821 | 0.03 | 0.04 | 0.07 | 0.07 | 0.24 | 0.19 | 0.11 | 0.30 | 1.00 | 0.31 |
| -73.18570128 | 19.90635229 | 0.06 | 0.07 | 0.10 | 0.11 | 0.27 | 0.31 | 0.19 | 0.30 | 1.00 | 0.30 |
| -73.18543179 | 19.90608279 | 0.06 | 0.07 | 0.10 | 0.11 | 0.27 | 0.31 | 0.20 | 0.30 | 1.00 | 0.12 |
| -73.38081536 | 19.83897864 | 0.02 | 0.04 | 0.07 | 0.07 | 0.26 | 0.23 | 0.13 | 0.30 | 1.00 | 0.05 |
| -73.40237493 | 19.7910086 | 0.03 | 0.04 | 0.07 | 0.07 | 0.25 | 0.23 | 0.13 | 0.30 | 1.00 | 0.03 |
| -73.32556897 | 19.62904236 | 0.05 | 0.05 | 0.07 | 0.07 | 0.19 | 0.14 | 0.09 | 0.30 | 1.00 | 0.40 |
| -72.8213446 | 18.14466618 | 0.03 | 0.04 | 0.06 | 0.05 | 0.38 | 0.21 | 0.10 | 0.30 | 1.00 | 0.00 |
| -72.85071951 | 18.14816961 | 0.03 | 0.04 | 0.08 | 0.08 | 0.34 | 0.21 | 0.11 | 0.30 | 1.00 | 0.70 |
| -73.43309731 | 19.76163369 | 0.04 | 0.05 | 0.07 | 0.07 | 0.28 | 0.25 | 0.14 | 0.30 | 1.00 | 0.44 |
| -72.88764027 | 18.84130968 | 0.03 | 0.05 | 0.09 | 0.09 | 0.34 | 0.24 | 0.14 | 0.30 | 1.00 | 0.21 |
| -73.13045489 | 19.92440842 | 0.05 | 0.07 | 0.10 | 0.12 | 0.28 | 0.30 | 0.19 | 0.30 | 1.00 | 0.65 |
| -72.78550182 | 20.07586438 | 0.03 | 0.04 | 0.06 | 0.05 | 0.34 | 0.21 | 0.10 | 0.30 | 1.00 | 0.13 |
| -72.19746464 | 18.22605355 | 0.02 | 0.03 | 0.05 | 0.04 | 0.29 | 0.18 | 0.09 | 0.30 | 1.00 | 0.66 |
| -73.42770742 | 19.78777467 | 0.04 | 0.06 | 0.09 | 0.10 | 0.26 | 0.28 | 0.17 | 0.30 | 1.00 | 0.63 |
| -73.42770742 | 19.78831366 | 0.04 | 0.06 | 0.09 | 0.10 | 0.27 | 0.28 | 0.18 | 0.30 | 1.00 | 0.16 |
| -73.36060327 | 19.6271559 | 0.05 | 0.06 | 0.09 | 0.10 | 0.26 | 0.20 | 0.13 | 0.30 | 1.00 | 0.66 |
| -73.35359641 | 19.81876655 | 0.01 | 0.01 | 0.03 | 0.02 | 0.20 | 0.13 | 0.07 | 0.30 | 1.00 | 0.61 |
| -72.19989009 | 18.22416708 | 0.02 | 0.03 | 0.05 | 0.04 | 0.25 | 0.16 | 0.08 | 0.30 | 1.00 | 0.31 |
| -73.40210543 | 19.7912781 | 0.03 | 0.04 | 0.07 | 0.08 | 0.25 | 0.24 | 0.14 | 0.30 | 1.00 | 0.05 |
| -73.31856211 | 19.62904236 | 0.05 | 0.06 | 0.08 | 0.08 | 0.23 | 0.17 | 0.11 | 0.30 | 1.00 | 0.46 |
| -73.26304623 | 19.69506853 | 0.04 | 0.05 | 0.08 | 0.08 | 0.29 | 0.30 | 0.17 | 0.30 | 1.00 | 0.49 |
| -73.40318341 | 19.83763117 | 0.01 | 0.03 | 0.06 | 0.06 | 0.23 | 0.20 | 0.11 | 0.30 | 1.00 | 0.67 |
| -72.92456103 | 18.16460878 | 0.02 | 0.02 | 0.05 | 0.03 | 0.38 | 0.16 | 0.06 | 0.30 | 1.00 | 0.48 |
| -73.4088428 | 19.78804416 | 0.03 | 0.04 | 0.07 | 0.06 | 0.27 | 0.23 | 0.12 | 0.30 | 1.00 | 0.45 |
| -72.19827312 | 18.22524506 | 0.02 | 0.02 | 0.04 | 0.03 | 0.27 | 0.15 | 0.06 | 0.30 | 1.00 | 0.59 |
| -73.05230146 | 19.89179958 | 0.02 | 0.04 | 0.08 | 0.08 | 0.22 | 0.21 | 0.14 | 0.30 | 1.00 | 0.13 |
| -73.35359641 | 19.81903604 | 0.02 | 0.03 | 0.05 | 0.04 | 0.24 | 0.19 | 0.10 | 0.30 | 1.00 | 0.41 |
| -73.18543179 | 19.90608279 | 0.06 | 0.07 | 0.10 | 0.11 | 0.27 | 0.31 | 0.20 | 0.30 | 1.00 | 0.07 |
| -73.42743792 | 19.78831366 | 0.04 | 0.06 | 0.09 | 0.11 | 0.26 | 0.29 | 0.18 | 0.30 | 1.00 | 0.34 |
| -73.31829262 | 19.62904236 | 0.05 | 0.06 | 0.08 | 0.08 | 0.25 | 0.18 | 0.11 | 0.30 | 1.00 | 0.40 |
| -73.1299159 | 19.92440842 | 0.07 | 0.10 | 0.14 | 0.17 | 0.29 | 0.34 | 0.23 | 0.30 | 1.00 | 0.26 |
| -72.78738828 | 20.0750559 | 0.02 | 0.03 | 0.05 | 0.05 | 0.33 | 0.20 | 0.09 | 0.30 | 1.00 | 0.26 |
| -73.40237493 | 19.83843965 | 0.02 | 0.03 | 0.08 | 0.08 | 0.25 | 0.24 | 0.14 | 0.30 | 1.00 | 0.50 |
| -73.40830381 | 19.78804416 | 0.03 | 0.04 | 0.07 | 0.07 | 0.26 | 0.23 | 0.13 | 0.30 | 1.00 | 0.45 |
| -72.81568522 | 20.08853063 | 0.03 | 0.05 | 0.09 | 0.09 | 0.33 | 0.26 | 0.15 | 0.30 | 1.00 | 0.20 |
| -73.05230146 | 19.89233857 | 0.02 | 0.04 | 0.07 | 0.08 | 0.21 | 0.20 | 0.13 | 0.30 | 1.00 | 0.31 |
| -73.45438738 | 19.74088261 | 0.03 | 0.04 | 0.05 | 0.06 | 0.17 | 0.11 | 0.07 | 0.30 | 1.00 | 0.01 |
| -72.78711879 | 20.07532539 | 0.02 | 0.03 | 0.06 | 0.05 | 0.31 | 0.21 | 0.10 | 0.30 | 1.00 | 0.45 |
| -73.26331572 | 19.69533803 | 0.03 | 0.04 | 0.06 | 0.06 | 0.27 | 0.21 | 0.12 | 0.30 | 1.00 | 0.53 |
| -73.40911229 | 19.78831366 | 0.03 | 0.04 | 0.07 | 0.06 | 0.27 | 0.24 | 0.13 | 0.30 | 1.00 | 0.10 |
| -72.1993511 | 18.22443658 | 0.02 | 0.02 | 0.05 | 0.03 | 0.25 | 0.15 | 0.07 | 0.30 | 1.00 | 0.02 |
| -73.32583847 | 19.62931185 | 0.05 | 0.05 | 0.07 | 0.07 | 0.19 | 0.15 | 0.10 | 0.30 | 1.00 | 0.21 |
| -73.36491518 | 19.62985084 | 0.03 | 0.04 | 0.07 | 0.07 | 0.23 | 0.18 | 0.11 | 0.30 | 1.00 | 0.26 |
| -73.36518468 | 19.63012034 | 0.03 | 0.04 | 0.07 | 0.07 | 0.24 | 0.18 | 0.11 | 0.30 | 1.00 | 0.09 |
| -73.18597078 | 19.90635229 | 0.06 | 0.08 | 0.11 | 0.13 | 0.29 | 0.33 | 0.21 | 0.30 | 1.00 | 0.30 |
| -72.19719514 | 18.22551456 | 0.02 | 0.03 | 0.05 | 0.04 | 0.26 | 0.16 | 0.08 | 0.30 | 1.00 | 0.23 |
| -73.43336681 | 19.76217268 | 0.04 | 0.05 | 0.07 | 0.07 | 0.27 | 0.25 | 0.14 | 0.30 | 1.00 | 0.30 |
| -73.37758143 | 19.82685138 | 0.02 | 0.03 | 0.06 | 0.07 | 0.24 | 0.24 | 0.14 | 0.30 | 1.00 | 0.18 |
| -73.4088428 | 19.78777467 | 0.02 | 0.03 | 0.06 | 0.05 | 0.23 | 0.21 | 0.11 | 0.30 | 1.00 | 0.05 |
| -73.36060327 | 19.62796438 | 0.05 | 0.06 | 0.09 | 0.10 | 0.25 | 0.20 | 0.13 | 0.30 | 1.00 | 0.03 |
| -73.18543179 | 19.90635229 | 0.06 | 0.07 | 0.10 | 0.11 | 0.27 | 0.32 | 0.20 | 0.30 | 1.00 | 0.18 |
| -73.26062078 | 19.69479904 | 0.04 | 0.04 | 0.07 | 0.07 | 0.29 | 0.24 | 0.14 | 0.30 | 1.00 | 0.32 |
| -73.10485291 | 19.91605409 | 0.04 | 0.05 | 0.08 | 0.09 | 0.26 | 0.28 | 0.17 | 0.30 | 1.00 | 0.18 |
| -73.43390579 | 19.76163369 | 0.05 | 0.05 | 0.08 | 0.08 | 0.27 | 0.25 | 0.15 | 0.30 | 1.00 | 0.36 |
| -73.05230146 | 19.89179958 | 0.02 | 0.04 | 0.08 | 0.08 | 0.22 | 0.21 | 0.14 | 0.30 | 1.00 | 0.15 |
| -73.36087276 | 19.6268864 | 0.06 | 0.08 | 0.11 | 0.14 | 0.25 | 0.25 | 0.18 | 0.30 | 1.00 | 0.19 |
| -73.4085733 | 19.78804416 | 0.03 | 0.04 | 0.07 | 0.07 | 0.27 | 0.23 | 0.13 | 0.30 | 1.00 | 0.40 |
| -73.43390579 | 19.76163369 | 0.05 | 0.05 | 0.08 | 0.08 | 0.27 | 0.25 | 0.15 | 0.30 | 1.00 | 0.58 |
| -72.78523233 | 20.0747864 | 0.03 | 0.04 | 0.08 | 0.07 | 0.38 | 0.26 | 0.13 | 0.30 | 1.00 | 0.65 |
| -73.26062078 | 19.69452954 | 0.03 | 0.04 | 0.06 | 0.06 | 0.26 | 0.21 | 0.12 | 0.30 | 1.00 | 0.28 |
| -73.4336363 | 19.76190319 | 0.05 | 0.05 | 0.07 | 0.07 | 0.27 | 0.24 | 0.14 | 0.30 | 1.00 | 0.41 |
| -72.92429153 | 18.16433929 | 0.02 | 0.02 | 0.05 | 0.03 | 0.37 | 0.17 | 0.07 | 0.30 | 1.00 | 0.29 |
| -72.8216141 | 18.14466618 | 0.02 | 0.03 | 0.06 | 0.05 | 0.38 | 0.21 | 0.11 | 0.30 | 1.00 | 0.04 |
| -73.40803431 | 19.78858315 | 0.03 | 0.03 | 0.06 | 0.06 | 0.26 | 0.23 | 0.12 | 0.30 | 1.00 | 0.65 |
| -73.3191011 | 19.62985084 | 0.06 | 0.07 | 0.08 | 0.09 | 0.22 | 0.20 | 0.13 | 0.30 | 1.00 | 0.40 |
| -73.37731193 | 19.82658189 | 0.02 | 0.03 | 0.07 | 0.07 | 0.25 | 0.24 | 0.14 | 0.30 | 1.00 | 0.01 |
| -73.36572366 | 19.63012034 | 0.04 | 0.05 | 0.07 | 0.08 | 0.23 | 0.19 | 0.13 | 0.30 | 1.00 | 0.17 |
| -72.88683179 | 18.84157918 | 0.09 | 0.11 | 0.16 | 0.19 | 0.35 | 0.39 | 0.30 | 0.30 | 1.00 | 0.23 |
| -73.38054587 | 19.83924813 | 0.02 | 0.04 | 0.07 | 0.08 | 0.25 | 0.24 | 0.14 | 0.30 | 1.00 | 0.36 |
| -73.12964641 | 19.92467792 | 0.07 | 0.10 | 0.15 | 0.18 | 0.31 | 0.35 | 0.24 | 0.30 | 1.00 | 0.02 |
| -73.42797691 | 19.78750517 | 0.05 | 0.07 | 0.11 | 0.12 | 0.28 | 0.29 | 0.18 | 0.30 | 1.00 | 0.40 |
| -73.3541354 | 19.81849705 | 0.01 | 0.02 | 0.04 | 0.04 | 0.21 | 0.16 | 0.08 | 0.30 | 1.00 | 0.57 |
| -73.45034496 | 19.7613642 | 0.03 | 0.04 | 0.06 | 0.06 | 0.23 | 0.14 | 0.08 | 0.30 | 1.00 | 0.10 |
| -73.45438738 | 19.7411521 | 0.04 | 0.05 | 0.07 | 0.07 | 0.22 | 0.15 | 0.09 | 0.30 | 1.00 | 0.21 |
| -73.4088428 | 19.78777467 | 0.02 | 0.03 | 0.06 | 0.05 | 0.23 | 0.21 | 0.11 | 0.30 | 1.00 | 0.49 |
| -73.38135435 | 19.83897864 | 0.01 | 0.03 | 0.06 | 0.06 | 0.25 | 0.21 | 0.11 | 0.30 | 1.00 | 0.37 |
| -73.38027637 | 19.83924813 | 0.02 | 0.04 | 0.07 | 0.08 | 0.23 | 0.23 | 0.14 | 0.30 | 1.00 | 0.19 |
| -73.3193706 | 19.62958135 | 0.06 | 0.07 | 0.09 | 0.09 | 0.23 | 0.20 | 0.13 | 0.30 | 1.00 | 0.09 |
| -73.29269063 | 19.86053821 | 0.03 | 0.04 | 0.07 | 0.07 | 0.24 | 0.19 | 0.11 | 0.30 | 1.00 | 0.68 |
| -73.29269063 | 19.8608077 | 0.03 | 0.04 | 0.06 | 0.06 | 0.16 | 0.15 | 0.09 | 0.30 | 1.00 | 0.25 |
| -73.45492637 | 19.74169109 | 0.03 | 0.04 | 0.05 | 0.05 | 0.18 | 0.10 | 0.07 | 0.30 | 1.00 | 0.19 |
| -73.29322962 | 19.85999922 | 0.03 | 0.04 | 0.07 | 0.07 | 0.27 | 0.22 | 0.12 | 0.30 | 1.00 | 0.51 |
| -72.19881211 | 18.22470607 | 0.03 | 0.03 | 0.05 | 0.04 | 0.26 | 0.17 | 0.09 | 0.30 | 1.00 | 0.41 |
| -73.35521338 | 19.75786077 | 0.04 | 0.04 | 0.06 | 0.06 | 0.27 | 0.25 | 0.14 | 0.30 | 1.00 | 0.23 |
| -72.88737078 | 18.84184867 | 0.09 | 0.11 | 0.16 | 0.20 | 0.38 | 0.38 | 0.27 | 0.30 | 1.00 | 0.46 |
| -73.4285159 | 19.78723568 | 0.05 | 0.07 | 0.11 | 0.12 | 0.28 | 0.30 | 0.19 | 0.30 | 1.00 | 0.19 |
| -73.40830381 | 19.78831366 | 0.03 | 0.04 | 0.07 | 0.07 | 0.27 | 0.24 | 0.14 | 0.30 | 1.00 | 0.61 |
| -72.19854262 | 18.22524506 | 0.02 | 0.02 | 0.05 | 0.03 | 0.28 | 0.15 | 0.07 | 0.30 | 1.00 | 0.34 |
| -72.88737078 | 18.84184867 | 0.09 | 0.11 | 0.16 | 0.20 | 0.38 | 0.38 | 0.27 | 0.30 | 1.00 | 0.08 |
| -72.78738828 | 18.15113405 | 0.02 | 0.03 | 0.06 | 0.04 | 0.32 | 0.18 | 0.09 | 0.30 | 1.00 | 0.61 |
| -73.38108486 | 19.83870914 | 0.03 | 0.05 | 0.08 | 0.08 | 0.26 | 0.24 | 0.14 | 0.30 | 1.00 | 0.21 |
| -72.92456103 | 18.16406979 | 0.02 | 0.02 | 0.05 | 0.03 | 0.37 | 0.16 | 0.07 | 0.30 | 1.00 | 0.51 |
| -73.38027637 | 19.83924813 | 0.02 | 0.04 | 0.07 | 0.08 | 0.23 | 0.23 | 0.14 | 0.30 | 1.00 | 0.10 |
| -72.8512585 | 18.14816961 | 0.04 | 0.05 | 0.08 | 0.09 | 0.32 | 0.25 | 0.14 | 0.30 | 1.00 | 0.46 |
| -73.36195074 | 19.6320068 | 0.03 | 0.05 | 0.08 | 0.08 | 0.28 | 0.18 | 0.11 | 0.30 | 1.00 | 0.60 |
| -72.19962059 | 18.22416708 | 0.02 | 0.03 | 0.05 | 0.04 | 0.24 | 0.16 | 0.08 | 0.30 | 1.00 | 0.63 |
| -73.01672818 | 19.91847954 | 0.05 | 0.07 | 0.10 | 0.11 | 0.26 | 0.30 | 0.19 | 0.30 | 1.00 | 0.11 |
| -72.7865798 | 20.07451691 | 0.04 | 0.05 | 0.09 | 0.08 | 0.39 | 0.27 | 0.14 | 0.30 | 1.00 | 0.27 |
| -72.7865798 | 20.0750559 | 0.02 | 0.02 | 0.06 | 0.05 | 0.34 | 0.21 | 0.09 | 0.30 | 1.00 | 0.16 |
| -73.35440489 | 19.81795806 | 0.01 | 0.02 | 0.04 | 0.03 | 0.21 | 0.15 | 0.08 | 0.30 | 1.00 | 0.20 |
| -73.37758143 | 19.82658189 | 0.02 | 0.03 | 0.07 | 0.07 | 0.25 | 0.24 | 0.14 | 0.30 | 1.00 | 0.00 |
| -72.78550182 | 20.07532539 | 0.03 | 0.04 | 0.07 | 0.06 | 0.36 | 0.25 | 0.12 | 0.30 | 1.00 | 0.46 |
| -73.18597078 | 19.9058133 | 0.06 | 0.07 | 0.10 | 0.11 | 0.27 | 0.31 | 0.19 | 0.30 | 1.00 | 0.28 |
| -73.18650977 | 19.90608279 | 0.06 | 0.08 | 0.11 | 0.12 | 0.28 | 0.34 | 0.21 | 0.30 | 1.00 | 0.21 |
| -72.19773413 | 18.22551456 | 0.02 | 0.02 | 0.05 | 0.03 | 0.27 | 0.15 | 0.07 | 0.30 | 1.00 | 0.18 |
| -73.45061446 | 19.7610947 | 0.04 | 0.05 | 0.08 | 0.09 | 0.23 | 0.19 | 0.12 | 0.30 | 1.00 | 0.11 |
| -72.78523233 | 20.07559489 | 0.03 | 0.03 | 0.06 | 0.05 | 0.35 | 0.22 | 0.11 | 0.30 | 1.00 | 0.59 |
| -72.8216141 | 18.14439669 | 0.03 | 0.03 | 0.06 | 0.05 | 0.38 | 0.20 | 0.10 | 0.30 | 1.00 | 0.65 |
| -73.01699767 | 19.91847954 | 0.05 | 0.07 | 0.10 | 0.12 | 0.27 | 0.32 | 0.20 | 0.30 | 1.00 | 0.36 |
| -72.7865798 | 20.0747864 | 0.03 | 0.04 | 0.07 | 0.07 | 0.37 | 0.26 | 0.13 | 0.30 | 1.00 | 0.22 |
| -73.43336681 | 19.76190319 | 0.04 | 0.05 | 0.07 | 0.07 | 0.26 | 0.22 | 0.13 | 0.30 | 1.00 | 0.46 |
| -73.26250724 | 19.69560752 | 0.04 | 0.05 | 0.07 | 0.07 | 0.28 | 0.26 | 0.16 | 0.30 | 1.00 | 0.45 |
| -72.19881211 | 18.22443658 | 0.03 | 0.04 | 0.07 | 0.06 | 0.31 | 0.22 | 0.12 | 0.30 | 1.00 | 0.22 |
| -73.40264442 | 19.83817016 | 0.02 | 0.04 | 0.07 | 0.07 | 0.24 | 0.22 | 0.13 | 0.30 | 1.00 | 0.60 |
| -72.78550182 | 20.0750559 | 0.03 | 0.04 | 0.07 | 0.06 | 0.37 | 0.24 | 0.12 | 0.30 | 1.00 | 0.34 |
| -72.88790976 | 18.84157918 | 0.06 | 0.08 | 0.13 | 0.14 | 0.35 | 0.32 | 0.21 | 0.30 | 1.00 | 0.01 |
| -73.26277673 | 19.69560752 | 0.04 | 0.04 | 0.06 | 0.06 | 0.27 | 0.22 | 0.12 | 0.30 | 1.00 | 0.35 |
| -72.1990816 | 18.22470607 | 0.03 | 0.03 | 0.05 | 0.04 | 0.25 | 0.17 | 0.08 | 0.30 | 1.00 | 0.70 |
| -73.18543179 | 19.90662178 | 0.07 | 0.08 | 0.11 | 0.13 | 0.28 | 0.33 | 0.21 | 0.30 | 1.00 | 0.16 |
| -73.32556897 | 19.62877286 | 0.05 | 0.05 | 0.07 | 0.07 | 0.20 | 0.14 | 0.09 | 0.30 | 1.00 | 0.34 |
| -72.78577132 | 20.07532539 | 0.02 | 0.03 | 0.06 | 0.05 | 0.34 | 0.21 | 0.10 | 0.30 | 1.00 | 0.38 |
| -72.8860233 | 18.15194254 | 0.04 | 0.05 | 0.07 | 0.07 | 0.32 | 0.25 | 0.14 | 0.30 | 1.00 | 0.49 |
| -72.19881211 | 18.22497557 | 0.02 | 0.03 | 0.05 | 0.03 | 0.26 | 0.15 | 0.07 | 0.30 | 1.00 | 0.28 |
| -72.19854262 | 18.22470607 | 0.02 | 0.03 | 0.06 | 0.04 | 0.28 | 0.18 | 0.09 | 0.30 | 1.00 | 0.55 |
| -72.78738828 | 20.07532539 | 0.03 | 0.03 | 0.06 | 0.05 | 0.31 | 0.21 | 0.10 | 0.30 | 1.00 | 0.11 |
| -73.45492637 | 19.74249958 | 0.03 | 0.04 | 0.05 | 0.06 | 0.18 | 0.12 | 0.07 | 0.30 | 1.00 | 0.32 |
| -73.05230146 | 19.89260806 | 0.03 | 0.04 | 0.07 | 0.08 | 0.22 | 0.21 | 0.13 | 0.30 | 1.00 | 0.13 |
| -73.26008179 | 19.69479904 | 0.03 | 0.04 | 0.06 | 0.06 | 0.26 | 0.21 | 0.12 | 0.30 | 1.00 | 0.50 |
| -73.4085733 | 19.78804416 | 0.03 | 0.04 | 0.07 | 0.07 | 0.27 | 0.23 | 0.13 | 0.30 | 1.00 | 0.07 |
| -72.19719514 | 18.22578405 | 0.02 | 0.03 | 0.05 | 0.04 | 0.27 | 0.17 | 0.09 | 0.30 | 1.00 | 0.67 |
| -73.35332691 | 19.81903604 | 0.02 | 0.02 | 0.04 | 0.04 | 0.21 | 0.17 | 0.09 | 0.30 | 1.00 | 0.52 |
| -72.19746464 | 18.22605355 | 0.02 | 0.03 | 0.05 | 0.04 | 0.29 | 0.18 | 0.09 | 0.30 | 1.00 | 0.13 |
| -73.40749533 | 19.78831366 | 0.03 | 0.04 | 0.07 | 0.07 | 0.25 | 0.23 | 0.12 | 0.30 | 1.00 | 0.32 |
| -72.82188359 | 18.14493568 | 0.03 | 0.03 | 0.06 | 0.05 | 0.35 | 0.21 | 0.10 | 0.30 | 1.00 | 0.12 |
| -73.36114226 | 19.6268864 | 0.07 | 0.08 | 0.11 | 0.14 | 0.25 | 0.25 | 0.18 | 0.30 | 1.00 | 0.01 |
| -73.40318341 | 19.83817016 | 0.01 | 0.03 | 0.06 | 0.06 | 0.23 | 0.20 | 0.12 | 0.30 | 1.00 | 0.43 |
| -73.40129695 | 19.7912781 | 0.03 | 0.04 | 0.07 | 0.07 | 0.25 | 0.25 | 0.14 | 0.30 | 1.00 | 0.22 |
| -73.26008179 | 19.69506853 | 0.03 | 0.04 | 0.06 | 0.06 | 0.25 | 0.21 | 0.12 | 0.30 | 1.00 | 0.58 |
| -73.45438738 | 19.7414216 | 0.03 | 0.04 | 0.06 | 0.06 | 0.22 | 0.13 | 0.08 | 0.30 | 1.00 | 0.55 |
| -73.36141175 | 19.62742539 | 0.04 | 0.05 | 0.08 | 0.08 | 0.24 | 0.18 | 0.11 | 0.30 | 1.00 | 0.27 |
| -72.1993511 | 18.22443658 | 0.02 | 0.02 | 0.05 | 0.03 | 0.25 | 0.15 | 0.07 | 0.30 | 1.00 | 0.50 |
| -73.45492637 | 19.74249958 | 0.03 | 0.04 | 0.05 | 0.06 | 0.18 | 0.12 | 0.07 | 0.30 | 1.00 | 0.19 |
| -72.19962059 | 18.22443658 | 0.02 | 0.02 | 0.04 | 0.02 | 0.26 | 0.14 | 0.06 | 0.30 | 1.00 | 0.50 |
| -73.37758143 | 19.82658189 | 0.02 | 0.03 | 0.07 | 0.07 | 0.25 | 0.24 | 0.14 | 0.30 | 1.00 | 0.29 |
| -73.13045489 | 19.92440842 | 0.05 | 0.07 | 0.10 | 0.12 | 0.28 | 0.30 | 0.19 | 0.30 | 1.00 | 0.70 |
| -72.92456103 | 18.16487828 | 0.02 | 0.02 | 0.05 | 0.04 | 0.36 | 0.18 | 0.08 | 0.30 | 1.00 | 0.61 |
| -72.19962059 | 18.22416708 | 0.02 | 0.03 | 0.05 | 0.04 | 0.24 | 0.16 | 0.08 | 0.30 | 1.00 | 0.51 |
| -73.25981229 | 19.69479904 | 0.04 | 0.04 | 0.07 | 0.07 | 0.27 | 0.24 | 0.14 | 0.30 | 1.00 | 0.05 |
| -73.37731193 | 19.82658189 | 0.02 | 0.03 | 0.07 | 0.07 | 0.25 | 0.24 | 0.14 | 0.30 | 1.00 | 0.53 |
| -73.25981229 | 19.69452954 | 0.03 | 0.04 | 0.06 | 0.06 | 0.26 | 0.22 | 0.12 | 0.30 | 1.00 | 0.21 |
| -73.35521338 | 19.75786077 | 0.04 | 0.04 | 0.06 | 0.06 | 0.27 | 0.25 | 0.14 | 0.30 | 1.00 | 0.60 |
| -73.05257096 | 19.89206907 | 0.03 | 0.05 | 0.10 | 0.12 | 0.24 | 0.28 | 0.19 | 0.30 | 1.00 | 0.48 |
| -73.42770742 | 19.78804416 | 0.03 | 0.05 | 0.09 | 0.09 | 0.25 | 0.28 | 0.17 | 0.30 | 1.00 | 0.61 |
| -72.8216141 | 18.14466618 | 0.02 | 0.03 | 0.06 | 0.05 | 0.38 | 0.21 | 0.11 | 0.30 | 1.00 | 0.56 |
| -73.45034496 | 19.7610947 | 0.03 | 0.05 | 0.07 | 0.08 | 0.22 | 0.17 | 0.10 | 0.30 | 1.00 | 0.19 |
| -73.29322962 | 19.85999922 | 0.03 | 0.04 | 0.07 | 0.07 | 0.27 | 0.22 | 0.12 | 0.30 | 1.00 | 0.16 |
| -73.36545417 | 19.63012034 | 0.04 | 0.05 | 0.07 | 0.08 | 0.23 | 0.19 | 0.12 | 0.30 | 1.00 | 0.05 |
| -73.4085733 | 19.78858315 | 0.03 | 0.04 | 0.07 | 0.07 | 0.26 | 0.25 | 0.13 | 0.30 | 1.00 | 0.60 |
| -73.00594839 | 19.91605409 | 0.05 | 0.07 | 0.10 | 0.13 | 0.25 | 0.30 | 0.21 | 0.30 | 1.00 | 0.50 |
| -73.18543179 | 19.9058133 | 0.06 | 0.07 | 0.10 | 0.11 | 0.28 | 0.32 | 0.20 | 0.30 | 1.00 | 0.12 |
| -73.31883161 | 19.62958135 | 0.05 | 0.06 | 0.07 | 0.08 | 0.22 | 0.18 | 0.11 | 0.30 | 1.00 | 0.16 |
| -73.35440489 | 19.81822756 | 0.01 | 0.02 | 0.04 | 0.04 | 0.22 | 0.16 | 0.09 | 0.30 | 1.00 | 0.09 |
| -73.45007547 | 19.7613642 | 0.02 | 0.03 | 0.06 | 0.06 | 0.24 | 0.14 | 0.08 | 0.30 | 1.00 | 0.19 |
| -72.88683179 | 18.84157918 | 0.09 | 0.11 | 0.16 | 0.19 | 0.35 | 0.39 | 0.30 | 0.30 | 1.00 | 0.28 |
| -73.0054094 | 19.9155151 | 0.05 | 0.07 | 0.10 | 0.12 | 0.25 | 0.29 | 0.20 | 0.30 | 1.00 | 0.11 |
| -73.35467439 | 19.75786077 | 0.03 | 0.04 | 0.06 | 0.06 | 0.27 | 0.25 | 0.14 | 0.30 | 1.00 | 0.20 |
| -73.35467439 | 19.81795806 | 0.01 | 0.02 | 0.04 | 0.04 | 0.23 | 0.17 | 0.09 | 0.30 | 1.00 | 0.12 |
| -72.78550182 | 20.07586438 | 0.03 | 0.04 | 0.06 | 0.05 | 0.34 | 0.21 | 0.10 | 0.30 | 1.00 | 0.46 |
| -72.88790976 | 18.84211817 | 0.06 | 0.09 | 0.14 | 0.15 | 0.36 | 0.31 | 0.20 | 0.30 | 1.00 | 0.37 |
| -73.35494388 | 19.81795806 | 0.02 | 0.02 | 0.05 | 0.05 | 0.24 | 0.19 | 0.11 | 0.30 | 1.00 | 0.45 |
| -73.45438738 | 19.74169109 | 0.03 | 0.04 | 0.06 | 0.06 | 0.21 | 0.12 | 0.07 | 0.30 | 1.00 | 0.19 |
| -73.45438738 | 19.7411521 | 0.04 | 0.05 | 0.07 | 0.07 | 0.22 | 0.15 | 0.09 | 0.30 | 1.00 | 0.40 |
| -72.8213446 | 18.14466618 | 0.03 | 0.04 | 0.06 | 0.05 | 0.38 | 0.21 | 0.10 | 0.30 | 1.00 | 0.49 |
| -72.19854262 | 18.22524506 | 0.02 | 0.02 | 0.05 | 0.03 | 0.28 | 0.15 | 0.07 | 0.30 | 1.00 | 0.54 |
| -73.1053919 | 19.91605409 | 0.05 | 0.06 | 0.10 | 0.13 | 0.28 | 0.31 | 0.21 | 0.30 | 1.00 | 0.41 |
| -72.82107511 | 18.14439669 | 0.02 | 0.03 | 0.06 | 0.04 | 0.37 | 0.18 | 0.08 | 0.30 | 1.00 | 0.52 |
| -73.0056789 | 19.9155151 | 0.04 | 0.05 | 0.09 | 0.09 | 0.24 | 0.27 | 0.18 | 0.30 | 1.00 | 0.69 |
| -72.78496283 | 20.0750559 | 0.03 | 0.03 | 0.06 | 0.05 | 0.35 | 0.21 | 0.10 | 0.30 | 1.00 | 0.35 |
| -73.40776482 | 19.78831366 | 0.03 | 0.04 | 0.07 | 0.07 | 0.27 | 0.25 | 0.13 | 0.30 | 1.00 | 0.42 |
| -73.34066067 | 19.62904236 | 0.06 | 0.06 | 0.08 | 0.09 | 0.18 | 0.16 | 0.11 | 0.30 | 1.00 | 0.28 |
| -73.40291392 | 19.83817016 | 0.02 | 0.03 | 0.06 | 0.07 | 0.23 | 0.22 | 0.13 | 0.30 | 1.00 | 0.61 |
| -73.3541354 | 19.81849705 | 0.01 | 0.02 | 0.04 | 0.04 | 0.21 | 0.16 | 0.08 | 0.30 | 1.00 | 0.29 |
| -73.37704244 | 19.82631239 | 0.02 | 0.03 | 0.06 | 0.06 | 0.24 | 0.24 | 0.14 | 0.30 | 1.00 | 0.28 |
| -73.35548287 | 19.75839976 | 0.04 | 0.05 | 0.07 | 0.07 | 0.28 | 0.28 | 0.16 | 0.30 | 1.00 | 0.05 |
| -72.81487673 | 20.08853063 | 0.03 | 0.05 | 0.09 | 0.09 | 0.33 | 0.26 | 0.15 | 0.30 | 1.00 | 0.07 |
| -73.40722583 | 19.78777467 | 0.02 | 0.02 | 0.05 | 0.04 | 0.22 | 0.16 | 0.08 | 0.30 | 1.00 | 0.41 |
| -73.10431392 | 19.91632359 | 0.04 | 0.06 | 0.09 | 0.10 | 0.26 | 0.29 | 0.18 | 0.30 | 1.00 | 0.47 |
| -72.78550182 | 20.07559489 | 0.03 | 0.03 | 0.06 | 0.06 | 0.34 | 0.22 | 0.11 | 0.30 | 1.00 | 0.43 |
| -72.81487673 | 20.08853063 | 0.03 | 0.05 | 0.09 | 0.09 | 0.33 | 0.26 | 0.15 | 0.30 | 1.00 | 0.55 |
| -73.4336363 | 19.7613642 | 0.05 | 0.05 | 0.07 | 0.07 | 0.27 | 0.26 | 0.15 | 0.30 | 1.00 | 0.43 |
| -73.0054094 | 19.9157846 | 0.06 | 0.08 | 0.13 | 0.15 | 0.27 | 0.34 | 0.25 | 0.30 | 1.00 | 0.62 |
| -73.35440489 | 19.81849705 | 0.02 | 0.03 | 0.05 | 0.06 | 0.23 | 0.20 | 0.12 | 0.30 | 1.00 | 0.55 |
| -73.05176247 | 19.89206907 | 0.02 | 0.05 | 0.09 | 0.09 | 0.22 | 0.23 | 0.15 | 0.30 | 1.00 | 0.21 |
| -72.19719514 | 18.22578405 | 0.02 | 0.03 | 0.05 | 0.04 | 0.27 | 0.17 | 0.09 | 0.30 | 1.00 | 0.14 |
| -73.45034496 | 19.76163369 | 0.03 | 0.04 | 0.07 | 0.07 | 0.24 | 0.16 | 0.09 | 0.30 | 1.00 | 0.52 |
| -73.40776482 | 19.78777467 | 0.02 | 0.03 | 0.05 | 0.05 | 0.22 | 0.19 | 0.11 | 0.30 | 1.00 | 0.64 |
| -73.40264442 | 19.83870914 | 0.04 | 0.05 | 0.08 | 0.09 | 0.26 | 0.25 | 0.15 | 0.30 | 1.00 | 0.03 |
| -73.2595428 | 19.69506853 | 0.04 | 0.04 | 0.07 | 0.07 | 0.27 | 0.24 | 0.14 | 0.30 | 1.00 | 0.36 |
| -73.18543179 | 19.90635229 | 0.06 | 0.07 | 0.10 | 0.11 | 0.27 | 0.32 | 0.20 | 0.30 | 1.00 | 0.35 |
| -73.32583847 | 19.62904236 | 0.05 | 0.05 | 0.07 | 0.07 | 0.18 | 0.14 | 0.09 | 0.30 | 1.00 | 0.37 |
| -73.43336681 | 19.76163369 | 0.04 | 0.05 | 0.07 | 0.07 | 0.27 | 0.23 | 0.14 | 0.30 | 1.00 | 0.58 |
| -72.85071951 | 18.14816961 | 0.03 | 0.04 | 0.08 | 0.08 | 0.34 | 0.21 | 0.11 | 0.30 | 1.00 | 0.05 |
| -72.78631031 | 20.0747864 | 0.04 | 0.05 | 0.08 | 0.08 | 0.38 | 0.26 | 0.14 | 0.30 | 1.00 | 0.32 |
| -73.45034496 | 19.76163369 | 0.03 | 0.04 | 0.07 | 0.07 | 0.24 | 0.16 | 0.09 | 0.30 | 1.00 | 0.45 |
| -73.18597078 | 19.90635229 | 0.06 | 0.08 | 0.11 | 0.13 | 0.29 | 0.33 | 0.21 | 0.30 | 1.00 | 0.28 |
| -72.85098901 | 18.14843911 | 0.03 | 0.04 | 0.07 | 0.06 | 0.34 | 0.21 | 0.11 | 0.30 | 1.00 | 0.63 |
| -73.26035128 | 19.69426005 | 0.03 | 0.04 | 0.06 | 0.06 | 0.26 | 0.21 | 0.12 | 0.30 | 1.00 | 0.53 |
| -73.37758143 | 19.82685138 | 0.02 | 0.03 | 0.06 | 0.07 | 0.24 | 0.24 | 0.14 | 0.30 | 1.00 | 0.48 |
| -73.38027637 | 19.83897864 | 0.02 | 0.03 | 0.06 | 0.07 | 0.23 | 0.22 | 0.12 | 0.30 | 1.00 | 0.15 |
| -73.05203197 | 19.89233857 | 0.03 | 0.04 | 0.08 | 0.09 | 0.22 | 0.22 | 0.14 | 0.30 | 1.00 | 0.26 |
| -73.40722583 | 19.78831366 | 0.03 | 0.03 | 0.06 | 0.06 | 0.25 | 0.22 | 0.12 | 0.30 | 1.00 | 0.53 |
| -73.25981229 | 19.69479904 | 0.04 | 0.04 | 0.07 | 0.07 | 0.27 | 0.24 | 0.14 | 0.30 | 1.00 | 0.57 |
| -72.8213446 | 18.14466618 | 0.03 | 0.04 | 0.06 | 0.05 | 0.38 | 0.21 | 0.10 | 0.30 | 1.00 | 0.20 |
| -72.78765778 | 20.07532539 | 0.03 | 0.03 | 0.06 | 0.05 | 0.31 | 0.21 | 0.10 | 0.30 | 1.00 | 0.16 |
| -73.40318341 | 19.83817016 | 0.01 | 0.03 | 0.06 | 0.06 | 0.23 | 0.20 | 0.12 | 0.30 | 1.00 | 0.26 |
| -73.36087276 | 19.6268864 | 0.06 | 0.08 | 0.11 | 0.14 | 0.25 | 0.25 | 0.18 | 0.30 | 1.00 | 0.01 |
| -73.12964641 | 19.92467792 | 0.07 | 0.10 | 0.15 | 0.18 | 0.31 | 0.35 | 0.24 | 0.30 | 1.00 | 0.10 |
| -73.18597078 | 19.90662178 | 0.07 | 0.08 | 0.12 | 0.14 | 0.30 | 0.35 | 0.23 | 0.30 | 1.00 | 0.46 |
| -72.78711879 | 20.07532539 | 0.02 | 0.03 | 0.06 | 0.05 | 0.31 | 0.21 | 0.10 | 0.30 | 1.00 | 0.24 |
| -72.19800363 | 18.22578405 | 0.03 | 0.04 | 0.06 | 0.05 | 0.33 | 0.20 | 0.10 | 0.30 | 1.00 | 0.58 |
| -73.37704244 | 19.82658189 | 0.02 | 0.04 | 0.07 | 0.07 | 0.24 | 0.23 | 0.13 | 0.30 | 1.00 | 0.52 |
| -73.42797691 | 19.78804416 | 0.04 | 0.05 | 0.09 | 0.10 | 0.25 | 0.28 | 0.17 | 0.30 | 1.00 | 0.29 |
| -73.36087276 | 19.6268864 | 0.06 | 0.08 | 0.11 | 0.14 | 0.25 | 0.25 | 0.18 | 0.30 | 1.00 | 0.57 |
| -73.3193706 | 19.62958135 | 0.06 | 0.07 | 0.09 | 0.09 | 0.23 | 0.20 | 0.13 | 0.30 | 1.00 | 0.59 |
| -72.78711879 | 20.07532539 | 0.02 | 0.03 | 0.06 | 0.05 | 0.31 | 0.21 | 0.10 | 0.30 | 1.00 | 0.23 |
| -73.45492637 | 19.7414216 | 0.03 | 0.04 | 0.07 | 0.07 | 0.21 | 0.13 | 0.08 | 0.30 | 1.00 | 0.22 |
| -72.78604081 | 20.07532539 | 0.02 | 0.03 | 0.06 | 0.05 | 0.35 | 0.22 | 0.10 | 0.30 | 1.00 | 0.02 |
| -72.20177655 | 18.52384506 | 0.04 | 0.05 | 0.08 | 0.08 | 0.24 | 0.21 | 0.14 | 0.30 | 2.00 | 0.27 |
| -72.66395976 | 18.46779019 | 0.02 | 0.04 | 0.07 | 0.06 | 0.36 | 0.19 | 0.09 | 0.30 | 2.00 | 0.66 |
| -72.20231554 | 18.52492304 | 0.04 | 0.05 | 0.08 | 0.09 | 0.24 | 0.21 | 0.15 | 0.30 | 2.00 | 0.06 |
| -72.05193756 | 19.64224759 | 0.03 | 0.04 | 0.07 | 0.09 | 0.24 | 0.28 | 0.17 | 0.30 | 2.00 | 0.56 |
| -72.04870363 | 19.64359507 | 0.03 | 0.04 | 0.07 | 0.07 | 0.26 | 0.26 | 0.14 | 0.30 | 2.00 | 0.51 |
| -72.50684442 | 19.15688784 | 0.02 | 0.02 | 0.06 | 0.05 | 0.23 | 0.11 | 0.06 | 0.30 | 2.00 | 0.22 |
| -72.20150706 | 18.52546203 | 0.04 | 0.05 | 0.08 | 0.07 | 0.21 | 0.20 | 0.13 | 0.30 | 2.00 | 0.35 |
| -72.66288179 | 18.46940716 | 0.02 | 0.03 | 0.08 | 0.06 | 0.34 | 0.21 | 0.10 | 0.30 | 2.00 | 0.31 |
| -72.52328359 | 19.15015048 | 0.04 | 0.04 | 0.08 | 0.07 | 0.32 | 0.21 | 0.10 | 0.30 | 2.00 | 0.58 |
| -72.19476969 | 18.53866727 | 0.04 | 0.05 | 0.09 | 0.10 | 0.26 | 0.23 | 0.15 | 0.30 | 2.00 | 0.14 |
| -72.15218955 | 19.70989073 | 0.05 | 0.05 | 0.08 | 0.09 | 0.21 | 0.28 | 0.22 | 0.30 | 2.00 | 0.30 |
| -72.50657493 | 19.16793712 | 0.02 | 0.03 | 0.06 | 0.05 | 0.23 | 0.14 | 0.06 | 0.30 | 2.00 | 0.34 |
| -72.05247655 | 19.64224759 | 0.03 | 0.05 | 0.07 | 0.09 | 0.24 | 0.30 | 0.19 | 0.30 | 2.00 | 0.45 |
| -72.20096807 | 18.52600102 | 0.04 | 0.05 | 0.08 | 0.08 | 0.23 | 0.20 | 0.13 | 0.30 | 2.00 | 0.39 |
| -72.20015958 | 18.52438405 | 0.04 | 0.05 | 0.08 | 0.08 | 0.26 | 0.21 | 0.14 | 0.30 | 2.00 | 0.50 |
| -72.50630543 | 19.15607936 | 0.02 | 0.03 | 0.05 | 0.05 | 0.18 | 0.11 | 0.06 | 0.30 | 2.00 | 0.14 |
| -72.52328359 | 19.15068947 | 0.04 | 0.05 | 0.08 | 0.07 | 0.29 | 0.20 | 0.11 | 0.30 | 2.00 | 0.60 |
| -72.1990816 | 18.52411456 | 0.03 | 0.04 | 0.06 | 0.06 | 0.20 | 0.17 | 0.11 | 0.30 | 2.00 | 0.55 |
| -72.0500511 | 19.6417086 | 0.03 | 0.04 | 0.08 | 0.09 | 0.26 | 0.27 | 0.16 | 0.30 | 2.00 | 0.69 |
| -72.04843413 | 19.64467304 | 0.03 | 0.04 | 0.07 | 0.07 | 0.27 | 0.27 | 0.15 | 0.30 | 2.00 | 0.22 |
| -72.20015958 | 18.54432665 | 0.04 | 0.05 | 0.09 | 0.09 | 0.28 | 0.21 | 0.13 | 0.30 | 2.00 | 0.39 |
| -72.58904027 | 19.16066077 | 0.03 | 0.04 | 0.07 | 0.07 | 0.26 | 0.15 | 0.07 | 0.30 | 2.00 | 0.48 |
| -72.0500511 | 19.64224759 | 0.03 | 0.03 | 0.06 | 0.06 | 0.28 | 0.25 | 0.13 | 0.30 | 2.00 | 0.20 |
| -72.57098413 | 19.16766763 | 0.03 | 0.04 | 0.08 | 0.07 | 0.26 | 0.16 | 0.07 | 0.30 | 2.00 | 0.54 |
| -72.56990615 | 19.16766763 | 0.02 | 0.03 | 0.07 | 0.06 | 0.22 | 0.13 | 0.06 | 0.30 | 2.00 | 0.38 |
| -72.58877078 | 18.5370503 | 0.04 | 0.05 | 0.08 | 0.07 | 0.31 | 0.22 | 0.11 | 0.30 | 2.00 | 0.06 |
| -72.66746319 | 18.46940716 | 0.02 | 0.03 | 0.06 | 0.05 | 0.31 | 0.18 | 0.09 | 0.30 | 2.00 | 0.60 |
| -72.19584767 | 18.53785878 | 0.04 | 0.05 | 0.09 | 0.09 | 0.30 | 0.24 | 0.15 | 0.30 | 2.00 | 0.37 |
| -72.57960796 | 19.16847611 | 0.03 | 0.03 | 0.07 | 0.06 | 0.28 | 0.15 | 0.07 | 0.30 | 2.00 | 0.65 |
| -72.21390381 | 19.69129561 | 0.03 | 0.04 | 0.08 | 0.07 | 0.33 | 0.26 | 0.13 | 0.30 | 2.00 | 0.08 |
| -72.58661482 | 19.15500138 | 0.02 | 0.03 | 0.07 | 0.05 | 0.30 | 0.15 | 0.08 | 0.30 | 2.00 | 0.66 |
| -72.4828594 | 19.09759904 | 0.03 | 0.04 | 0.08 | 0.07 | 0.29 | 0.20 | 0.10 | 0.30 | 2.00 | 0.59 |
| -72.19396121 | 18.59795607 | 0.05 | 0.06 | 0.09 | 0.10 | 0.24 | 0.22 | 0.15 | 0.30 | 2.00 | 0.60 |
| -72.66611572 | 18.46940716 | 0.03 | 0.04 | 0.08 | 0.08 | 0.33 | 0.26 | 0.13 | 0.30 | 2.00 | 0.38 |
| -72.19261373 | 18.59984254 | 0.04 | 0.05 | 0.09 | 0.08 | 0.26 | 0.21 | 0.14 | 0.30 | 2.00 | 0.55 |
| -73.77427746 | 18.20314651 | 0.03 | 0.04 | 0.08 | 0.07 | 0.31 | 0.18 | 0.09 | 0.30 | 2.00 | 0.02 |
| -72.21767673 | 19.68967864 | 0.03 | 0.04 | 0.08 | 0.08 | 0.34 | 0.26 | 0.14 | 0.30 | 2.00 | 0.59 |
| -72.66638522 | 18.46940716 | 0.03 | 0.04 | 0.08 | 0.07 | 0.33 | 0.23 | 0.12 | 0.30 | 2.00 | 0.30 |
| -72.21659875 | 19.68887016 | 0.03 | 0.04 | 0.08 | 0.07 | 0.35 | 0.25 | 0.13 | 0.30 | 2.00 | 0.18 |
| -72.5230141 | 19.15068947 | 0.04 | 0.05 | 0.08 | 0.08 | 0.30 | 0.21 | 0.11 | 0.30 | 2.00 | 0.10 |
| -72.05301554 | 19.64278658 | 0.03 | 0.04 | 0.07 | 0.08 | 0.24 | 0.29 | 0.18 | 0.30 | 2.00 | 0.32 |
| -72.21579027 | 19.69129561 | 0.02 | 0.03 | 0.07 | 0.05 | 0.35 | 0.20 | 0.09 | 0.30 | 2.00 | 0.17 |
| -72.19207475 | 18.59930355 | 0.05 | 0.06 | 0.09 | 0.10 | 0.27 | 0.22 | 0.15 | 0.30 | 2.00 | 0.47 |
| -72.19234424 | 18.60011203 | 0.04 | 0.05 | 0.08 | 0.09 | 0.26 | 0.20 | 0.14 | 0.30 | 2.00 | 0.20 |
| -72.05247655 | 19.64305608 | 0.03 | 0.04 | 0.07 | 0.07 | 0.25 | 0.29 | 0.18 | 0.30 | 2.00 | 0.64 |
| -72.5825724 | 18.48611582 | 0.04 | 0.04 | 0.07 | 0.07 | 0.30 | 0.21 | 0.12 | 0.30 | 2.00 | 0.22 |
| -72.20150706 | 18.54459615 | 0.04 | 0.05 | 0.09 | 0.09 | 0.26 | 0.21 | 0.14 | 0.30 | 2.00 | 0.25 |
| -72.21767673 | 19.69021763 | 0.02 | 0.03 | 0.07 | 0.06 | 0.35 | 0.22 | 0.10 | 0.30 | 2.00 | 0.05 |
| -72.66422926 | 18.4675207 | 0.02 | 0.03 | 0.07 | 0.05 | 0.37 | 0.17 | 0.07 | 0.30 | 2.00 | 0.49 |
| -72.04897312 | 19.64332557 | 0.03 | 0.03 | 0.06 | 0.06 | 0.27 | 0.26 | 0.14 | 0.30 | 2.00 | 0.40 |
| -72.57933846 | 19.1690151 | 0.03 | 0.04 | 0.07 | 0.06 | 0.24 | 0.13 | 0.07 | 0.30 | 2.00 | 0.30 |
| -72.20123756 | 18.54459615 | 0.04 | 0.05 | 0.09 | 0.09 | 0.29 | 0.21 | 0.13 | 0.30 | 2.00 | 0.25 |
| -72.19261373 | 18.59984254 | 0.04 | 0.05 | 0.09 | 0.08 | 0.26 | 0.21 | 0.14 | 0.30 | 2.00 | 0.48 |
| -72.0500511 | 19.64467304 | 0.02 | 0.03 | 0.06 | 0.07 | 0.28 | 0.25 | 0.14 | 0.30 | 2.00 | 0.62 |
| -72.04951211 | 19.64386456 | 0.02 | 0.04 | 0.07 | 0.08 | 0.25 | 0.26 | 0.15 | 0.30 | 2.00 | 0.25 |
| -72.58014695 | 19.16632015 | 0.03 | 0.04 | 0.07 | 0.07 | 0.27 | 0.16 | 0.08 | 0.30 | 2.00 | 0.00 |
| -72.1942307 | 18.53785878 | 0.05 | 0.06 | 0.10 | 0.10 | 0.31 | 0.26 | 0.17 | 0.30 | 2.00 | 0.29 |
| -72.56316879 | 19.1641642 | 0.03 | 0.04 | 0.08 | 0.07 | 0.25 | 0.16 | 0.08 | 0.30 | 2.00 | 0.30 |
| -72.5329854 | 18.79819055 | 0.06 | 0.07 | 0.11 | 0.11 | 0.32 | 0.30 | 0.18 | 0.30 | 2.00 | 0.16 |
| -72.58203341 | 18.48611582 | 0.04 | 0.04 | 0.07 | 0.06 | 0.31 | 0.22 | 0.12 | 0.30 | 2.00 | 0.58 |
| -72.05247655 | 19.64224759 | 0.03 | 0.05 | 0.07 | 0.09 | 0.24 | 0.30 | 0.19 | 0.30 | 2.00 | 0.07 |
| -72.05193756 | 19.64251709 | 0.03 | 0.04 | 0.07 | 0.08 | 0.25 | 0.27 | 0.16 | 0.30 | 2.00 | 0.18 |
| -72.19503919 | 18.59957304 | 0.04 | 0.06 | 0.10 | 0.11 | 0.28 | 0.22 | 0.14 | 0.30 | 2.00 | 0.58 |
| -72.50630543 | 19.16793712 | 0.02 | 0.03 | 0.06 | 0.05 | 0.24 | 0.14 | 0.06 | 0.30 | 2.00 | 0.30 |
| -72.58014695 | 18.48692431 | 0.04 | 0.05 | 0.09 | 0.09 | 0.31 | 0.24 | 0.14 | 0.30 | 2.00 | 0.40 |
| -72.05274605 | 19.64629001 | 0.03 | 0.04 | 0.07 | 0.08 | 0.28 | 0.26 | 0.15 | 0.30 | 2.00 | 0.10 |
| -72.66476825 | 18.46967665 | 0.02 | 0.03 | 0.07 | 0.05 | 0.37 | 0.17 | 0.07 | 0.30 | 2.00 | 0.49 |
| -72.19396121 | 18.59849506 | 0.05 | 0.06 | 0.09 | 0.09 | 0.26 | 0.22 | 0.15 | 0.30 | 2.00 | 0.65 |
| -72.21498179 | 19.68913965 | 0.03 | 0.03 | 0.07 | 0.06 | 0.32 | 0.22 | 0.11 | 0.30 | 2.00 | 0.16 |
| -72.58796229 | 18.5370503 | 0.04 | 0.05 | 0.08 | 0.07 | 0.31 | 0.22 | 0.11 | 0.30 | 2.00 | 0.67 |
| -72.2144428 | 19.69102611 | 0.03 | 0.04 | 0.08 | 0.06 | 0.28 | 0.24 | 0.13 | 0.30 | 2.00 | 0.60 |
| -72.05032059 | 19.64251709 | 0.02 | 0.04 | 0.07 | 0.07 | 0.28 | 0.26 | 0.15 | 0.30 | 2.00 | 0.65 |
| -72.20015958 | 18.54351817 | 0.04 | 0.05 | 0.09 | 0.10 | 0.27 | 0.21 | 0.14 | 0.30 | 2.00 | 0.42 |
| -72.52328359 | 19.15068947 | 0.04 | 0.05 | 0.08 | 0.07 | 0.29 | 0.20 | 0.11 | 0.30 | 2.00 | 0.62 |
| -72.21686825 | 19.6915651 | 0.02 | 0.02 | 0.08 | 0.04 | 0.42 | 0.18 | 0.08 | 0.30 | 2.00 | 0.01 |
| -72.05166807 | 19.64332557 | 0.03 | 0.04 | 0.07 | 0.07 | 0.26 | 0.26 | 0.15 | 0.30 | 2.00 | 0.53 |
| -72.58877078 | 18.5370503 | 0.04 | 0.05 | 0.08 | 0.07 | 0.31 | 0.22 | 0.11 | 0.30 | 2.00 | 0.48 |
| -72.2144428 | 19.6918346 | 0.03 | 0.03 | 0.07 | 0.06 | 0.32 | 0.21 | 0.11 | 0.30 | 2.00 | 0.26 |
| -72.0497816 | 19.64278658 | 0.03 | 0.04 | 0.07 | 0.07 | 0.27 | 0.25 | 0.13 | 0.30 | 2.00 | 0.48 |
| -72.21740724 | 19.68994813 | 0.02 | 0.03 | 0.07 | 0.05 | 0.34 | 0.21 | 0.10 | 0.30 | 2.00 | 0.20 |
| -72.60170652 | 19.15473189 | 0.03 | 0.04 | 0.07 | 0.06 | 0.26 | 0.18 | 0.11 | 0.30 | 2.00 | 0.58 |
| -72.58634532 | 19.1541929 | 0.02 | 0.03 | 0.07 | 0.06 | 0.27 | 0.16 | 0.08 | 0.30 | 2.00 | 0.68 |
| -72.20096807 | 18.52357557 | 0.04 | 0.04 | 0.07 | 0.07 | 0.23 | 0.19 | 0.12 | 0.30 | 2.00 | 0.02 |
| -72.20231554 | 18.52411456 | 0.04 | 0.05 | 0.08 | 0.08 | 0.24 | 0.21 | 0.14 | 0.30 | 2.00 | 0.44 |
| -72.66557673 | 18.46913766 | 0.03 | 0.05 | 0.08 | 0.08 | 0.32 | 0.27 | 0.14 | 0.30 | 2.00 | 0.48 |
| -72.57906897 | 19.16685914 | 0.03 | 0.04 | 0.07 | 0.06 | 0.24 | 0.14 | 0.07 | 0.30 | 2.00 | 0.61 |
| -72.19288323 | 18.60011203 | 0.04 | 0.05 | 0.09 | 0.09 | 0.23 | 0.20 | 0.14 | 0.30 | 2.00 | 0.54 |
| -72.50630543 | 19.16766763 | 0.02 | 0.03 | 0.05 | 0.05 | 0.23 | 0.13 | 0.06 | 0.30 | 2.00 | 0.18 |
| -72.21552077 | 19.69129561 | 0.02 | 0.03 | 0.07 | 0.05 | 0.35 | 0.20 | 0.09 | 0.30 | 2.00 | 0.10 |
| -72.5227446 | 19.15068947 | 0.04 | 0.05 | 0.08 | 0.08 | 0.27 | 0.21 | 0.10 | 0.30 | 2.00 | 0.46 |
| -72.19315272 | 18.59903405 | 0.05 | 0.06 | 0.09 | 0.10 | 0.26 | 0.22 | 0.15 | 0.30 | 2.00 | 0.32 |
| -72.20096807 | 18.52465355 | 0.04 | 0.05 | 0.08 | 0.08 | 0.25 | 0.21 | 0.13 | 0.30 | 2.00 | 0.36 |
| -72.05139857 | 19.64494254 | 0.02 | 0.03 | 0.06 | 0.07 | 0.29 | 0.25 | 0.13 | 0.30 | 2.00 | 0.23 |
| -72.56343828 | 19.16362521 | 0.03 | 0.03 | 0.08 | 0.07 | 0.25 | 0.16 | 0.07 | 0.30 | 2.00 | 0.06 |
| -72.56182132 | 19.16443369 | 0.03 | 0.04 | 0.07 | 0.06 | 0.25 | 0.15 | 0.07 | 0.30 | 2.00 | 0.51 |
| -72.19261373 | 18.59849506 | 0.05 | 0.06 | 0.09 | 0.09 | 0.27 | 0.22 | 0.15 | 0.30 | 2.00 | 0.11 |
| -72.57826049 | 19.16605066 | 0.02 | 0.04 | 0.07 | 0.07 | 0.23 | 0.15 | 0.07 | 0.30 | 2.00 | 0.49 |
| -72.21740724 | 19.69048712 | 0.02 | 0.03 | 0.07 | 0.05 | 0.37 | 0.20 | 0.09 | 0.30 | 2.00 | 0.02 |
| -72.58095543 | 18.48665481 | 0.03 | 0.04 | 0.07 | 0.06 | 0.31 | 0.20 | 0.11 | 0.30 | 2.00 | 0.10 |
| -72.63674081 | 18.48099542 | 0.02 | 0.03 | 0.07 | 0.05 | 0.40 | 0.18 | 0.08 | 0.30 | 2.00 | 0.23 |
| -72.52355309 | 19.15041997 | 0.04 | 0.05 | 0.09 | 0.08 | 0.31 | 0.21 | 0.12 | 0.30 | 2.00 | 0.18 |
| -72.04951211 | 19.64521203 | 0.02 | 0.03 | 0.06 | 0.06 | 0.27 | 0.24 | 0.13 | 0.30 | 2.00 | 0.09 |
| -72.56316879 | 19.16497268 | 0.02 | 0.03 | 0.06 | 0.06 | 0.23 | 0.12 | 0.06 | 0.30 | 2.00 | 0.34 |
| -72.58930976 | 18.53570283 | 0.04 | 0.04 | 0.08 | 0.08 | 0.31 | 0.23 | 0.14 | 0.30 | 2.00 | 0.54 |
| -72.56343828 | 19.16470319 | 0.03 | 0.04 | 0.07 | 0.06 | 0.26 | 0.15 | 0.08 | 0.30 | 2.00 | 0.52 |
| -72.19315272 | 18.59930355 | 0.05 | 0.06 | 0.09 | 0.09 | 0.26 | 0.22 | 0.15 | 0.30 | 2.00 | 0.28 |
| -72.21605976 | 19.6918346 | 0.03 | 0.04 | 0.08 | 0.06 | 0.33 | 0.21 | 0.10 | 0.30 | 2.00 | 0.03 |
| -72.21767673 | 19.69021763 | 0.02 | 0.03 | 0.07 | 0.06 | 0.35 | 0.22 | 0.10 | 0.30 | 2.00 | 0.42 |
| -72.47693052 | 19.14179615 | 0.02 | 0.03 | 0.06 | 0.05 | 0.28 | 0.16 | 0.08 | 0.30 | 2.00 | 0.07 |
| -72.6022455 | 19.15473189 | 0.03 | 0.04 | 0.08 | 0.07 | 0.28 | 0.19 | 0.11 | 0.30 | 2.00 | 0.37 |
| -72.66584623 | 18.46859867 | 0.03 | 0.04 | 0.08 | 0.07 | 0.34 | 0.23 | 0.12 | 0.30 | 2.00 | 0.47 |
| -72.20177655 | 18.52492304 | 0.05 | 0.06 | 0.09 | 0.09 | 0.25 | 0.22 | 0.15 | 0.30 | 2.00 | 0.63 |
| -72.58095543 | 18.48692431 | 0.03 | 0.04 | 0.07 | 0.07 | 0.29 | 0.21 | 0.12 | 0.30 | 2.00 | 0.24 |
| -72.12631807 | 19.69749398 | 0.04 | 0.05 | 0.08 | 0.08 | 0.29 | 0.25 | 0.15 | 0.30 | 2.00 | 0.13 |
| -72.12577908 | 19.69749398 | 0.03 | 0.04 | 0.07 | 0.07 | 0.31 | 0.23 | 0.13 | 0.30 | 2.00 | 0.24 |
| -72.20015958 | 18.54432665 | 0.04 | 0.05 | 0.09 | 0.09 | 0.28 | 0.21 | 0.13 | 0.30 | 2.00 | 0.69 |
| -72.57044514 | 19.16739813 | 0.03 | 0.04 | 0.08 | 0.07 | 0.26 | 0.16 | 0.08 | 0.30 | 2.00 | 0.44 |
| -72.5076529 | 19.15527088 | 0.02 | 0.03 | 0.05 | 0.06 | 0.21 | 0.14 | 0.06 | 0.30 | 2.00 | 0.42 |
| -72.56424677 | 19.16362521 | 0.03 | 0.04 | 0.08 | 0.07 | 0.26 | 0.17 | 0.08 | 0.30 | 2.00 | 0.61 |
| -72.63701031 | 18.48180391 | 0.02 | 0.03 | 0.07 | 0.05 | 0.37 | 0.18 | 0.08 | 0.30 | 2.00 | 0.11 |
| -73.77454695 | 18.20314651 | 0.03 | 0.04 | 0.07 | 0.07 | 0.31 | 0.19 | 0.09 | 0.30 | 2.00 | 0.28 |
| -72.19288323 | 18.59903405 | 0.05 | 0.06 | 0.09 | 0.10 | 0.27 | 0.22 | 0.15 | 0.30 | 2.00 | 0.17 |
| -72.52220561 | 19.15041997 | 0.04 | 0.05 | 0.09 | 0.08 | 0.32 | 0.23 | 0.13 | 0.30 | 2.00 | 0.60 |
| -72.04843413 | 19.64386456 | 0.03 | 0.03 | 0.06 | 0.05 | 0.24 | 0.25 | 0.13 | 0.30 | 2.00 | 0.64 |
| -72.05166807 | 19.64548153 | 0.02 | 0.03 | 0.06 | 0.06 | 0.27 | 0.26 | 0.14 | 0.30 | 2.00 | 0.55 |
| -72.5076529 | 19.15554037 | 0.02 | 0.03 | 0.05 | 0.05 | 0.20 | 0.13 | 0.06 | 0.30 | 2.00 | 0.44 |
| -72.05085958 | 19.64332557 | 0.02 | 0.03 | 0.06 | 0.07 | 0.27 | 0.26 | 0.14 | 0.30 | 2.00 | 0.19 |
| -72.60170652 | 19.19677304 | 0.05 | 0.06 | 0.09 | 0.09 | 0.28 | 0.24 | 0.14 | 0.30 | 2.00 | 0.48 |
| -72.21605976 | 19.68967864 | 0.03 | 0.03 | 0.07 | 0.05 | 0.33 | 0.22 | 0.10 | 0.30 | 2.00 | 0.05 |
| -73.78424876 | 18.18023947 | 0.01 | 0.02 | 0.06 | 0.05 | 0.29 | 0.15 | 0.07 | 0.30 | 2.00 | 0.40 |
| -72.47720002 | 19.14179615 | 0.02 | 0.03 | 0.07 | 0.06 | 0.27 | 0.16 | 0.08 | 0.30 | 2.00 | 0.58 |
| -72.21632926 | 19.69021763 | 0.02 | 0.03 | 0.07 | 0.05 | 0.35 | 0.20 | 0.09 | 0.30 | 2.00 | 0.18 |
| -72.56074334 | 19.16470319 | 0.02 | 0.04 | 0.07 | 0.06 | 0.21 | 0.12 | 0.06 | 0.30 | 2.00 | 0.60 |
| -72.19315272 | 18.59957304 | 0.04 | 0.05 | 0.09 | 0.09 | 0.28 | 0.22 | 0.14 | 0.30 | 2.00 | 0.15 |
| -72.56909767 | 19.16874561 | 0.03 | 0.04 | 0.08 | 0.07 | 0.29 | 0.18 | 0.09 | 0.30 | 2.00 | 0.07 |
| -72.04843413 | 19.64143911 | 0.02 | 0.03 | 0.07 | 0.07 | 0.27 | 0.26 | 0.14 | 0.30 | 2.00 | 0.53 |
| -72.52247511 | 19.14988098 | 0.04 | 0.05 | 0.08 | 0.07 | 0.30 | 0.21 | 0.11 | 0.30 | 2.00 | 0.10 |
| -72.52112763 | 19.14988098 | 0.04 | 0.05 | 0.08 | 0.08 | 0.29 | 0.21 | 0.11 | 0.30 | 2.00 | 0.46 |
| -72.56370778 | 19.16443369 | 0.03 | 0.04 | 0.07 | 0.07 | 0.29 | 0.17 | 0.08 | 0.30 | 2.00 | 0.16 |
| -72.56236031 | 19.1638947 | 0.03 | 0.04 | 0.08 | 0.07 | 0.24 | 0.16 | 0.09 | 0.30 | 2.00 | 0.36 |
| -72.5227446 | 19.15015048 | 0.04 | 0.05 | 0.08 | 0.08 | 0.30 | 0.22 | 0.13 | 0.30 | 2.00 | 0.36 |
| -72.05220706 | 19.64440355 | 0.02 | 0.03 | 0.07 | 0.07 | 0.29 | 0.26 | 0.14 | 0.30 | 2.00 | 0.48 |
| -72.47773901 | 19.14206564 | 0.02 | 0.03 | 0.06 | 0.06 | 0.23 | 0.16 | 0.08 | 0.30 | 2.00 | 0.20 |
| -72.58904027 | 19.16173875 | 0.02 | 0.03 | 0.07 | 0.06 | 0.25 | 0.14 | 0.07 | 0.30 | 2.00 | 0.42 |
| -72.19989009 | 18.52519254 | 0.04 | 0.05 | 0.07 | 0.07 | 0.17 | 0.19 | 0.13 | 0.30 | 2.00 | 0.18 |
| -72.66611572 | 18.47021564 | 0.03 | 0.04 | 0.08 | 0.08 | 0.34 | 0.25 | 0.13 | 0.30 | 2.00 | 0.27 |
| -72.19342222 | 18.59957304 | 0.04 | 0.05 | 0.09 | 0.08 | 0.27 | 0.22 | 0.13 | 0.30 | 2.00 | 0.14 |
| -72.50684442 | 19.15446239 | 0.02 | 0.02 | 0.06 | 0.05 | 0.21 | 0.11 | 0.05 | 0.30 | 2.00 | 0.46 |
| -72.05085958 | 19.64359507 | 0.02 | 0.03 | 0.06 | 0.07 | 0.27 | 0.25 | 0.14 | 0.30 | 2.00 | 0.55 |
| -72.19261373 | 18.59876456 | 0.05 | 0.06 | 0.09 | 0.10 | 0.28 | 0.22 | 0.15 | 0.30 | 2.00 | 0.20 |
| -72.19530868 | 18.59930355 | 0.04 | 0.06 | 0.09 | 0.10 | 0.26 | 0.22 | 0.15 | 0.30 | 2.00 | 0.17 |
| -72.5876928 | 18.5370503 | 0.03 | 0.04 | 0.08 | 0.07 | 0.31 | 0.22 | 0.11 | 0.30 | 2.00 | 0.38 |
| -72.04870363 | 19.64251709 | 0.02 | 0.03 | 0.06 | 0.06 | 0.28 | 0.25 | 0.13 | 0.30 | 2.00 | 0.62 |
| -72.60278449 | 19.19596456 | 0.04 | 0.05 | 0.08 | 0.08 | 0.25 | 0.18 | 0.09 | 0.30 | 2.00 | 0.36 |
| -72.66530724 | 18.46940716 | 0.03 | 0.04 | 0.07 | 0.07 | 0.34 | 0.24 | 0.12 | 0.30 | 2.00 | 0.36 |
| -72.58957926 | 19.16146925 | 0.02 | 0.03 | 0.07 | 0.06 | 0.29 | 0.15 | 0.07 | 0.30 | 2.00 | 0.20 |
| -72.20069857 | 18.52465355 | 0.03 | 0.05 | 0.08 | 0.07 | 0.26 | 0.21 | 0.13 | 0.30 | 2.00 | 0.51 |
| -72.6375493 | 18.48099542 | 0.02 | 0.03 | 0.07 | 0.05 | 0.38 | 0.18 | 0.08 | 0.30 | 2.00 | 0.67 |
| -72.52247511 | 19.1490725 | 0.04 | 0.05 | 0.08 | 0.07 | 0.30 | 0.22 | 0.12 | 0.30 | 2.00 | 0.23 |
| -72.5230141 | 19.15041997 | 0.04 | 0.05 | 0.08 | 0.08 | 0.29 | 0.22 | 0.12 | 0.30 | 2.00 | 0.39 |
| -72.50711392 | 19.15634886 | 0.02 | 0.02 | 0.05 | 0.04 | 0.20 | 0.11 | 0.06 | 0.30 | 2.00 | 0.41 |
| -72.58122493 | 18.48611582 | 0.04 | 0.04 | 0.07 | 0.06 | 0.31 | 0.21 | 0.11 | 0.30 | 2.00 | 0.02 |
| -72.20177655 | 18.52384506 | 0.04 | 0.05 | 0.08 | 0.08 | 0.24 | 0.21 | 0.14 | 0.30 | 2.00 | 0.62 |
| -72.58823179 | 18.53731979 | 0.04 | 0.05 | 0.08 | 0.07 | 0.28 | 0.22 | 0.12 | 0.30 | 2.00 | 0.39 |
| -72.57852998 | 19.16820662 | 0.03 | 0.03 | 0.06 | 0.06 | 0.23 | 0.12 | 0.06 | 0.30 | 2.00 | 0.61 |
| -72.63781879 | 18.48153441 | 0.02 | 0.04 | 0.07 | 0.06 | 0.38 | 0.20 | 0.09 | 0.30 | 2.00 | 0.35 |
| -72.20204605 | 18.54378766 | 0.04 | 0.05 | 0.09 | 0.09 | 0.27 | 0.21 | 0.13 | 0.30 | 2.00 | 0.06 |
| -73.78424876 | 18.17996997 | 0.02 | 0.03 | 0.06 | 0.05 | 0.26 | 0.14 | 0.07 | 0.30 | 2.00 | 0.07 |
| -72.56182132 | 19.16524218 | 0.03 | 0.04 | 0.07 | 0.07 | 0.22 | 0.14 | 0.07 | 0.30 | 2.00 | 0.45 |
| -72.05166807 | 19.64305608 | 0.03 | 0.04 | 0.07 | 0.07 | 0.25 | 0.26 | 0.15 | 0.30 | 2.00 | 0.11 |
| -72.21686825 | 19.69021763 | 0.02 | 0.03 | 0.07 | 0.05 | 0.33 | 0.21 | 0.10 | 0.30 | 2.00 | 0.40 |
| -72.21471229 | 19.68994813 | 0.03 | 0.04 | 0.07 | 0.06 | 0.28 | 0.23 | 0.12 | 0.30 | 2.00 | 0.40 |
| -72.56936717 | 19.16739813 | 0.03 | 0.04 | 0.07 | 0.07 | 0.26 | 0.14 | 0.07 | 0.30 | 2.00 | 0.08 |
| -72.52139713 | 19.149342 | 0.04 | 0.05 | 0.08 | 0.09 | 0.29 | 0.21 | 0.14 | 0.30 | 2.00 | 0.55 |
| -72.21767673 | 19.68860066 | 0.04 | 0.05 | 0.09 | 0.09 | 0.33 | 0.31 | 0.19 | 0.30 | 2.00 | 0.47 |
| -72.21632926 | 19.68994813 | 0.02 | 0.03 | 0.07 | 0.05 | 0.32 | 0.20 | 0.09 | 0.30 | 2.00 | 0.51 |
| -72.56936717 | 19.1690151 | 0.03 | 0.04 | 0.07 | 0.07 | 0.26 | 0.18 | 0.09 | 0.30 | 2.00 | 0.19 |
| -72.04816464 | 19.64386456 | 0.03 | 0.03 | 0.05 | 0.06 | 0.22 | 0.25 | 0.13 | 0.30 | 2.00 | 0.49 |
| -72.58634532 | 19.15473189 | 0.03 | 0.04 | 0.08 | 0.07 | 0.26 | 0.15 | 0.07 | 0.30 | 2.00 | 0.04 |
| -72.5876928 | 18.53731979 | 0.03 | 0.04 | 0.08 | 0.07 | 0.31 | 0.22 | 0.11 | 0.30 | 2.00 | 0.34 |
| -72.20150706 | 18.54324867 | 0.04 | 0.06 | 0.09 | 0.10 | 0.27 | 0.21 | 0.14 | 0.30 | 2.00 | 0.25 |
| -72.5874233 | 19.15473189 | 0.02 | 0.03 | 0.07 | 0.05 | 0.27 | 0.15 | 0.07 | 0.30 | 2.00 | 0.42 |
| -72.50684442 | 19.15500138 | 0.02 | 0.03 | 0.06 | 0.05 | 0.20 | 0.11 | 0.05 | 0.30 | 2.00 | 0.35 |
| -72.56936717 | 19.1690151 | 0.03 | 0.04 | 0.07 | 0.07 | 0.26 | 0.18 | 0.09 | 0.30 | 2.00 | 0.03 |
| -72.58311139 | 18.48638532 | 0.04 | 0.04 | 0.07 | 0.06 | 0.33 | 0.21 | 0.12 | 0.30 | 2.00 | 0.68 |
| -72.19530868 | 18.5370503 | 0.04 | 0.05 | 0.09 | 0.08 | 0.30 | 0.23 | 0.14 | 0.30 | 2.00 | 0.15 |
| -72.56963666 | 19.16820662 | 0.03 | 0.04 | 0.07 | 0.07 | 0.27 | 0.16 | 0.08 | 0.30 | 2.00 | 0.51 |
| -72.19207475 | 18.59930355 | 0.05 | 0.06 | 0.09 | 0.10 | 0.27 | 0.22 | 0.15 | 0.30 | 2.00 | 0.24 |
| -72.53352439 | 18.79819055 | 0.07 | 0.08 | 0.11 | 0.13 | 0.32 | 0.34 | 0.21 | 0.30 | 2.00 | 0.23 |
| -72.14706915 | 19.7265994 | 0.03 | 0.04 | 0.08 | 0.07 | 0.34 | 0.24 | 0.12 | 0.30 | 2.00 | 0.64 |
| -72.20015958 | 18.52438405 | 0.04 | 0.05 | 0.08 | 0.08 | 0.26 | 0.21 | 0.14 | 0.30 | 2.00 | 0.35 |
| -72.04843413 | 19.64386456 | 0.03 | 0.03 | 0.06 | 0.05 | 0.24 | 0.25 | 0.13 | 0.30 | 2.00 | 0.54 |
| -72.21498179 | 19.69048712 | 0.03 | 0.04 | 0.07 | 0.06 | 0.33 | 0.21 | 0.10 | 0.30 | 2.00 | 0.19 |
| -73.78397926 | 18.17996997 | 0.02 | 0.03 | 0.06 | 0.05 | 0.26 | 0.14 | 0.07 | 0.30 | 2.00 | 0.13 |
| -72.20150706 | 18.52384506 | 0.04 | 0.05 | 0.08 | 0.08 | 0.26 | 0.21 | 0.13 | 0.30 | 2.00 | 0.38 |
| -72.52220561 | 19.15015048 | 0.04 | 0.05 | 0.08 | 0.08 | 0.30 | 0.23 | 0.14 | 0.30 | 2.00 | 0.01 |
| -72.57960796 | 19.16820662 | 0.03 | 0.03 | 0.07 | 0.06 | 0.24 | 0.14 | 0.07 | 0.30 | 2.00 | 0.00 |
| -72.57879948 | 19.16820662 | 0.02 | 0.03 | 0.07 | 0.06 | 0.28 | 0.13 | 0.07 | 0.30 | 2.00 | 0.26 |
| -72.5874233 | 18.53839777 | 0.03 | 0.04 | 0.08 | 0.07 | 0.30 | 0.22 | 0.11 | 0.30 | 2.00 | 0.26 |
| -72.21471229 | 19.68940914 | 0.03 | 0.03 | 0.07 | 0.06 | 0.31 | 0.21 | 0.10 | 0.30 | 2.00 | 0.03 |
| -72.50630543 | 19.15446239 | 0.02 | 0.02 | 0.05 | 0.05 | 0.18 | 0.11 | 0.05 | 0.30 | 2.00 | 0.16 |
| -72.58930976 | 18.5370503 | 0.04 | 0.05 | 0.09 | 0.08 | 0.30 | 0.23 | 0.12 | 0.30 | 2.00 | 0.03 |
| -73.77481644 | 18.18401239 | 0.02 | 0.03 | 0.07 | 0.05 | 0.33 | 0.18 | 0.09 | 0.30 | 2.00 | 0.40 |
| -72.14653016 | 19.7265994 | 0.03 | 0.04 | 0.07 | 0.06 | 0.33 | 0.21 | 0.11 | 0.30 | 2.00 | 0.69 |
| -72.60143702 | 19.19704254 | 0.05 | 0.06 | 0.09 | 0.09 | 0.27 | 0.24 | 0.14 | 0.30 | 2.00 | 0.33 |
| -72.58904027 | 19.16039127 | 0.03 | 0.04 | 0.08 | 0.07 | 0.24 | 0.16 | 0.08 | 0.30 | 2.00 | 0.49 |
| -72.56316879 | 19.16443369 | 0.03 | 0.04 | 0.08 | 0.07 | 0.27 | 0.18 | 0.09 | 0.30 | 2.00 | 0.61 |
| -72.60278449 | 19.19623405 | 0.04 | 0.05 | 0.08 | 0.07 | 0.26 | 0.19 | 0.10 | 0.30 | 2.00 | 0.69 |
| -72.20204605 | 18.52438405 | 0.04 | 0.05 | 0.08 | 0.09 | 0.24 | 0.22 | 0.15 | 0.30 | 2.00 | 0.56 |
| -72.0500511 | 19.6419781 | 0.03 | 0.03 | 0.06 | 0.06 | 0.27 | 0.25 | 0.13 | 0.30 | 2.00 | 0.31 |
| -72.50684442 | 19.16739813 | 0.03 | 0.03 | 0.07 | 0.05 | 0.28 | 0.17 | 0.09 | 0.30 | 2.00 | 0.20 |
| -72.56155182 | 19.16362521 | 0.03 | 0.04 | 0.08 | 0.07 | 0.27 | 0.17 | 0.08 | 0.30 | 2.00 | 0.23 |
| -72.50630543 | 19.16739813 | 0.02 | 0.03 | 0.06 | 0.05 | 0.22 | 0.14 | 0.06 | 0.30 | 2.00 | 0.25 |
| -72.0500511 | 19.64332557 | 0.02 | 0.03 | 0.06 | 0.06 | 0.27 | 0.25 | 0.13 | 0.30 | 2.00 | 0.23 |
| -72.04816464 | 19.64305608 | 0.02 | 0.03 | 0.06 | 0.07 | 0.26 | 0.25 | 0.14 | 0.30 | 2.00 | 0.13 |
| -72.05032059 | 19.64548153 | 0.03 | 0.04 | 0.07 | 0.07 | 0.29 | 0.26 | 0.14 | 0.30 | 2.00 | 0.38 |
| -73.77373847 | 18.20287701 | 0.02 | 0.03 | 0.07 | 0.06 | 0.27 | 0.17 | 0.08 | 0.30 | 2.00 | 0.11 |
| -72.58095543 | 18.48584633 | 0.04 | 0.04 | 0.07 | 0.06 | 0.30 | 0.21 | 0.12 | 0.30 | 2.00 | 0.34 |
| -73.77346897 | 18.20260752 | 0.02 | 0.03 | 0.06 | 0.06 | 0.25 | 0.16 | 0.08 | 0.30 | 2.00 | 0.17 |
| -72.19530868 | 18.59903405 | 0.04 | 0.06 | 0.09 | 0.10 | 0.26 | 0.22 | 0.15 | 0.30 | 2.00 | 0.21 |
| -72.05220706 | 19.64224759 | 0.03 | 0.04 | 0.07 | 0.08 | 0.25 | 0.29 | 0.17 | 0.30 | 2.00 | 0.58 |
| -72.20123756 | 18.52519254 | 0.04 | 0.05 | 0.08 | 0.08 | 0.22 | 0.20 | 0.13 | 0.30 | 2.00 | 0.25 |
| -72.60278449 | 19.15473189 | 0.02 | 0.03 | 0.07 | 0.06 | 0.24 | 0.13 | 0.06 | 0.30 | 2.00 | 0.31 |
| -72.52193612 | 19.14961149 | 0.04 | 0.04 | 0.08 | 0.08 | 0.27 | 0.20 | 0.11 | 0.30 | 2.00 | 0.40 |
| -72.58203341 | 18.48611582 | 0.04 | 0.04 | 0.07 | 0.06 | 0.31 | 0.22 | 0.12 | 0.30 | 2.00 | 0.59 |
| -72.57987745 | 19.16605066 | 0.03 | 0.04 | 0.08 | 0.07 | 0.29 | 0.17 | 0.08 | 0.30 | 2.00 | 0.63 |
| -72.21390381 | 19.69129561 | 0.03 | 0.04 | 0.08 | 0.07 | 0.33 | 0.26 | 0.13 | 0.30 | 2.00 | 0.64 |
| -72.05193756 | 19.64575102 | 0.03 | 0.04 | 0.06 | 0.07 | 0.28 | 0.26 | 0.14 | 0.30 | 2.00 | 0.20 |
| -72.58176392 | 18.48584633 | 0.04 | 0.04 | 0.07 | 0.06 | 0.29 | 0.21 | 0.12 | 0.30 | 2.00 | 0.33 |
| -72.20150706 | 18.52438405 | 0.04 | 0.05 | 0.08 | 0.08 | 0.26 | 0.22 | 0.14 | 0.30 | 2.00 | 0.27 |
| -72.58904027 | 18.53758929 | 0.04 | 0.05 | 0.08 | 0.07 | 0.30 | 0.22 | 0.12 | 0.30 | 2.00 | 0.39 |
| -72.19989009 | 18.54351817 | 0.04 | 0.05 | 0.09 | 0.09 | 0.27 | 0.21 | 0.13 | 0.30 | 2.00 | 0.09 |
| -72.66449875 | 18.46805968 | 0.02 | 0.04 | 0.08 | 0.06 | 0.35 | 0.22 | 0.10 | 0.30 | 2.00 | 0.62 |
| -72.05085958 | 19.6417086 | 0.03 | 0.04 | 0.07 | 0.07 | 0.27 | 0.26 | 0.15 | 0.30 | 2.00 | 0.61 |
| -72.05112908 | 19.64386456 | 0.02 | 0.03 | 0.06 | 0.06 | 0.27 | 0.25 | 0.13 | 0.30 | 2.00 | 0.42 |
| -72.21686825 | 19.68913965 | 0.03 | 0.05 | 0.08 | 0.08 | 0.35 | 0.28 | 0.15 | 0.30 | 2.00 | 0.41 |
| -72.21713774 | 19.69021763 | 0.02 | 0.03 | 0.07 | 0.05 | 0.34 | 0.21 | 0.09 | 0.30 | 2.00 | 0.52 |
| -72.66638522 | 18.46886817 | 0.03 | 0.04 | 0.07 | 0.07 | 0.32 | 0.23 | 0.11 | 0.30 | 2.00 | 0.19 |
| -72.5874233 | 18.53812828 | 0.03 | 0.04 | 0.08 | 0.07 | 0.30 | 0.22 | 0.11 | 0.30 | 2.00 | 0.22 |
| -72.57852998 | 19.16847611 | 0.02 | 0.03 | 0.07 | 0.06 | 0.22 | 0.12 | 0.06 | 0.30 | 2.00 | 0.37 |
| -72.56289929 | 19.16497268 | 0.02 | 0.03 | 0.06 | 0.06 | 0.22 | 0.11 | 0.06 | 0.30 | 2.00 | 0.27 |
| -72.52355309 | 19.15015048 | 0.04 | 0.05 | 0.08 | 0.08 | 0.33 | 0.21 | 0.10 | 0.30 | 2.00 | 0.04 |
| -72.50684442 | 19.15473189 | 0.02 | 0.03 | 0.06 | 0.05 | 0.21 | 0.11 | 0.05 | 0.30 | 2.00 | 0.34 |
| -72.53379388 | 18.79738207 | 0.07 | 0.08 | 0.12 | 0.14 | 0.32 | 0.34 | 0.23 | 0.30 | 2.00 | 0.67 |
| -72.58688431 | 19.15554037 | 0.02 | 0.03 | 0.07 | 0.05 | 0.27 | 0.14 | 0.07 | 0.30 | 2.00 | 0.61 |
| -72.20258503 | 18.54432665 | 0.04 | 0.05 | 0.09 | 0.08 | 0.27 | 0.20 | 0.13 | 0.30 | 2.00 | 0.15 |
| -72.47666103 | 19.14287413 | 0.03 | 0.04 | 0.07 | 0.06 | 0.27 | 0.18 | 0.10 | 0.30 | 2.00 | 0.27 |
| -72.5626298 | 19.1638947 | 0.03 | 0.04 | 0.08 | 0.07 | 0.25 | 0.17 | 0.07 | 0.30 | 2.00 | 0.50 |
| -72.19207475 | 18.59903405 | 0.05 | 0.06 | 0.09 | 0.10 | 0.27 | 0.22 | 0.15 | 0.30 | 2.00 | 0.07 |
| -72.20123756 | 18.54405716 | 0.04 | 0.05 | 0.09 | 0.09 | 0.28 | 0.21 | 0.13 | 0.30 | 2.00 | 0.69 |
| -72.58607583 | 19.15527088 | 0.03 | 0.03 | 0.08 | 0.06 | 0.25 | 0.16 | 0.08 | 0.30 | 2.00 | 0.13 |
| -72.21498179 | 19.68860066 | 0.03 | 0.03 | 0.08 | 0.05 | 0.34 | 0.22 | 0.10 | 0.30 | 2.00 | 0.48 |
| -72.05193756 | 19.64305608 | 0.03 | 0.04 | 0.07 | 0.06 | 0.26 | 0.25 | 0.15 | 0.30 | 2.00 | 0.57 |
| -72.57879948 | 19.16847611 | 0.02 | 0.03 | 0.07 | 0.06 | 0.25 | 0.14 | 0.07 | 0.30 | 2.00 | 0.27 |
| -72.05193756 | 19.64494254 | 0.02 | 0.04 | 0.07 | 0.07 | 0.26 | 0.27 | 0.15 | 0.30 | 2.00 | 0.30 |
| -72.52166662 | 19.15015048 | 0.04 | 0.05 | 0.09 | 0.08 | 0.30 | 0.22 | 0.13 | 0.30 | 2.00 | 0.35 |
| -72.20177655 | 18.52411456 | 0.04 | 0.05 | 0.08 | 0.08 | 0.25 | 0.22 | 0.14 | 0.30 | 2.00 | 0.17 |
| -72.19396121 | 18.59957304 | 0.05 | 0.05 | 0.09 | 0.09 | 0.25 | 0.22 | 0.15 | 0.30 | 2.00 | 0.11 |
| -72.04816464 | 19.64440355 | 0.03 | 0.04 | 0.07 | 0.07 | 0.24 | 0.25 | 0.14 | 0.30 | 2.00 | 0.10 |
| -72.05220706 | 19.64278658 | 0.03 | 0.04 | 0.07 | 0.07 | 0.27 | 0.28 | 0.16 | 0.30 | 2.00 | 0.52 |
| -72.52247511 | 19.15015048 | 0.04 | 0.05 | 0.08 | 0.07 | 0.31 | 0.23 | 0.12 | 0.30 | 2.00 | 0.22 |
| -72.19503919 | 18.59930355 | 0.04 | 0.05 | 0.09 | 0.09 | 0.27 | 0.22 | 0.13 | 0.30 | 2.00 | 0.59 |
| -72.19638666 | 18.53731979 | 0.04 | 0.06 | 0.09 | 0.09 | 0.33 | 0.25 | 0.15 | 0.30 | 2.00 | 0.10 |
| -72.1945002 | 18.53758929 | 0.05 | 0.06 | 0.09 | 0.10 | 0.31 | 0.26 | 0.17 | 0.30 | 2.00 | 0.64 |
| -72.56209081 | 19.1638947 | 0.03 | 0.04 | 0.08 | 0.07 | 0.25 | 0.17 | 0.08 | 0.30 | 2.00 | 0.07 |
| -72.53352439 | 18.79846005 | 0.07 | 0.08 | 0.11 | 0.12 | 0.32 | 0.32 | 0.20 | 0.30 | 2.00 | 0.34 |
| -72.47746951 | 19.14152665 | 0.03 | 0.04 | 0.07 | 0.07 | 0.25 | 0.18 | 0.10 | 0.30 | 2.00 | 0.14 |
| -72.57879948 | 19.1692846 | 0.02 | 0.03 | 0.07 | 0.06 | 0.26 | 0.13 | 0.07 | 0.30 | 2.00 | 0.11 |
| -72.602515 | 19.19569506 | 0.04 | 0.06 | 0.09 | 0.10 | 0.25 | 0.19 | 0.11 | 0.30 | 2.00 | 0.12 |
| -72.21686825 | 19.68994813 | 0.02 | 0.03 | 0.07 | 0.05 | 0.31 | 0.22 | 0.10 | 0.30 | 2.00 | 0.33 |
| -72.20204605 | 18.52573153 | 0.04 | 0.05 | 0.08 | 0.08 | 0.22 | 0.21 | 0.15 | 0.30 | 2.00 | 0.21 |
| -72.04816464 | 19.64251709 | 0.02 | 0.03 | 0.06 | 0.06 | 0.28 | 0.25 | 0.14 | 0.30 | 2.00 | 0.08 |
| -72.15353702 | 19.72067052 | 0.04 | 0.05 | 0.08 | 0.09 | 0.31 | 0.26 | 0.15 | 0.30 | 2.00 | 0.39 |
| -72.53325489 | 18.79819055 | 0.07 | 0.08 | 0.12 | 0.12 | 0.32 | 0.32 | 0.20 | 0.30 | 2.00 | 0.43 |
| -72.20096807 | 18.54486564 | 0.04 | 0.05 | 0.09 | 0.09 | 0.27 | 0.22 | 0.14 | 0.30 | 2.00 | 0.52 |
| -72.57987745 | 19.16820662 | 0.03 | 0.04 | 0.07 | 0.07 | 0.25 | 0.16 | 0.07 | 0.30 | 2.00 | 0.43 |
| -72.05247655 | 19.64278658 | 0.03 | 0.05 | 0.07 | 0.09 | 0.24 | 0.31 | 0.19 | 0.30 | 2.00 | 0.32 |
| -72.04870363 | 19.64143911 | 0.02 | 0.04 | 0.07 | 0.07 | 0.27 | 0.26 | 0.14 | 0.30 | 2.00 | 0.42 |
| -72.20204605 | 18.52546203 | 0.05 | 0.06 | 0.08 | 0.09 | 0.22 | 0.21 | 0.15 | 0.30 | 2.00 | 0.57 |
| -72.15111157 | 19.70962124 | 0.04 | 0.05 | 0.07 | 0.08 | 0.18 | 0.26 | 0.19 | 0.30 | 2.00 | 0.22 |
| -72.60278449 | 19.15500138 | 0.02 | 0.03 | 0.07 | 0.06 | 0.26 | 0.14 | 0.08 | 0.30 | 2.00 | 0.27 |
| -72.21579027 | 19.69129561 | 0.02 | 0.03 | 0.07 | 0.05 | 0.35 | 0.20 | 0.09 | 0.30 | 2.00 | 0.56 |
| -72.05032059 | 19.64278658 | 0.03 | 0.04 | 0.07 | 0.08 | 0.26 | 0.26 | 0.15 | 0.30 | 2.00 | 0.66 |
| -72.58203341 | 18.4871938 | 0.04 | 0.04 | 0.07 | 0.07 | 0.30 | 0.21 | 0.12 | 0.30 | 2.00 | 0.45 |
| -72.57933846 | 19.16632015 | 0.03 | 0.03 | 0.08 | 0.06 | 0.27 | 0.17 | 0.08 | 0.30 | 2.00 | 0.03 |
| -72.57879948 | 19.16793712 | 0.02 | 0.03 | 0.07 | 0.06 | 0.25 | 0.13 | 0.06 | 0.30 | 2.00 | 0.55 |
| -72.58877078 | 18.5370503 | 0.04 | 0.05 | 0.08 | 0.07 | 0.31 | 0.22 | 0.11 | 0.30 | 2.00 | 0.02 |
| -72.58607583 | 19.1541929 | 0.02 | 0.03 | 0.07 | 0.05 | 0.30 | 0.16 | 0.08 | 0.30 | 2.00 | 0.53 |
| -72.5329854 | 18.79819055 | 0.06 | 0.07 | 0.11 | 0.11 | 0.32 | 0.30 | 0.18 | 0.30 | 2.00 | 0.13 |
| -72.19369171 | 18.59957304 | 0.04 | 0.05 | 0.08 | 0.08 | 0.26 | 0.22 | 0.14 | 0.30 | 2.00 | 0.68 |
| -72.20177655 | 18.52546203 | 0.04 | 0.05 | 0.08 | 0.08 | 0.22 | 0.20 | 0.14 | 0.30 | 2.00 | 0.57 |
| -72.58930976 | 18.53570283 | 0.04 | 0.04 | 0.08 | 0.08 | 0.31 | 0.23 | 0.14 | 0.30 | 2.00 | 0.60 |
| -72.4831289 | 19.09813802 | 0.03 | 0.04 | 0.08 | 0.06 | 0.29 | 0.19 | 0.10 | 0.30 | 2.00 | 0.02 |
| -72.2144428 | 19.6918346 | 0.03 | 0.03 | 0.07 | 0.06 | 0.32 | 0.21 | 0.11 | 0.30 | 2.00 | 0.47 |
| -72.66557673 | 18.46913766 | 0.03 | 0.05 | 0.08 | 0.08 | 0.32 | 0.27 | 0.14 | 0.30 | 2.00 | 0.45 |
| -72.6022455 | 19.15500138 | 0.03 | 0.04 | 0.08 | 0.07 | 0.27 | 0.19 | 0.11 | 0.30 | 2.00 | 0.52 |
| -72.52166662 | 19.1490725 | 0.04 | 0.04 | 0.08 | 0.07 | 0.32 | 0.20 | 0.11 | 0.30 | 2.00 | 0.06 |
| -72.04951211 | 19.64251709 | 0.03 | 0.04 | 0.07 | 0.07 | 0.27 | 0.25 | 0.14 | 0.30 | 2.00 | 0.45 |
| -72.58149442 | 18.4871938 | 0.04 | 0.04 | 0.07 | 0.07 | 0.29 | 0.21 | 0.12 | 0.30 | 2.00 | 0.64 |
| -72.58634532 | 19.15527088 | 0.03 | 0.03 | 0.08 | 0.06 | 0.24 | 0.15 | 0.08 | 0.30 | 2.00 | 0.46 |
| -72.5876928 | 18.5367808 | 0.03 | 0.04 | 0.08 | 0.07 | 0.32 | 0.23 | 0.12 | 0.30 | 2.00 | 0.27 |
| -72.20042908 | 18.52600102 | 0.04 | 0.05 | 0.08 | 0.07 | 0.27 | 0.20 | 0.12 | 0.30 | 2.00 | 0.33 |
| -72.52220561 | 19.1490725 | 0.04 | 0.05 | 0.08 | 0.07 | 0.29 | 0.22 | 0.12 | 0.30 | 2.00 | 0.18 |
| -72.20285453 | 18.52546203 | 0.04 | 0.05 | 0.08 | 0.08 | 0.25 | 0.21 | 0.14 | 0.30 | 2.00 | 0.23 |
| -72.04843413 | 19.64143911 | 0.02 | 0.03 | 0.07 | 0.07 | 0.27 | 0.26 | 0.14 | 0.30 | 2.00 | 0.35 |
| -72.04789514 | 19.64413405 | 0.03 | 0.04 | 0.07 | 0.07 | 0.25 | 0.26 | 0.14 | 0.30 | 2.00 | 0.70 |
| -72.5076529 | 19.15527088 | 0.02 | 0.03 | 0.05 | 0.06 | 0.21 | 0.14 | 0.06 | 0.30 | 2.00 | 0.22 |
| -72.50603594 | 19.15607936 | 0.02 | 0.03 | 0.05 | 0.06 | 0.19 | 0.12 | 0.06 | 0.30 | 2.00 | 0.21 |
| -72.60170652 | 19.19650355 | 0.04 | 0.05 | 0.09 | 0.08 | 0.28 | 0.20 | 0.12 | 0.30 | 2.00 | 0.51 |
| -72.04897312 | 19.64359507 | 0.03 | 0.04 | 0.07 | 0.08 | 0.25 | 0.26 | 0.15 | 0.30 | 2.00 | 0.11 |
| -72.21632926 | 19.6918346 | 0.02 | 0.03 | 0.07 | 0.05 | 0.35 | 0.20 | 0.09 | 0.30 | 2.00 | 0.10 |
| -72.56936717 | 19.16820662 | 0.03 | 0.04 | 0.08 | 0.07 | 0.28 | 0.18 | 0.09 | 0.30 | 2.00 | 0.38 |
| -72.19207475 | 18.59957304 | 0.04 | 0.06 | 0.09 | 0.09 | 0.28 | 0.22 | 0.14 | 0.30 | 2.00 | 0.38 |
| -72.05112908 | 19.64494254 | 0.02 | 0.03 | 0.06 | 0.07 | 0.27 | 0.25 | 0.14 | 0.30 | 2.00 | 0.31 |
| -72.57933846 | 19.16847611 | 0.03 | 0.04 | 0.07 | 0.06 | 0.25 | 0.15 | 0.07 | 0.30 | 2.00 | 0.30 |
| -72.56370778 | 19.16362521 | 0.03 | 0.04 | 0.08 | 0.08 | 0.25 | 0.16 | 0.08 | 0.30 | 2.00 | 0.38 |
| -72.5079224 | 19.15500138 | 0.02 | 0.02 | 0.06 | 0.05 | 0.20 | 0.13 | 0.06 | 0.30 | 2.00 | 0.08 |
| -72.1945002 | 18.59903405 | 0.04 | 0.05 | 0.09 | 0.09 | 0.25 | 0.23 | 0.14 | 0.30 | 2.00 | 0.63 |
| -72.19611716 | 18.53812828 | 0.04 | 0.06 | 0.09 | 0.09 | 0.30 | 0.25 | 0.15 | 0.30 | 2.00 | 0.56 |
| -72.21498179 | 19.6918346 | 0.02 | 0.03 | 0.07 | 0.06 | 0.32 | 0.21 | 0.10 | 0.30 | 2.00 | 0.15 |
| -72.5076529 | 19.15634886 | 0.02 | 0.03 | 0.06 | 0.05 | 0.22 | 0.12 | 0.06 | 0.30 | 2.00 | 0.08 |
| -72.20231554 | 18.52438405 | 0.04 | 0.05 | 0.08 | 0.08 | 0.26 | 0.21 | 0.14 | 0.30 | 2.00 | 0.13 |
| -72.20150706 | 18.54405716 | 0.04 | 0.06 | 0.09 | 0.09 | 0.28 | 0.21 | 0.14 | 0.30 | 2.00 | 0.45 |
| -72.48339839 | 19.09840752 | 0.03 | 0.04 | 0.07 | 0.07 | 0.27 | 0.18 | 0.10 | 0.30 | 2.00 | 0.31 |
| -72.58796229 | 18.53785878 | 0.03 | 0.04 | 0.08 | 0.07 | 0.28 | 0.22 | 0.12 | 0.30 | 2.00 | 0.53 |
| -72.66773269 | 18.46832918 | 0.03 | 0.04 | 0.07 | 0.07 | 0.29 | 0.19 | 0.11 | 0.30 | 2.00 | 0.45 |
| -72.58284189 | 18.48638532 | 0.04 | 0.04 | 0.07 | 0.06 | 0.29 | 0.21 | 0.12 | 0.30 | 2.00 | 0.08 |
| -72.2141733 | 19.69075662 | 0.04 | 0.05 | 0.08 | 0.08 | 0.25 | 0.26 | 0.18 | 0.30 | 2.00 | 0.33 |
| -72.19989009 | 18.54351817 | 0.04 | 0.05 | 0.09 | 0.09 | 0.27 | 0.21 | 0.13 | 0.30 | 2.00 | 0.21 |
| -72.05166807 | 19.64251709 | 0.03 | 0.04 | 0.07 | 0.08 | 0.23 | 0.28 | 0.16 | 0.30 | 2.00 | 0.02 |
| -72.2144428 | 19.69102611 | 0.03 | 0.04 | 0.08 | 0.06 | 0.28 | 0.24 | 0.13 | 0.30 | 2.00 | 0.06 |
| -72.52220561 | 19.14988098 | 0.04 | 0.05 | 0.08 | 0.08 | 0.31 | 0.22 | 0.13 | 0.30 | 2.00 | 0.09 |
| -72.56209081 | 19.16497268 | 0.02 | 0.03 | 0.06 | 0.05 | 0.21 | 0.12 | 0.06 | 0.30 | 2.00 | 0.15 |
| -72.56209081 | 19.1638947 | 0.03 | 0.04 | 0.08 | 0.07 | 0.25 | 0.17 | 0.08 | 0.30 | 2.00 | 0.16 |
| -72.21498179 | 19.69102611 | 0.02 | 0.04 | 0.07 | 0.06 | 0.34 | 0.20 | 0.09 | 0.30 | 2.00 | 0.52 |
| -72.14706915 | 19.72740788 | 0.04 | 0.04 | 0.08 | 0.07 | 0.34 | 0.23 | 0.13 | 0.30 | 2.00 | 0.05 |
| -72.63781879 | 18.48153441 | 0.02 | 0.04 | 0.07 | 0.06 | 0.38 | 0.20 | 0.09 | 0.30 | 2.00 | 0.63 |
| -72.57879948 | 19.16632015 | 0.03 | 0.04 | 0.07 | 0.06 | 0.24 | 0.15 | 0.07 | 0.30 | 2.00 | 0.68 |
| -72.5874233 | 19.15500138 | 0.02 | 0.03 | 0.06 | 0.05 | 0.26 | 0.14 | 0.07 | 0.30 | 2.00 | 0.68 |
| -73.51178973 | 18.51953315 | 0.01 | 0.02 | 0.06 | 0.03 | 0.40 | 0.15 | 0.06 | 0.30 | 3.00 | 0.55 |
| -72.60601843 | 18.52977394 | 0.03 | 0.03 | 0.06 | 0.04 | 0.30 | 0.16 | 0.08 | 0.30 | 3.00 | 0.14 |
| -72.58526735 | 18.49932106 | 0.02 | 0.03 | 0.06 | 0.04 | 0.31 | 0.15 | 0.08 | 0.30 | 3.00 | 0.53 |
| -72.70465345 | 19.92629489 | 0.01 | 0.02 | 0.06 | 0.04 | 0.35 | 0.16 | 0.07 | 0.30 | 3.00 | 0.10 |
| -72.6073659 | 18.5319299 | 0.02 | 0.02 | 0.05 | 0.03 | 0.33 | 0.13 | 0.05 | 0.30 | 3.00 | 0.69 |
| -72.60682691 | 18.5321994 | 0.02 | 0.03 | 0.06 | 0.05 | 0.31 | 0.17 | 0.08 | 0.30 | 3.00 | 0.38 |
| -73.47810291 | 18.25650643 | 0.01 | 0.02 | 0.06 | 0.03 | 0.40 | 0.12 | 0.04 | 0.30 | 3.00 | 0.45 |
| -72.6173372 | 18.52141961 | 0.02 | 0.03 | 0.06 | 0.04 | 0.29 | 0.16 | 0.08 | 0.30 | 3.00 | 0.12 |
| -72.63404587 | 19.89772846 | 0.01 | 0.02 | 0.05 | 0.03 | 0.36 | 0.14 | 0.06 | 0.30 | 3.00 | 0.23 |
| -72.21040038 | 18.54648261 | 0.05 | 0.06 | 0.10 | 0.09 | 0.33 | 0.28 | 0.15 | 0.30 | 3.00 | 0.64 |
| -72.20527998 | 18.54513514 | 0.03 | 0.04 | 0.09 | 0.07 | 0.34 | 0.22 | 0.12 | 0.30 | 3.00 | 0.59 |
| -73.34685904 | 18.26917268 | 0.01 | 0.02 | 0.06 | 0.04 | 0.33 | 0.14 | 0.06 | 0.30 | 3.00 | 0.32 |
| -72.07295814 | 19.64575102 | 0.03 | 0.04 | 0.07 | 0.08 | 0.28 | 0.29 | 0.17 | 0.30 | 3.00 | 0.47 |
| -73.54439857 | 18.51737719 | 0.00 | 0.01 | 0.05 | 0.03 | 0.45 | 0.11 | 0.04 | 0.30 | 3.00 | 0.04 |
| -72.58499785 | 18.49905156 | 0.02 | 0.03 | 0.06 | 0.04 | 0.33 | 0.15 | 0.07 | 0.30 | 3.00 | 0.43 |
| -72.29502168 | 18.58987124 | 0.28 | 0.31 | 0.34 | 0.34 | 0.38 | 0.53 | 0.51 | 0.30 | 3.00 | 0.11 |
| -72.70438395 | 19.92602539 | 0.01 | 0.02 | 0.06 | 0.04 | 0.37 | 0.16 | 0.07 | 0.30 | 3.00 | 0.30 |
| -73.42070056 | 18.25704542 | 0.02 | 0.02 | 0.06 | 0.04 | 0.40 | 0.16 | 0.07 | 0.30 | 3.00 | 0.06 |
| -72.70411446 | 19.92656438 | 0.02 | 0.04 | 0.08 | 0.07 | 0.31 | 0.18 | 0.11 | 0.30 | 3.00 | 0.27 |
| -72.20554947 | 18.54459615 | 0.03 | 0.04 | 0.08 | 0.06 | 0.35 | 0.19 | 0.10 | 0.30 | 3.00 | 0.56 |
| -72.65614442 | 19.90958622 | 0.03 | 0.03 | 0.08 | 0.06 | 0.35 | 0.22 | 0.11 | 0.30 | 3.00 | 0.66 |
| -73.41477168 | 18.27159813 | 0.01 | 0.02 | 0.05 | 0.03 | 0.38 | 0.12 | 0.05 | 0.30 | 3.00 | 0.35 |
| -72.27373161 | 18.60819687 | 0.02 | 0.03 | 0.06 | 0.03 | 0.34 | 0.14 | 0.06 | 0.30 | 3.00 | 0.40 |
| -72.20608846 | 18.5467521 | 0.03 | 0.04 | 0.09 | 0.07 | 0.35 | 0.22 | 0.11 | 0.30 | 3.00 | 0.57 |
| -72.57314009 | 18.49770409 | 0.02 | 0.03 | 0.06 | 0.04 | 0.34 | 0.16 | 0.08 | 0.30 | 3.00 | 0.37 |
| -72.07322763 | 19.64548153 | 0.03 | 0.04 | 0.07 | 0.08 | 0.28 | 0.28 | 0.16 | 0.30 | 3.00 | 0.25 |
| -73.51367619 | 18.51953315 | 0.01 | 0.01 | 0.06 | 0.03 | 0.41 | 0.13 | 0.05 | 0.30 | 3.00 | 0.32 |
| -72.20042908 | 18.53866727 | 0.02 | 0.03 | 0.06 | 0.05 | 0.29 | 0.16 | 0.08 | 0.30 | 3.00 | 0.16 |
| -73.51286771 | 18.51980264 | 0.01 | 0.01 | 0.05 | 0.02 | 0.42 | 0.13 | 0.04 | 0.30 | 3.00 | 0.22 |
| -72.60601843 | 18.53004344 | 0.02 | 0.03 | 0.05 | 0.04 | 0.31 | 0.16 | 0.08 | 0.30 | 3.00 | 0.41 |
| -72.60467096 | 18.52896546 | 0.02 | 0.02 | 0.05 | 0.03 | 0.32 | 0.14 | 0.06 | 0.30 | 3.00 | 0.12 |
| -72.20608846 | 18.54567412 | 0.05 | 0.06 | 0.10 | 0.09 | 0.35 | 0.27 | 0.15 | 0.30 | 3.00 | 0.02 |
| -73.47810291 | 18.25650643 | 0.01 | 0.02 | 0.06 | 0.03 | 0.40 | 0.12 | 0.04 | 0.30 | 3.00 | 0.13 |
| -72.20797493 | 18.53327737 | 0.02 | 0.03 | 0.07 | 0.05 | 0.33 | 0.17 | 0.08 | 0.30 | 3.00 | 0.70 |
| -72.20878341 | 18.54783008 | 0.05 | 0.06 | 0.10 | 0.10 | 0.32 | 0.29 | 0.16 | 0.30 | 3.00 | 0.37 |
| -72.20824442 | 18.53327737 | 0.02 | 0.03 | 0.07 | 0.05 | 0.33 | 0.17 | 0.09 | 0.30 | 3.00 | 0.21 |
| -72.20959189 | 18.54729109 | 0.05 | 0.07 | 0.10 | 0.10 | 0.32 | 0.31 | 0.18 | 0.30 | 3.00 | 0.07 |
| -72.20878341 | 18.54863857 | 0.05 | 0.06 | 0.10 | 0.10 | 0.34 | 0.29 | 0.17 | 0.30 | 3.00 | 0.28 |
| -73.51178973 | 18.51980264 | 0.01 | 0.02 | 0.05 | 0.03 | 0.41 | 0.14 | 0.05 | 0.30 | 3.00 | 0.31 |
| -72.20878341 | 18.54809958 | 0.05 | 0.06 | 0.10 | 0.09 | 0.32 | 0.27 | 0.15 | 0.30 | 3.00 | 0.39 |
| -72.20527998 | 18.54729109 | 0.03 | 0.04 | 0.08 | 0.05 | 0.36 | 0.19 | 0.09 | 0.30 | 3.00 | 0.57 |
| -72.6073659 | 18.53166041 | 0.02 | 0.02 | 0.05 | 0.03 | 0.33 | 0.15 | 0.06 | 0.30 | 3.00 | 0.44 |
| -72.27346211 | 18.60792737 | 0.02 | 0.03 | 0.06 | 0.03 | 0.33 | 0.14 | 0.05 | 0.30 | 3.00 | 0.53 |
| -72.07268864 | 19.64548153 | 0.03 | 0.04 | 0.07 | 0.08 | 0.28 | 0.30 | 0.17 | 0.30 | 3.00 | 0.24 |
| -72.77768648 | 19.9403086 | 0.03 | 0.03 | 0.07 | 0.06 | 0.33 | 0.21 | 0.11 | 0.30 | 3.00 | 0.36 |
| -72.20581897 | 18.5467521 | 0.03 | 0.04 | 0.09 | 0.06 | 0.35 | 0.21 | 0.11 | 0.30 | 3.00 | 0.24 |
| -72.20689695 | 18.53408586 | 0.02 | 0.03 | 0.07 | 0.05 | 0.35 | 0.16 | 0.07 | 0.30 | 3.00 | 0.43 |
| -72.2093224 | 18.54783008 | 0.05 | 0.06 | 0.10 | 0.11 | 0.32 | 0.30 | 0.17 | 0.30 | 3.00 | 0.16 |
| -72.20824442 | 18.53300788 | 0.02 | 0.03 | 0.07 | 0.05 | 0.34 | 0.17 | 0.08 | 0.30 | 3.00 | 0.25 |
| -72.60467096 | 18.53004344 | 0.03 | 0.03 | 0.06 | 0.05 | 0.31 | 0.19 | 0.09 | 0.30 | 3.00 | 0.20 |
| -72.07295814 | 19.6419781 | 0.03 | 0.04 | 0.08 | 0.07 | 0.32 | 0.29 | 0.16 | 0.30 | 3.00 | 0.17 |
| -72.20770543 | 18.53435535 | 0.02 | 0.03 | 0.06 | 0.04 | 0.34 | 0.15 | 0.07 | 0.30 | 3.00 | 0.09 |
| -72.20851392 | 18.54729109 | 0.05 | 0.07 | 0.10 | 0.11 | 0.33 | 0.31 | 0.18 | 0.30 | 3.00 | 0.63 |
| -72.60494045 | 18.52896546 | 0.02 | 0.02 | 0.05 | 0.03 | 0.33 | 0.15 | 0.06 | 0.30 | 3.00 | 0.24 |
| -73.51259821 | 18.52061113 | 0.01 | 0.02 | 0.06 | 0.03 | 0.44 | 0.14 | 0.05 | 0.30 | 3.00 | 0.13 |
| -72.61679821 | 18.52115012 | 0.02 | 0.03 | 0.06 | 0.04 | 0.31 | 0.15 | 0.07 | 0.30 | 3.00 | 0.69 |
| -72.58445886 | 18.49851257 | 0.02 | 0.02 | 0.05 | 0.03 | 0.32 | 0.15 | 0.06 | 0.30 | 3.00 | 0.09 |
| -72.2740011 | 18.60846636 | 0.03 | 0.03 | 0.06 | 0.04 | 0.33 | 0.14 | 0.06 | 0.30 | 3.00 | 0.31 |
| -72.07322763 | 19.64305608 | 0.03 | 0.04 | 0.07 | 0.07 | 0.31 | 0.28 | 0.16 | 0.30 | 3.00 | 0.39 |
| -72.20662745 | 18.54594362 | 0.03 | 0.03 | 0.08 | 0.06 | 0.35 | 0.19 | 0.09 | 0.30 | 3.00 | 0.20 |
| -72.20015958 | 18.53920625 | 0.04 | 0.05 | 0.08 | 0.08 | 0.28 | 0.22 | 0.13 | 0.30 | 3.00 | 0.45 |
| -72.20069857 | 18.53839777 | 0.02 | 0.03 | 0.06 | 0.05 | 0.30 | 0.16 | 0.08 | 0.30 | 3.00 | 0.16 |
| -72.6073659 | 18.53246889 | 0.02 | 0.02 | 0.05 | 0.03 | 0.33 | 0.13 | 0.05 | 0.30 | 3.00 | 0.40 |
| -72.20554947 | 18.5467521 | 0.03 | 0.04 | 0.08 | 0.06 | 0.35 | 0.20 | 0.10 | 0.30 | 3.00 | 0.67 |
| -72.2090529 | 18.53435535 | 0.02 | 0.03 | 0.06 | 0.04 | 0.33 | 0.15 | 0.07 | 0.30 | 3.00 | 0.26 |
| -73.54412908 | 18.5171077 | 0.01 | 0.01 | 0.06 | 0.04 | 0.45 | 0.13 | 0.04 | 0.30 | 3.00 | 0.37 |
| -72.20716644 | 18.53462485 | 0.02 | 0.03 | 0.06 | 0.04 | 0.35 | 0.14 | 0.06 | 0.30 | 3.00 | 0.36 |
| -72.20716644 | 18.53435535 | 0.02 | 0.02 | 0.06 | 0.04 | 0.36 | 0.14 | 0.06 | 0.30 | 3.00 | 0.07 |
| -72.57314009 | 18.49770409 | 0.02 | 0.03 | 0.06 | 0.04 | 0.34 | 0.16 | 0.08 | 0.30 | 3.00 | 0.03 |
| -72.60547944 | 18.53300788 | 0.03 | 0.03 | 0.06 | 0.04 | 0.33 | 0.17 | 0.08 | 0.30 | 3.00 | 0.67 |
| -72.2093224 | 18.5470216 | 0.05 | 0.06 | 0.10 | 0.10 | 0.33 | 0.30 | 0.18 | 0.30 | 3.00 | 0.12 |
| -72.20824442 | 18.54783008 | 0.05 | 0.06 | 0.10 | 0.10 | 0.34 | 0.30 | 0.17 | 0.30 | 3.00 | 0.17 |
| -72.60628792 | 18.52977394 | 0.03 | 0.03 | 0.06 | 0.05 | 0.27 | 0.18 | 0.10 | 0.30 | 3.00 | 0.01 |
| -72.2090529 | 18.54783008 | 0.05 | 0.06 | 0.10 | 0.11 | 0.31 | 0.30 | 0.17 | 0.30 | 3.00 | 0.14 |
| -72.60494045 | 18.52923496 | 0.02 | 0.02 | 0.05 | 0.03 | 0.32 | 0.14 | 0.06 | 0.30 | 3.00 | 0.50 |
| -72.60574893 | 18.53112142 | 0.02 | 0.02 | 0.05 | 0.03 | 0.34 | 0.14 | 0.06 | 0.30 | 3.00 | 0.02 |
| -72.20554947 | 18.5470216 | 0.03 | 0.04 | 0.09 | 0.07 | 0.35 | 0.23 | 0.12 | 0.30 | 3.00 | 0.38 |
| -72.552928 | 18.52249759 | 0.02 | 0.03 | 0.06 | 0.04 | 0.35 | 0.18 | 0.08 | 0.30 | 3.00 | 0.58 |
| -72.20554947 | 18.54486564 | 0.03 | 0.04 | 0.08 | 0.05 | 0.34 | 0.18 | 0.09 | 0.30 | 3.00 | 0.50 |
| -72.60628792 | 18.52923496 | 0.03 | 0.03 | 0.06 | 0.05 | 0.28 | 0.17 | 0.09 | 0.30 | 3.00 | 0.08 |
| -73.54385959 | 18.51629922 | 0.01 | 0.02 | 0.05 | 0.03 | 0.38 | 0.12 | 0.05 | 0.30 | 3.00 | 0.12 |
| -72.58445886 | 18.49905156 | 0.02 | 0.02 | 0.06 | 0.03 | 0.33 | 0.16 | 0.08 | 0.30 | 3.00 | 0.05 |
| -72.20069857 | 18.53866727 | 0.02 | 0.03 | 0.06 | 0.04 | 0.30 | 0.15 | 0.07 | 0.30 | 3.00 | 0.29 |
| -72.66045633 | 19.45144543 | 0.04 | 0.04 | 0.08 | 0.06 | 0.36 | 0.19 | 0.10 | 0.30 | 3.00 | 0.31 |
| -72.20554947 | 18.54486564 | 0.03 | 0.04 | 0.08 | 0.05 | 0.34 | 0.18 | 0.09 | 0.30 | 3.00 | 0.61 |
| -72.07295814 | 19.64278658 | 0.03 | 0.04 | 0.08 | 0.08 | 0.31 | 0.29 | 0.17 | 0.30 | 3.00 | 0.12 |
| -72.72971644 | 19.93168478 | 0.02 | 0.03 | 0.08 | 0.06 | 0.32 | 0.21 | 0.12 | 0.30 | 3.00 | 0.34 |
| -72.20662745 | 18.5470216 | 0.03 | 0.04 | 0.08 | 0.06 | 0.36 | 0.20 | 0.09 | 0.30 | 3.00 | 0.38 |
| -72.77795597 | 19.94003911 | 0.02 | 0.03 | 0.06 | 0.05 | 0.32 | 0.19 | 0.09 | 0.30 | 3.00 | 0.22 |
| -72.72998594 | 19.93168478 | 0.02 | 0.04 | 0.08 | 0.07 | 0.32 | 0.20 | 0.11 | 0.30 | 3.00 | 0.41 |
| -72.07241915 | 19.64467304 | 0.04 | 0.05 | 0.08 | 0.09 | 0.29 | 0.32 | 0.18 | 0.30 | 3.00 | 0.00 |
| -72.07295814 | 19.6419781 | 0.03 | 0.04 | 0.08 | 0.07 | 0.32 | 0.29 | 0.16 | 0.30 | 3.00 | 0.29 |
| -72.6073659 | 18.5319299 | 0.02 | 0.02 | 0.05 | 0.03 | 0.33 | 0.13 | 0.05 | 0.30 | 3.00 | 0.26 |
| -72.20770543 | 18.53435535 | 0.02 | 0.03 | 0.06 | 0.04 | 0.34 | 0.15 | 0.07 | 0.30 | 3.00 | 0.58 |
| -73.54439857 | 18.5171077 | 0.01 | 0.01 | 0.06 | 0.03 | 0.48 | 0.12 | 0.04 | 0.30 | 3.00 | 0.69 |
| -72.66045633 | 19.45090644 | 0.03 | 0.04 | 0.07 | 0.06 | 0.38 | 0.18 | 0.09 | 0.30 | 3.00 | 0.12 |
| -72.20527998 | 18.5467521 | 0.03 | 0.04 | 0.08 | 0.05 | 0.35 | 0.19 | 0.09 | 0.30 | 3.00 | 0.10 |
| -72.20770543 | 18.53381636 | 0.02 | 0.03 | 0.06 | 0.04 | 0.34 | 0.14 | 0.06 | 0.30 | 3.00 | 0.25 |
| -72.72971644 | 19.93168478 | 0.02 | 0.03 | 0.08 | 0.06 | 0.32 | 0.21 | 0.12 | 0.30 | 3.00 | 0.15 |
| -72.60547944 | 18.5319299 | 0.02 | 0.02 | 0.06 | 0.03 | 0.34 | 0.14 | 0.05 | 0.30 | 3.00 | 0.11 |
| -73.54439857 | 18.5168382 | 0.01 | 0.02 | 0.06 | 0.03 | 0.46 | 0.14 | 0.05 | 0.30 | 3.00 | 0.30 |
| -72.07322763 | 19.64494254 | 0.03 | 0.04 | 0.08 | 0.08 | 0.29 | 0.30 | 0.17 | 0.30 | 3.00 | 0.38 |
| -72.20770543 | 18.53408586 | 0.02 | 0.02 | 0.06 | 0.03 | 0.33 | 0.14 | 0.06 | 0.30 | 3.00 | 0.36 |
| -72.21040038 | 18.54890806 | 0.03 | 0.04 | 0.08 | 0.06 | 0.37 | 0.21 | 0.10 | 0.30 | 3.00 | 0.47 |
| -72.58365038 | 18.49851257 | 0.02 | 0.02 | 0.05 | 0.04 | 0.29 | 0.14 | 0.07 | 0.30 | 3.00 | 0.57 |
| -72.20042908 | 18.53974524 | 0.02 | 0.03 | 0.06 | 0.04 | 0.34 | 0.16 | 0.08 | 0.30 | 3.00 | 0.32 |
| -73.54493756 | 18.51737719 | 0.01 | 0.01 | 0.05 | 0.03 | 0.40 | 0.12 | 0.04 | 0.30 | 3.00 | 0.30 |
| -72.60682691 | 18.53273838 | 0.02 | 0.02 | 0.05 | 0.03 | 0.33 | 0.15 | 0.06 | 0.30 | 3.00 | 0.01 |
| -72.20824442 | 18.53300788 | 0.02 | 0.03 | 0.07 | 0.05 | 0.34 | 0.17 | 0.08 | 0.30 | 3.00 | 0.06 |
| -72.20797493 | 18.53354687 | 0.02 | 0.03 | 0.07 | 0.05 | 0.33 | 0.16 | 0.08 | 0.30 | 3.00 | 0.27 |
| -72.60709641 | 18.53058243 | 0.02 | 0.03 | 0.05 | 0.04 | 0.34 | 0.15 | 0.06 | 0.30 | 3.00 | 0.60 |
| -72.60520994 | 18.52896546 | 0.02 | 0.02 | 0.05 | 0.03 | 0.34 | 0.14 | 0.05 | 0.30 | 3.00 | 0.52 |
| -73.41450218 | 18.27159813 | 0.01 | 0.02 | 0.06 | 0.03 | 0.39 | 0.14 | 0.06 | 0.30 | 3.00 | 0.66 |
| -72.20554947 | 18.54621311 | 0.03 | 0.04 | 0.08 | 0.06 | 0.36 | 0.20 | 0.10 | 0.30 | 3.00 | 0.25 |
| -72.2093224 | 18.54836907 | 0.05 | 0.06 | 0.11 | 0.10 | 0.34 | 0.28 | 0.16 | 0.30 | 3.00 | 0.43 |
| -72.20797493 | 18.53408586 | 0.03 | 0.03 | 0.07 | 0.05 | 0.35 | 0.16 | 0.08 | 0.30 | 3.00 | 0.23 |
| -72.07268864 | 19.64521203 | 0.03 | 0.04 | 0.08 | 0.09 | 0.28 | 0.30 | 0.17 | 0.30 | 3.00 | 0.25 |
| -72.20716644 | 18.56049633 | 0.03 | 0.04 | 0.07 | 0.06 | 0.34 | 0.20 | 0.10 | 0.30 | 3.00 | 0.58 |
| -72.07322763 | 19.64575102 | 0.03 | 0.04 | 0.07 | 0.08 | 0.28 | 0.29 | 0.16 | 0.30 | 3.00 | 0.61 |
| -72.57421807 | 18.49824308 | 0.02 | 0.02 | 0.06 | 0.04 | 0.33 | 0.15 | 0.07 | 0.30 | 3.00 | 0.13 |
| -72.77822547 | 19.9403086 | 0.02 | 0.03 | 0.06 | 0.05 | 0.34 | 0.19 | 0.09 | 0.30 | 3.00 | 0.22 |
| -72.07268864 | 19.64467304 | 0.03 | 0.04 | 0.08 | 0.09 | 0.29 | 0.30 | 0.17 | 0.30 | 3.00 | 0.51 |
| -73.42043107 | 18.25704542 | 0.01 | 0.02 | 0.06 | 0.04 | 0.39 | 0.15 | 0.07 | 0.30 | 3.00 | 0.69 |
| -73.51152024 | 18.52007214 | 0.01 | 0.02 | 0.05 | 0.03 | 0.41 | 0.14 | 0.05 | 0.30 | 3.00 | 0.40 |
| -72.20797493 | 18.54729109 | 0.04 | 0.05 | 0.09 | 0.08 | 0.33 | 0.24 | 0.13 | 0.30 | 3.00 | 0.11 |
| -72.20851392 | 18.53354687 | 0.02 | 0.03 | 0.07 | 0.04 | 0.33 | 0.16 | 0.07 | 0.30 | 3.00 | 0.69 |
| -72.20527998 | 18.54621311 | 0.03 | 0.04 | 0.08 | 0.06 | 0.36 | 0.20 | 0.10 | 0.30 | 3.00 | 0.39 |
| -72.6073659 | 18.53246889 | 0.02 | 0.02 | 0.05 | 0.03 | 0.33 | 0.13 | 0.05 | 0.30 | 3.00 | 0.43 |
| -72.20474099 | 18.54594362 | 0.03 | 0.04 | 0.09 | 0.06 | 0.35 | 0.22 | 0.11 | 0.30 | 3.00 | 0.32 |
| -72.60682691 | 18.53085192 | 0.02 | 0.03 | 0.06 | 0.04 | 0.33 | 0.16 | 0.07 | 0.30 | 3.00 | 0.39 |
| -72.20770543 | 18.56076582 | 0.03 | 0.04 | 0.08 | 0.06 | 0.36 | 0.20 | 0.10 | 0.30 | 3.00 | 0.48 |
| -72.60547944 | 18.5321994 | 0.02 | 0.03 | 0.06 | 0.04 | 0.33 | 0.15 | 0.06 | 0.30 | 3.00 | 0.29 |
| -72.60709641 | 18.5319299 | 0.02 | 0.02 | 0.05 | 0.03 | 0.33 | 0.14 | 0.06 | 0.30 | 3.00 | 0.14 |
| -72.60628792 | 18.53139091 | 0.02 | 0.02 | 0.05 | 0.03 | 0.33 | 0.14 | 0.06 | 0.30 | 3.00 | 0.55 |
| -72.20581897 | 18.54486564 | 0.03 | 0.04 | 0.08 | 0.06 | 0.34 | 0.19 | 0.10 | 0.30 | 3.00 | 0.18 |
| -72.20608846 | 18.54648261 | 0.03 | 0.04 | 0.08 | 0.06 | 0.35 | 0.19 | 0.09 | 0.30 | 3.00 | 0.12 |
| -72.60547944 | 18.52950445 | 0.02 | 0.02 | 0.05 | 0.04 | 0.32 | 0.16 | 0.08 | 0.30 | 3.00 | 0.48 |
| -73.45088395 | 18.27105914 | 0.02 | 0.02 | 0.07 | 0.05 | 0.36 | 0.15 | 0.07 | 0.30 | 3.00 | 0.35 |
| -72.07295814 | 19.64467304 | 0.03 | 0.04 | 0.08 | 0.09 | 0.29 | 0.29 | 0.17 | 0.30 | 3.00 | 0.15 |
| -72.20689695 | 18.56049633 | 0.03 | 0.03 | 0.07 | 0.05 | 0.36 | 0.17 | 0.08 | 0.30 | 3.00 | 0.60 |
| -73.54493756 | 18.51656871 | 0.01 | 0.02 | 0.05 | 0.03 | 0.40 | 0.13 | 0.05 | 0.30 | 3.00 | 0.07 |
| -72.20797493 | 18.53327737 | 0.02 | 0.03 | 0.07 | 0.05 | 0.33 | 0.17 | 0.08 | 0.30 | 3.00 | 0.52 |
| -73.5134067 | 18.51980264 | 0.01 | 0.01 | 0.06 | 0.03 | 0.44 | 0.12 | 0.04 | 0.30 | 3.00 | 0.19 |
| -73.47810291 | 18.25650643 | 0.01 | 0.02 | 0.06 | 0.03 | 0.40 | 0.12 | 0.04 | 0.30 | 3.00 | 0.47 |
| -72.60547944 | 18.53300788 | 0.03 | 0.03 | 0.06 | 0.04 | 0.33 | 0.17 | 0.08 | 0.30 | 3.00 | 0.24 |
| -72.20554947 | 18.54594362 | 0.04 | 0.05 | 0.10 | 0.09 | 0.36 | 0.26 | 0.14 | 0.30 | 3.00 | 0.25 |
| -72.77795597 | 19.9405781 | 0.02 | 0.03 | 0.06 | 0.05 | 0.32 | 0.20 | 0.10 | 0.30 | 3.00 | 0.46 |
| -72.20824442 | 18.54756059 | 0.05 | 0.06 | 0.10 | 0.10 | 0.34 | 0.31 | 0.18 | 0.30 | 3.00 | 0.57 |
| -72.6073659 | 18.53273838 | 0.02 | 0.02 | 0.06 | 0.03 | 0.34 | 0.14 | 0.06 | 0.30 | 3.00 | 0.64 |
| -72.20474099 | 18.54513514 | 0.03 | 0.04 | 0.09 | 0.07 | 0.34 | 0.21 | 0.11 | 0.30 | 3.00 | 0.12 |
| -73.51178973 | 18.51953315 | 0.01 | 0.02 | 0.06 | 0.03 | 0.40 | 0.15 | 0.06 | 0.30 | 3.00 | 0.34 |
| -73.51178973 | 18.52061113 | 0.01 | 0.02 | 0.06 | 0.03 | 0.44 | 0.13 | 0.04 | 0.30 | 3.00 | 0.65 |
| -72.20554947 | 18.54540463 | 0.04 | 0.05 | 0.10 | 0.08 | 0.35 | 0.25 | 0.14 | 0.30 | 3.00 | 0.17 |
| -72.60628792 | 18.53246889 | 0.02 | 0.03 | 0.06 | 0.04 | 0.33 | 0.17 | 0.08 | 0.30 | 3.00 | 0.05 |
| -73.50532186 | 18.25030806 | 0.01 | 0.02 | 0.05 | 0.03 | 0.40 | 0.13 | 0.05 | 0.30 | 3.00 | 0.39 |
| -72.07214966 | 19.6419781 | 0.03 | 0.04 | 0.08 | 0.08 | 0.30 | 0.29 | 0.17 | 0.30 | 3.00 | 0.26 |
| -72.29448269 | 18.58960174 | 0.20 | 0.25 | 0.31 | 0.32 | 0.37 | 0.46 | 0.44 | 0.30 | 3.00 | 0.47 |
| -72.55238901 | 18.5222281 | 0.02 | 0.03 | 0.06 | 0.05 | 0.30 | 0.18 | 0.09 | 0.30 | 3.00 | 0.01 |
| -72.20824442 | 18.54729109 | 0.05 | 0.06 | 0.10 | 0.10 | 0.33 | 0.29 | 0.17 | 0.30 | 3.00 | 0.69 |
| -72.20797493 | 18.53435535 | 0.02 | 0.02 | 0.06 | 0.03 | 0.35 | 0.14 | 0.06 | 0.30 | 3.00 | 0.01 |
| -73.42177854 | 18.25731492 | 0.01 | 0.02 | 0.05 | 0.03 | 0.37 | 0.13 | 0.05 | 0.30 | 3.00 | 0.33 |
| -72.60601843 | 18.53139091 | 0.02 | 0.02 | 0.05 | 0.03 | 0.35 | 0.14 | 0.06 | 0.30 | 3.00 | 0.18 |
| -72.20797493 | 18.56103532 | 0.03 | 0.04 | 0.08 | 0.07 | 0.35 | 0.20 | 0.11 | 0.30 | 3.00 | 0.06 |
| -72.70492294 | 19.92602539 | 0.02 | 0.03 | 0.06 | 0.04 | 0.32 | 0.16 | 0.08 | 0.30 | 3.00 | 0.35 |
| -72.20527998 | 18.54540463 | 0.04 | 0.05 | 0.10 | 0.08 | 0.34 | 0.25 | 0.13 | 0.30 | 3.00 | 0.16 |
| -72.60628792 | 18.53246889 | 0.02 | 0.03 | 0.06 | 0.04 | 0.33 | 0.17 | 0.08 | 0.30 | 3.00 | 0.32 |
| -72.20662745 | 18.5470216 | 0.03 | 0.04 | 0.08 | 0.06 | 0.36 | 0.20 | 0.09 | 0.30 | 3.00 | 0.65 |
| -73.51152024 | 18.52061113 | 0.01 | 0.02 | 0.06 | 0.04 | 0.44 | 0.16 | 0.06 | 0.30 | 3.00 | 0.14 |
| -72.07241915 | 19.64440355 | 0.03 | 0.04 | 0.08 | 0.09 | 0.29 | 0.32 | 0.18 | 0.30 | 3.00 | 0.26 |
| -72.77795597 | 19.9403086 | 0.02 | 0.03 | 0.06 | 0.05 | 0.33 | 0.20 | 0.11 | 0.30 | 3.00 | 0.40 |
| -72.20501049 | 18.54648261 | 0.03 | 0.04 | 0.08 | 0.05 | 0.36 | 0.18 | 0.09 | 0.30 | 3.00 | 0.12 |
| -72.21013088 | 18.54756059 | 0.04 | 0.05 | 0.10 | 0.08 | 0.33 | 0.24 | 0.13 | 0.30 | 3.00 | 0.38 |
| -73.42097005 | 18.25704542 | 0.01 | 0.02 | 0.06 | 0.03 | 0.40 | 0.15 | 0.06 | 0.30 | 3.00 | 0.13 |
| -72.72890796 | 19.93168478 | 0.03 | 0.04 | 0.08 | 0.08 | 0.31 | 0.24 | 0.14 | 0.30 | 3.00 | 0.04 |
| -72.20069857 | 18.53893676 | 0.02 | 0.03 | 0.06 | 0.04 | 0.28 | 0.15 | 0.08 | 0.30 | 3.00 | 0.33 |
| -72.07295814 | 19.64332557 | 0.03 | 0.04 | 0.06 | 0.07 | 0.31 | 0.26 | 0.15 | 0.30 | 3.00 | 0.09 |
| -72.20851392 | 18.54890806 | 0.03 | 0.04 | 0.09 | 0.06 | 0.37 | 0.20 | 0.10 | 0.30 | 3.00 | 0.38 |
| -72.57340958 | 18.49797358 | 0.01 | 0.02 | 0.05 | 0.03 | 0.33 | 0.13 | 0.05 | 0.30 | 3.00 | 0.29 |
| -72.58445886 | 18.49851257 | 0.02 | 0.02 | 0.05 | 0.03 | 0.32 | 0.15 | 0.06 | 0.30 | 3.00 | 0.67 |
| -72.20986139 | 18.54809958 | 0.05 | 0.06 | 0.10 | 0.09 | 0.34 | 0.29 | 0.16 | 0.30 | 3.00 | 0.50 |
| -73.54439857 | 18.51764669 | 0.02 | 0.03 | 0.07 | 0.05 | 0.38 | 0.14 | 0.05 | 0.30 | 3.00 | 0.15 |
| -72.58445886 | 18.49905156 | 0.02 | 0.02 | 0.06 | 0.03 | 0.33 | 0.16 | 0.08 | 0.30 | 3.00 | 0.43 |
| -72.07268864 | 19.64521203 | 0.03 | 0.04 | 0.08 | 0.09 | 0.28 | 0.30 | 0.17 | 0.30 | 3.00 | 0.16 |
| -72.60628792 | 18.52950445 | 0.03 | 0.03 | 0.05 | 0.04 | 0.28 | 0.14 | 0.07 | 0.30 | 3.00 | 0.12 |
| -72.60574893 | 18.52950445 | 0.02 | 0.03 | 0.05 | 0.04 | 0.32 | 0.15 | 0.07 | 0.30 | 3.00 | 0.09 |
| -72.60520994 | 18.53273838 | 0.02 | 0.03 | 0.05 | 0.03 | 0.32 | 0.14 | 0.06 | 0.30 | 3.00 | 0.22 |
| -73.51205922 | 18.51926366 | 0.01 | 0.02 | 0.05 | 0.03 | 0.39 | 0.14 | 0.06 | 0.30 | 3.00 | 0.60 |
| -72.20554947 | 18.54486564 | 0.03 | 0.04 | 0.08 | 0.05 | 0.34 | 0.18 | 0.09 | 0.30 | 3.00 | 0.34 |
| -73.47729442 | 18.25704542 | 0.01 | 0.03 | 0.06 | 0.05 | 0.35 | 0.14 | 0.06 | 0.30 | 3.00 | 0.31 |
| -72.60817439 | 18.53273838 | 0.02 | 0.03 | 0.05 | 0.03 | 0.33 | 0.16 | 0.07 | 0.30 | 3.00 | 0.12 |
| -72.60655742 | 18.53139091 | 0.02 | 0.03 | 0.05 | 0.04 | 0.31 | 0.15 | 0.07 | 0.30 | 3.00 | 0.70 |
| -72.60494045 | 18.52950445 | 0.02 | 0.02 | 0.05 | 0.03 | 0.32 | 0.15 | 0.06 | 0.30 | 3.00 | 0.26 |
| -72.20635796 | 18.54621311 | 0.03 | 0.03 | 0.08 | 0.05 | 0.36 | 0.18 | 0.08 | 0.30 | 3.00 | 0.17 |
| -72.20878341 | 18.54836907 | 0.05 | 0.07 | 0.10 | 0.10 | 0.33 | 0.30 | 0.17 | 0.30 | 3.00 | 0.08 |
| -72.61787619 | 18.52141961 | 0.03 | 0.04 | 0.06 | 0.05 | 0.31 | 0.15 | 0.08 | 0.30 | 3.00 | 0.48 |
| -72.20581897 | 18.54621311 | 0.03 | 0.04 | 0.09 | 0.06 | 0.35 | 0.22 | 0.11 | 0.30 | 3.00 | 0.66 |
| -73.51286771 | 18.51980264 | 0.01 | 0.01 | 0.05 | 0.02 | 0.42 | 0.13 | 0.04 | 0.30 | 3.00 | 0.48 |
| -73.42150904 | 18.25731492 | 0.01 | 0.02 | 0.05 | 0.03 | 0.39 | 0.13 | 0.05 | 0.30 | 3.00 | 0.29 |
| -73.5131372 | 18.51953315 | 0.01 | 0.02 | 0.06 | 0.03 | 0.45 | 0.13 | 0.04 | 0.30 | 3.00 | 0.09 |
| -72.20096807 | 18.53812828 | 0.02 | 0.03 | 0.06 | 0.05 | 0.29 | 0.15 | 0.08 | 0.30 | 3.00 | 0.20 |
| -72.61518124 | 18.5222281 | 0.02 | 0.02 | 0.06 | 0.03 | 0.33 | 0.14 | 0.06 | 0.30 | 3.00 | 0.57 |
| -73.51205922 | 18.52034163 | 0.01 | 0.01 | 0.05 | 0.03 | 0.42 | 0.12 | 0.04 | 0.30 | 3.00 | 0.47 |
| -72.20689695 | 18.53408586 | 0.02 | 0.03 | 0.07 | 0.05 | 0.35 | 0.16 | 0.07 | 0.30 | 3.00 | 0.25 |
| -72.20716644 | 18.56103532 | 0.03 | 0.03 | 0.07 | 0.05 | 0.35 | 0.19 | 0.10 | 0.30 | 3.00 | 0.60 |
| -73.54385959 | 18.5168382 | 0.01 | 0.02 | 0.05 | 0.03 | 0.35 | 0.16 | 0.07 | 0.30 | 3.00 | 0.69 |
| -72.07241915 | 19.64440355 | 0.03 | 0.04 | 0.08 | 0.09 | 0.29 | 0.32 | 0.18 | 0.30 | 3.00 | 0.15 |
| -72.20527998 | 18.54648261 | 0.03 | 0.04 | 0.08 | 0.05 | 0.36 | 0.19 | 0.09 | 0.30 | 3.00 | 0.56 |
| -72.72944695 | 19.93141528 | 0.02 | 0.03 | 0.07 | 0.05 | 0.33 | 0.19 | 0.09 | 0.30 | 3.00 | 0.03 |
| -72.60817439 | 18.53273838 | 0.02 | 0.03 | 0.05 | 0.03 | 0.33 | 0.16 | 0.07 | 0.30 | 3.00 | 0.61 |
| -72.6073659 | 18.5321994 | 0.02 | 0.02 | 0.05 | 0.03 | 0.33 | 0.14 | 0.06 | 0.30 | 3.00 | 0.51 |
| -72.60547944 | 18.53004344 | 0.02 | 0.02 | 0.05 | 0.03 | 0.33 | 0.16 | 0.07 | 0.30 | 3.00 | 0.36 |
| -72.58365038 | 18.49824308 | 0.01 | 0.02 | 0.05 | 0.03 | 0.32 | 0.14 | 0.06 | 0.30 | 3.00 | 0.40 |
| -72.21013088 | 18.54836907 | 0.03 | 0.04 | 0.08 | 0.06 | 0.38 | 0.20 | 0.09 | 0.30 | 3.00 | 0.36 |
| -72.77795597 | 19.94003911 | 0.02 | 0.03 | 0.06 | 0.05 | 0.32 | 0.19 | 0.09 | 0.30 | 3.00 | 0.39 |
| -72.07295814 | 19.64224759 | 0.03 | 0.04 | 0.08 | 0.08 | 0.31 | 0.29 | 0.16 | 0.30 | 3.00 | 0.45 |
| -72.07295814 | 19.64278658 | 0.03 | 0.04 | 0.08 | 0.08 | 0.31 | 0.29 | 0.17 | 0.30 | 3.00 | 0.01 |
| -72.20851392 | 18.54917755 | 0.03 | 0.03 | 0.08 | 0.05 | 0.38 | 0.17 | 0.07 | 0.30 | 3.00 | 0.16 |
| -72.60601843 | 18.52950445 | 0.02 | 0.03 | 0.05 | 0.04 | 0.31 | 0.15 | 0.07 | 0.30 | 3.00 | 0.47 |
| -73.42016157 | 18.25758441 | 0.02 | 0.02 | 0.06 | 0.04 | 0.37 | 0.17 | 0.08 | 0.30 | 3.00 | 0.17 |
| -72.20042908 | 18.53839777 | 0.02 | 0.03 | 0.06 | 0.05 | 0.29 | 0.16 | 0.09 | 0.30 | 3.00 | 0.57 |
| -72.20474099 | 18.54540463 | 0.03 | 0.04 | 0.09 | 0.08 | 0.34 | 0.22 | 0.12 | 0.30 | 3.00 | 0.55 |
| -72.07214966 | 19.64548153 | 0.04 | 0.04 | 0.08 | 0.09 | 0.28 | 0.31 | 0.18 | 0.30 | 3.00 | 0.32 |
| -72.20824442 | 18.54756059 | 0.05 | 0.06 | 0.10 | 0.10 | 0.34 | 0.31 | 0.18 | 0.30 | 3.00 | 0.09 |
| -72.07241915 | 19.64548153 | 0.04 | 0.04 | 0.07 | 0.09 | 0.28 | 0.31 | 0.18 | 0.30 | 3.00 | 0.63 |
| -72.20608846 | 18.54513514 | 0.04 | 0.05 | 0.09 | 0.07 | 0.34 | 0.22 | 0.12 | 0.30 | 3.00 | 0.13 |
| -72.20662745 | 18.5467521 | 0.03 | 0.04 | 0.08 | 0.05 | 0.36 | 0.19 | 0.09 | 0.30 | 3.00 | 0.16 |
| -72.07241915 | 19.64224759 | 0.03 | 0.04 | 0.08 | 0.08 | 0.30 | 0.29 | 0.16 | 0.30 | 3.00 | 0.64 |
| -72.20635796 | 18.5470216 | 0.03 | 0.04 | 0.08 | 0.06 | 0.36 | 0.22 | 0.10 | 0.30 | 3.00 | 0.52 |
| -72.60547944 | 18.53058243 | 0.02 | 0.02 | 0.05 | 0.03 | 0.32 | 0.14 | 0.06 | 0.30 | 3.00 | 0.53 |
| -72.77822547 | 19.9403086 | 0.02 | 0.03 | 0.06 | 0.05 | 0.34 | 0.19 | 0.09 | 0.30 | 3.00 | 0.63 |
| -73.54466807 | 18.51737719 | 0.01 | 0.01 | 0.05 | 0.03 | 0.44 | 0.11 | 0.04 | 0.30 | 3.00 | 0.58 |
| -73.50559135 | 18.24976907 | 0.01 | 0.02 | 0.05 | 0.03 | 0.36 | 0.12 | 0.04 | 0.30 | 3.00 | 0.58 |
| -73.54412908 | 18.51602972 | 0.01 | 0.02 | 0.05 | 0.03 | 0.39 | 0.13 | 0.05 | 0.30 | 3.00 | 0.10 |
| -73.54439857 | 18.5171077 | 0.01 | 0.01 | 0.06 | 0.03 | 0.48 | 0.12 | 0.04 | 0.30 | 3.00 | 0.62 |
| -72.60574893 | 18.53058243 | 0.02 | 0.02 | 0.05 | 0.03 | 0.33 | 0.14 | 0.06 | 0.30 | 3.00 | 0.04 |
| -72.58472836 | 18.49824308 | 0.02 | 0.03 | 0.06 | 0.05 | 0.28 | 0.15 | 0.08 | 0.30 | 3.00 | 0.56 |
| -72.2093224 | 18.54756059 | 0.05 | 0.07 | 0.10 | 0.11 | 0.32 | 0.31 | 0.19 | 0.30 | 3.00 | 0.56 |
| -72.20716644 | 18.53354687 | 0.02 | 0.02 | 0.06 | 0.04 | 0.36 | 0.14 | 0.06 | 0.30 | 3.00 | 0.26 |
| -72.20878341 | 18.54729109 | 0.05 | 0.07 | 0.10 | 0.11 | 0.31 | 0.30 | 0.18 | 0.30 | 3.00 | 0.69 |
| -72.20797493 | 18.56103532 | 0.03 | 0.04 | 0.08 | 0.07 | 0.35 | 0.20 | 0.11 | 0.30 | 3.00 | 0.62 |
| -72.60655742 | 18.53058243 | 0.02 | 0.03 | 0.05 | 0.03 | 0.34 | 0.15 | 0.06 | 0.30 | 3.00 | 0.38 |
| -72.6073659 | 18.5321994 | 0.02 | 0.02 | 0.05 | 0.03 | 0.33 | 0.14 | 0.06 | 0.30 | 3.00 | 0.31 |
| -72.21093937 | 18.54890806 | 0.04 | 0.05 | 0.09 | 0.07 | 0.35 | 0.23 | 0.13 | 0.30 | 3.00 | 0.10 |
| -72.20069857 | 18.53947575 | 0.02 | 0.03 | 0.06 | 0.04 | 0.31 | 0.14 | 0.07 | 0.30 | 3.00 | 0.20 |
| -72.20554947 | 18.54540463 | 0.04 | 0.05 | 0.10 | 0.08 | 0.35 | 0.25 | 0.14 | 0.30 | 3.00 | 0.03 |
| -72.20069857 | 18.53812828 | 0.02 | 0.03 | 0.06 | 0.05 | 0.30 | 0.15 | 0.08 | 0.30 | 3.00 | 0.61 |
| -72.07322763 | 19.64278658 | 0.03 | 0.04 | 0.07 | 0.08 | 0.30 | 0.28 | 0.15 | 0.30 | 3.00 | 0.13 |
| -72.20042908 | 18.53785878 | 0.02 | 0.03 | 0.06 | 0.04 | 0.31 | 0.15 | 0.07 | 0.30 | 3.00 | 0.64 |
| -72.20716644 | 18.56130481 | 0.05 | 0.07 | 0.11 | 0.10 | 0.32 | 0.23 | 0.15 | 0.30 | 3.00 | 0.09 |
| -72.70438395 | 19.92629489 | 0.01 | 0.02 | 0.06 | 0.04 | 0.35 | 0.16 | 0.07 | 0.30 | 3.00 | 0.01 |
| -72.60547944 | 18.52923496 | 0.02 | 0.02 | 0.05 | 0.03 | 0.33 | 0.15 | 0.06 | 0.30 | 3.00 | 0.27 |
| -72.21040038 | 18.54863857 | 0.03 | 0.03 | 0.07 | 0.04 | 0.37 | 0.18 | 0.08 | 0.30 | 3.00 | 0.22 |
| -72.552928 | 18.5222281 | 0.03 | 0.03 | 0.07 | 0.06 | 0.35 | 0.19 | 0.10 | 0.30 | 3.00 | 0.44 |
| -72.21040038 | 18.54809958 | 0.04 | 0.05 | 0.10 | 0.08 | 0.36 | 0.24 | 0.13 | 0.30 | 3.00 | 0.63 |
| -72.20069857 | 18.53839777 | 0.02 | 0.03 | 0.06 | 0.05 | 0.30 | 0.16 | 0.08 | 0.30 | 3.00 | 0.61 |
| -72.60547944 | 18.5321994 | 0.02 | 0.03 | 0.06 | 0.04 | 0.33 | 0.15 | 0.06 | 0.30 | 3.00 | 0.49 |
| -72.57340958 | 18.49770409 | 0.01 | 0.02 | 0.05 | 0.03 | 0.34 | 0.15 | 0.06 | 0.30 | 3.00 | 0.30 |
| -72.58445886 | 18.49878207 | 0.02 | 0.02 | 0.05 | 0.03 | 0.31 | 0.15 | 0.07 | 0.30 | 3.00 | 0.66 |
| -72.6073659 | 18.5319299 | 0.02 | 0.02 | 0.05 | 0.03 | 0.33 | 0.13 | 0.05 | 0.30 | 3.00 | 0.21 |
| -72.07322763 | 19.64467304 | 0.03 | 0.04 | 0.08 | 0.09 | 0.29 | 0.29 | 0.17 | 0.30 | 3.00 | 0.02 |
| -72.2093224 | 18.54809958 | 0.05 | 0.06 | 0.10 | 0.10 | 0.32 | 0.30 | 0.17 | 0.30 | 3.00 | 0.01 |
| -72.58418937 | 18.49878207 | 0.02 | 0.02 | 0.05 | 0.03 | 0.31 | 0.14 | 0.06 | 0.30 | 3.00 | 0.54 |
| -72.60520994 | 18.53112142 | 0.03 | 0.03 | 0.06 | 0.05 | 0.31 | 0.19 | 0.09 | 0.30 | 3.00 | 0.55 |
| -73.41477168 | 18.27213712 | 0.02 | 0.02 | 0.05 | 0.04 | 0.33 | 0.16 | 0.07 | 0.30 | 3.00 | 0.44 |
| -72.20851392 | 18.54890806 | 0.03 | 0.04 | 0.09 | 0.06 | 0.37 | 0.20 | 0.10 | 0.30 | 3.00 | 0.44 |
| -72.07241915 | 19.64116961 | 0.03 | 0.04 | 0.08 | 0.08 | 0.31 | 0.28 | 0.15 | 0.30 | 3.00 | 0.09 |
| -72.72890796 | 19.93195427 | 0.03 | 0.04 | 0.08 | 0.07 | 0.30 | 0.21 | 0.12 | 0.30 | 3.00 | 0.16 |
| -72.61679821 | 18.52061113 | 0.02 | 0.02 | 0.05 | 0.04 | 0.31 | 0.14 | 0.07 | 0.30 | 3.00 | 0.11 |
| -73.54466807 | 18.51737719 | 0.01 | 0.01 | 0.05 | 0.03 | 0.44 | 0.11 | 0.04 | 0.30 | 3.00 | 0.13 |
| -72.20501049 | 18.54486564 | 0.03 | 0.03 | 0.08 | 0.05 | 0.35 | 0.18 | 0.09 | 0.30 | 3.00 | 0.06 |
| -72.60709641 | 18.53273838 | 0.03 | 0.03 | 0.06 | 0.05 | 0.32 | 0.17 | 0.08 | 0.30 | 3.00 | 0.51 |
| -73.42016157 | 18.25731492 | 0.01 | 0.02 | 0.06 | 0.03 | 0.40 | 0.16 | 0.07 | 0.30 | 3.00 | 0.58 |
| -72.60520994 | 18.52896546 | 0.02 | 0.02 | 0.05 | 0.03 | 0.34 | 0.14 | 0.05 | 0.30 | 3.00 | 0.45 |
| -72.21040038 | 18.54783008 | 0.05 | 0.06 | 0.10 | 0.10 | 0.33 | 0.29 | 0.17 | 0.30 | 3.00 | 0.69 |
| -72.57394857 | 18.49770409 | 0.02 | 0.03 | 0.06 | 0.04 | 0.33 | 0.15 | 0.07 | 0.30 | 3.00 | 0.68 |
| -73.50559135 | 18.24949958 | 0.01 | 0.02 | 0.05 | 0.03 | 0.38 | 0.12 | 0.04 | 0.30 | 3.00 | 0.50 |
| -73.54466807 | 18.51629922 | 0.01 | 0.02 | 0.05 | 0.03 | 0.39 | 0.13 | 0.05 | 0.30 | 3.00 | 0.47 |
| -72.6173372 | 18.52115012 | 0.02 | 0.03 | 0.06 | 0.04 | 0.32 | 0.15 | 0.07 | 0.30 | 3.00 | 0.23 |
| -72.20635796 | 18.54513514 | 0.04 | 0.05 | 0.09 | 0.08 | 0.35 | 0.24 | 0.13 | 0.30 | 3.00 | 0.29 |
| -72.07268864 | 19.6419781 | 0.03 | 0.04 | 0.07 | 0.07 | 0.31 | 0.28 | 0.16 | 0.30 | 3.00 | 0.25 |
| -73.42016157 | 18.25704542 | 0.01 | 0.02 | 0.06 | 0.04 | 0.40 | 0.15 | 0.06 | 0.30 | 3.00 | 0.23 |
| -72.58472836 | 18.49905156 | 0.01 | 0.02 | 0.05 | 0.03 | 0.34 | 0.14 | 0.06 | 0.30 | 3.00 | 0.37 |
| -72.70465345 | 19.92629489 | 0.01 | 0.02 | 0.06 | 0.04 | 0.35 | 0.16 | 0.07 | 0.30 | 3.00 | 0.23 |
| -72.77795597 | 19.9405781 | 0.02 | 0.03 | 0.06 | 0.05 | 0.32 | 0.20 | 0.10 | 0.30 | 3.00 | 0.20 |
| -72.20824442 | 18.53408586 | 0.02 | 0.03 | 0.07 | 0.04 | 0.35 | 0.15 | 0.07 | 0.30 | 3.00 | 0.61 |
| -72.60601843 | 18.53031293 | 0.02 | 0.02 | 0.05 | 0.03 | 0.33 | 0.16 | 0.07 | 0.30 | 3.00 | 0.50 |
| -72.60467096 | 18.52950445 | 0.02 | 0.03 | 0.06 | 0.04 | 0.32 | 0.16 | 0.07 | 0.30 | 3.00 | 0.20 |
| -72.60709641 | 18.53246889 | 0.02 | 0.03 | 0.06 | 0.04 | 0.34 | 0.17 | 0.08 | 0.30 | 3.00 | 0.18 |
| -72.61518124 | 18.52276709 | 0.02 | 0.03 | 0.07 | 0.05 | 0.33 | 0.16 | 0.08 | 0.30 | 3.00 | 0.00 |
| -73.42123955 | 18.25704542 | 0.01 | 0.02 | 0.07 | 0.03 | 0.43 | 0.15 | 0.06 | 0.30 | 3.00 | 0.42 |
| -72.20554947 | 18.54621311 | 0.03 | 0.04 | 0.08 | 0.06 | 0.36 | 0.20 | 0.10 | 0.30 | 3.00 | 0.03 |
| -72.2090529 | 18.53408586 | 0.02 | 0.03 | 0.06 | 0.04 | 0.33 | 0.15 | 0.07 | 0.30 | 3.00 | 0.63 |
| -72.61652872 | 18.52034163 | 0.02 | 0.02 | 0.05 | 0.04 | 0.31 | 0.14 | 0.07 | 0.30 | 3.00 | 0.15 |
| -73.34712854 | 18.26863369 | 0.01 | 0.02 | 0.05 | 0.04 | 0.34 | 0.13 | 0.06 | 0.30 | 3.00 | 0.11 |
| -72.77822547 | 19.9403086 | 0.02 | 0.03 | 0.06 | 0.05 | 0.34 | 0.19 | 0.09 | 0.30 | 3.00 | 0.11 |
| -73.51178973 | 18.52034163 | 0.01 | 0.02 | 0.05 | 0.03 | 0.43 | 0.14 | 0.05 | 0.30 | 3.00 | 0.61 |
| -72.20689695 | 18.56103532 | 0.03 | 0.03 | 0.07 | 0.05 | 0.38 | 0.18 | 0.09 | 0.30 | 3.00 | 0.30 |
| -73.50559135 | 18.24976907 | 0.01 | 0.02 | 0.05 | 0.03 | 0.36 | 0.12 | 0.04 | 0.30 | 3.00 | 0.62 |
| -73.51205922 | 18.52115012 | 0.01 | 0.02 | 0.06 | 0.03 | 0.42 | 0.13 | 0.04 | 0.30 | 3.00 | 0.10 |
| -72.60547944 | 18.53004344 | 0.02 | 0.02 | 0.05 | 0.03 | 0.33 | 0.16 | 0.07 | 0.30 | 3.00 | 0.52 |
| -72.29421319 | 18.58933225 | 0.07 | 0.08 | 0.10 | 0.10 | 0.26 | 0.18 | 0.13 | 0.30 | 3.00 | 0.02 |
| -72.60628792 | 18.53300788 | 0.03 | 0.03 | 0.07 | 0.05 | 0.34 | 0.19 | 0.09 | 0.30 | 3.00 | 0.01 |
| -72.07241915 | 19.64440355 | 0.03 | 0.04 | 0.08 | 0.09 | 0.29 | 0.32 | 0.18 | 0.30 | 3.00 | 0.37 |
| -72.20581897 | 18.54648261 | 0.03 | 0.04 | 0.07 | 0.05 | 0.36 | 0.18 | 0.09 | 0.30 | 3.00 | 0.51 |
| -73.51286771 | 18.51980264 | 0.01 | 0.01 | 0.05 | 0.02 | 0.42 | 0.13 | 0.04 | 0.30 | 3.00 | 0.10 |
| -72.60709641 | 18.53246889 | 0.02 | 0.03 | 0.06 | 0.04 | 0.34 | 0.17 | 0.08 | 0.30 | 3.00 | 0.03 |
| -72.63458485 | 19.89772846 | 0.03 | 0.04 | 0.06 | 0.05 | 0.31 | 0.16 | 0.09 | 0.30 | 3.00 | 0.40 |
| -73.41477168 | 18.27213712 | 0.02 | 0.02 | 0.05 | 0.04 | 0.33 | 0.16 | 0.07 | 0.30 | 3.00 | 0.40 |
| -72.66045633 | 19.45090644 | 0.03 | 0.04 | 0.07 | 0.06 | 0.38 | 0.18 | 0.09 | 0.30 | 3.00 | 0.27 |
| -72.20797493 | 18.54756059 | 0.04 | 0.05 | 0.10 | 0.08 | 0.34 | 0.26 | 0.14 | 0.30 | 3.00 | 0.26 |
| -72.60601843 | 18.53112142 | 0.02 | 0.02 | 0.05 | 0.03 | 0.33 | 0.15 | 0.07 | 0.30 | 3.00 | 0.11 |
| -72.20527998 | 18.54459615 | 0.04 | 0.04 | 0.09 | 0.07 | 0.34 | 0.20 | 0.11 | 0.30 | 3.00 | 0.39 |
| -72.20096807 | 18.53920625 | 0.02 | 0.03 | 0.06 | 0.04 | 0.29 | 0.15 | 0.08 | 0.30 | 3.00 | 0.27 |
| -72.07322763 | 19.64224759 | 0.03 | 0.04 | 0.07 | 0.07 | 0.30 | 0.27 | 0.15 | 0.30 | 3.00 | 0.30 |
| -72.20069857 | 18.53947575 | 0.02 | 0.03 | 0.06 | 0.04 | 0.31 | 0.14 | 0.07 | 0.30 | 3.00 | 0.06 |
| -72.07295814 | 19.64278658 | 0.03 | 0.04 | 0.08 | 0.08 | 0.31 | 0.29 | 0.17 | 0.30 | 3.00 | 0.38 |
| -72.20689695 | 18.56049633 | 0.03 | 0.03 | 0.07 | 0.05 | 0.36 | 0.17 | 0.08 | 0.30 | 3.00 | 0.03 |
| -72.20042908 | 18.53866727 | 0.02 | 0.03 | 0.06 | 0.05 | 0.29 | 0.16 | 0.08 | 0.30 | 3.00 | 0.07 |
| -72.58418937 | 18.49851257 | 0.02 | 0.02 | 0.06 | 0.03 | 0.34 | 0.15 | 0.06 | 0.30 | 3.00 | 0.65 |
| -72.20689695 | 18.5470216 | 0.02 | 0.03 | 0.08 | 0.05 | 0.37 | 0.19 | 0.09 | 0.30 | 3.00 | 0.53 |
| -72.20824442 | 18.53408586 | 0.02 | 0.03 | 0.07 | 0.04 | 0.35 | 0.15 | 0.07 | 0.30 | 3.00 | 0.04 |
| -72.20042908 | 18.53974524 | 0.02 | 0.03 | 0.06 | 0.04 | 0.34 | 0.16 | 0.08 | 0.30 | 3.00 | 0.54 |
| -72.07268864 | 19.64143911 | 0.03 | 0.04 | 0.07 | 0.08 | 0.30 | 0.27 | 0.16 | 0.30 | 3.00 | 0.14 |
| -73.47756392 | 18.25677593 | 0.01 | 0.02 | 0.06 | 0.04 | 0.35 | 0.12 | 0.05 | 0.30 | 3.00 | 0.55 |
| -72.66045633 | 19.45171492 | 0.04 | 0.04 | 0.08 | 0.06 | 0.36 | 0.20 | 0.11 | 0.30 | 3.00 | 0.14 |
| -72.2090529 | 18.54783008 | 0.05 | 0.06 | 0.10 | 0.11 | 0.31 | 0.30 | 0.17 | 0.30 | 3.00 | 0.69 |
| -72.20581897 | 18.54567412 | 0.05 | 0.06 | 0.10 | 0.10 | 0.35 | 0.30 | 0.17 | 0.30 | 3.00 | 0.36 |
| -72.20608846 | 18.54486564 | 0.03 | 0.04 | 0.09 | 0.07 | 0.33 | 0.21 | 0.11 | 0.30 | 3.00 | 0.05 |
| -72.20851392 | 18.53408586 | 0.02 | 0.03 | 0.07 | 0.05 | 0.32 | 0.16 | 0.08 | 0.30 | 3.00 | 0.21 |
| -73.47729442 | 18.25623694 | 0.01 | 0.02 | 0.06 | 0.03 | 0.37 | 0.13 | 0.05 | 0.30 | 3.00 | 0.11 |
| -73.51232872 | 18.52034163 | 0.01 | 0.01 | 0.05 | 0.03 | 0.42 | 0.13 | 0.04 | 0.30 | 3.00 | 0.25 |
| -72.29475218 | 18.58987124 | 0.33 | 0.36 | 0.40 | 0.41 | 0.43 | 0.61 | 0.59 | 0.30 | 3.00 | 0.40 |
| -72.72917745 | 19.93114579 | 0.03 | 0.05 | 0.09 | 0.10 | 0.32 | 0.26 | 0.15 | 0.30 | 3.00 | 0.32 |
| -72.60547944 | 18.53300788 | 0.03 | 0.03 | 0.06 | 0.04 | 0.33 | 0.17 | 0.08 | 0.30 | 3.00 | 0.49 |
| -73.51232872 | 18.52088062 | 0.01 | 0.02 | 0.06 | 0.03 | 0.43 | 0.13 | 0.04 | 0.30 | 3.00 | 0.06 |
| -73.42150904 | 18.25758441 | 0.02 | 0.02 | 0.05 | 0.03 | 0.38 | 0.15 | 0.07 | 0.30 | 3.00 | 0.03 |
| -72.20581897 | 18.54621311 | 0.03 | 0.04 | 0.09 | 0.06 | 0.35 | 0.22 | 0.11 | 0.30 | 3.00 | 0.67 |
| -72.07295814 | 19.64575102 | 0.03 | 0.04 | 0.07 | 0.08 | 0.28 | 0.29 | 0.17 | 0.30 | 3.00 | 0.46 |
| -73.54520706 | 18.5168382 | 0.01 | 0.01 | 0.04 | 0.02 | 0.40 | 0.12 | 0.05 | 0.30 | 3.00 | 0.20 |
| -73.51259821 | 18.52034163 | 0.01 | 0.02 | 0.05 | 0.03 | 0.42 | 0.13 | 0.05 | 0.30 | 3.00 | 0.45 |
| -72.20878341 | 18.53327737 | 0.04 | 0.06 | 0.11 | 0.10 | 0.35 | 0.22 | 0.13 | 0.30 | 3.00 | 0.69 |
| -72.27454009 | 18.60873586 | 0.03 | 0.04 | 0.07 | 0.05 | 0.31 | 0.16 | 0.09 | 0.30 | 3.00 | 0.48 |
| -72.21040038 | 18.54863857 | 0.03 | 0.03 | 0.07 | 0.04 | 0.37 | 0.18 | 0.08 | 0.30 | 3.00 | 0.27 |
| -73.51152024 | 18.52034163 | 0.01 | 0.02 | 0.06 | 0.03 | 0.42 | 0.16 | 0.06 | 0.30 | 3.00 | 0.64 |
| -73.42150904 | 18.25785391 | 0.02 | 0.02 | 0.06 | 0.04 | 0.36 | 0.16 | 0.08 | 0.30 | 3.00 | 0.04 |
| -72.20878341 | 18.53381636 | 0.02 | 0.02 | 0.06 | 0.04 | 0.33 | 0.15 | 0.07 | 0.30 | 3.00 | 0.37 |
| -72.20581897 | 18.54486564 | 0.03 | 0.04 | 0.08 | 0.06 | 0.34 | 0.19 | 0.10 | 0.30 | 3.00 | 0.56 |
| -72.20986139 | 18.54729109 | 0.05 | 0.06 | 0.10 | 0.10 | 0.33 | 0.29 | 0.16 | 0.30 | 3.00 | 0.62 |
| -73.42097005 | 18.25758441 | 0.02 | 0.03 | 0.06 | 0.04 | 0.39 | 0.17 | 0.08 | 0.30 | 3.00 | 0.44 |
| -73.47729442 | 18.25650643 | 0.01 | 0.02 | 0.06 | 0.03 | 0.39 | 0.14 | 0.05 | 0.30 | 3.00 | 0.52 |
| -72.72944695 | 19.93195427 | 0.03 | 0.05 | 0.09 | 0.09 | 0.29 | 0.21 | 0.13 | 0.30 | 3.00 | 0.64 |
| -73.75622132 | 18.18535986 | 0.02 | 0.03 | 0.07 | 0.06 | 0.33 | 0.18 | 0.09 | 0.30 | 3.00 | 0.43 |
| -72.61652872 | 18.52061113 | 0.03 | 0.03 | 0.06 | 0.04 | 0.29 | 0.15 | 0.08 | 0.30 | 3.00 | 0.41 |
| -72.20851392 | 18.54917755 | 0.03 | 0.03 | 0.08 | 0.05 | 0.38 | 0.17 | 0.07 | 0.30 | 3.00 | 0.45 |
| -72.07322763 | 19.64548153 | 0.03 | 0.04 | 0.07 | 0.08 | 0.28 | 0.28 | 0.16 | 0.30 | 3.00 | 0.57 |
| -72.20959189 | 18.54729109 | 0.05 | 0.07 | 0.10 | 0.10 | 0.32 | 0.31 | 0.18 | 0.30 | 3.00 | 0.44 |
| -72.20716644 | 18.53435535 | 0.02 | 0.02 | 0.06 | 0.04 | 0.36 | 0.14 | 0.06 | 0.30 | 3.00 | 0.49 |
| -72.21093937 | 18.54809958 | 0.03 | 0.04 | 0.09 | 0.06 | 0.37 | 0.21 | 0.10 | 0.30 | 3.00 | 0.51 |
| -72.55238901 | 18.52276709 | 0.03 | 0.03 | 0.07 | 0.06 | 0.34 | 0.21 | 0.10 | 0.30 | 3.00 | 0.42 |
| -72.60655742 | 18.53004344 | 0.03 | 0.04 | 0.07 | 0.06 | 0.28 | 0.22 | 0.12 | 0.30 | 3.00 | 0.11 |
| -72.6176067 | 18.52168911 | 0.02 | 0.03 | 0.06 | 0.04 | 0.30 | 0.16 | 0.08 | 0.30 | 3.00 | 0.34 |
| -72.20824442 | 18.54890806 | 0.03 | 0.03 | 0.08 | 0.05 | 0.38 | 0.18 | 0.08 | 0.30 | 3.00 | 0.36 |
| -72.60709641 | 18.5319299 | 0.02 | 0.02 | 0.05 | 0.03 | 0.33 | 0.14 | 0.06 | 0.30 | 3.00 | 0.06 |
| -72.60494045 | 18.52950445 | 0.02 | 0.02 | 0.05 | 0.03 | 0.32 | 0.15 | 0.06 | 0.30 | 3.00 | 0.66 |
| -72.20581897 | 18.54567412 | 0.05 | 0.06 | 0.10 | 0.10 | 0.35 | 0.30 | 0.17 | 0.30 | 3.00 | 0.17 |
| -73.54439857 | 18.51629922 | 0.01 | 0.02 | 0.05 | 0.03 | 0.37 | 0.13 | 0.05 | 0.30 | 3.00 | 0.02 |
| -73.54439857 | 18.51629922 | 0.01 | 0.02 | 0.05 | 0.03 | 0.37 | 0.13 | 0.05 | 0.30 | 3.00 | 0.19 |
| -72.20878341 | 18.54890806 | 0.04 | 0.05 | 0.09 | 0.08 | 0.35 | 0.25 | 0.12 | 0.30 | 3.00 | 0.01 |
| -72.07214966 | 19.64251709 | 0.03 | 0.04 | 0.07 | 0.07 | 0.30 | 0.28 | 0.16 | 0.30 | 3.00 | 0.55 |
| -72.20608846 | 18.54621311 | 0.03 | 0.04 | 0.08 | 0.06 | 0.36 | 0.21 | 0.10 | 0.30 | 3.00 | 0.31 |
| -72.20959189 | 18.54809958 | 0.05 | 0.06 | 0.10 | 0.11 | 0.33 | 0.30 | 0.18 | 0.30 | 3.00 | 0.56 |
| -71.99399623 | 18.51252629 | 0.07 | 0.09 | 0.12 | 0.14 | 0.28 | 0.34 | 0.23 | 0.30 | 4.00 | 0.01 |
| -72.0996381 | 19.19758153 | 0.02 | 0.04 | 0.07 | 0.08 | 0.24 | 0.24 | 0.13 | 0.30 | 4.00 | 0.00 |
| -72.2289955 | 18.41200481 | 0.04 | 0.05 | 0.09 | 0.12 | 0.21 | 0.24 | 0.18 | 0.31 | 4.00 | 0.31 |
| -71.80481103 | 19.41533315 | 0.03 | 0.03 | 0.06 | 0.06 | 0.22 | 0.24 | 0.14 | 0.30 | 4.00 | 0.13 |
| -72.57017565 | 19.00920481 | 0.04 | 0.05 | 0.10 | 0.09 | 0.30 | 0.31 | 0.20 | 0.30 | 4.00 | 0.09 |
| -72.57421807 | 19.01324723 | 0.05 | 0.06 | 0.11 | 0.12 | 0.25 | 0.32 | 0.23 | 0.30 | 4.00 | 0.41 |
| -72.10071608 | 18.70817936 | 0.05 | 0.07 | 0.12 | 0.12 | 0.34 | 0.35 | 0.23 | 0.30 | 4.00 | 0.57 |
| -72.18479839 | 18.98036889 | 0.04 | 0.05 | 0.09 | 0.12 | 0.32 | 0.36 | 0.20 | 0.30 | 4.00 | 0.57 |
| -72.23249893 | 18.41496925 | 0.04 | 0.05 | 0.10 | 0.13 | 0.22 | 0.24 | 0.17 | 0.31 | 4.00 | 0.11 |
| -72.21498179 | 18.40877088 | 0.05 | 0.06 | 0.11 | 0.14 | 0.23 | 0.30 | 0.21 | 0.31 | 4.00 | 0.44 |
| -72.24624316 | 18.92700896 | 0.04 | 0.05 | 0.09 | 0.11 | 0.36 | 0.27 | 0.16 | 0.30 | 4.00 | 0.58 |
| -71.80292456 | 19.41533315 | 0.03 | 0.04 | 0.06 | 0.07 | 0.23 | 0.25 | 0.15 | 0.30 | 4.00 | 0.21 |
| -72.0999076 | 19.198929 | 0.03 | 0.05 | 0.09 | 0.10 | 0.25 | 0.30 | 0.18 | 0.30 | 4.00 | 0.02 |
| -71.8061585 | 19.41344669 | 0.02 | 0.03 | 0.06 | 0.06 | 0.25 | 0.24 | 0.14 | 0.30 | 4.00 | 0.25 |
| -72.21632926 | 18.40823189 | 0.03 | 0.05 | 0.08 | 0.10 | 0.19 | 0.23 | 0.15 | 0.31 | 4.00 | 0.27 |
| -71.80292456 | 19.41263821 | 0.03 | 0.03 | 0.06 | 0.06 | 0.20 | 0.23 | 0.15 | 0.30 | 4.00 | 0.13 |
| -72.57152312 | 19.01216925 | 0.05 | 0.08 | 0.13 | 0.14 | 0.35 | 0.36 | 0.22 | 0.30 | 4.00 | 0.33 |
| -72.24974659 | 18.92835644 | 0.04 | 0.05 | 0.09 | 0.10 | 0.30 | 0.27 | 0.15 | 0.30 | 4.00 | 0.11 |
| -72.57583504 | 19.01297774 | 0.04 | 0.05 | 0.08 | 0.09 | 0.24 | 0.27 | 0.18 | 0.30 | 4.00 | 0.57 |
| -72.22657005 | 19.24231763 | 0.03 | 0.03 | 0.06 | 0.06 | 0.20 | 0.22 | 0.14 | 0.30 | 4.00 | 0.41 |
| -72.98843125 | 19.85218387 | 0.09 | 0.11 | 0.18 | 0.21 | 0.29 | 0.43 | 0.35 | 0.30 | 4.00 | 0.41 |
| -73.18758774 | 19.65464434 | 0.06 | 0.08 | 0.12 | 0.15 | 0.25 | 0.34 | 0.22 | 0.30 | 4.00 | 0.08 |
| -73.18893522 | 19.65545283 | 0.07 | 0.10 | 0.14 | 0.17 | 0.29 | 0.36 | 0.24 | 0.30 | 4.00 | 0.60 |
| -72.18722384 | 18.97929091 | 0.04 | 0.05 | 0.09 | 0.10 | 0.30 | 0.33 | 0.21 | 0.30 | 4.00 | 0.02 |
| -73.19082168 | 19.65491384 | 0.06 | 0.08 | 0.12 | 0.14 | 0.26 | 0.34 | 0.22 | 0.30 | 4.00 | 0.28 |
| -71.8905103 | 19.27169254 | 0.04 | 0.06 | 0.11 | 0.14 | 0.30 | 0.35 | 0.24 | 0.30 | 4.00 | 0.22 |
| -72.10071608 | 18.70817936 | 0.05 | 0.07 | 0.12 | 0.12 | 0.34 | 0.35 | 0.23 | 0.30 | 4.00 | 0.24 |
| -72.22953449 | 18.41173532 | 0.04 | 0.05 | 0.09 | 0.12 | 0.20 | 0.23 | 0.17 | 0.31 | 4.00 | 0.48 |
| -72.57637402 | 18.85747936 | 0.08 | 0.10 | 0.15 | 0.17 | 0.36 | 0.34 | 0.23 | 0.30 | 4.00 | 0.29 |
| -72.23007348 | 18.41065734 | 0.03 | 0.04 | 0.08 | 0.10 | 0.19 | 0.21 | 0.15 | 0.31 | 4.00 | 0.02 |
| -72.98735327 | 19.85433983 | 0.08 | 0.11 | 0.17 | 0.20 | 0.30 | 0.42 | 0.34 | 0.30 | 4.00 | 0.22 |
| -72.07134117 | 18.69012322 | 0.06 | 0.07 | 0.11 | 0.13 | 0.34 | 0.36 | 0.24 | 0.30 | 4.00 | 0.37 |
| -72.35996987 | 18.4076929 | 0.02 | 0.02 | 0.05 | 0.07 | 0.17 | 0.18 | 0.11 | 0.30 | 4.00 | 0.13 |
| -72.21740724 | 18.40823189 | 0.03 | 0.05 | 0.08 | 0.10 | 0.20 | 0.24 | 0.16 | 0.30 | 4.00 | 0.39 |
| -71.83068251 | 19.20620535 | 0.05 | 0.07 | 0.11 | 0.14 | 0.31 | 0.38 | 0.25 | 0.30 | 4.00 | 0.43 |
| -72.21498179 | 18.41065734 | 0.04 | 0.06 | 0.10 | 0.13 | 0.22 | 0.28 | 0.19 | 0.31 | 4.00 | 0.19 |
| -72.99112619 | 19.85353135 | 0.08 | 0.09 | 0.16 | 0.19 | 0.31 | 0.42 | 0.33 | 0.31 | 4.00 | 0.02 |
| -73.91710959 | 18.32873098 | 0.05 | 0.06 | 0.11 | 0.12 | 0.35 | 0.34 | 0.22 | 0.30 | 4.00 | 0.30 |
| -72.58122493 | 18.85667088 | 0.09 | 0.11 | 0.16 | 0.17 | 0.37 | 0.39 | 0.26 | 0.30 | 4.00 | 0.30 |
| -72.56963666 | 19.0097438 | 0.05 | 0.07 | 0.12 | 0.12 | 0.32 | 0.41 | 0.25 | 0.30 | 4.00 | 0.19 |
| -72.57098413 | 19.00785734 | 0.05 | 0.07 | 0.11 | 0.12 | 0.27 | 0.35 | 0.25 | 0.30 | 4.00 | 0.34 |
| -72.58068594 | 18.85613189 | 0.08 | 0.10 | 0.15 | 0.17 | 0.37 | 0.34 | 0.22 | 0.30 | 4.00 | 0.42 |
| -72.23357691 | 18.41389127 | 0.04 | 0.06 | 0.11 | 0.14 | 0.22 | 0.25 | 0.19 | 0.31 | 4.00 | 0.25 |
| -71.89024081 | 19.27330951 | 0.04 | 0.05 | 0.09 | 0.12 | 0.32 | 0.32 | 0.22 | 0.30 | 4.00 | 0.23 |
| -72.22872601 | 18.41146582 | 0.03 | 0.05 | 0.09 | 0.12 | 0.21 | 0.25 | 0.18 | 0.31 | 4.00 | 0.11 |
| -72.15138106 | 18.70952683 | 0.04 | 0.06 | 0.09 | 0.11 | 0.31 | 0.33 | 0.21 | 0.30 | 4.00 | 0.58 |
| -72.98870074 | 19.85137539 | 0.09 | 0.11 | 0.18 | 0.21 | 0.31 | 0.44 | 0.35 | 0.30 | 4.00 | 0.62 |
| -72.57394857 | 19.01270824 | 0.07 | 0.09 | 0.14 | 0.15 | 0.31 | 0.39 | 0.28 | 0.30 | 4.00 | 0.69 |
| -73.17060958 | 19.78966113 | 0.06 | 0.07 | 0.10 | 0.11 | 0.31 | 0.37 | 0.24 | 0.30 | 4.00 | 0.54 |
| -72.57233161 | 19.01082178 | 0.05 | 0.06 | 0.12 | 0.11 | 0.30 | 0.36 | 0.23 | 0.30 | 4.00 | 0.24 |
| -72.57233161 | 19.01351672 | 0.06 | 0.08 | 0.12 | 0.14 | 0.26 | 0.37 | 0.26 | 0.30 | 4.00 | 0.42 |
| -72.58041644 | 18.85667088 | 0.08 | 0.10 | 0.14 | 0.16 | 0.36 | 0.35 | 0.23 | 0.30 | 4.00 | 0.19 |
| -72.57475706 | 19.01028279 | 0.04 | 0.06 | 0.11 | 0.12 | 0.28 | 0.35 | 0.23 | 0.30 | 4.00 | 0.61 |
| -72.57421807 | 19.01163026 | 0.05 | 0.07 | 0.13 | 0.14 | 0.32 | 0.38 | 0.25 | 0.30 | 4.00 | 0.50 |
| -72.57421807 | 19.00408441 | 0.05 | 0.07 | 0.11 | 0.12 | 0.31 | 0.37 | 0.25 | 0.30 | 4.00 | 0.42 |
| -72.27777402 | 18.32091564 | 0.03 | 0.05 | 0.10 | 0.13 | 0.26 | 0.26 | 0.18 | 0.30 | 4.00 | 0.01 |
| -73.17087908 | 19.78966113 | 0.07 | 0.08 | 0.11 | 0.11 | 0.31 | 0.38 | 0.26 | 0.30 | 4.00 | 0.69 |
| -72.38179893 | 18.73027792 | 0.08 | 0.10 | 0.14 | 0.17 | 0.33 | 0.36 | 0.24 | 0.30 | 4.00 | 0.29 |
| -72.57017565 | 19.00839633 | 0.06 | 0.07 | 0.11 | 0.13 | 0.27 | 0.38 | 0.26 | 0.30 | 4.00 | 0.40 |
| -72.51061735 | 18.83160788 | 0.07 | 0.09 | 0.13 | 0.16 | 0.31 | 0.31 | 0.21 | 0.30 | 4.00 | 0.59 |
| -72.57017565 | 19.00839633 | 0.06 | 0.07 | 0.11 | 0.13 | 0.27 | 0.38 | 0.26 | 0.30 | 4.00 | 0.29 |
| -72.21659875 | 18.40877088 | 0.04 | 0.06 | 0.09 | 0.12 | 0.21 | 0.26 | 0.18 | 0.31 | 4.00 | 0.51 |
| -72.57071464 | 19.00920481 | 0.04 | 0.06 | 0.10 | 0.12 | 0.31 | 0.35 | 0.21 | 0.30 | 4.00 | 0.20 |
| -72.57987745 | 18.85451492 | 0.09 | 0.10 | 0.15 | 0.18 | 0.38 | 0.39 | 0.24 | 0.30 | 4.00 | 0.44 |
| -72.0699937 | 18.68985373 | 0.05 | 0.07 | 0.11 | 0.13 | 0.32 | 0.34 | 0.24 | 0.30 | 4.00 | 0.13 |
| -72.51061735 | 18.83241636 | 0.06 | 0.08 | 0.13 | 0.15 | 0.31 | 0.32 | 0.20 | 0.30 | 4.00 | 0.00 |
| -72.22683955 | 19.24285662 | 0.03 | 0.03 | 0.05 | 0.06 | 0.19 | 0.21 | 0.13 | 0.30 | 4.00 | 0.30 |
| -72.32655254 | 18.70386745 | 0.10 | 0.12 | 0.17 | 0.19 | 0.34 | 0.40 | 0.28 | 0.30 | 4.00 | 0.25 |
| -72.57691301 | 18.85720987 | 0.08 | 0.10 | 0.14 | 0.17 | 0.37 | 0.34 | 0.23 | 0.30 | 4.00 | 0.03 |
| -71.83068251 | 19.2086308 | 0.05 | 0.07 | 0.11 | 0.14 | 0.29 | 0.35 | 0.25 | 0.30 | 4.00 | 0.13 |
| -72.32601355 | 18.70332846 | 0.09 | 0.11 | 0.15 | 0.18 | 0.33 | 0.38 | 0.26 | 0.30 | 4.00 | 0.44 |
| -71.89158828 | 19.27088405 | 0.04 | 0.05 | 0.09 | 0.13 | 0.28 | 0.35 | 0.23 | 0.30 | 4.00 | 0.24 |
| -72.57206211 | 19.01163026 | 0.05 | 0.07 | 0.12 | 0.13 | 0.32 | 0.37 | 0.24 | 0.30 | 4.00 | 0.43 |
| -72.22710904 | 19.24150914 | 0.03 | 0.03 | 0.05 | 0.06 | 0.19 | 0.22 | 0.13 | 0.30 | 4.00 | 0.50 |
| -72.21632926 | 18.40823189 | 0.03 | 0.05 | 0.08 | 0.10 | 0.19 | 0.23 | 0.15 | 0.31 | 4.00 | 0.15 |
| -72.22495309 | 19.24123965 | 0.02 | 0.03 | 0.06 | 0.07 | 0.20 | 0.23 | 0.13 | 0.30 | 4.00 | 0.35 |
| -72.07188016 | 18.69093171 | 0.06 | 0.07 | 0.12 | 0.13 | 0.33 | 0.37 | 0.25 | 0.30 | 4.00 | 0.01 |
| -72.57826049 | 18.85801835 | 0.07 | 0.09 | 0.13 | 0.15 | 0.33 | 0.32 | 0.20 | 0.30 | 4.00 | 0.28 |
| -72.99328215 | 19.85380084 | 0.07 | 0.08 | 0.14 | 0.18 | 0.30 | 0.39 | 0.30 | 0.31 | 4.00 | 0.18 |
| -72.98762276 | 19.85380084 | 0.08 | 0.10 | 0.16 | 0.20 | 0.31 | 0.43 | 0.34 | 0.31 | 4.00 | 0.56 |
| -72.21579027 | 18.40850138 | 0.04 | 0.05 | 0.10 | 0.12 | 0.20 | 0.25 | 0.17 | 0.31 | 4.00 | 0.18 |
| -72.99193467 | 19.85433983 | 0.08 | 0.10 | 0.16 | 0.20 | 0.31 | 0.44 | 0.36 | 0.31 | 4.00 | 0.58 |
| -72.09882962 | 19.198929 | 0.03 | 0.04 | 0.08 | 0.09 | 0.24 | 0.25 | 0.14 | 0.30 | 4.00 | 0.11 |
| -72.24273973 | 18.2980086 | 0.04 | 0.06 | 0.10 | 0.13 | 0.28 | 0.33 | 0.24 | 0.30 | 4.00 | 0.02 |
| -72.09882962 | 19.19785102 | 0.02 | 0.04 | 0.06 | 0.08 | 0.23 | 0.24 | 0.13 | 0.30 | 4.00 | 0.33 |
| -72.09775164 | 19.20000698 | 0.03 | 0.04 | 0.07 | 0.08 | 0.23 | 0.23 | 0.14 | 0.30 | 4.00 | 0.62 |
| -72.98816175 | 19.85353135 | 0.08 | 0.10 | 0.16 | 0.19 | 0.31 | 0.42 | 0.33 | 0.31 | 4.00 | 0.42 |
| -72.23169045 | 18.41281329 | 0.03 | 0.05 | 0.08 | 0.11 | 0.20 | 0.22 | 0.16 | 0.31 | 4.00 | 0.45 |
| -71.89131879 | 19.27034506 | 0.04 | 0.05 | 0.09 | 0.13 | 0.32 | 0.33 | 0.22 | 0.30 | 4.00 | 0.50 |
| -72.58122493 | 18.85613189 | 0.08 | 0.10 | 0.15 | 0.17 | 0.38 | 0.38 | 0.24 | 0.30 | 4.00 | 0.15 |
| -72.06810724 | 18.69039272 | 0.06 | 0.08 | 0.12 | 0.14 | 0.35 | 0.37 | 0.24 | 0.30 | 4.00 | 0.51 |
| -72.99139569 | 19.85353135 | 0.07 | 0.10 | 0.16 | 0.18 | 0.31 | 0.42 | 0.32 | 0.31 | 4.00 | 0.68 |
| -72.21767673 | 18.40850138 | 0.03 | 0.04 | 0.07 | 0.09 | 0.18 | 0.21 | 0.14 | 0.30 | 4.00 | 0.12 |
| -72.57826049 | 18.85720987 | 0.07 | 0.09 | 0.13 | 0.15 | 0.34 | 0.34 | 0.21 | 0.30 | 4.00 | 0.66 |
| -72.09909911 | 19.19919849 | 0.03 | 0.04 | 0.08 | 0.10 | 0.22 | 0.26 | 0.15 | 0.30 | 4.00 | 0.16 |
| -72.06918521 | 18.68958423 | 0.05 | 0.07 | 0.11 | 0.13 | 0.34 | 0.35 | 0.23 | 0.30 | 4.00 | 0.51 |
| -72.57852998 | 18.8555929 | 0.09 | 0.11 | 0.16 | 0.19 | 0.40 | 0.40 | 0.25 | 0.30 | 4.00 | 0.28 |
| -73.19216915 | 19.65464434 | 0.06 | 0.08 | 0.11 | 0.13 | 0.25 | 0.35 | 0.22 | 0.30 | 4.00 | 0.56 |
| -72.35700543 | 18.40661492 | 0.03 | 0.05 | 0.09 | 0.11 | 0.26 | 0.28 | 0.18 | 0.30 | 4.00 | 0.22 |
| -72.21632926 | 18.41065734 | 0.03 | 0.04 | 0.08 | 0.10 | 0.17 | 0.19 | 0.13 | 0.30 | 4.00 | 0.45 |
| -72.57529605 | 19.01055228 | 0.04 | 0.06 | 0.10 | 0.11 | 0.26 | 0.34 | 0.23 | 0.30 | 4.00 | 0.70 |
| -71.82987402 | 19.20701384 | 0.04 | 0.06 | 0.11 | 0.12 | 0.26 | 0.31 | 0.21 | 0.30 | 4.00 | 0.15 |
| -72.07080218 | 18.6912012 | 0.06 | 0.08 | 0.12 | 0.14 | 0.35 | 0.38 | 0.26 | 0.30 | 4.00 | 0.25 |
| -72.57448756 | 19.01432521 | 0.05 | 0.07 | 0.11 | 0.12 | 0.25 | 0.32 | 0.22 | 0.30 | 4.00 | 0.21 |
| -72.57718251 | 18.85720987 | 0.07 | 0.09 | 0.13 | 0.15 | 0.35 | 0.31 | 0.20 | 0.30 | 4.00 | 0.58 |
| -71.8905103 | 19.27007557 | 0.04 | 0.06 | 0.09 | 0.12 | 0.28 | 0.31 | 0.21 | 0.30 | 4.00 | 0.36 |
| -72.24759063 | 18.92862593 | 0.03 | 0.04 | 0.09 | 0.10 | 0.37 | 0.26 | 0.15 | 0.30 | 4.00 | 0.13 |
| -72.21579027 | 18.40984885 | 0.04 | 0.05 | 0.09 | 0.12 | 0.20 | 0.23 | 0.16 | 0.31 | 4.00 | 0.62 |
| -72.3586224 | 18.4074234 | 0.03 | 0.05 | 0.08 | 0.10 | 0.23 | 0.26 | 0.17 | 0.30 | 4.00 | 0.16 |
| -71.80427204 | 19.41506366 | 0.03 | 0.04 | 0.06 | 0.06 | 0.20 | 0.24 | 0.15 | 0.30 | 4.00 | 0.60 |
| -72.57637402 | 18.85720987 | 0.08 | 0.10 | 0.15 | 0.18 | 0.37 | 0.36 | 0.24 | 0.30 | 4.00 | 0.03 |
| -72.57987745 | 18.85640138 | 0.08 | 0.09 | 0.14 | 0.16 | 0.36 | 0.35 | 0.22 | 0.30 | 4.00 | 0.41 |
| -72.57691301 | 18.85720987 | 0.08 | 0.10 | 0.14 | 0.17 | 0.37 | 0.34 | 0.23 | 0.30 | 4.00 | 0.20 |
| -72.98950922 | 19.85191438 | 0.09 | 0.11 | 0.17 | 0.21 | 0.30 | 0.45 | 0.37 | 0.31 | 4.00 | 0.05 |
| -73.18785724 | 19.65518333 | 0.06 | 0.08 | 0.12 | 0.14 | 0.26 | 0.34 | 0.22 | 0.30 | 4.00 | 0.49 |
| -72.09856012 | 19.19919849 | 0.03 | 0.04 | 0.07 | 0.08 | 0.25 | 0.25 | 0.14 | 0.30 | 4.00 | 0.13 |
| -72.98950922 | 19.85460933 | 0.09 | 0.11 | 0.17 | 0.21 | 0.28 | 0.44 | 0.36 | 0.31 | 4.00 | 0.08 |
| -72.07080218 | 18.68931474 | 0.06 | 0.08 | 0.12 | 0.14 | 0.35 | 0.36 | 0.23 | 0.30 | 4.00 | 0.21 |
| -72.3685937 | 18.42278459 | 0.03 | 0.04 | 0.08 | 0.11 | 0.26 | 0.29 | 0.18 | 0.31 | 4.00 | 0.58 |
| -72.21713774 | 18.40904037 | 0.03 | 0.05 | 0.08 | 0.11 | 0.19 | 0.24 | 0.16 | 0.30 | 4.00 | 0.05 |
| -72.57394857 | 19.00704885 | 0.05 | 0.07 | 0.12 | 0.13 | 0.27 | 0.37 | 0.25 | 0.30 | 4.00 | 0.14 |
| -72.06756825 | 18.69254867 | 0.05 | 0.07 | 0.10 | 0.12 | 0.31 | 0.36 | 0.24 | 0.30 | 4.00 | 0.50 |
| -72.57044514 | 19.00947431 | 0.05 | 0.07 | 0.11 | 0.13 | 0.30 | 0.37 | 0.23 | 0.30 | 4.00 | 0.44 |
| -72.57233161 | 19.01028279 | 0.05 | 0.07 | 0.12 | 0.12 | 0.33 | 0.37 | 0.23 | 0.30 | 4.00 | 0.48 |
| -72.23276843 | 18.4125438 | 0.03 | 0.05 | 0.10 | 0.12 | 0.20 | 0.21 | 0.16 | 0.31 | 4.00 | 0.24 |
| -73.91603161 | 18.32846149 | 0.04 | 0.05 | 0.09 | 0.11 | 0.34 | 0.32 | 0.19 | 0.30 | 4.00 | 0.11 |
| -71.83041301 | 19.20701384 | 0.06 | 0.08 | 0.13 | 0.16 | 0.31 | 0.38 | 0.26 | 0.30 | 4.00 | 0.04 |
| -72.57421807 | 19.01378622 | 0.04 | 0.06 | 0.10 | 0.10 | 0.25 | 0.29 | 0.21 | 0.30 | 4.00 | 0.26 |
| -72.23142096 | 18.41146582 | 0.04 | 0.05 | 0.09 | 0.12 | 0.21 | 0.24 | 0.17 | 0.31 | 4.00 | 0.65 |
| -72.24705164 | 18.92862593 | 0.03 | 0.04 | 0.09 | 0.10 | 0.35 | 0.26 | 0.14 | 0.30 | 4.00 | 0.10 |
| -72.98923973 | 19.85433983 | 0.09 | 0.11 | 0.17 | 0.20 | 0.28 | 0.42 | 0.34 | 0.31 | 4.00 | 0.11 |
| -72.57718251 | 18.85774885 | 0.07 | 0.09 | 0.13 | 0.15 | 0.35 | 0.33 | 0.21 | 0.30 | 4.00 | 0.18 |
| -72.57664352 | 18.85747936 | 0.07 | 0.09 | 0.14 | 0.15 | 0.35 | 0.35 | 0.23 | 0.30 | 4.00 | 0.21 |
| -72.10071608 | 18.70790987 | 0.05 | 0.07 | 0.11 | 0.11 | 0.34 | 0.33 | 0.21 | 0.30 | 4.00 | 0.22 |
| -72.21632926 | 18.40957936 | 0.03 | 0.05 | 0.08 | 0.11 | 0.18 | 0.22 | 0.15 | 0.31 | 4.00 | 0.07 |
| -72.21740724 | 18.41038784 | 0.02 | 0.03 | 0.05 | 0.07 | 0.12 | 0.15 | 0.10 | 0.30 | 4.00 | 0.14 |
| -72.07134117 | 18.69039272 | 0.06 | 0.08 | 0.11 | 0.14 | 0.34 | 0.38 | 0.26 | 0.30 | 4.00 | 0.30 |
| -72.06729875 | 18.69012322 | 0.06 | 0.07 | 0.12 | 0.14 | 0.35 | 0.38 | 0.24 | 0.30 | 4.00 | 0.10 |
| -71.89185778 | 19.273579 | 0.03 | 0.04 | 0.07 | 0.10 | 0.31 | 0.30 | 0.19 | 0.30 | 4.00 | 0.19 |
| -72.57233161 | 19.00677936 | 0.07 | 0.09 | 0.13 | 0.16 | 0.31 | 0.44 | 0.30 | 0.30 | 4.00 | 0.13 |
| -71.80454153 | 19.41479416 | 0.03 | 0.04 | 0.06 | 0.06 | 0.19 | 0.23 | 0.15 | 0.30 | 4.00 | 0.38 |
| -72.22522258 | 19.24097015 | 0.02 | 0.03 | 0.06 | 0.07 | 0.20 | 0.22 | 0.13 | 0.30 | 4.00 | 0.11 |
| -72.22818702 | 18.41038784 | 0.04 | 0.06 | 0.10 | 0.13 | 0.22 | 0.26 | 0.18 | 0.31 | 4.00 | 0.14 |
| -72.58014695 | 18.85613189 | 0.09 | 0.10 | 0.15 | 0.18 | 0.38 | 0.36 | 0.23 | 0.30 | 4.00 | 0.10 |
| -72.07053269 | 18.69227918 | 0.06 | 0.08 | 0.13 | 0.15 | 0.34 | 0.38 | 0.25 | 0.30 | 4.00 | 0.30 |
| -72.57287059 | 19.0100133 | 0.05 | 0.06 | 0.10 | 0.11 | 0.27 | 0.35 | 0.23 | 0.30 | 4.00 | 0.13 |
| -72.57799099 | 18.85801835 | 0.07 | 0.09 | 0.13 | 0.15 | 0.35 | 0.32 | 0.20 | 0.30 | 4.00 | 0.22 |
| -72.57960796 | 18.85451492 | 0.09 | 0.10 | 0.15 | 0.18 | 0.38 | 0.39 | 0.24 | 0.30 | 4.00 | 0.11 |
| -72.09829063 | 19.20000698 | 0.03 | 0.04 | 0.07 | 0.09 | 0.22 | 0.25 | 0.15 | 0.30 | 4.00 | 0.41 |
| -72.38099045 | 18.7310864 | 0.09 | 0.11 | 0.15 | 0.17 | 0.35 | 0.39 | 0.26 | 0.30 | 4.00 | 0.04 |
| -72.57367908 | 19.01405571 | 0.05 | 0.06 | 0.09 | 0.10 | 0.25 | 0.31 | 0.22 | 0.30 | 4.00 | 0.65 |
| -74.02194298 | 18.33088694 | 0.02 | 0.04 | 0.08 | 0.08 | 0.33 | 0.27 | 0.17 | 0.30 | 4.00 | 0.20 |
| -72.06837673 | 18.68931474 | 0.07 | 0.09 | 0.13 | 0.16 | 0.39 | 0.41 | 0.24 | 0.30 | 4.00 | 0.39 |
| -72.98843125 | 19.85299236 | 0.07 | 0.10 | 0.15 | 0.19 | 0.30 | 0.41 | 0.32 | 0.31 | 4.00 | 0.46 |
| -72.09882962 | 19.1986595 | 0.03 | 0.04 | 0.07 | 0.08 | 0.26 | 0.24 | 0.13 | 0.30 | 4.00 | 0.14 |
| -72.21390381 | 18.41011835 | 0.04 | 0.06 | 0.10 | 0.13 | 0.21 | 0.28 | 0.19 | 0.31 | 4.00 | 0.53 |
| -72.3583529 | 18.40823189 | 0.04 | 0.05 | 0.08 | 0.10 | 0.22 | 0.24 | 0.16 | 0.30 | 4.00 | 0.24 |
| -72.21632926 | 18.40877088 | 0.04 | 0.05 | 0.10 | 0.12 | 0.21 | 0.25 | 0.17 | 0.31 | 4.00 | 0.13 |
| -72.38045146 | 18.73054741 | 0.10 | 0.12 | 0.17 | 0.20 | 0.37 | 0.42 | 0.29 | 0.30 | 4.00 | 0.45 |
| -72.99274316 | 19.85460933 | 0.07 | 0.08 | 0.15 | 0.17 | 0.31 | 0.40 | 0.30 | 0.31 | 4.00 | 0.25 |
| -72.36751572 | 18.42332358 | 0.04 | 0.05 | 0.09 | 0.11 | 0.31 | 0.29 | 0.18 | 0.30 | 4.00 | 0.06 |
| -72.57367908 | 19.0097438 | 0.06 | 0.07 | 0.11 | 0.13 | 0.31 | 0.39 | 0.26 | 0.30 | 4.00 | 0.42 |
| -72.23142096 | 18.4125438 | 0.03 | 0.05 | 0.09 | 0.12 | 0.22 | 0.23 | 0.16 | 0.31 | 4.00 | 0.07 |
| -72.06810724 | 18.68931474 | 0.07 | 0.09 | 0.14 | 0.16 | 0.39 | 0.40 | 0.26 | 0.30 | 4.00 | 0.46 |
| -72.57179262 | 19.00893532 | 0.06 | 0.08 | 0.13 | 0.15 | 0.38 | 0.40 | 0.26 | 0.30 | 4.00 | 0.67 |
| -72.99112619 | 19.85407034 | 0.09 | 0.11 | 0.17 | 0.20 | 0.30 | 0.45 | 0.37 | 0.31 | 4.00 | 0.02 |
| -71.89024081 | 19.27115355 | 0.03 | 0.04 | 0.08 | 0.11 | 0.31 | 0.31 | 0.19 | 0.30 | 4.00 | 0.54 |
| -71.8907798 | 19.27277052 | 0.04 | 0.06 | 0.09 | 0.11 | 0.30 | 0.33 | 0.22 | 0.30 | 4.00 | 0.28 |
| -71.82933503 | 19.20701384 | 0.04 | 0.06 | 0.09 | 0.12 | 0.27 | 0.29 | 0.20 | 0.30 | 4.00 | 0.26 |
| -72.57448756 | 19.0048929 | 0.06 | 0.07 | 0.12 | 0.12 | 0.30 | 0.37 | 0.24 | 0.30 | 4.00 | 0.30 |
| -73.18920471 | 19.65626131 | 0.06 | 0.08 | 0.12 | 0.14 | 0.26 | 0.34 | 0.22 | 0.30 | 4.00 | 0.52 |
| -72.21579027 | 18.41065734 | 0.04 | 0.06 | 0.10 | 0.13 | 0.21 | 0.24 | 0.16 | 0.31 | 4.00 | 0.20 |
| -72.06271734 | 18.69066221 | 0.06 | 0.08 | 0.14 | 0.16 | 0.38 | 0.41 | 0.25 | 0.30 | 4.00 | 0.50 |
| -71.8907798 | 19.27546546 | 0.03 | 0.04 | 0.08 | 0.10 | 0.30 | 0.32 | 0.20 | 0.30 | 4.00 | 0.66 |
| -71.82987402 | 19.20809182 | 0.05 | 0.06 | 0.10 | 0.13 | 0.25 | 0.32 | 0.21 | 0.30 | 4.00 | 0.53 |
| -73.17249605 | 19.78939164 | 0.05 | 0.06 | 0.09 | 0.09 | 0.29 | 0.32 | 0.22 | 0.30 | 4.00 | 0.54 |
| -72.27804352 | 18.32064615 | 0.04 | 0.05 | 0.11 | 0.15 | 0.28 | 0.28 | 0.19 | 0.30 | 4.00 | 0.03 |
| -72.32628305 | 18.70305896 | 0.09 | 0.10 | 0.14 | 0.16 | 0.33 | 0.36 | 0.24 | 0.30 | 4.00 | 0.31 |
| -73.17222655 | 19.78939164 | 0.05 | 0.05 | 0.08 | 0.09 | 0.27 | 0.31 | 0.20 | 0.30 | 4.00 | 0.35 |
| -71.80588901 | 19.41398568 | 0.03 | 0.04 | 0.07 | 0.08 | 0.25 | 0.28 | 0.16 | 0.30 | 4.00 | 0.20 |
| -72.07026319 | 18.69200968 | 0.07 | 0.09 | 0.13 | 0.16 | 0.35 | 0.40 | 0.26 | 0.30 | 4.00 | 0.56 |
| -73.17249605 | 19.78939164 | 0.05 | 0.06 | 0.09 | 0.09 | 0.29 | 0.32 | 0.22 | 0.30 | 4.00 | 0.40 |
| -72.37883449 | 18.73135589 | 0.08 | 0.10 | 0.14 | 0.17 | 0.32 | 0.38 | 0.29 | 0.30 | 4.00 | 0.48 |
| -72.24597366 | 18.92781745 | 0.03 | 0.05 | 0.10 | 0.11 | 0.34 | 0.28 | 0.15 | 0.30 | 4.00 | 0.58 |
| -73.17114857 | 19.78993062 | 0.05 | 0.06 | 0.09 | 0.10 | 0.28 | 0.34 | 0.22 | 0.30 | 4.00 | 0.00 |
| -72.57933846 | 18.85801835 | 0.07 | 0.08 | 0.12 | 0.14 | 0.35 | 0.33 | 0.21 | 0.30 | 4.00 | 0.03 |
| -72.07107168 | 18.69039272 | 0.06 | 0.08 | 0.12 | 0.14 | 0.34 | 0.36 | 0.24 | 0.30 | 4.00 | 0.10 |
| -72.22953449 | 18.41119633 | 0.03 | 0.05 | 0.09 | 0.12 | 0.21 | 0.23 | 0.17 | 0.31 | 4.00 | 0.12 |
| -72.57502655 | 19.01351672 | 0.05 | 0.07 | 0.11 | 0.12 | 0.27 | 0.32 | 0.23 | 0.30 | 4.00 | 0.20 |
| -71.82960453 | 19.20755283 | 0.06 | 0.08 | 0.15 | 0.16 | 0.32 | 0.39 | 0.27 | 0.30 | 4.00 | 0.30 |
| -72.57529605 | 19.01270824 | 0.05 | 0.06 | 0.11 | 0.11 | 0.24 | 0.30 | 0.22 | 0.30 | 4.00 | 0.52 |
| -71.99399623 | 18.51252629 | 0.07 | 0.09 | 0.12 | 0.14 | 0.28 | 0.34 | 0.23 | 0.30 | 4.00 | 0.02 |
| -72.21686825 | 18.40930986 | 0.03 | 0.05 | 0.08 | 0.11 | 0.19 | 0.25 | 0.16 | 0.30 | 4.00 | 0.11 |
| -73.19001319 | 19.65491384 | 0.06 | 0.08 | 0.12 | 0.14 | 0.26 | 0.35 | 0.23 | 0.30 | 4.00 | 0.67 |
| -72.99193467 | 19.85460933 | 0.08 | 0.10 | 0.17 | 0.20 | 0.31 | 0.44 | 0.35 | 0.31 | 4.00 | 0.50 |
| -72.32709153 | 18.70251997 | 0.10 | 0.12 | 0.17 | 0.21 | 0.36 | 0.43 | 0.31 | 0.30 | 4.00 | 0.44 |
| -71.82933503 | 19.20620535 | 0.05 | 0.07 | 0.11 | 0.13 | 0.26 | 0.33 | 0.23 | 0.30 | 4.00 | 0.15 |
| -71.80535002 | 19.41425517 | 0.03 | 0.04 | 0.07 | 0.07 | 0.23 | 0.28 | 0.16 | 0.30 | 4.00 | 0.39 |
| -72.07214966 | 18.69174019 | 0.06 | 0.08 | 0.12 | 0.13 | 0.33 | 0.34 | 0.23 | 0.30 | 4.00 | 0.39 |
| -72.22845651 | 18.41119633 | 0.04 | 0.06 | 0.10 | 0.12 | 0.21 | 0.25 | 0.17 | 0.31 | 4.00 | 0.55 |
| -72.98762276 | 19.85514831 | 0.09 | 0.11 | 0.18 | 0.22 | 0.29 | 0.47 | 0.39 | 0.31 | 4.00 | 0.53 |
| -73.91684009 | 18.32900048 | 0.04 | 0.05 | 0.09 | 0.09 | 0.31 | 0.29 | 0.17 | 0.30 | 4.00 | 0.44 |
| -73.91549262 | 18.32846149 | 0.03 | 0.04 | 0.08 | 0.10 | 0.32 | 0.28 | 0.17 | 0.30 | 4.00 | 0.64 |
| -72.98897023 | 19.85541781 | 0.09 | 0.11 | 0.18 | 0.22 | 0.30 | 0.46 | 0.38 | 0.31 | 4.00 | 0.19 |
| -73.98286626 | 18.33870228 | 0.02 | 0.03 | 0.06 | 0.06 | 0.28 | 0.17 | 0.10 | 0.30 | 4.00 | 0.60 |
| -72.57367908 | 19.01189976 | 0.06 | 0.08 | 0.14 | 0.14 | 0.34 | 0.38 | 0.26 | 0.30 | 4.00 | 0.42 |
| -72.10017709 | 19.19812052 | 0.03 | 0.04 | 0.07 | 0.09 | 0.25 | 0.27 | 0.15 | 0.30 | 4.00 | 0.19 |
| -72.24812962 | 18.92781745 | 0.05 | 0.07 | 0.12 | 0.14 | 0.41 | 0.35 | 0.21 | 0.30 | 4.00 | 0.40 |
| -72.57179262 | 19.01216925 | 0.05 | 0.08 | 0.15 | 0.15 | 0.37 | 0.39 | 0.26 | 0.30 | 4.00 | 0.67 |
| -72.06568179 | 18.69039272 | 0.06 | 0.08 | 0.12 | 0.15 | 0.35 | 0.38 | 0.24 | 0.30 | 4.00 | 0.06 |
| -72.24759063 | 18.92943441 | 0.03 | 0.05 | 0.09 | 0.10 | 0.36 | 0.26 | 0.14 | 0.30 | 4.00 | 0.16 |
| -72.06541229 | 18.69012322 | 0.06 | 0.08 | 0.13 | 0.15 | 0.35 | 0.39 | 0.25 | 0.30 | 4.00 | 0.54 |
| -72.06810724 | 18.69066221 | 0.06 | 0.07 | 0.12 | 0.14 | 0.34 | 0.37 | 0.24 | 0.30 | 4.00 | 0.42 |
| -72.57314009 | 19.01136077 | 0.05 | 0.07 | 0.13 | 0.13 | 0.32 | 0.37 | 0.24 | 0.30 | 4.00 | 0.30 |
| -72.57448756 | 19.01432521 | 0.05 | 0.07 | 0.11 | 0.12 | 0.25 | 0.32 | 0.22 | 0.30 | 4.00 | 0.13 |
| -72.57529605 | 19.00920481 | 0.05 | 0.07 | 0.10 | 0.11 | 0.27 | 0.38 | 0.24 | 0.30 | 4.00 | 0.37 |
| -72.23007348 | 18.41146582 | 0.03 | 0.05 | 0.09 | 0.12 | 0.20 | 0.24 | 0.17 | 0.31 | 4.00 | 0.47 |
| -72.577452 | 18.85855734 | 0.08 | 0.09 | 0.13 | 0.16 | 0.36 | 0.36 | 0.23 | 0.30 | 4.00 | 0.55 |
| -72.229265 | 18.41092683 | 0.04 | 0.05 | 0.10 | 0.12 | 0.21 | 0.24 | 0.17 | 0.31 | 4.00 | 0.05 |
| -71.83041301 | 19.20836131 | 0.05 | 0.07 | 0.11 | 0.14 | 0.30 | 0.37 | 0.25 | 0.30 | 4.00 | 0.61 |
| -72.57017565 | 19.00866582 | 0.04 | 0.06 | 0.10 | 0.11 | 0.26 | 0.33 | 0.22 | 0.30 | 4.00 | 0.57 |
| -71.80346355 | 19.41398568 | 0.03 | 0.03 | 0.05 | 0.06 | 0.22 | 0.23 | 0.13 | 0.30 | 4.00 | 0.63 |
| -72.35619695 | 18.40688441 | 0.03 | 0.04 | 0.07 | 0.08 | 0.23 | 0.26 | 0.15 | 0.30 | 4.00 | 0.33 |
| -72.38045146 | 18.73000842 | 0.07 | 0.09 | 0.13 | 0.15 | 0.33 | 0.32 | 0.22 | 0.30 | 4.00 | 0.10 |
| -72.2144428 | 18.40877088 | 0.05 | 0.07 | 0.12 | 0.15 | 0.24 | 0.30 | 0.22 | 0.31 | 4.00 | 0.30 |
| -72.38018197 | 18.73162539 | 0.08 | 0.10 | 0.15 | 0.17 | 0.36 | 0.40 | 0.26 | 0.30 | 4.00 | 0.57 |
| -72.57071464 | 19.01082178 | 0.03 | 0.05 | 0.09 | 0.09 | 0.32 | 0.32 | 0.19 | 0.30 | 4.00 | 0.30 |
| -71.82879604 | 19.20836131 | 0.06 | 0.08 | 0.13 | 0.16 | 0.31 | 0.37 | 0.26 | 0.30 | 4.00 | 0.49 |
| -72.57421807 | 19.00624037 | 0.05 | 0.06 | 0.12 | 0.11 | 0.28 | 0.34 | 0.23 | 0.30 | 4.00 | 0.41 |
| -72.54187872 | 18.8954781 | 0.06 | 0.08 | 0.12 | 0.13 | 0.32 | 0.36 | 0.25 | 0.30 | 4.00 | 0.65 |
| -71.80481103 | 19.41398568 | 0.03 | 0.03 | 0.06 | 0.06 | 0.25 | 0.24 | 0.13 | 0.30 | 4.00 | 0.20 |
| -72.32601355 | 18.70413694 | 0.09 | 0.11 | 0.15 | 0.17 | 0.34 | 0.38 | 0.26 | 0.30 | 4.00 | 0.54 |
| -73.18893522 | 19.65545283 | 0.07 | 0.10 | 0.14 | 0.17 | 0.29 | 0.36 | 0.24 | 0.30 | 4.00 | 0.31 |
| -72.22953449 | 18.41200481 | 0.04 | 0.05 | 0.09 | 0.12 | 0.20 | 0.24 | 0.17 | 0.31 | 4.00 | 0.05 |
| -72.57071464 | 19.01109127 | 0.04 | 0.05 | 0.10 | 0.11 | 0.34 | 0.35 | 0.21 | 0.30 | 4.00 | 0.66 |
| -72.57206211 | 19.01351672 | 0.06 | 0.08 | 0.11 | 0.14 | 0.27 | 0.37 | 0.26 | 0.30 | 4.00 | 0.59 |
| -72.57287059 | 19.00920481 | 0.06 | 0.07 | 0.11 | 0.13 | 0.31 | 0.36 | 0.24 | 0.30 | 4.00 | 0.39 |
| -71.830952 | 19.20647485 | 0.06 | 0.08 | 0.14 | 0.17 | 0.31 | 0.40 | 0.29 | 0.30 | 4.00 | 0.10 |
| -72.32601355 | 18.70332846 | 0.09 | 0.11 | 0.15 | 0.18 | 0.33 | 0.38 | 0.26 | 0.30 | 4.00 | 0.32 |
| -72.57448756 | 19.0097438 | 0.03 | 0.04 | 0.09 | 0.10 | 0.28 | 0.31 | 0.20 | 0.30 | 4.00 | 0.33 |
| -72.24651265 | 18.92700896 | 0.03 | 0.05 | 0.09 | 0.11 | 0.38 | 0.26 | 0.16 | 0.30 | 4.00 | 0.43 |
| -72.0996381 | 19.19704254 | 0.02 | 0.04 | 0.07 | 0.08 | 0.23 | 0.24 | 0.14 | 0.30 | 4.00 | 0.20 |
| -72.18641536 | 18.97875192 | 0.04 | 0.05 | 0.09 | 0.11 | 0.31 | 0.34 | 0.21 | 0.30 | 4.00 | 0.36 |
| -72.57098413 | 19.00677936 | 0.04 | 0.06 | 0.11 | 0.11 | 0.30 | 0.33 | 0.21 | 0.30 | 4.00 | 0.40 |
| -72.06918521 | 18.69174019 | 0.05 | 0.07 | 0.12 | 0.14 | 0.38 | 0.38 | 0.24 | 0.30 | 4.00 | 0.06 |
| -72.57583504 | 19.01136077 | 0.03 | 0.05 | 0.08 | 0.09 | 0.21 | 0.28 | 0.19 | 0.30 | 4.00 | 0.55 |
| -71.89239677 | 19.27223153 | 0.03 | 0.04 | 0.07 | 0.09 | 0.27 | 0.26 | 0.16 | 0.30 | 4.00 | 0.09 |
| -72.23115146 | 18.41065734 | 0.03 | 0.05 | 0.09 | 0.11 | 0.20 | 0.22 | 0.16 | 0.31 | 4.00 | 0.33 |
| -72.18506789 | 18.98063839 | 0.04 | 0.06 | 0.10 | 0.14 | 0.36 | 0.37 | 0.23 | 0.30 | 4.00 | 0.26 |
| -72.06810724 | 18.68985373 | 0.05 | 0.06 | 0.11 | 0.12 | 0.33 | 0.36 | 0.22 | 0.30 | 4.00 | 0.00 |
| -72.57799099 | 18.85855734 | 0.08 | 0.10 | 0.14 | 0.16 | 0.37 | 0.34 | 0.22 | 0.30 | 4.00 | 0.11 |
| -72.27804352 | 18.32118514 | 0.03 | 0.05 | 0.10 | 0.13 | 0.28 | 0.28 | 0.19 | 0.30 | 4.00 | 0.15 |
| -73.12802944 | 19.66838857 | 0.09 | 0.10 | 0.13 | 0.15 | 0.26 | 0.36 | 0.23 | 0.30 | 4.00 | 0.45 |
| -72.06595128 | 18.69227918 | 0.06 | 0.08 | 0.12 | 0.13 | 0.35 | 0.37 | 0.24 | 0.30 | 4.00 | 0.01 |
| -71.89239677 | 19.27465698 | 0.04 | 0.05 | 0.09 | 0.11 | 0.30 | 0.32 | 0.21 | 0.30 | 4.00 | 0.36 |
| -72.57152312 | 19.00866582 | 0.05 | 0.07 | 0.12 | 0.14 | 0.36 | 0.39 | 0.25 | 0.30 | 4.00 | 0.42 |
| -73.19001319 | 19.65329687 | 0.06 | 0.08 | 0.12 | 0.15 | 0.25 | 0.34 | 0.23 | 0.30 | 4.00 | 0.33 |
| -72.09748215 | 19.19973748 | 0.03 | 0.04 | 0.08 | 0.08 | 0.25 | 0.26 | 0.14 | 0.30 | 4.00 | 0.55 |
| -72.57314009 | 19.00677936 | 0.05 | 0.06 | 0.11 | 0.12 | 0.29 | 0.37 | 0.24 | 0.30 | 4.00 | 0.68 |
| -72.21767673 | 18.40957936 | 0.03 | 0.05 | 0.09 | 0.11 | 0.19 | 0.22 | 0.15 | 0.30 | 4.00 | 0.63 |
| -72.0999076 | 19.19785102 | 0.03 | 0.04 | 0.08 | 0.09 | 0.25 | 0.26 | 0.14 | 0.30 | 4.00 | 0.50 |
| -72.57987745 | 18.8555929 | 0.09 | 0.11 | 0.16 | 0.18 | 0.39 | 0.38 | 0.24 | 0.30 | 4.00 | 0.32 |
| -71.89158828 | 19.27250102 | 0.03 | 0.05 | 0.08 | 0.10 | 0.27 | 0.30 | 0.19 | 0.30 | 4.00 | 0.63 |
| -72.57556554 | 19.01324723 | 0.05 | 0.07 | 0.11 | 0.11 | 0.26 | 0.31 | 0.22 | 0.30 | 4.00 | 0.37 |
| -72.57071464 | 19.00785734 | 0.06 | 0.08 | 0.13 | 0.14 | 0.28 | 0.37 | 0.27 | 0.30 | 4.00 | 0.38 |
| -72.18587637 | 18.97794344 | 0.05 | 0.06 | 0.10 | 0.13 | 0.34 | 0.34 | 0.22 | 0.30 | 4.00 | 0.65 |
| -72.06945471 | 18.6914707 | 0.06 | 0.08 | 0.13 | 0.14 | 0.37 | 0.39 | 0.25 | 0.30 | 4.00 | 0.56 |
| -72.57960796 | 18.85451492 | 0.09 | 0.10 | 0.15 | 0.18 | 0.38 | 0.39 | 0.24 | 0.30 | 4.00 | 0.45 |
| -72.18641536 | 18.97821293 | 0.04 | 0.06 | 0.09 | 0.12 | 0.33 | 0.36 | 0.22 | 0.30 | 4.00 | 0.67 |
| -72.24651265 | 18.92916492 | 0.03 | 0.04 | 0.07 | 0.09 | 0.29 | 0.22 | 0.12 | 0.30 | 4.00 | 0.11 |
| -72.09882962 | 19.19919849 | 0.03 | 0.04 | 0.08 | 0.09 | 0.24 | 0.26 | 0.15 | 0.30 | 4.00 | 0.40 |
| -72.06729875 | 18.69039272 | 0.06 | 0.07 | 0.12 | 0.13 | 0.34 | 0.37 | 0.24 | 0.30 | 4.00 | 0.70 |
| -72.54214821 | 18.8952086 | 0.05 | 0.07 | 0.11 | 0.13 | 0.36 | 0.38 | 0.23 | 0.30 | 4.00 | 0.34 |
| -73.1897437 | 19.65626131 | 0.06 | 0.08 | 0.11 | 0.13 | 0.26 | 0.35 | 0.22 | 0.30 | 4.00 | 0.00 |
| -73.98313576 | 18.33816329 | 0.02 | 0.04 | 0.08 | 0.09 | 0.36 | 0.26 | 0.15 | 0.30 | 4.00 | 0.37 |
| -72.06837673 | 18.69254867 | 0.06 | 0.07 | 0.11 | 0.12 | 0.34 | 0.34 | 0.23 | 0.30 | 4.00 | 0.06 |
| -71.80373305 | 19.41587214 | 0.03 | 0.03 | 0.06 | 0.06 | 0.21 | 0.25 | 0.15 | 0.30 | 4.00 | 0.30 |
| -72.57394857 | 19.0148642 | 0.05 | 0.06 | 0.10 | 0.10 | 0.27 | 0.29 | 0.20 | 0.30 | 4.00 | 0.65 |
| -72.98950922 | 19.85326185 | 0.08 | 0.10 | 0.16 | 0.19 | 0.30 | 0.43 | 0.35 | 0.31 | 4.00 | 0.15 |
| -72.57152312 | 19.00947431 | 0.05 | 0.07 | 0.13 | 0.14 | 0.37 | 0.40 | 0.25 | 0.30 | 4.00 | 0.03 |
| -72.278852 | 18.32010716 | 0.03 | 0.05 | 0.10 | 0.13 | 0.26 | 0.26 | 0.18 | 0.30 | 4.00 | 0.07 |
| -72.1845289 | 18.97929091 | 0.04 | 0.05 | 0.08 | 0.09 | 0.31 | 0.31 | 0.19 | 0.30 | 4.00 | 0.04 |
| -72.57071464 | 19.00812683 | 0.06 | 0.07 | 0.11 | 0.12 | 0.28 | 0.34 | 0.24 | 0.30 | 4.00 | 0.60 |
| -71.80292456 | 19.41317719 | 0.02 | 0.03 | 0.05 | 0.05 | 0.20 | 0.20 | 0.12 | 0.30 | 4.00 | 0.15 |
| -72.21579027 | 18.40850138 | 0.04 | 0.05 | 0.10 | 0.12 | 0.20 | 0.25 | 0.17 | 0.31 | 4.00 | 0.13 |
| -72.99112619 | 19.85380084 | 0.08 | 0.10 | 0.17 | 0.20 | 0.31 | 0.44 | 0.36 | 0.31 | 4.00 | 0.08 |
| -72.54322619 | 18.89493911 | 0.05 | 0.07 | 0.12 | 0.14 | 0.38 | 0.36 | 0.22 | 0.30 | 4.00 | 0.47 |
| -72.38072096 | 18.73135589 | 0.09 | 0.11 | 0.15 | 0.18 | 0.37 | 0.38 | 0.25 | 0.30 | 4.00 | 0.25 |
| -72.57367908 | 19.01109127 | 0.06 | 0.09 | 0.13 | 0.16 | 0.34 | 0.39 | 0.25 | 0.30 | 4.00 | 0.00 |
| -72.24112276 | 18.29908658 | 0.03 | 0.04 | 0.08 | 0.09 | 0.22 | 0.24 | 0.17 | 0.30 | 4.00 | 0.54 |
| -72.57071464 | 19.00650987 | 0.04 | 0.05 | 0.10 | 0.10 | 0.29 | 0.34 | 0.22 | 0.30 | 4.00 | 0.18 |
| -72.57987745 | 18.85586239 | 0.09 | 0.11 | 0.16 | 0.18 | 0.39 | 0.37 | 0.24 | 0.30 | 4.00 | 0.16 |
| -73.17034009 | 19.73279777 | 0.07 | 0.09 | 0.13 | 0.15 | 0.32 | 0.38 | 0.25 | 0.30 | 4.00 | 0.60 |
| -72.10152457 | 18.70737088 | 0.06 | 0.08 | 0.13 | 0.15 | 0.36 | 0.40 | 0.26 | 0.30 | 4.00 | 0.16 |
| -72.23195994 | 18.41308279 | 0.03 | 0.05 | 0.09 | 0.11 | 0.20 | 0.21 | 0.15 | 0.31 | 4.00 | 0.47 |
| -72.21659875 | 18.40930986 | 0.03 | 0.05 | 0.08 | 0.10 | 0.19 | 0.23 | 0.16 | 0.31 | 4.00 | 0.03 |
| -72.23303792 | 18.41308279 | 0.03 | 0.05 | 0.09 | 0.12 | 0.19 | 0.22 | 0.16 | 0.31 | 4.00 | 0.40 |
| -72.24786012 | 18.9302429 | 0.03 | 0.04 | 0.08 | 0.08 | 0.35 | 0.24 | 0.13 | 0.30 | 4.00 | 0.16 |
| -71.82987402 | 19.20701384 | 0.04 | 0.06 | 0.11 | 0.12 | 0.26 | 0.31 | 0.21 | 0.30 | 4.00 | 0.22 |
| -72.57987745 | 18.8555929 | 0.09 | 0.11 | 0.16 | 0.18 | 0.39 | 0.38 | 0.24 | 0.30 | 4.00 | 0.58 |
| -72.10260254 | 18.70790987 | 0.06 | 0.07 | 0.11 | 0.13 | 0.33 | 0.34 | 0.23 | 0.30 | 4.00 | 0.39 |
| -72.27831301 | 18.32091564 | 0.04 | 0.05 | 0.11 | 0.14 | 0.28 | 0.30 | 0.20 | 0.30 | 4.00 | 0.68 |
| -72.51088684 | 18.83187737 | 0.07 | 0.09 | 0.14 | 0.16 | 0.30 | 0.32 | 0.22 | 0.30 | 4.00 | 0.37 |
| -73.18893522 | 19.65383586 | 0.06 | 0.07 | 0.11 | 0.13 | 0.25 | 0.34 | 0.22 | 0.30 | 4.00 | 0.16 |
| -71.80265507 | 19.41506366 | 0.03 | 0.03 | 0.06 | 0.06 | 0.24 | 0.26 | 0.15 | 0.30 | 4.00 | 0.50 |
| -72.2144428 | 18.41011835 | 0.04 | 0.06 | 0.11 | 0.14 | 0.22 | 0.30 | 0.20 | 0.31 | 4.00 | 0.53 |
| -72.10125507 | 19.1986595 | 0.03 | 0.04 | 0.08 | 0.09 | 0.25 | 0.28 | 0.15 | 0.30 | 4.00 | 0.43 |
| -72.5726011 | 19.01513369 | 0.05 | 0.07 | 0.10 | 0.12 | 0.31 | 0.33 | 0.23 | 0.30 | 4.00 | 0.42 |
| -73.19001319 | 19.65599182 | 0.05 | 0.08 | 0.11 | 0.13 | 0.25 | 0.34 | 0.22 | 0.30 | 4.00 | 0.01 |
| -72.57556554 | 19.01405571 | 0.05 | 0.07 | 0.12 | 0.13 | 0.27 | 0.33 | 0.22 | 0.30 | 4.00 | 0.53 |
| -72.06649027 | 18.69174019 | 0.07 | 0.08 | 0.11 | 0.13 | 0.31 | 0.35 | 0.24 | 0.30 | 4.00 | 0.25 |
| -72.0648733 | 18.69174019 | 0.06 | 0.08 | 0.13 | 0.15 | 0.35 | 0.39 | 0.26 | 0.30 | 4.00 | 0.64 |
| -72.229265 | 18.4125438 | 0.03 | 0.05 | 0.08 | 0.11 | 0.19 | 0.21 | 0.15 | 0.31 | 4.00 | 0.35 |
| -73.9165706 | 18.32846149 | 0.05 | 0.06 | 0.11 | 0.12 | 0.35 | 0.33 | 0.20 | 0.30 | 4.00 | 0.57 |
| -72.57421807 | 19.01297774 | 0.06 | 0.08 | 0.12 | 0.14 | 0.30 | 0.35 | 0.25 | 0.30 | 4.00 | 0.16 |
| -72.07161067 | 18.68985373 | 0.06 | 0.08 | 0.12 | 0.13 | 0.37 | 0.38 | 0.25 | 0.30 | 4.00 | 0.19 |
| -72.57664352 | 18.85667088 | 0.08 | 0.10 | 0.14 | 0.16 | 0.36 | 0.40 | 0.25 | 0.30 | 4.00 | 0.52 |
| -72.57367908 | 19.00731835 | 0.05 | 0.07 | 0.11 | 0.13 | 0.28 | 0.37 | 0.23 | 0.30 | 4.00 | 0.27 |
| -72.57394857 | 19.00839633 | 0.05 | 0.06 | 0.10 | 0.12 | 0.26 | 0.36 | 0.25 | 0.30 | 4.00 | 0.61 |
| -72.06595128 | 18.6914707 | 0.06 | 0.07 | 0.11 | 0.13 | 0.32 | 0.36 | 0.23 | 0.30 | 4.00 | 0.26 |
| -72.37964298 | 18.72973893 | 0.08 | 0.09 | 0.13 | 0.16 | 0.34 | 0.35 | 0.23 | 0.30 | 4.00 | 0.11 |
| -71.99318774 | 18.51306528 | 0.07 | 0.09 | 0.12 | 0.15 | 0.30 | 0.34 | 0.24 | 0.30 | 4.00 | 0.41 |
| -72.18776283 | 18.97902142 | 0.04 | 0.05 | 0.09 | 0.10 | 0.30 | 0.33 | 0.20 | 0.30 | 4.00 | 0.00 |
| -72.21525128 | 18.41065734 | 0.04 | 0.06 | 0.10 | 0.13 | 0.22 | 0.27 | 0.18 | 0.31 | 4.00 | 0.60 |
| -72.23061247 | 18.41065734 | 0.04 | 0.05 | 0.09 | 0.12 | 0.21 | 0.25 | 0.17 | 0.31 | 4.00 | 0.01 |
| -72.99247366 | 19.85353135 | 0.06 | 0.08 | 0.13 | 0.15 | 0.33 | 0.38 | 0.28 | 0.31 | 4.00 | 0.26 |
| -72.10044659 | 19.19785102 | 0.03 | 0.04 | 0.08 | 0.09 | 0.26 | 0.28 | 0.16 | 0.30 | 4.00 | 0.02 |
| -72.09829063 | 19.19839001 | 0.02 | 0.04 | 0.07 | 0.08 | 0.21 | 0.24 | 0.13 | 0.30 | 4.00 | 0.50 |
| -72.18587637 | 18.9800994 | 0.04 | 0.06 | 0.10 | 0.12 | 0.34 | 0.36 | 0.22 | 0.30 | 4.00 | 0.22 |
| -72.15057258 | 18.70925734 | 0.04 | 0.05 | 0.09 | 0.10 | 0.34 | 0.32 | 0.19 | 0.30 | 4.00 | 0.47 |
| -72.18560688 | 18.97848243 | 0.05 | 0.06 | 0.10 | 0.14 | 0.35 | 0.38 | 0.23 | 0.30 | 4.00 | 0.27 |
| -72.57152312 | 19.00866582 | 0.05 | 0.07 | 0.12 | 0.14 | 0.36 | 0.39 | 0.25 | 0.30 | 4.00 | 0.26 |
| -72.98923973 | 19.85218387 | 0.08 | 0.10 | 0.16 | 0.19 | 0.30 | 0.42 | 0.33 | 0.30 | 4.00 | 0.59 |
| -72.23007348 | 18.41065734 | 0.03 | 0.04 | 0.08 | 0.10 | 0.19 | 0.21 | 0.15 | 0.31 | 4.00 | 0.69 |
| -72.51061735 | 18.83241636 | 0.06 | 0.08 | 0.13 | 0.15 | 0.31 | 0.32 | 0.20 | 0.30 | 4.00 | 0.52 |
| -72.35673594 | 18.4076929 | 0.04 | 0.05 | 0.09 | 0.10 | 0.23 | 0.26 | 0.16 | 0.30 | 4.00 | 0.31 |
| -72.18506789 | 18.98063839 | 0.04 | 0.06 | 0.10 | 0.14 | 0.36 | 0.37 | 0.23 | 0.30 | 4.00 | 0.16 |
| -72.32709153 | 18.70278947 | 0.10 | 0.12 | 0.16 | 0.19 | 0.35 | 0.40 | 0.27 | 0.30 | 4.00 | 0.60 |
| -72.57071464 | 19.00947431 | 0.05 | 0.07 | 0.12 | 0.13 | 0.32 | 0.37 | 0.25 | 0.30 | 4.00 | 0.30 |
| -71.89158828 | 19.27061456 | 0.04 | 0.05 | 0.09 | 0.12 | 0.30 | 0.34 | 0.22 | 0.30 | 4.00 | 0.12 |
| -71.8907798 | 19.27061456 | 0.04 | 0.05 | 0.09 | 0.11 | 0.27 | 0.31 | 0.21 | 0.30 | 4.00 | 0.45 |
| -72.23115146 | 18.4125438 | 0.03 | 0.05 | 0.09 | 0.11 | 0.24 | 0.23 | 0.16 | 0.31 | 4.00 | 0.24 |
| -72.06729875 | 18.69012322 | 0.06 | 0.07 | 0.12 | 0.14 | 0.35 | 0.38 | 0.24 | 0.30 | 4.00 | 0.30 |
| -72.06702926 | 18.69039272 | 0.05 | 0.07 | 0.12 | 0.13 | 0.34 | 0.36 | 0.23 | 0.30 | 4.00 | 0.53 |
| -71.89131879 | 19.27061456 | 0.04 | 0.05 | 0.09 | 0.11 | 0.30 | 0.31 | 0.22 | 0.30 | 4.00 | 0.30 |
| -72.36967168 | 18.42278459 | 0.03 | 0.05 | 0.08 | 0.11 | 0.30 | 0.35 | 0.23 | 0.30 | 4.00 | 0.66 |
| -72.21552077 | 18.40930986 | 0.04 | 0.06 | 0.10 | 0.13 | 0.21 | 0.26 | 0.17 | 0.31 | 4.00 | 0.21 |
| -72.32574406 | 18.70305896 | 0.10 | 0.12 | 0.16 | 0.19 | 0.33 | 0.38 | 0.27 | 0.30 | 4.00 | 0.25 |
| -74.12003901 | 18.63918875 | 0.02 | 0.03 | 0.07 | 0.07 | 0.30 | 0.17 | 0.10 | 0.30 | 5.00 | 0.09 |
| -72.20258503 | 19.76028622 | 0.06 | 0.07 | 0.11 | 0.13 | 0.18 | 0.21 | 0.17 | 0.30 | 5.00 | 0.21 |
| -73.09299514 | 18.4424577 | 0.08 | 0.09 | 0.13 | 0.14 | 0.28 | 0.25 | 0.18 | 0.30 | 5.00 | 0.63 |
| -73.7621502 | 18.21689073 | 0.05 | 0.08 | 0.14 | 0.14 | 0.32 | 0.27 | 0.20 | 0.30 | 5.00 | 0.61 |
| -72.68120742 | 19.43931817 | 0.10 | 0.13 | 0.18 | 0.20 | 0.27 | 0.26 | 0.21 | 0.30 | 5.00 | 0.34 |
| -72.21848522 | 19.73603171 | 0.06 | 0.08 | 0.13 | 0.14 | 0.20 | 0.22 | 0.18 | 0.30 | 5.00 | 0.42 |
| -72.82754298 | 19.93384073 | 0.07 | 0.09 | 0.13 | 0.15 | 0.23 | 0.26 | 0.20 | 0.30 | 5.00 | 0.01 |
| -73.75514334 | 18.18913279 | 0.05 | 0.08 | 0.12 | 0.13 | 0.21 | 0.22 | 0.18 | 0.30 | 5.00 | 0.21 |
| -72.25594496 | 18.53031293 | 0.09 | 0.11 | 0.16 | 0.17 | 0.33 | 0.28 | 0.22 | 0.30 | 5.00 | 0.28 |
| -72.22387511 | 19.72983333 | 0.06 | 0.08 | 0.12 | 0.13 | 0.22 | 0.23 | 0.18 | 0.30 | 5.00 | 0.44 |
| -71.83229947 | 19.65491384 | 0.05 | 0.07 | 0.11 | 0.13 | 0.22 | 0.23 | 0.18 | 0.30 | 5.00 | 0.62 |
| -72.84290417 | 19.93437972 | 0.08 | 0.10 | 0.15 | 0.17 | 0.25 | 0.29 | 0.25 | 0.30 | 5.00 | 0.40 |
| -72.68120742 | 19.43904867 | 0.12 | 0.15 | 0.19 | 0.22 | 0.28 | 0.27 | 0.22 | 0.30 | 5.00 | 0.65 |
| -72.21471229 | 18.56912015 | 0.07 | 0.10 | 0.14 | 0.15 | 0.27 | 0.25 | 0.19 | 0.30 | 5.00 | 0.45 |
| -73.76161121 | 18.21554326 | 0.07 | 0.09 | 0.14 | 0.15 | 0.32 | 0.29 | 0.22 | 0.30 | 5.00 | 0.58 |
| -72.82080561 | 19.94515951 | 0.06 | 0.09 | 0.14 | 0.15 | 0.26 | 0.26 | 0.20 | 0.30 | 5.00 | 0.43 |
| -72.22387511 | 19.72875535 | 0.06 | 0.07 | 0.11 | 0.13 | 0.21 | 0.23 | 0.19 | 0.30 | 5.00 | 0.08 |
| -73.76268919 | 18.21635174 | 0.10 | 0.13 | 0.19 | 0.21 | 0.30 | 0.31 | 0.27 | 0.30 | 5.00 | 0.13 |
| -72.34218323 | 18.54998604 | 0.08 | 0.10 | 0.13 | 0.14 | 0.25 | 0.23 | 0.17 | 0.30 | 5.00 | 0.19 |
| -72.21794623 | 19.73495373 | 0.07 | 0.09 | 0.13 | 0.15 | 0.19 | 0.24 | 0.21 | 0.30 | 5.00 | 0.63 |
| -72.22576157 | 19.72929434 | 0.04 | 0.05 | 0.09 | 0.10 | 0.24 | 0.19 | 0.15 | 0.30 | 5.00 | 0.30 |
| -72.63620182 | 18.51037033 | 0.05 | 0.06 | 0.10 | 0.10 | 0.20 | 0.20 | 0.15 | 0.30 | 5.00 | 0.23 |
| -73.74436356 | 18.19640914 | 0.07 | 0.09 | 0.13 | 0.15 | 0.23 | 0.24 | 0.20 | 0.30 | 5.00 | 0.54 |
| -72.70141951 | 19.09813802 | 0.06 | 0.08 | 0.13 | 0.14 | 0.24 | 0.23 | 0.18 | 0.30 | 5.00 | 0.57 |
| -72.21848522 | 18.5168382 | 0.07 | 0.10 | 0.16 | 0.17 | 0.31 | 0.28 | 0.21 | 0.30 | 5.00 | 0.68 |
| -73.74625002 | 18.19452268 | 0.08 | 0.11 | 0.15 | 0.16 | 0.23 | 0.26 | 0.21 | 0.30 | 5.00 | 0.70 |
| -72.18991879 | 19.72767738 | 0.06 | 0.08 | 0.11 | 0.12 | 0.20 | 0.22 | 0.18 | 0.30 | 5.00 | 0.17 |
| -72.20096807 | 19.75947774 | 0.07 | 0.09 | 0.13 | 0.15 | 0.19 | 0.22 | 0.18 | 0.30 | 5.00 | 0.42 |
| -72.34245272 | 18.54890806 | 0.08 | 0.10 | 0.13 | 0.14 | 0.29 | 0.23 | 0.16 | 0.30 | 5.00 | 0.59 |
| -72.82781247 | 19.93384073 | 0.07 | 0.10 | 0.14 | 0.15 | 0.22 | 0.27 | 0.23 | 0.30 | 5.00 | 0.00 |
| -72.29205724 | 18.51063983 | 0.10 | 0.13 | 0.17 | 0.18 | 0.22 | 0.26 | 0.23 | 0.30 | 5.00 | 0.02 |
| -72.12362312 | 19.66946655 | 0.03 | 0.05 | 0.10 | 0.12 | 0.22 | 0.22 | 0.17 | 0.30 | 5.00 | 0.56 |
| -72.70249749 | 19.09732954 | 0.06 | 0.08 | 0.12 | 0.13 | 0.26 | 0.24 | 0.18 | 0.30 | 5.00 | 0.35 |
| -72.22953449 | 18.52276709 | 0.08 | 0.11 | 0.16 | 0.17 | 0.33 | 0.30 | 0.23 | 0.30 | 5.00 | 0.13 |
| -72.20312402 | 19.75893875 | 0.07 | 0.09 | 0.12 | 0.14 | 0.18 | 0.22 | 0.19 | 0.30 | 5.00 | 0.05 |
| -72.48151193 | 19.12508748 | 0.05 | 0.07 | 0.11 | 0.13 | 0.25 | 0.24 | 0.18 | 0.30 | 5.00 | 0.45 |
| -72.12335363 | 19.67027503 | 0.05 | 0.07 | 0.11 | 0.12 | 0.20 | 0.20 | 0.16 | 0.30 | 5.00 | 0.24 |
| -73.76134172 | 18.21527376 | 0.04 | 0.05 | 0.10 | 0.11 | 0.34 | 0.26 | 0.18 | 0.30 | 5.00 | 0.30 |
| -72.34406969 | 18.54863857 | 0.12 | 0.13 | 0.17 | 0.19 | 0.24 | 0.24 | 0.21 | 0.30 | 5.00 | 0.24 |
| -72.61652872 | 19.14826402 | 0.06 | 0.09 | 0.14 | 0.16 | 0.25 | 0.26 | 0.21 | 0.30 | 5.00 | 0.42 |
| -72.29259623 | 18.51037033 | 0.08 | 0.10 | 0.15 | 0.17 | 0.21 | 0.24 | 0.20 | 0.30 | 5.00 | 0.07 |
| -72.54107023 | 19.12643496 | 0.06 | 0.08 | 0.13 | 0.15 | 0.25 | 0.24 | 0.19 | 0.30 | 5.00 | 0.46 |
| -72.6822854 | 19.4425521 | 0.09 | 0.11 | 0.16 | 0.17 | 0.24 | 0.23 | 0.18 | 0.30 | 5.00 | 0.54 |
| -72.69629911 | 19.11296023 | 0.05 | 0.09 | 0.13 | 0.15 | 0.23 | 0.27 | 0.23 | 0.30 | 5.00 | 0.60 |
| -72.25432799 | 18.52896546 | 0.07 | 0.09 | 0.13 | 0.13 | 0.31 | 0.23 | 0.18 | 0.30 | 5.00 | 0.64 |
| -72.18911031 | 19.72740788 | 0.06 | 0.08 | 0.11 | 0.12 | 0.19 | 0.22 | 0.18 | 0.30 | 5.00 | 0.49 |
| -72.29475218 | 18.51063983 | 0.08 | 0.11 | 0.16 | 0.18 | 0.22 | 0.25 | 0.21 | 0.30 | 5.00 | 0.55 |
| -73.74625002 | 18.19479217 | 0.08 | 0.11 | 0.15 | 0.17 | 0.25 | 0.26 | 0.21 | 0.30 | 5.00 | 0.66 |
| -72.71354677 | 19.93006781 | 0.08 | 0.10 | 0.13 | 0.15 | 0.19 | 0.21 | 0.17 | 0.30 | 5.00 | 0.59 |
| -72.12308413 | 19.67135301 | 0.04 | 0.06 | 0.10 | 0.12 | 0.21 | 0.22 | 0.17 | 0.30 | 5.00 | 0.38 |
| -72.21686825 | 19.73576221 | 0.07 | 0.09 | 0.13 | 0.15 | 0.20 | 0.23 | 0.20 | 0.30 | 5.00 | 0.60 |
| -73.74436356 | 18.19613965 | 0.05 | 0.07 | 0.12 | 0.13 | 0.23 | 0.23 | 0.18 | 0.30 | 5.00 | 0.43 |
| -73.74436356 | 18.19452268 | 0.06 | 0.08 | 0.12 | 0.13 | 0.19 | 0.21 | 0.18 | 0.30 | 5.00 | 0.22 |
| -72.61572023 | 18.5119873 | 0.05 | 0.06 | 0.11 | 0.10 | 0.33 | 0.24 | 0.16 | 0.30 | 5.00 | 0.69 |
| -73.74409406 | 18.19506167 | 0.04 | 0.06 | 0.10 | 0.10 | 0.25 | 0.20 | 0.16 | 0.30 | 5.00 | 0.50 |
| -72.68093792 | 19.44174362 | 0.09 | 0.12 | 0.17 | 0.19 | 0.24 | 0.24 | 0.20 | 0.30 | 5.00 | 0.17 |
| -72.69495164 | 19.46330319 | 0.12 | 0.15 | 0.21 | 0.24 | 0.31 | 0.31 | 0.24 | 0.30 | 5.00 | 0.44 |
| -72.48097294 | 19.12293153 | 0.03 | 0.05 | 0.09 | 0.10 | 0.22 | 0.20 | 0.15 | 0.30 | 5.00 | 0.11 |
| -73.74651951 | 18.19640914 | 0.04 | 0.07 | 0.10 | 0.12 | 0.19 | 0.21 | 0.17 | 0.30 | 5.00 | 0.17 |
| -72.22387511 | 18.58502033 | 0.07 | 0.10 | 0.15 | 0.16 | 0.30 | 0.27 | 0.22 | 0.30 | 5.00 | 0.03 |
| -72.68363287 | 19.43850969 | 0.10 | 0.12 | 0.16 | 0.20 | 0.23 | 0.26 | 0.22 | 0.30 | 5.00 | 0.68 |
| -72.34218323 | 18.54836907 | 0.10 | 0.12 | 0.15 | 0.17 | 0.22 | 0.23 | 0.19 | 0.30 | 5.00 | 0.32 |
| -73.74463305 | 18.19560066 | 0.09 | 0.11 | 0.15 | 0.16 | 0.23 | 0.24 | 0.20 | 0.30 | 5.00 | 0.41 |
| -72.70034153 | 19.09813802 | 0.06 | 0.09 | 0.14 | 0.17 | 0.25 | 0.27 | 0.23 | 0.30 | 5.00 | 0.49 |
| -72.48124244 | 19.12239254 | 0.05 | 0.07 | 0.11 | 0.12 | 0.26 | 0.22 | 0.17 | 0.30 | 5.00 | 0.28 |
| -72.70115002 | 19.09840752 | 0.05 | 0.08 | 0.14 | 0.15 | 0.25 | 0.27 | 0.23 | 0.30 | 5.00 | 0.55 |
| -72.34245272 | 18.54944705 | 0.09 | 0.10 | 0.13 | 0.15 | 0.22 | 0.23 | 0.18 | 0.30 | 5.00 | 0.44 |
| -72.68147691 | 19.43958766 | 0.10 | 0.13 | 0.17 | 0.20 | 0.26 | 0.28 | 0.22 | 0.30 | 5.00 | 0.24 |
| -72.84209569 | 19.93626618 | 0.08 | 0.11 | 0.15 | 0.17 | 0.25 | 0.28 | 0.24 | 0.30 | 5.00 | 0.61 |
| -72.69602962 | 19.11242124 | 0.06 | 0.09 | 0.15 | 0.16 | 0.26 | 0.27 | 0.23 | 0.30 | 5.00 | 0.11 |
| -72.85476193 | 18.43140842 | 0.05 | 0.07 | 0.10 | 0.10 | 0.24 | 0.19 | 0.14 | 0.30 | 5.00 | 0.41 |
| -72.25486698 | 18.52950445 | 0.08 | 0.10 | 0.14 | 0.16 | 0.32 | 0.28 | 0.22 | 0.30 | 5.00 | 0.12 |
| -72.7019585 | 19.09867701 | 0.06 | 0.09 | 0.14 | 0.16 | 0.27 | 0.28 | 0.23 | 0.30 | 5.00 | 0.50 |
| -72.48205092 | 19.12374001 | 0.03 | 0.05 | 0.08 | 0.09 | 0.23 | 0.19 | 0.13 | 0.30 | 5.00 | 0.03 |
| -72.20231554 | 19.76055572 | 0.07 | 0.08 | 0.11 | 0.13 | 0.19 | 0.20 | 0.16 | 0.30 | 5.00 | 0.49 |
| -72.19072727 | 19.72902485 | 0.06 | 0.08 | 0.13 | 0.14 | 0.21 | 0.24 | 0.19 | 0.30 | 5.00 | 0.13 |
| -72.21740724 | 19.7363012 | 0.07 | 0.09 | 0.13 | 0.15 | 0.19 | 0.22 | 0.19 | 0.30 | 5.00 | 0.38 |
| -72.29151825 | 18.51144831 | 0.07 | 0.09 | 0.13 | 0.15 | 0.19 | 0.22 | 0.20 | 0.30 | 5.00 | 0.14 |
| -72.85503143 | 18.43006095 | 0.09 | 0.11 | 0.14 | 0.17 | 0.25 | 0.23 | 0.17 | 0.30 | 5.00 | 0.38 |
| -72.18991879 | 19.72740788 | 0.07 | 0.09 | 0.13 | 0.15 | 0.21 | 0.23 | 0.19 | 0.30 | 5.00 | 0.65 |
| -72.8415567 | 19.93518821 | 0.09 | 0.11 | 0.16 | 0.18 | 0.22 | 0.27 | 0.23 | 0.30 | 5.00 | 0.45 |
| -72.6820159 | 19.43958766 | 0.11 | 0.13 | 0.17 | 0.19 | 0.26 | 0.27 | 0.22 | 0.30 | 5.00 | 0.47 |
| -72.70141951 | 19.09813802 | 0.06 | 0.08 | 0.13 | 0.14 | 0.24 | 0.23 | 0.18 | 0.30 | 5.00 | 0.31 |
| -72.54241771 | 19.12616546 | 0.04 | 0.06 | 0.11 | 0.13 | 0.24 | 0.23 | 0.19 | 0.30 | 5.00 | 0.69 |
| -72.1244316 | 19.67135301 | 0.06 | 0.07 | 0.10 | 0.12 | 0.22 | 0.21 | 0.17 | 0.30 | 5.00 | 0.44 |
| -72.21767673 | 19.73603171 | 0.06 | 0.08 | 0.12 | 0.13 | 0.18 | 0.21 | 0.18 | 0.30 | 5.00 | 0.36 |
| -72.12389262 | 19.66946655 | 0.05 | 0.08 | 0.12 | 0.13 | 0.23 | 0.22 | 0.18 | 0.30 | 5.00 | 0.09 |
| -72.34272222 | 18.54971654 | 0.11 | 0.13 | 0.17 | 0.19 | 0.27 | 0.27 | 0.21 | 0.30 | 5.00 | 0.02 |
| -72.63674081 | 18.51063983 | 0.05 | 0.07 | 0.11 | 0.12 | 0.28 | 0.24 | 0.18 | 0.30 | 5.00 | 0.22 |
| -73.76134172 | 18.21635174 | 0.04 | 0.06 | 0.10 | 0.10 | 0.33 | 0.23 | 0.17 | 0.30 | 5.00 | 0.12 |
| -72.34299171 | 18.54756059 | 0.09 | 0.11 | 0.14 | 0.15 | 0.22 | 0.21 | 0.16 | 0.30 | 5.00 | 0.34 |
| -73.09272565 | 18.4421882 | 0.11 | 0.13 | 0.17 | 0.18 | 0.29 | 0.28 | 0.23 | 0.30 | 5.00 | 0.31 |
| -73.76053323 | 18.21527376 | 0.08 | 0.11 | 0.16 | 0.17 | 0.36 | 0.30 | 0.21 | 0.30 | 5.00 | 0.34 |
| -71.83176049 | 19.65599182 | 0.06 | 0.08 | 0.12 | 0.14 | 0.23 | 0.25 | 0.20 | 0.30 | 5.00 | 0.04 |
| -73.74625002 | 18.19479217 | 0.08 | 0.11 | 0.15 | 0.17 | 0.25 | 0.26 | 0.21 | 0.30 | 5.00 | 0.33 |
| -72.19072727 | 19.72875535 | 0.06 | 0.08 | 0.12 | 0.14 | 0.21 | 0.23 | 0.19 | 0.30 | 5.00 | 0.58 |
| -72.54241771 | 19.12535698 | 0.06 | 0.08 | 0.13 | 0.15 | 0.28 | 0.26 | 0.20 | 0.30 | 5.00 | 0.15 |
| -72.84236518 | 19.93599669 | 0.09 | 0.11 | 0.16 | 0.18 | 0.25 | 0.27 | 0.22 | 0.30 | 5.00 | 0.10 |
| -72.1244316 | 19.67054452 | 0.05 | 0.07 | 0.11 | 0.12 | 0.21 | 0.21 | 0.16 | 0.30 | 5.00 | 0.47 |
| -72.29448269 | 18.51090932 | 0.07 | 0.09 | 0.13 | 0.15 | 0.19 | 0.21 | 0.17 | 0.30 | 5.00 | 0.43 |
| -72.54187872 | 19.12589597 | 0.09 | 0.12 | 0.16 | 0.18 | 0.25 | 0.26 | 0.23 | 0.30 | 5.00 | 0.63 |
| -73.7618807 | 18.21608225 | 0.07 | 0.10 | 0.14 | 0.16 | 0.29 | 0.29 | 0.24 | 0.30 | 5.00 | 0.53 |
| -72.68120742 | 19.44174362 | 0.10 | 0.12 | 0.17 | 0.19 | 0.24 | 0.25 | 0.21 | 0.30 | 5.00 | 0.49 |
| -74.12003901 | 18.63811077 | 0.01 | 0.02 | 0.06 | 0.05 | 0.33 | 0.18 | 0.10 | 0.30 | 5.00 | 0.01 |
| -72.84236518 | 19.93518821 | 0.07 | 0.10 | 0.15 | 0.17 | 0.26 | 0.30 | 0.24 | 0.30 | 5.00 | 0.34 |
| -73.7470585 | 18.1934447 | 0.05 | 0.07 | 0.12 | 0.13 | 0.24 | 0.23 | 0.17 | 0.30 | 5.00 | 0.63 |
| -72.12389262 | 19.67135301 | 0.04 | 0.06 | 0.09 | 0.11 | 0.18 | 0.20 | 0.16 | 0.30 | 5.00 | 0.41 |
| -72.61652872 | 19.149342 | 0.04 | 0.06 | 0.12 | 0.12 | 0.26 | 0.24 | 0.18 | 0.30 | 5.00 | 0.01 |
| -72.68336337 | 19.44147413 | 0.10 | 0.12 | 0.17 | 0.19 | 0.23 | 0.25 | 0.20 | 0.30 | 5.00 | 0.61 |
| -73.74463305 | 18.19398369 | 0.09 | 0.11 | 0.14 | 0.16 | 0.20 | 0.22 | 0.19 | 0.30 | 5.00 | 0.10 |
| -72.20204605 | 19.75920824 | 0.07 | 0.09 | 0.12 | 0.15 | 0.18 | 0.21 | 0.18 | 0.30 | 5.00 | 0.68 |
| -72.70034153 | 19.09894651 | 0.07 | 0.10 | 0.15 | 0.18 | 0.25 | 0.30 | 0.26 | 0.30 | 5.00 | 0.22 |
| -74.11842204 | 18.63918875 | 0.04 | 0.05 | 0.09 | 0.10 | 0.26 | 0.19 | 0.13 | 0.30 | 5.00 | 0.68 |
| -72.23007348 | 18.52330607 | 0.08 | 0.11 | 0.16 | 0.18 | 0.31 | 0.28 | 0.21 | 0.30 | 5.00 | 0.19 |
| -72.4828594 | 19.12374001 | 0.05 | 0.07 | 0.12 | 0.13 | 0.24 | 0.22 | 0.17 | 0.30 | 5.00 | 0.22 |
| -72.19045778 | 19.72794687 | 0.06 | 0.07 | 0.12 | 0.13 | 0.23 | 0.23 | 0.18 | 0.30 | 5.00 | 0.58 |
| -72.12416211 | 19.66946655 | 0.05 | 0.06 | 0.10 | 0.11 | 0.20 | 0.21 | 0.16 | 0.30 | 5.00 | 0.54 |
| -72.2244141 | 19.72902485 | 0.07 | 0.09 | 0.13 | 0.14 | 0.21 | 0.24 | 0.20 | 0.30 | 5.00 | 0.18 |
| -72.3438002 | 18.54890806 | 0.07 | 0.09 | 0.12 | 0.13 | 0.22 | 0.19 | 0.15 | 0.30 | 5.00 | 0.27 |
| -72.25351951 | 18.53004344 | 0.08 | 0.10 | 0.14 | 0.16 | 0.31 | 0.28 | 0.22 | 0.30 | 5.00 | 0.14 |
| -73.7618807 | 18.21662124 | 0.06 | 0.09 | 0.14 | 0.15 | 0.31 | 0.29 | 0.22 | 0.30 | 5.00 | 0.21 |
| -72.48258991 | 19.1240095 | 0.04 | 0.06 | 0.11 | 0.12 | 0.23 | 0.22 | 0.17 | 0.30 | 5.00 | 0.46 |
| -72.61625922 | 19.14880301 | 0.06 | 0.09 | 0.14 | 0.16 | 0.24 | 0.25 | 0.21 | 0.30 | 5.00 | 0.33 |
| -71.83176049 | 19.65545283 | 0.06 | 0.08 | 0.12 | 0.14 | 0.23 | 0.25 | 0.19 | 0.30 | 5.00 | 0.09 |
| -72.48232041 | 19.12374001 | 0.05 | 0.07 | 0.11 | 0.12 | 0.24 | 0.23 | 0.17 | 0.30 | 5.00 | 0.45 |
| -71.83229947 | 19.65464434 | 0.06 | 0.08 | 0.12 | 0.14 | 0.22 | 0.24 | 0.19 | 0.30 | 5.00 | 0.67 |
| -72.86527222 | 18.43006095 | 0.07 | 0.09 | 0.13 | 0.13 | 0.27 | 0.22 | 0.16 | 0.30 | 5.00 | 0.39 |
| -73.09245615 | 18.44272719 | 0.08 | 0.09 | 0.13 | 0.14 | 0.28 | 0.23 | 0.16 | 0.30 | 5.00 | 0.61 |
| -72.12389262 | 19.67081402 | 0.05 | 0.07 | 0.11 | 0.12 | 0.23 | 0.22 | 0.17 | 0.30 | 5.00 | 0.12 |
| -72.82107511 | 19.945429 | 0.07 | 0.10 | 0.14 | 0.16 | 0.23 | 0.25 | 0.20 | 0.30 | 5.00 | 0.25 |
| -74.120578 | 18.63811077 | 0.02 | 0.04 | 0.08 | 0.07 | 0.33 | 0.19 | 0.11 | 0.30 | 5.00 | 0.16 |
| -72.27103666 | 18.62786997 | 0.09 | 0.11 | 0.15 | 0.17 | 0.22 | 0.24 | 0.20 | 0.30 | 5.00 | 0.19 |
| -72.29475218 | 18.51090932 | 0.07 | 0.09 | 0.13 | 0.16 | 0.22 | 0.22 | 0.19 | 0.30 | 5.00 | 0.06 |
| -72.2936742 | 18.51117882 | 0.09 | 0.12 | 0.17 | 0.18 | 0.23 | 0.25 | 0.22 | 0.30 | 5.00 | 0.47 |
| -72.21632926 | 19.73576221 | 0.10 | 0.12 | 0.16 | 0.17 | 0.21 | 0.26 | 0.23 | 0.30 | 5.00 | 0.17 |
| -72.48258991 | 19.12481799 | 0.10 | 0.12 | 0.17 | 0.20 | 0.27 | 0.30 | 0.25 | 0.30 | 5.00 | 0.17 |
| -72.21848522 | 19.73576221 | 0.05 | 0.07 | 0.11 | 0.12 | 0.21 | 0.21 | 0.16 | 0.30 | 5.00 | 0.45 |
| -72.6820159 | 19.44120463 | 0.10 | 0.13 | 0.17 | 0.20 | 0.24 | 0.26 | 0.22 | 0.30 | 5.00 | 0.06 |
| -72.29151825 | 18.51117882 | 0.08 | 0.10 | 0.14 | 0.16 | 0.20 | 0.24 | 0.21 | 0.30 | 5.00 | 0.47 |
| -72.34326121 | 18.54836907 | 0.10 | 0.12 | 0.14 | 0.16 | 0.22 | 0.24 | 0.21 | 0.30 | 5.00 | 0.13 |
| -72.20258503 | 19.75947774 | 0.08 | 0.10 | 0.13 | 0.15 | 0.19 | 0.24 | 0.21 | 0.30 | 5.00 | 0.36 |
| -72.21686825 | 19.73549272 | 0.07 | 0.09 | 0.14 | 0.15 | 0.20 | 0.24 | 0.20 | 0.30 | 5.00 | 0.44 |
| -72.29232673 | 18.51144831 | 0.12 | 0.15 | 0.20 | 0.22 | 0.25 | 0.31 | 0.28 | 0.30 | 5.00 | 0.34 |
| -72.22387511 | 19.72875535 | 0.06 | 0.07 | 0.11 | 0.13 | 0.21 | 0.23 | 0.19 | 0.30 | 5.00 | 0.14 |
| -71.8312215 | 19.65545283 | 0.07 | 0.08 | 0.13 | 0.15 | 0.23 | 0.24 | 0.19 | 0.30 | 5.00 | 0.13 |
| -72.84182619 | 19.93626618 | 0.08 | 0.10 | 0.15 | 0.17 | 0.24 | 0.26 | 0.22 | 0.30 | 5.00 | 0.59 |
| -72.54133973 | 19.12616546 | 0.04 | 0.07 | 0.12 | 0.13 | 0.24 | 0.23 | 0.17 | 0.30 | 5.00 | 0.55 |
| -72.82781247 | 19.93357124 | 0.05 | 0.07 | 0.11 | 0.13 | 0.26 | 0.24 | 0.18 | 0.30 | 5.00 | 0.27 |
| -72.68066843 | 19.4425521 | 0.08 | 0.10 | 0.15 | 0.16 | 0.24 | 0.22 | 0.17 | 0.30 | 5.00 | 0.26 |
| -72.29313522 | 18.51090932 | 0.08 | 0.11 | 0.16 | 0.18 | 0.23 | 0.26 | 0.22 | 0.30 | 5.00 | 0.40 |
| -73.09191717 | 18.4424577 | 0.11 | 0.13 | 0.16 | 0.18 | 0.26 | 0.26 | 0.20 | 0.30 | 5.00 | 0.17 |
| -72.29259623 | 18.51279579 | 0.08 | 0.10 | 0.15 | 0.16 | 0.22 | 0.24 | 0.21 | 0.30 | 5.00 | 0.68 |
| -74.120578 | 18.63891925 | 0.03 | 0.04 | 0.08 | 0.08 | 0.32 | 0.20 | 0.13 | 0.30 | 5.00 | 0.51 |
| -74.11923052 | 18.63864976 | 0.03 | 0.05 | 0.10 | 0.10 | 0.34 | 0.21 | 0.13 | 0.30 | 5.00 | 0.16 |
| -72.18964929 | 19.72875535 | 0.05 | 0.07 | 0.11 | 0.11 | 0.22 | 0.22 | 0.18 | 0.30 | 5.00 | 0.47 |
| -72.22549207 | 19.72929434 | 0.06 | 0.08 | 0.13 | 0.15 | 0.23 | 0.23 | 0.20 | 0.30 | 5.00 | 0.59 |
| -72.29232673 | 18.51117882 | 0.09 | 0.12 | 0.16 | 0.19 | 0.23 | 0.27 | 0.24 | 0.30 | 5.00 | 0.65 |
| -72.68255489 | 19.43904867 | 0.09 | 0.12 | 0.17 | 0.19 | 0.27 | 0.26 | 0.21 | 0.30 | 5.00 | 0.48 |
| -72.22468359 | 19.72875535 | 0.06 | 0.08 | 0.12 | 0.14 | 0.20 | 0.24 | 0.20 | 0.30 | 5.00 | 0.33 |
| -72.63701031 | 18.51090932 | 0.06 | 0.07 | 0.11 | 0.12 | 0.24 | 0.25 | 0.20 | 0.30 | 5.00 | 0.55 |
| -72.21525128 | 18.56992864 | 0.08 | 0.11 | 0.15 | 0.17 | 0.27 | 0.26 | 0.21 | 0.30 | 5.00 | 0.27 |
| -72.54187872 | 19.12535698 | 0.05 | 0.07 | 0.12 | 0.12 | 0.25 | 0.23 | 0.18 | 0.30 | 5.00 | 0.08 |
| -72.84209569 | 19.93437972 | 0.07 | 0.10 | 0.15 | 0.17 | 0.25 | 0.29 | 0.24 | 0.30 | 5.00 | 0.24 |
| -72.68174641 | 19.44120463 | 0.12 | 0.15 | 0.19 | 0.22 | 0.26 | 0.27 | 0.23 | 0.30 | 5.00 | 0.19 |
| -72.54241771 | 19.12616546 | 0.04 | 0.06 | 0.11 | 0.13 | 0.24 | 0.23 | 0.19 | 0.30 | 5.00 | 0.15 |
| -72.48205092 | 19.12562647 | 0.08 | 0.10 | 0.14 | 0.17 | 0.27 | 0.26 | 0.21 | 0.30 | 5.00 | 0.62 |
| -73.76241969 | 18.21635174 | 0.06 | 0.08 | 0.13 | 0.13 | 0.29 | 0.29 | 0.23 | 0.30 | 5.00 | 0.53 |
| -72.34326121 | 18.54971654 | 0.09 | 0.11 | 0.13 | 0.15 | 0.20 | 0.21 | 0.17 | 0.30 | 5.00 | 0.52 |
| -72.70168901 | 19.09813802 | 0.07 | 0.10 | 0.14 | 0.15 | 0.24 | 0.24 | 0.20 | 0.30 | 5.00 | 0.18 |
| -72.21713774 | 19.73522322 | 0.07 | 0.09 | 0.13 | 0.15 | 0.20 | 0.22 | 0.19 | 0.30 | 5.00 | 0.00 |
| -72.33436789 | 18.54729109 | 0.08 | 0.10 | 0.13 | 0.15 | 0.20 | 0.22 | 0.18 | 0.30 | 5.00 | 0.55 |
| -74.11976951 | 18.63811077 | 0.02 | 0.04 | 0.08 | 0.08 | 0.30 | 0.20 | 0.13 | 0.30 | 5.00 | 0.54 |
| -73.08841374 | 18.44515264 | 0.10 | 0.11 | 0.14 | 0.15 | 0.24 | 0.22 | 0.17 | 0.30 | 5.00 | 0.61 |
| -72.27022818 | 18.62733098 | 0.08 | 0.10 | 0.15 | 0.15 | 0.25 | 0.24 | 0.19 | 0.30 | 5.00 | 0.65 |
| -72.29421319 | 18.51144831 | 0.07 | 0.09 | 0.13 | 0.15 | 0.20 | 0.23 | 0.19 | 0.30 | 5.00 | 0.08 |
| -72.29178774 | 18.51117882 | 0.05 | 0.07 | 0.11 | 0.12 | 0.18 | 0.20 | 0.17 | 0.30 | 5.00 | 0.69 |
| -72.82053612 | 19.945429 | 0.08 | 0.10 | 0.16 | 0.17 | 0.24 | 0.26 | 0.21 | 0.30 | 5.00 | 0.42 |
| -72.69737709 | 19.11296023 | 0.05 | 0.08 | 0.13 | 0.14 | 0.24 | 0.25 | 0.21 | 0.30 | 5.00 | 0.41 |
| -72.70168901 | 19.09759904 | 0.09 | 0.10 | 0.13 | 0.14 | 0.23 | 0.22 | 0.17 | 0.30 | 5.00 | 0.56 |
| -73.74517204 | 18.19317521 | 0.05 | 0.07 | 0.11 | 0.12 | 0.21 | 0.22 | 0.18 | 0.30 | 5.00 | 0.15 |
| -72.27076716 | 18.62733098 | 0.08 | 0.10 | 0.14 | 0.15 | 0.21 | 0.22 | 0.18 | 0.30 | 5.00 | 0.35 |
| -72.85583991 | 18.43033044 | 0.08 | 0.10 | 0.13 | 0.15 | 0.25 | 0.23 | 0.17 | 0.30 | 5.00 | 0.47 |
| -72.68282439 | 19.44066564 | 0.09 | 0.12 | 0.16 | 0.18 | 0.23 | 0.25 | 0.19 | 0.30 | 5.00 | 0.38 |
| -72.29259623 | 18.51063983 | 0.08 | 0.11 | 0.15 | 0.17 | 0.22 | 0.25 | 0.22 | 0.30 | 5.00 | 0.18 |
| -72.84263467 | 19.93680517 | 0.07 | 0.09 | 0.12 | 0.13 | 0.23 | 0.26 | 0.21 | 0.30 | 5.00 | 0.13 |
| -72.68174641 | 19.43958766 | 0.10 | 0.12 | 0.17 | 0.21 | 0.26 | 0.30 | 0.25 | 0.30 | 5.00 | 0.57 |
| -72.12362312 | 19.67027503 | 0.05 | 0.07 | 0.10 | 0.11 | 0.20 | 0.20 | 0.16 | 0.30 | 5.00 | 0.07 |
| -72.1247011 | 19.66973604 | 0.04 | 0.06 | 0.09 | 0.10 | 0.23 | 0.20 | 0.15 | 0.30 | 5.00 | 0.45 |
| -72.12389262 | 19.67000553 | 0.04 | 0.05 | 0.09 | 0.11 | 0.22 | 0.19 | 0.14 | 0.30 | 5.00 | 0.14 |
| -72.20150706 | 19.75893875 | 0.06 | 0.08 | 0.11 | 0.13 | 0.17 | 0.20 | 0.17 | 0.30 | 5.00 | 0.59 |
| -72.68039893 | 19.4425521 | 0.08 | 0.11 | 0.15 | 0.17 | 0.23 | 0.22 | 0.17 | 0.30 | 5.00 | 0.24 |
| -73.74651951 | 18.19667864 | 0.06 | 0.08 | 0.12 | 0.13 | 0.21 | 0.24 | 0.20 | 0.30 | 5.00 | 0.41 |
| -72.29178774 | 18.51171781 | 0.07 | 0.09 | 0.13 | 0.14 | 0.20 | 0.21 | 0.18 | 0.30 | 5.00 | 0.45 |
| -72.18857132 | 19.72740788 | 0.06 | 0.07 | 0.11 | 0.13 | 0.20 | 0.22 | 0.19 | 0.30 | 5.00 | 0.39 |
| -72.20204605 | 19.75974723 | 0.10 | 0.12 | 0.14 | 0.15 | 0.19 | 0.23 | 0.20 | 0.30 | 5.00 | 0.25 |
| -73.74463305 | 18.19613965 | 0.06 | 0.08 | 0.12 | 0.14 | 0.22 | 0.22 | 0.18 | 0.30 | 5.00 | 0.35 |
| -72.20123756 | 19.75947774 | 0.07 | 0.09 | 0.12 | 0.14 | 0.17 | 0.20 | 0.17 | 0.30 | 5.00 | 0.70 |
| -73.74490255 | 18.19587015 | 0.04 | 0.06 | 0.11 | 0.12 | 0.21 | 0.22 | 0.17 | 0.30 | 5.00 | 0.02 |
| -73.76268919 | 18.21662124 | 0.11 | 0.13 | 0.18 | 0.19 | 0.29 | 0.31 | 0.26 | 0.30 | 5.00 | 0.44 |
| -72.20339352 | 19.75974723 | 0.07 | 0.08 | 0.12 | 0.14 | 0.17 | 0.21 | 0.18 | 0.30 | 5.00 | 0.30 |
| -72.84209569 | 19.93464922 | 0.09 | 0.11 | 0.16 | 0.17 | 0.25 | 0.28 | 0.24 | 0.30 | 5.00 | 0.19 |
| -73.74490255 | 18.19613965 | 0.04 | 0.06 | 0.10 | 0.11 | 0.18 | 0.20 | 0.16 | 0.30 | 5.00 | 0.51 |
| -72.22468359 | 18.58475084 | 0.07 | 0.11 | 0.16 | 0.17 | 0.27 | 0.26 | 0.21 | 0.30 | 5.00 | 0.21 |
| -73.74517204 | 18.19452268 | 0.04 | 0.06 | 0.11 | 0.11 | 0.22 | 0.21 | 0.16 | 0.30 | 5.00 | 0.15 |
| -72.48205092 | 19.12347052 | 0.05 | 0.07 | 0.11 | 0.12 | 0.24 | 0.22 | 0.16 | 0.30 | 5.00 | 0.28 |
| -72.84344316 | 19.9354577 | 0.08 | 0.10 | 0.14 | 0.15 | 0.22 | 0.24 | 0.19 | 0.30 | 5.00 | 0.64 |
| -73.76161121 | 18.21635174 | 0.05 | 0.07 | 0.12 | 0.12 | 0.31 | 0.25 | 0.19 | 0.30 | 5.00 | 0.52 |
| -72.1244316 | 19.67135301 | 0.06 | 0.07 | 0.10 | 0.12 | 0.22 | 0.21 | 0.17 | 0.30 | 5.00 | 0.70 |
| -72.68255489 | 19.44228261 | 0.08 | 0.10 | 0.14 | 0.17 | 0.24 | 0.24 | 0.19 | 0.30 | 5.00 | 0.33 |
| -72.20177655 | 19.75920824 | 0.07 | 0.08 | 0.12 | 0.14 | 0.17 | 0.21 | 0.18 | 0.30 | 5.00 | 0.10 |
| -72.68309388 | 19.44228261 | 0.09 | 0.11 | 0.15 | 0.18 | 0.23 | 0.24 | 0.18 | 0.30 | 5.00 | 0.04 |
| -72.29205724 | 18.51063983 | 0.10 | 0.13 | 0.17 | 0.18 | 0.22 | 0.26 | 0.23 | 0.30 | 5.00 | 0.67 |
| -72.21875471 | 19.7363012 | 0.06 | 0.08 | 0.11 | 0.13 | 0.19 | 0.21 | 0.18 | 0.30 | 5.00 | 0.60 |
| -72.48016446 | 19.12374001 | 0.06 | 0.07 | 0.11 | 0.13 | 0.22 | 0.24 | 0.19 | 0.30 | 5.00 | 0.49 |
| -74.12003901 | 18.63945824 | 0.05 | 0.06 | 0.11 | 0.12 | 0.27 | 0.21 | 0.15 | 0.30 | 5.00 | 0.10 |
| -73.74598052 | 18.19506167 | 0.07 | 0.09 | 0.14 | 0.15 | 0.22 | 0.24 | 0.20 | 0.30 | 5.00 | 0.03 |
| -73.74517204 | 18.19398369 | 0.07 | 0.09 | 0.14 | 0.15 | 0.24 | 0.24 | 0.20 | 0.30 | 5.00 | 0.01 |
| -72.70330597 | 19.09652106 | 0.06 | 0.08 | 0.13 | 0.15 | 0.24 | 0.26 | 0.21 | 0.30 | 5.00 | 0.15 |
| -72.82026662 | 19.94596799 | 0.06 | 0.08 | 0.12 | 0.14 | 0.23 | 0.22 | 0.18 | 0.30 | 5.00 | 0.28 |
| -72.70115002 | 19.09706005 | 0.09 | 0.10 | 0.15 | 0.16 | 0.29 | 0.27 | 0.21 | 0.30 | 5.00 | 0.58 |
| -72.29286572 | 18.51252629 | 0.09 | 0.12 | 0.16 | 0.18 | 0.22 | 0.26 | 0.23 | 0.30 | 5.00 | 0.16 |
| -71.83283846 | 19.65599182 | 0.06 | 0.08 | 0.12 | 0.14 | 0.21 | 0.23 | 0.19 | 0.30 | 5.00 | 0.02 |
| -71.83256897 | 19.65572232 | 0.07 | 0.09 | 0.13 | 0.16 | 0.23 | 0.25 | 0.21 | 0.30 | 5.00 | 0.15 |
| -72.3335594 | 18.54648261 | 0.11 | 0.13 | 0.16 | 0.18 | 0.23 | 0.25 | 0.22 | 0.30 | 5.00 | 0.03 |
| -72.34272222 | 18.54783008 | 0.08 | 0.09 | 0.12 | 0.13 | 0.20 | 0.19 | 0.15 | 0.30 | 5.00 | 0.49 |
| -72.63701031 | 18.51063983 | 0.07 | 0.09 | 0.13 | 0.15 | 0.24 | 0.27 | 0.22 | 0.30 | 5.00 | 0.09 |
| -74.11869154 | 18.63945824 | 0.06 | 0.08 | 0.11 | 0.12 | 0.25 | 0.21 | 0.15 | 0.30 | 5.00 | 0.20 |
| -72.33302041 | 18.5467521 | 0.10 | 0.12 | 0.15 | 0.17 | 0.21 | 0.23 | 0.20 | 0.30 | 5.00 | 0.69 |
| -72.22333612 | 19.72902485 | 0.07 | 0.08 | 0.13 | 0.14 | 0.20 | 0.23 | 0.19 | 0.30 | 5.00 | 0.28 |
| -72.21740724 | 19.7365707 | 0.07 | 0.09 | 0.14 | 0.16 | 0.20 | 0.22 | 0.19 | 0.30 | 5.00 | 0.28 |
| -72.84317366 | 19.93626618 | 0.09 | 0.11 | 0.16 | 0.18 | 0.25 | 0.27 | 0.23 | 0.30 | 5.00 | 0.12 |
| -72.2241446 | 19.72929434 | 0.05 | 0.06 | 0.10 | 0.11 | 0.21 | 0.21 | 0.17 | 0.30 | 5.00 | 0.38 |
| -74.12003901 | 18.63999723 | 0.06 | 0.08 | 0.11 | 0.12 | 0.31 | 0.23 | 0.15 | 0.30 | 5.00 | 0.11 |
| -72.6822854 | 19.44093514 | 0.10 | 0.13 | 0.17 | 0.20 | 0.25 | 0.25 | 0.20 | 0.30 | 5.00 | 0.34 |
| -72.21848522 | 19.7363012 | 0.07 | 0.08 | 0.12 | 0.13 | 0.18 | 0.22 | 0.19 | 0.30 | 5.00 | 0.34 |
| -72.69495164 | 19.46303369 | 0.12 | 0.15 | 0.20 | 0.24 | 0.30 | 0.28 | 0.21 | 0.30 | 5.00 | 0.57 |
| -72.61652872 | 19.14853351 | 0.05 | 0.08 | 0.13 | 0.15 | 0.27 | 0.27 | 0.21 | 0.30 | 5.00 | 0.67 |
| -72.2939437 | 18.51117882 | 0.08 | 0.10 | 0.14 | 0.16 | 0.21 | 0.24 | 0.20 | 0.30 | 5.00 | 0.19 |
| -72.48232041 | 19.12535698 | 0.04 | 0.05 | 0.10 | 0.11 | 0.24 | 0.22 | 0.17 | 0.30 | 5.00 | 0.43 |
| -72.20231554 | 19.75893875 | 0.08 | 0.09 | 0.12 | 0.14 | 0.18 | 0.21 | 0.18 | 0.30 | 5.00 | 0.21 |
| -73.75514334 | 18.18913279 | 0.05 | 0.08 | 0.12 | 0.13 | 0.21 | 0.22 | 0.18 | 0.30 | 5.00 | 0.58 |
| -72.26995868 | 18.62760048 | 0.07 | 0.09 | 0.14 | 0.15 | 0.23 | 0.24 | 0.20 | 0.30 | 5.00 | 0.32 |
| -72.7019585 | 19.09679055 | 0.06 | 0.08 | 0.14 | 0.15 | 0.26 | 0.29 | 0.25 | 0.30 | 5.00 | 0.03 |
| -72.3435307 | 18.54971654 | 0.08 | 0.10 | 0.13 | 0.15 | 0.21 | 0.21 | 0.17 | 0.30 | 5.00 | 0.37 |
| -72.29232673 | 18.51117882 | 0.09 | 0.12 | 0.16 | 0.19 | 0.23 | 0.27 | 0.24 | 0.30 | 5.00 | 0.40 |
| -73.74678901 | 18.19587015 | 0.07 | 0.10 | 0.14 | 0.15 | 0.22 | 0.23 | 0.20 | 0.30 | 5.00 | 0.53 |
| -72.21821572 | 19.73522322 | 0.06 | 0.08 | 0.13 | 0.14 | 0.22 | 0.21 | 0.17 | 0.30 | 5.00 | 0.42 |
| -72.68093792 | 19.44201312 | 0.09 | 0.11 | 0.15 | 0.17 | 0.24 | 0.24 | 0.18 | 0.30 | 5.00 | 0.50 |
| -73.74544153 | 18.1937142 | 0.05 | 0.08 | 0.12 | 0.14 | 0.22 | 0.23 | 0.19 | 0.30 | 5.00 | 0.39 |
| -72.70115002 | 19.09894651 | 0.06 | 0.09 | 0.14 | 0.15 | 0.25 | 0.29 | 0.24 | 0.30 | 5.00 | 0.55 |
| -72.48258991 | 19.12347052 | 0.05 | 0.07 | 0.11 | 0.12 | 0.23 | 0.21 | 0.16 | 0.30 | 5.00 | 0.60 |
| -73.7621502 | 18.21635174 | 0.06 | 0.08 | 0.13 | 0.14 | 0.30 | 0.28 | 0.22 | 0.30 | 5.00 | 0.24 |
| -72.21686825 | 19.73522322 | 0.06 | 0.08 | 0.13 | 0.15 | 0.19 | 0.22 | 0.19 | 0.30 | 5.00 | 0.26 |
| -73.74571103 | 18.19640914 | 0.07 | 0.09 | 0.13 | 0.15 | 0.23 | 0.24 | 0.20 | 0.30 | 5.00 | 0.35 |
| -72.85583991 | 18.43059994 | 0.07 | 0.09 | 0.12 | 0.13 | 0.24 | 0.21 | 0.16 | 0.30 | 5.00 | 0.01 |
| -72.68120742 | 19.44012665 | 0.09 | 0.11 | 0.16 | 0.19 | 0.24 | 0.24 | 0.20 | 0.30 | 5.00 | 0.12 |
| -72.21686825 | 19.7365707 | 0.07 | 0.09 | 0.12 | 0.14 | 0.17 | 0.22 | 0.19 | 0.30 | 5.00 | 0.57 |
| -72.21740724 | 19.73710969 | 0.06 | 0.08 | 0.11 | 0.13 | 0.16 | 0.20 | 0.18 | 0.30 | 5.00 | 0.07 |
| -72.85503143 | 18.43059994 | 0.06 | 0.08 | 0.12 | 0.12 | 0.24 | 0.21 | 0.15 | 0.30 | 5.00 | 0.69 |
| -72.6822854 | 19.44012665 | 0.10 | 0.12 | 0.16 | 0.18 | 0.23 | 0.23 | 0.19 | 0.30 | 5.00 | 0.17 |
| -72.20339352 | 19.75920824 | 0.07 | 0.09 | 0.11 | 0.14 | 0.18 | 0.21 | 0.18 | 0.30 | 5.00 | 0.16 |
| -72.12389262 | 19.67135301 | 0.04 | 0.06 | 0.09 | 0.11 | 0.18 | 0.20 | 0.16 | 0.30 | 5.00 | 0.66 |
| -72.70168901 | 19.09786853 | 0.08 | 0.10 | 0.14 | 0.16 | 0.23 | 0.22 | 0.18 | 0.30 | 5.00 | 0.35 |
| -72.6820159 | 19.44066564 | 0.09 | 0.12 | 0.16 | 0.19 | 0.23 | 0.23 | 0.19 | 0.30 | 5.00 | 0.48 |
| -72.68093792 | 19.44066564 | 0.10 | 0.12 | 0.17 | 0.19 | 0.26 | 0.26 | 0.21 | 0.30 | 5.00 | 0.15 |
| -72.25325002 | 18.52977394 | 0.10 | 0.12 | 0.16 | 0.20 | 0.32 | 0.32 | 0.27 | 0.30 | 5.00 | 0.51 |
| -72.70303648 | 19.09679055 | 0.05 | 0.08 | 0.14 | 0.16 | 0.24 | 0.28 | 0.23 | 0.30 | 5.00 | 0.11 |
| -73.75999424 | 18.21554326 | 0.03 | 0.05 | 0.10 | 0.10 | 0.36 | 0.26 | 0.16 | 0.30 | 5.00 | 0.21 |
| -72.61518124 | 18.5119873 | 0.04 | 0.05 | 0.09 | 0.09 | 0.32 | 0.24 | 0.15 | 0.30 | 5.00 | 0.69 |
| -72.84344316 | 19.93599669 | 0.08 | 0.10 | 0.15 | 0.17 | 0.24 | 0.26 | 0.23 | 0.30 | 5.00 | 0.48 |
| -72.29205724 | 18.5122568 | 0.08 | 0.11 | 0.15 | 0.17 | 0.23 | 0.24 | 0.20 | 0.30 | 5.00 | 0.26 |
| -72.6822854 | 19.4428216 | 0.09 | 0.11 | 0.15 | 0.17 | 0.26 | 0.24 | 0.18 | 0.30 | 5.00 | 0.60 |
| -72.20339352 | 19.76028622 | 0.07 | 0.09 | 0.12 | 0.13 | 0.16 | 0.20 | 0.17 | 0.30 | 5.00 | 0.19 |
| -72.29340471 | 18.50983135 | 0.06 | 0.09 | 0.14 | 0.16 | 0.21 | 0.23 | 0.20 | 0.30 | 5.00 | 0.04 |
| -72.34245272 | 18.54998604 | 0.07 | 0.08 | 0.11 | 0.12 | 0.27 | 0.22 | 0.16 | 0.30 | 5.00 | 0.39 |
| -72.2244141 | 19.72983333 | 0.08 | 0.10 | 0.14 | 0.16 | 0.23 | 0.24 | 0.20 | 0.30 | 5.00 | 0.46 |
| -72.5426872 | 19.12643496 | 0.06 | 0.08 | 0.13 | 0.14 | 0.25 | 0.25 | 0.21 | 0.30 | 5.00 | 0.06 |
| -72.34191374 | 18.54890806 | 0.09 | 0.10 | 0.14 | 0.15 | 0.26 | 0.25 | 0.20 | 0.30 | 5.00 | 0.57 |
| -73.74544153 | 18.19452268 | 0.07 | 0.09 | 0.13 | 0.15 | 0.22 | 0.23 | 0.19 | 0.30 | 5.00 | 0.50 |
| -72.8213446 | 19.945429 | 0.08 | 0.10 | 0.14 | 0.16 | 0.23 | 0.25 | 0.21 | 0.30 | 5.00 | 0.34 |
| -72.68174641 | 19.43958766 | 0.10 | 0.12 | 0.17 | 0.21 | 0.26 | 0.30 | 0.25 | 0.30 | 5.00 | 0.35 |
| -72.68282439 | 19.43931817 | 0.10 | 0.12 | 0.16 | 0.18 | 0.25 | 0.23 | 0.18 | 0.30 | 5.00 | 0.42 |
| -72.29313522 | 18.5122568 | 0.08 | 0.11 | 0.15 | 0.18 | 0.24 | 0.28 | 0.25 | 0.30 | 5.00 | 0.06 |
| -72.54133973 | 19.12535698 | 0.05 | 0.08 | 0.13 | 0.14 | 0.27 | 0.26 | 0.19 | 0.30 | 5.00 | 0.61 |
| -72.68093792 | 19.43904867 | 0.12 | 0.14 | 0.20 | 0.22 | 0.28 | 0.28 | 0.23 | 0.30 | 5.00 | 0.16 |
| -72.2241446 | 18.58502033 | 0.08 | 0.11 | 0.16 | 0.17 | 0.27 | 0.27 | 0.22 | 0.30 | 5.00 | 0.25 |
| -72.82053612 | 19.945429 | 0.08 | 0.10 | 0.16 | 0.17 | 0.24 | 0.26 | 0.21 | 0.30 | 5.00 | 0.17 |
| -72.61598973 | 19.14961149 | 0.06 | 0.08 | 0.13 | 0.15 | 0.24 | 0.25 | 0.20 | 0.30 | 5.00 | 0.58 |
| -72.53487186 | 19.11996709 | 0.06 | 0.08 | 0.13 | 0.14 | 0.32 | 0.27 | 0.19 | 0.30 | 5.00 | 0.17 |
| -73.76161121 | 18.21608225 | 0.07 | 0.09 | 0.14 | 0.14 | 0.31 | 0.27 | 0.21 | 0.30 | 5.00 | 0.55 |
| -74.11923052 | 18.63891925 | 0.01 | 0.02 | 0.06 | 0.05 | 0.35 | 0.17 | 0.09 | 0.30 | 5.00 | 0.56 |
| -72.48232041 | 19.124279 | 0.04 | 0.06 | 0.10 | 0.11 | 0.24 | 0.19 | 0.14 | 0.30 | 5.00 | 0.44 |
| -72.22387511 | 19.72821636 | 0.04 | 0.06 | 0.11 | 0.12 | 0.22 | 0.21 | 0.16 | 0.30 | 5.00 | 0.62 |
| -73.74409406 | 18.1937142 | 0.10 | 0.11 | 0.14 | 0.16 | 0.24 | 0.23 | 0.18 | 0.30 | 5.00 | 0.18 |
| -72.33275092 | 18.5470216 | 0.10 | 0.12 | 0.15 | 0.17 | 0.20 | 0.23 | 0.20 | 0.30 | 5.00 | 0.58 |
| -72.18964929 | 19.72794687 | 0.05 | 0.07 | 0.11 | 0.13 | 0.22 | 0.22 | 0.17 | 0.30 | 5.00 | 0.60 |
| -72.21686825 | 19.73603171 | 0.07 | 0.10 | 0.15 | 0.16 | 0.20 | 0.24 | 0.21 | 0.30 | 5.00 | 0.16 |
| -72.84290417 | 19.93491871 | 0.08 | 0.10 | 0.15 | 0.17 | 0.26 | 0.27 | 0.22 | 0.30 | 5.00 | 0.70 |
| -72.21525128 | 18.57046763 | 0.07 | 0.10 | 0.15 | 0.17 | 0.26 | 0.26 | 0.21 | 0.30 | 5.00 | 0.27 |
| -72.21686825 | 19.73495373 | 0.06 | 0.08 | 0.12 | 0.14 | 0.19 | 0.22 | 0.19 | 0.30 | 5.00 | 0.66 |
| -71.83176049 | 19.65518333 | 0.07 | 0.09 | 0.13 | 0.15 | 0.23 | 0.25 | 0.20 | 0.30 | 5.00 | 0.55 |
| -72.3435307 | 18.54863857 | 0.09 | 0.10 | 0.14 | 0.15 | 0.20 | 0.21 | 0.17 | 0.30 | 5.00 | 0.53 |
| -72.86581121 | 18.43113893 | 0.09 | 0.11 | 0.14 | 0.16 | 0.30 | 0.26 | 0.20 | 0.30 | 5.00 | 0.41 |
| -73.74490255 | 18.19587015 | 0.04 | 0.06 | 0.11 | 0.12 | 0.21 | 0.22 | 0.17 | 0.30 | 5.00 | 0.22 |
| -72.21740724 | 19.73441474 | 0.05 | 0.07 | 0.10 | 0.11 | 0.21 | 0.20 | 0.16 | 0.30 | 5.00 | 0.58 |
| -72.22387511 | 19.72983333 | 0.06 | 0.08 | 0.12 | 0.13 | 0.22 | 0.23 | 0.18 | 0.30 | 5.00 | 0.42 |
| -72.12416211 | 19.67054452 | 0.05 | 0.07 | 0.10 | 0.10 | 0.22 | 0.19 | 0.15 | 0.30 | 5.00 | 0.56 |
| -72.70168901 | 19.09894651 | 0.05 | 0.08 | 0.14 | 0.15 | 0.26 | 0.27 | 0.23 | 0.30 | 5.00 | 0.19 |
| -72.34272222 | 18.54998604 | 0.14 | 0.16 | 0.21 | 0.24 | 0.33 | 0.34 | 0.27 | 0.30 | 5.00 | 0.26 |
| -72.70088052 | 19.09679055 | 0.08 | 0.11 | 0.16 | 0.19 | 0.28 | 0.30 | 0.25 | 0.30 | 5.00 | 0.53 |
| -73.74544153 | 18.19398369 | 0.08 | 0.11 | 0.15 | 0.16 | 0.25 | 0.25 | 0.21 | 0.30 | 5.00 | 0.32 |
| -72.48178142 | 19.12320102 | 0.05 | 0.06 | 0.11 | 0.11 | 0.26 | 0.22 | 0.17 | 0.30 | 5.00 | 0.51 |
| -72.70115002 | 19.09732954 | 0.09 | 0.10 | 0.15 | 0.16 | 0.29 | 0.27 | 0.21 | 0.30 | 5.00 | 0.35 |
| -72.68255489 | 19.44093514 | 0.10 | 0.12 | 0.17 | 0.20 | 0.24 | 0.24 | 0.19 | 0.30 | 5.00 | 0.48 |
| -72.34245272 | 18.54756059 | 0.09 | 0.11 | 0.14 | 0.15 | 0.20 | 0.21 | 0.18 | 0.30 | 5.00 | 0.33 |
| -72.48178142 | 19.12266203 | 0.05 | 0.07 | 0.11 | 0.12 | 0.25 | 0.23 | 0.17 | 0.30 | 5.00 | 0.22 |
| -72.2144428 | 18.56965914 | 0.05 | 0.07 | 0.12 | 0.12 | 0.27 | 0.25 | 0.18 | 0.30 | 5.00 | 0.58 |
| -72.70330597 | 19.09625156 | 0.04 | 0.06 | 0.11 | 0.13 | 0.24 | 0.25 | 0.21 | 0.30 | 5.00 | 0.52 |
| -73.74436356 | 18.19398369 | 0.08 | 0.09 | 0.12 | 0.13 | 0.21 | 0.22 | 0.17 | 0.30 | 5.00 | 0.60 |
| -72.71381626 | 19.92979831 | 0.07 | 0.09 | 0.13 | 0.14 | 0.20 | 0.23 | 0.19 | 0.30 | 5.00 | 0.66 |
| -72.2540585 | 18.52977394 | 0.12 | 0.14 | 0.19 | 0.21 | 0.35 | 0.31 | 0.23 | 0.30 | 5.00 | 0.18 |
| -72.34299171 | 18.54917755 | 0.09 | 0.11 | 0.14 | 0.15 | 0.24 | 0.23 | 0.19 | 0.30 | 5.00 | 0.38 |
| -72.29151825 | 18.51090932 | 0.09 | 0.12 | 0.16 | 0.18 | 0.22 | 0.26 | 0.23 | 0.30 | 5.00 | 0.39 |
| -72.22360561 | 19.72794687 | 0.05 | 0.08 | 0.11 | 0.13 | 0.21 | 0.23 | 0.19 | 0.30 | 5.00 | 0.15 |
| -72.20204605 | 19.75920824 | 0.07 | 0.09 | 0.12 | 0.15 | 0.18 | 0.21 | 0.18 | 0.30 | 5.00 | 0.69 |
| -72.70168901 | 19.09840752 | 0.06 | 0.09 | 0.15 | 0.16 | 0.25 | 0.26 | 0.21 | 0.30 | 5.00 | 0.67 |
| -72.21498179 | 18.56912015 | 0.08 | 0.11 | 0.15 | 0.17 | 0.28 | 0.26 | 0.21 | 0.30 | 5.00 | 0.60 |
| -72.34460868 | 18.54917755 | 0.09 | 0.11 | 0.14 | 0.16 | 0.21 | 0.22 | 0.17 | 0.30 | 5.00 | 0.17 |
| -72.68066843 | 19.4425521 | 0.08 | 0.10 | 0.15 | 0.16 | 0.24 | 0.22 | 0.17 | 0.30 | 5.00 | 0.50 |
| -73.74678901 | 18.19613965 | 0.04 | 0.07 | 0.11 | 0.12 | 0.23 | 0.21 | 0.16 | 0.30 | 5.00 | 0.46 |
| -72.68282439 | 19.4428216 | 0.09 | 0.11 | 0.15 | 0.18 | 0.27 | 0.24 | 0.19 | 0.30 | 5.00 | 0.62 |
| -72.81972763 | 19.94489001 | 0.09 | 0.12 | 0.16 | 0.18 | 0.25 | 0.28 | 0.23 | 0.30 | 5.00 | 0.32 |
| -72.82754298 | 19.93276276 | 0.08 | 0.09 | 0.14 | 0.15 | 0.25 | 0.27 | 0.23 | 0.30 | 5.00 | 0.56 |
| -72.29448269 | 18.51010084 | 0.06 | 0.08 | 0.12 | 0.13 | 0.20 | 0.21 | 0.18 | 0.30 | 5.00 | 0.20 |
| -72.61491175 | 18.5122568 | 0.05 | 0.06 | 0.11 | 0.11 | 0.32 | 0.24 | 0.16 | 0.30 | 5.00 | 0.16 |
| -72.3338289 | 18.54729109 | 0.08 | 0.10 | 0.13 | 0.15 | 0.20 | 0.21 | 0.17 | 0.30 | 5.00 | 0.61 |
| -72.21686825 | 19.73576221 | 0.07 | 0.09 | 0.13 | 0.15 | 0.20 | 0.23 | 0.20 | 0.30 | 5.00 | 0.31 |
| -72.12335363 | 19.67081402 | 0.05 | 0.07 | 0.10 | 0.12 | 0.18 | 0.20 | 0.16 | 0.30 | 5.00 | 0.02 |
| -74.11869154 | 18.63972774 | 0.08 | 0.10 | 0.14 | 0.15 | 0.26 | 0.23 | 0.17 | 0.30 | 5.00 | 0.61 |
| -73.74463305 | 18.19506167 | 0.08 | 0.10 | 0.13 | 0.16 | 0.22 | 0.24 | 0.19 | 0.30 | 5.00 | 0.46 |
| -72.48070345 | 19.12347052 | 0.06 | 0.09 | 0.13 | 0.14 | 0.23 | 0.23 | 0.17 | 0.30 | 5.00 | 0.27 |
| -72.82808197 | 19.93303225 | 0.07 | 0.09 | 0.13 | 0.15 | 0.24 | 0.27 | 0.22 | 0.30 | 5.00 | 0.27 |
| -72.70141951 | 19.09652106 | 0.06 | 0.08 | 0.13 | 0.15 | 0.25 | 0.28 | 0.23 | 0.30 | 5.00 | 0.19 |
| -73.74625002 | 18.19587015 | 0.10 | 0.12 | 0.17 | 0.19 | 0.24 | 0.26 | 0.21 | 0.30 | 5.00 | 0.35 |
| -72.20177655 | 19.75920824 | 0.07 | 0.08 | 0.12 | 0.14 | 0.17 | 0.21 | 0.18 | 0.30 | 5.00 | 0.08 |
| -74.1203085 | 18.63811077 | 0.01 | 0.03 | 0.07 | 0.06 | 0.32 | 0.17 | 0.10 | 0.30 | 5.00 | 0.33 |
| -72.81972763 | 19.94462052 | 0.08 | 0.10 | 0.16 | 0.17 | 0.26 | 0.27 | 0.21 | 0.30 | 5.00 | 0.63 |
| -72.30445399 | 18.5917577 | 0.11 | 0.13 | 0.17 | 0.18 | 0.28 | 0.28 | 0.24 | 0.30 | 5.00 | 0.21 |
| -72.29286572 | 18.51171781 | 0.07 | 0.10 | 0.15 | 0.17 | 0.22 | 0.25 | 0.22 | 0.30 | 5.00 | 0.24 |
| -72.12335363 | 19.67000553 | 0.04 | 0.06 | 0.10 | 0.11 | 0.21 | 0.19 | 0.15 | 0.30 | 5.00 | 0.65 |
| -72.82026662 | 19.94435102 | 0.06 | 0.08 | 0.12 | 0.13 | 0.24 | 0.25 | 0.20 | 0.30 | 5.00 | 0.59 |
| -73.76080273 | 18.21527376 | 0.04 | 0.06 | 0.10 | 0.11 | 0.36 | 0.25 | 0.17 | 0.30 | 5.00 | 0.23 |
| -72.71408576 | 19.92979831 | 0.06 | 0.08 | 0.12 | 0.13 | 0.21 | 0.20 | 0.16 | 0.30 | 5.00 | 0.53 |
| -73.76107222 | 18.21662124 | 0.05 | 0.08 | 0.13 | 0.14 | 0.33 | 0.26 | 0.19 | 0.30 | 5.00 | 0.32 |
| -72.86581121 | 18.43033044 | 0.07 | 0.08 | 0.12 | 0.13 | 0.25 | 0.23 | 0.17 | 0.30 | 5.00 | 0.33 |
| -72.71381626 | 19.9306068 | 0.07 | 0.10 | 0.13 | 0.15 | 0.20 | 0.22 | 0.18 | 0.30 | 5.00 | 0.03 |
| -72.48151193 | 19.12320102 | 0.03 | 0.04 | 0.09 | 0.09 | 0.28 | 0.20 | 0.14 | 0.30 | 5.00 | 0.48 |
| -72.25244153 | 18.53004344 | 0.09 | 0.11 | 0.16 | 0.18 | 0.31 | 0.28 | 0.23 | 0.30 | 5.00 | 0.31 |
| -72.29259623 | 18.51010084 | 0.08 | 0.11 | 0.15 | 0.17 | 0.21 | 0.25 | 0.22 | 0.30 | 5.00 | 0.41 |
| -72.25432799 | 18.53112142 | 0.10 | 0.12 | 0.18 | 0.20 | 0.36 | 0.32 | 0.24 | 0.30 | 5.00 | 0.37 |
| -72.20204605 | 19.75866925 | 0.08 | 0.09 | 0.12 | 0.14 | 0.19 | 0.22 | 0.19 | 0.30 | 5.00 | 0.58 |
| -72.61545074 | 18.51279579 | 0.05 | 0.06 | 0.10 | 0.10 | 0.29 | 0.22 | 0.15 | 0.30 | 5.00 | 0.14 |
| -72.70303648 | 19.09706005 | 0.07 | 0.10 | 0.14 | 0.16 | 0.26 | 0.28 | 0.23 | 0.30 | 5.00 | 0.65 |
| -72.20339352 | 19.75974723 | 0.07 | 0.08 | 0.12 | 0.14 | 0.17 | 0.21 | 0.18 | 0.30 | 5.00 | 0.62 |
| -72.1244316 | 19.67054452 | 0.05 | 0.07 | 0.11 | 0.12 | 0.21 | 0.21 | 0.16 | 0.30 | 5.00 | 0.15 |
| -72.29286572 | 18.51063983 | 0.10 | 0.12 | 0.17 | 0.20 | 0.25 | 0.28 | 0.25 | 0.30 | 5.00 | 0.09 |
| -72.84263467 | 19.93626618 | 0.08 | 0.11 | 0.15 | 0.17 | 0.24 | 0.28 | 0.24 | 0.30 | 5.00 | 0.37 |
| -74.11923052 | 18.63891925 | 0.01 | 0.02 | 0.06 | 0.05 | 0.35 | 0.17 | 0.09 | 0.30 | 5.00 | 0.37 |
| -72.19072727 | 19.72875535 | 0.06 | 0.08 | 0.12 | 0.14 | 0.21 | 0.23 | 0.19 | 0.30 | 5.00 | 0.37 |
| -73.74678901 | 18.19452268 | 0.03 | 0.05 | 0.09 | 0.09 | 0.24 | 0.20 | 0.15 | 0.30 | 5.00 | 0.20 |
| -72.32763052 | 18.49932106 | 0.23 | 0.26 | 0.33 | 0.39 | 0.56 | 0.66 | 0.44 | 0.30 | 6.00 | 0.26 |
| -73.25819532 | 18.87688297 | 0.13 | 0.16 | 0.21 | 0.23 | 0.34 | 0.25 | 0.13 | 0.30 | 6.00 | 0.27 |
| -72.0597529 | 19.68509723 | 0.05 | 0.08 | 0.11 | 0.12 | 0.17 | 0.12 | 0.05 | 0.30 | 6.00 | 0.57 |
| -72.3737141 | 18.72569651 | 0.18 | 0.22 | 0.34 | 0.40 | 0.49 | 0.56 | 0.41 | 0.30 | 6.00 | 0.59 |
| -72.37317511 | 18.72542701 | 0.21 | 0.25 | 0.37 | 0.44 | 0.52 | 0.61 | 0.47 | 0.30 | 6.00 | 0.61 |
| -73.42555146 | 18.38909777 | 0.11 | 0.13 | 0.17 | 0.20 | 0.33 | 0.32 | 0.23 | 0.30 | 6.00 | 0.40 |
| -71.96677727 | 18.427366 | 0.23 | 0.27 | 0.34 | 0.39 | 0.51 | 0.50 | 0.37 | 0.30 | 6.00 | 0.15 |
| -72.81487673 | 19.50965626 | 0.15 | 0.17 | 0.22 | 0.27 | 0.35 | 0.36 | 0.27 | 0.31 | 6.00 | 0.23 |
| -74.01897854 | 18.55321997 | 0.22 | 0.25 | 0.31 | 0.34 | 0.44 | 0.43 | 0.31 | 0.30 | 6.00 | 0.51 |
| -73.60557385 | 18.05654145 | 0.05 | 0.06 | 0.12 | 0.11 | 0.38 | 0.32 | 0.18 | 0.30 | 6.00 | 0.22 |
| -73.91360616 | 18.30690192 | 0.18 | 0.20 | 0.26 | 0.29 | 0.37 | 0.36 | 0.26 | 0.30 | 6.00 | 0.42 |
| -72.0597529 | 19.68509723 | 0.05 | 0.08 | 0.11 | 0.12 | 0.17 | 0.12 | 0.05 | 0.30 | 6.00 | 0.06 |
| -72.81460724 | 19.50965626 | 0.12 | 0.14 | 0.19 | 0.23 | 0.31 | 0.34 | 0.27 | 0.31 | 6.00 | 0.64 |
| -72.37317511 | 18.72569651 | 0.19 | 0.24 | 0.35 | 0.41 | 0.50 | 0.59 | 0.44 | 0.30 | 6.00 | 0.47 |
| -72.3386798 | 18.4820734 | 0.21 | 0.26 | 0.37 | 0.41 | 0.58 | 0.66 | 0.48 | 0.30 | 6.00 | 0.16 |
| -72.37290561 | 18.72569651 | 0.21 | 0.25 | 0.37 | 0.44 | 0.52 | 0.61 | 0.45 | 0.30 | 6.00 | 0.53 |
| -72.37317511 | 18.725966 | 0.21 | 0.25 | 0.36 | 0.42 | 0.51 | 0.61 | 0.45 | 0.30 | 6.00 | 0.67 |
| -72.0597529 | 19.68509723 | 0.05 | 0.08 | 0.11 | 0.12 | 0.17 | 0.12 | 0.05 | 0.30 | 6.00 | 0.10 |
| -72.23842781 | 18.68230788 | 0.24 | 0.28 | 0.38 | 0.45 | 0.62 | 0.70 | 0.52 | 0.30 | 6.00 | 0.67 |
| -72.98519731 | 18.87634398 | 0.19 | 0.22 | 0.32 | 0.41 | 0.55 | 0.47 | 0.28 | 0.30 | 6.00 | 0.42 |
| -72.32628305 | 18.49878207 | 0.28 | 0.32 | 0.38 | 0.42 | 0.60 | 0.68 | 0.48 | 0.30 | 6.00 | 0.02 |
| -72.32709153 | 18.49905156 | 0.20 | 0.22 | 0.27 | 0.32 | 0.50 | 0.65 | 0.43 | 0.30 | 6.00 | 0.05 |
| -72.702228 | 19.91362864 | 0.10 | 0.11 | 0.16 | 0.18 | 0.25 | 0.26 | 0.19 | 0.30 | 6.00 | 0.45 |
| -73.87533793 | 18.37400607 | 0.05 | 0.06 | 0.11 | 0.12 | 0.41 | 0.31 | 0.18 | 0.30 | 6.00 | 0.67 |
| -71.96677727 | 18.427366 | 0.23 | 0.27 | 0.34 | 0.39 | 0.51 | 0.50 | 0.37 | 0.30 | 6.00 | 0.43 |
| -72.32682204 | 18.49878207 | 0.27 | 0.31 | 0.39 | 0.46 | 0.64 | 0.75 | 0.52 | 0.30 | 6.00 | 0.27 |
| -72.32736103 | 18.49932106 | 0.24 | 0.28 | 0.35 | 0.39 | 0.53 | 0.67 | 0.49 | 0.30 | 6.00 | 0.46 |
| -73.25900381 | 18.87769145 | 0.15 | 0.19 | 0.26 | 0.28 | 0.28 | 0.15 | 0.08 | 0.30 | 6.00 | 0.46 |
| -73.25846482 | 18.87742196 | 0.12 | 0.14 | 0.19 | 0.21 | 0.34 | 0.22 | 0.11 | 0.30 | 6.00 | 0.06 |
| -73.89366356 | 18.29261871 | 0.18 | 0.21 | 0.26 | 0.29 | 0.38 | 0.38 | 0.28 | 0.30 | 6.00 | 0.32 |
| -72.49390868 | 18.5168382 | 0.13 | 0.16 | 0.23 | 0.28 | 0.45 | 0.48 | 0.33 | 0.30 | 6.00 | 0.48 |
| -72.0848159 | 19.69237359 | 0.11 | 0.13 | 0.19 | 0.23 | 0.35 | 0.44 | 0.33 | 0.30 | 6.00 | 0.17 |
| -74.10845074 | 18.57747449 | 0.06 | 0.07 | 0.13 | 0.14 | 0.29 | 0.29 | 0.19 | 0.30 | 6.00 | 0.59 |
| -74.10818125 | 18.57774398 | 0.09 | 0.11 | 0.15 | 0.18 | 0.30 | 0.30 | 0.20 | 0.30 | 6.00 | 0.29 |
| -73.25873431 | 18.87742196 | 0.15 | 0.18 | 0.25 | 0.27 | 0.29 | 0.18 | 0.09 | 0.30 | 6.00 | 0.20 |
| -73.87803287 | 18.18643784 | 0.11 | 0.14 | 0.19 | 0.21 | 0.35 | 0.34 | 0.26 | 0.30 | 6.00 | 0.06 |
| -72.33814081 | 18.4823429 | 0.27 | 0.33 | 0.43 | 0.51 | 0.64 | 0.73 | 0.55 | 0.30 | 6.00 | 0.68 |
| -73.87830237 | 18.18643784 | 0.12 | 0.14 | 0.19 | 0.23 | 0.35 | 0.36 | 0.28 | 0.30 | 6.00 | 0.29 |
| -73.89339406 | 18.29207972 | 0.19 | 0.22 | 0.27 | 0.30 | 0.38 | 0.38 | 0.28 | 0.30 | 6.00 | 0.23 |
| -73.89393305 | 18.2928882 | 0.19 | 0.22 | 0.27 | 0.31 | 0.38 | 0.37 | 0.28 | 0.30 | 6.00 | 0.39 |
| -73.60530435 | 18.05654145 | 0.05 | 0.06 | 0.11 | 0.11 | 0.35 | 0.33 | 0.19 | 0.30 | 6.00 | 0.14 |
| -72.37317511 | 18.725966 | 0.21 | 0.25 | 0.36 | 0.42 | 0.51 | 0.61 | 0.45 | 0.30 | 6.00 | 0.03 |
| -73.85323937 | 18.27348459 | 0.24 | 0.27 | 0.32 | 0.35 | 0.43 | 0.43 | 0.30 | 0.30 | 6.00 | 0.69 |
| -72.08427691 | 19.6915651 | 0.11 | 0.13 | 0.18 | 0.21 | 0.30 | 0.33 | 0.22 | 0.30 | 6.00 | 0.41 |
| -74.10845074 | 18.57774398 | 0.04 | 0.06 | 0.10 | 0.10 | 0.31 | 0.26 | 0.15 | 0.30 | 6.00 | 0.47 |
| -72.08427691 | 19.6918346 | 0.11 | 0.13 | 0.18 | 0.21 | 0.30 | 0.32 | 0.21 | 0.30 | 6.00 | 0.36 |
| -73.42635995 | 18.38882828 | 0.10 | 0.12 | 0.17 | 0.19 | 0.29 | 0.25 | 0.18 | 0.30 | 6.00 | 0.37 |
| -72.15730994 | 18.69308766 | 0.19 | 0.23 | 0.31 | 0.36 | 0.52 | 0.55 | 0.42 | 0.30 | 6.00 | 0.27 |
| -72.32682204 | 18.49878207 | 0.27 | 0.31 | 0.39 | 0.46 | 0.64 | 0.75 | 0.52 | 0.30 | 6.00 | 0.04 |
| -72.08427691 | 19.69210409 | 0.10 | 0.12 | 0.17 | 0.19 | 0.28 | 0.31 | 0.18 | 0.30 | 6.00 | 0.02 |
| -71.97162817 | 18.46078333 | 0.20 | 0.24 | 0.34 | 0.40 | 0.56 | 0.57 | 0.40 | 0.30 | 6.00 | 0.48 |
| -73.25846482 | 18.87715246 | 0.14 | 0.16 | 0.22 | 0.24 | 0.31 | 0.19 | 0.11 | 0.30 | 6.00 | 0.41 |
| -72.32709153 | 18.49878207 | 0.25 | 0.29 | 0.36 | 0.41 | 0.57 | 0.67 | 0.47 | 0.30 | 6.00 | 0.32 |
| -72.58176392 | 18.5222281 | 0.10 | 0.12 | 0.16 | 0.18 | 0.28 | 0.27 | 0.20 | 0.30 | 6.00 | 0.21 |
| -73.25819532 | 18.87634398 | 0.13 | 0.16 | 0.22 | 0.21 | 0.07 | 0.02 | 0.01 | 0.30 | 6.00 | 0.23 |
| -72.32655254 | 18.49878207 | 0.27 | 0.31 | 0.39 | 0.46 | 0.64 | 0.75 | 0.52 | 0.30 | 6.00 | 0.34 |
| -73.42609045 | 18.38909777 | 0.08 | 0.10 | 0.15 | 0.16 | 0.23 | 0.19 | 0.14 | 0.30 | 6.00 | 0.45 |
| -72.32763052 | 18.49932106 | 0.23 | 0.26 | 0.33 | 0.39 | 0.56 | 0.66 | 0.44 | 0.30 | 6.00 | 0.09 |
| -72.0600224 | 19.68482774 | 0.06 | 0.07 | 0.11 | 0.11 | 0.18 | 0.15 | 0.07 | 0.30 | 6.00 | 0.40 |
| -72.32709153 | 18.49851257 | 0.26 | 0.30 | 0.39 | 0.45 | 0.63 | 0.73 | 0.51 | 0.30 | 6.00 | 0.26 |
| -72.32682204 | 18.49851257 | 0.25 | 0.29 | 0.37 | 0.44 | 0.63 | 0.68 | 0.46 | 0.30 | 6.00 | 0.39 |
| -73.42609045 | 18.38882828 | 0.12 | 0.14 | 0.19 | 0.22 | 0.34 | 0.32 | 0.24 | 0.30 | 6.00 | 0.46 |
| -72.08427691 | 19.6918346 | 0.11 | 0.13 | 0.18 | 0.21 | 0.30 | 0.32 | 0.21 | 0.30 | 6.00 | 0.32 |
| -72.0600224 | 19.68509723 | 0.06 | 0.07 | 0.11 | 0.11 | 0.18 | 0.14 | 0.07 | 0.30 | 6.00 | 0.41 |
| -72.0600224 | 19.68482774 | 0.06 | 0.07 | 0.11 | 0.11 | 0.18 | 0.15 | 0.07 | 0.30 | 6.00 | 0.43 |
| -72.32736103 | 18.49851257 | 0.30 | 0.34 | 0.44 | 0.53 | 0.72 | 0.79 | 0.55 | 0.30 | 6.00 | 0.28 |
| -73.89366356 | 18.29261871 | 0.18 | 0.21 | 0.26 | 0.29 | 0.38 | 0.38 | 0.28 | 0.30 | 6.00 | 0.34 |
| -72.32763052 | 18.49932106 | 0.23 | 0.26 | 0.33 | 0.39 | 0.56 | 0.66 | 0.44 | 0.30 | 6.00 | 0.18 |
| -73.20860832 | 19.90204037 | 0.10 | 0.11 | 0.14 | 0.13 | 0.09 | 0.07 | 0.05 | 0.30 | 6.00 | 0.29 |
| -72.08454641 | 19.6918346 | 0.13 | 0.16 | 0.21 | 0.25 | 0.36 | 0.46 | 0.33 | 0.30 | 6.00 | 0.05 |
| -73.36060327 | 19.84221257 | 0.16 | 0.19 | 0.26 | 0.33 | 0.45 | 0.42 | 0.25 | 0.30 | 6.00 | 0.36 |
| -72.25971789 | 18.67961293 | 0.30 | 0.36 | 0.46 | 0.54 | 0.65 | 0.71 | 0.54 | 0.30 | 6.00 | 0.05 |
| -73.89366356 | 18.29234921 | 0.19 | 0.22 | 0.27 | 0.30 | 0.38 | 0.37 | 0.28 | 0.30 | 6.00 | 0.26 |
| -72.15730994 | 18.69308766 | 0.19 | 0.23 | 0.31 | 0.36 | 0.52 | 0.55 | 0.42 | 0.30 | 6.00 | 0.29 |
| -72.08427691 | 19.6918346 | 0.11 | 0.13 | 0.18 | 0.21 | 0.30 | 0.32 | 0.21 | 0.30 | 6.00 | 0.09 |
| -72.3386798 | 18.48180391 | 0.22 | 0.27 | 0.38 | 0.45 | 0.59 | 0.68 | 0.48 | 0.30 | 6.00 | 0.00 |
| -72.25998738 | 18.67934344 | 0.26 | 0.31 | 0.40 | 0.46 | 0.61 | 0.64 | 0.48 | 0.30 | 6.00 | 0.54 |
| -73.87479894 | 18.37373658 | 0.07 | 0.09 | 0.15 | 0.15 | 0.41 | 0.36 | 0.23 | 0.30 | 6.00 | 0.01 |
| -73.87803287 | 18.18589885 | 0.15 | 0.18 | 0.22 | 0.26 | 0.34 | 0.31 | 0.24 | 0.30 | 6.00 | 0.55 |
| -72.08454641 | 19.69237359 | 0.12 | 0.15 | 0.21 | 0.25 | 0.35 | 0.38 | 0.26 | 0.30 | 6.00 | 0.41 |
| -73.25792583 | 18.87661348 | 0.13 | 0.15 | 0.21 | 0.23 | 0.37 | 0.26 | 0.14 | 0.30 | 6.00 | 0.65 |
| -73.87803287 | 18.18616835 | 0.14 | 0.16 | 0.21 | 0.24 | 0.35 | 0.33 | 0.25 | 0.30 | 6.00 | 0.35 |
| -73.25846482 | 18.87742196 | 0.12 | 0.14 | 0.19 | 0.21 | 0.34 | 0.22 | 0.11 | 0.30 | 6.00 | 0.24 |
| -73.85323937 | 18.27375409 | 0.20 | 0.23 | 0.28 | 0.32 | 0.41 | 0.40 | 0.28 | 0.30 | 6.00 | 0.14 |
| -72.3734446 | 18.72569651 | 0.19 | 0.23 | 0.35 | 0.42 | 0.49 | 0.58 | 0.43 | 0.30 | 6.00 | 0.18 |
| -72.3734446 | 18.725966 | 0.20 | 0.24 | 0.36 | 0.41 | 0.50 | 0.59 | 0.44 | 0.30 | 6.00 | 0.18 |
| -73.25819532 | 18.87661348 | 0.14 | 0.18 | 0.24 | 0.25 | 0.18 | 0.10 | 0.06 | 0.30 | 6.00 | 0.14 |
| -73.20833883 | 19.90204037 | 0.13 | 0.15 | 0.19 | 0.19 | 0.19 | 0.19 | 0.14 | 0.30 | 6.00 | 0.41 |
| -72.08454641 | 19.6918346 | 0.13 | 0.16 | 0.21 | 0.25 | 0.36 | 0.46 | 0.33 | 0.30 | 6.00 | 0.24 |
| -73.25873431 | 18.87742196 | 0.15 | 0.18 | 0.25 | 0.27 | 0.29 | 0.18 | 0.09 | 0.30 | 6.00 | 0.63 |
| -73.42582096 | 18.38909777 | 0.11 | 0.13 | 0.18 | 0.20 | 0.30 | 0.27 | 0.20 | 0.30 | 6.00 | 0.07 |
| -73.89339406 | 18.29234921 | 0.19 | 0.22 | 0.27 | 0.30 | 0.38 | 0.38 | 0.28 | 0.30 | 6.00 | 0.03 |
| -72.32736103 | 18.49932106 | 0.24 | 0.28 | 0.35 | 0.39 | 0.53 | 0.67 | 0.49 | 0.30 | 6.00 | 0.63 |
| -72.37317511 | 18.72569651 | 0.19 | 0.24 | 0.35 | 0.41 | 0.50 | 0.59 | 0.44 | 0.30 | 6.00 | 0.30 |
| -73.89339406 | 18.29234921 | 0.19 | 0.22 | 0.27 | 0.30 | 0.38 | 0.38 | 0.28 | 0.30 | 6.00 | 0.63 |
| -72.3734446 | 18.72569651 | 0.19 | 0.23 | 0.35 | 0.42 | 0.49 | 0.58 | 0.43 | 0.30 | 6.00 | 0.43 |
| -72.37290561 | 18.72542701 | 0.20 | 0.24 | 0.36 | 0.42 | 0.50 | 0.60 | 0.44 | 0.30 | 6.00 | 0.19 |
| -72.32709153 | 18.49905156 | 0.20 | 0.22 | 0.27 | 0.32 | 0.50 | 0.65 | 0.43 | 0.30 | 6.00 | 0.11 |
| -73.87506843 | 18.37373658 | 0.08 | 0.10 | 0.15 | 0.16 | 0.41 | 0.36 | 0.25 | 0.30 | 6.00 | 0.00 |
| -72.32709153 | 18.49878207 | 0.25 | 0.29 | 0.36 | 0.41 | 0.57 | 0.67 | 0.47 | 0.30 | 6.00 | 0.08 |
| -73.25819532 | 18.87661348 | 0.14 | 0.18 | 0.24 | 0.25 | 0.18 | 0.10 | 0.06 | 0.30 | 6.00 | 0.53 |
| -72.08427691 | 19.6918346 | 0.11 | 0.13 | 0.18 | 0.21 | 0.30 | 0.32 | 0.21 | 0.30 | 6.00 | 0.04 |
| -72.33841031 | 18.4823429 | 0.26 | 0.31 | 0.43 | 0.50 | 0.64 | 0.74 | 0.56 | 0.30 | 6.00 | 0.41 |
| -73.25873431 | 18.87715246 | 0.15 | 0.19 | 0.26 | 0.25 | 0.12 | 0.06 | 0.04 | 0.30 | 6.00 | 0.44 |
| -74.10818125 | 18.57801348 | 0.06 | 0.08 | 0.13 | 0.14 | 0.31 | 0.30 | 0.19 | 0.30 | 6.00 | 0.58 |
| -72.37317511 | 18.72569651 | 0.19 | 0.24 | 0.35 | 0.41 | 0.50 | 0.59 | 0.44 | 0.30 | 6.00 | 0.42 |
| -72.15730994 | 18.69335716 | 0.12 | 0.16 | 0.22 | 0.26 | 0.45 | 0.49 | 0.34 | 0.30 | 6.00 | 0.46 |
| -72.33841031 | 18.4823429 | 0.26 | 0.31 | 0.43 | 0.50 | 0.64 | 0.74 | 0.56 | 0.30 | 6.00 | 0.21 |
| -72.08454641 | 19.6918346 | 0.13 | 0.16 | 0.21 | 0.25 | 0.36 | 0.46 | 0.33 | 0.30 | 6.00 | 0.43 |
| -72.32628305 | 18.49878207 | 0.28 | 0.32 | 0.38 | 0.42 | 0.60 | 0.68 | 0.48 | 0.30 | 6.00 | 0.24 |
| -73.87830237 | 18.18589885 | 0.15 | 0.18 | 0.23 | 0.26 | 0.36 | 0.33 | 0.25 | 0.30 | 6.00 | 0.36 |
| -74.10845074 | 18.57720499 | 0.08 | 0.10 | 0.15 | 0.16 | 0.30 | 0.31 | 0.21 | 0.30 | 6.00 | 0.55 |
| -72.06029189 | 19.68455824 | 0.05 | 0.06 | 0.09 | 0.11 | 0.17 | 0.14 | 0.07 | 0.30 | 6.00 | 0.66 |
| -72.98519731 | 18.87634398 | 0.19 | 0.22 | 0.32 | 0.41 | 0.55 | 0.47 | 0.28 | 0.30 | 6.00 | 0.52 |
| -73.20887782 | 19.90177088 | 0.10 | 0.12 | 0.15 | 0.15 | 0.14 | 0.13 | 0.09 | 0.30 | 6.00 | 0.53 |
| -72.33814081 | 18.4820734 | 0.26 | 0.32 | 0.43 | 0.51 | 0.65 | 0.74 | 0.55 | 0.30 | 6.00 | 0.54 |
| -73.87830237 | 18.18670734 | 0.11 | 0.13 | 0.18 | 0.22 | 0.34 | 0.36 | 0.27 | 0.30 | 6.00 | 0.63 |
| -72.08454641 | 19.69237359 | 0.12 | 0.15 | 0.21 | 0.25 | 0.35 | 0.38 | 0.26 | 0.30 | 6.00 | 0.23 |
| -73.25846482 | 18.87688297 | 0.13 | 0.17 | 0.24 | 0.25 | 0.15 | 0.08 | 0.05 | 0.30 | 6.00 | 0.51 |
| -73.25846482 | 18.87715246 | 0.14 | 0.16 | 0.22 | 0.24 | 0.31 | 0.19 | 0.11 | 0.30 | 6.00 | 0.57 |
| -73.91333666 | 18.30717142 | 0.07 | 0.09 | 0.13 | 0.13 | 0.34 | 0.28 | 0.17 | 0.30 | 6.00 | 0.09 |
| -73.89366356 | 18.29261871 | 0.18 | 0.21 | 0.26 | 0.29 | 0.38 | 0.38 | 0.28 | 0.30 | 6.00 | 0.05 |
| -73.91414515 | 18.35918387 | 0.08 | 0.10 | 0.15 | 0.16 | 0.35 | 0.37 | 0.23 | 0.30 | 6.00 | 0.33 |
| -72.37317511 | 18.72542701 | 0.21 | 0.25 | 0.37 | 0.44 | 0.52 | 0.61 | 0.47 | 0.30 | 6.00 | 0.33 |
| -72.0848159 | 19.69237359 | 0.11 | 0.13 | 0.19 | 0.23 | 0.35 | 0.44 | 0.33 | 0.30 | 6.00 | 0.44 |
| -72.08427691 | 19.69210409 | 0.10 | 0.12 | 0.17 | 0.19 | 0.28 | 0.31 | 0.18 | 0.30 | 6.00 | 0.63 |
| -73.60530435 | 18.05654145 | 0.05 | 0.06 | 0.11 | 0.11 | 0.35 | 0.33 | 0.19 | 0.30 | 6.00 | 0.05 |
| -72.08454641 | 19.69237359 | 0.12 | 0.15 | 0.21 | 0.25 | 0.35 | 0.38 | 0.26 | 0.30 | 6.00 | 0.56 |
| -73.60584334 | 18.05654145 | 0.05 | 0.06 | 0.12 | 0.11 | 0.38 | 0.31 | 0.17 | 0.30 | 6.00 | 0.28 |
| -71.97135868 | 18.46078333 | 0.19 | 0.23 | 0.33 | 0.39 | 0.56 | 0.57 | 0.40 | 0.30 | 6.00 | 0.58 |
| -72.37317511 | 18.72542701 | 0.21 | 0.25 | 0.37 | 0.44 | 0.52 | 0.61 | 0.47 | 0.30 | 6.00 | 0.07 |
| -73.60503486 | 18.05627196 | 0.08 | 0.10 | 0.14 | 0.15 | 0.38 | 0.40 | 0.25 | 0.30 | 6.00 | 0.17 |
| -72.3386798 | 18.4820734 | 0.21 | 0.26 | 0.37 | 0.41 | 0.58 | 0.66 | 0.48 | 0.30 | 6.00 | 0.52 |
| -73.87803287 | 18.18616835 | 0.14 | 0.16 | 0.21 | 0.24 | 0.35 | 0.33 | 0.25 | 0.30 | 6.00 | 0.19 |
| -74.10872024 | 18.57720499 | 0.03 | 0.04 | 0.08 | 0.07 | 0.35 | 0.23 | 0.13 | 0.30 | 6.00 | 0.33 |
| -72.08454641 | 19.6918346 | 0.13 | 0.16 | 0.21 | 0.25 | 0.36 | 0.46 | 0.33 | 0.30 | 6.00 | 0.17 |
| -74.10818125 | 18.57774398 | 0.09 | 0.11 | 0.15 | 0.18 | 0.30 | 0.30 | 0.20 | 0.30 | 6.00 | 0.48 |
| -72.98492782 | 18.87688297 | 0.14 | 0.16 | 0.23 | 0.29 | 0.47 | 0.35 | 0.18 | 0.30 | 6.00 | 0.16 |
| -72.16243034 | 18.69066221 | 0.21 | 0.25 | 0.35 | 0.43 | 0.61 | 0.68 | 0.49 | 0.30 | 6.00 | 0.62 |
| -72.33894929 | 18.4820734 | 0.19 | 0.23 | 0.31 | 0.36 | 0.48 | 0.56 | 0.41 | 0.30 | 6.00 | 0.26 |
| -72.98519731 | 18.87634398 | 0.19 | 0.22 | 0.32 | 0.41 | 0.55 | 0.47 | 0.28 | 0.30 | 6.00 | 0.49 |
| -72.15730994 | 18.69335716 | 0.12 | 0.16 | 0.22 | 0.26 | 0.45 | 0.49 | 0.34 | 0.30 | 6.00 | 0.25 |
| -74.10845074 | 18.57720499 | 0.08 | 0.10 | 0.15 | 0.16 | 0.30 | 0.31 | 0.21 | 0.30 | 6.00 | 0.66 |
| -73.85323937 | 18.27348459 | 0.24 | 0.27 | 0.32 | 0.35 | 0.43 | 0.43 | 0.30 | 0.30 | 6.00 | 0.29 |
| -72.98492782 | 18.87661348 | 0.17 | 0.21 | 0.28 | 0.36 | 0.51 | 0.42 | 0.24 | 0.30 | 6.00 | 0.10 |
| -74.10845074 | 18.57747449 | 0.06 | 0.07 | 0.13 | 0.14 | 0.29 | 0.29 | 0.19 | 0.30 | 6.00 | 0.69 |
| -73.91333666 | 18.30690192 | 0.14 | 0.16 | 0.21 | 0.24 | 0.35 | 0.34 | 0.24 | 0.30 | 6.00 | 0.68 |
| -73.60503486 | 18.05654145 | 0.04 | 0.05 | 0.09 | 0.09 | 0.35 | 0.28 | 0.15 | 0.30 | 6.00 | 0.40 |
| -72.08454641 | 19.69210409 | 0.13 | 0.16 | 0.21 | 0.26 | 0.37 | 0.45 | 0.32 | 0.30 | 6.00 | 0.40 |
| -72.33814081 | 18.48261239 | 0.26 | 0.31 | 0.43 | 0.51 | 0.62 | 0.73 | 0.55 | 0.30 | 6.00 | 0.53 |
| -72.08427691 | 19.69210409 | 0.10 | 0.12 | 0.17 | 0.19 | 0.28 | 0.31 | 0.18 | 0.30 | 6.00 | 0.52 |
| -72.32763052 | 18.49932106 | 0.23 | 0.26 | 0.33 | 0.39 | 0.56 | 0.66 | 0.44 | 0.30 | 6.00 | 0.31 |
| -72.3386798 | 18.4820734 | 0.21 | 0.26 | 0.37 | 0.41 | 0.58 | 0.66 | 0.48 | 0.30 | 6.00 | 0.57 |
| -73.42609045 | 18.38855878 | 0.12 | 0.14 | 0.18 | 0.21 | 0.34 | 0.33 | 0.25 | 0.30 | 6.00 | 0.55 |
| -72.3737141 | 18.725966 | 0.19 | 0.24 | 0.35 | 0.41 | 0.49 | 0.56 | 0.40 | 0.30 | 6.00 | 0.60 |
| -73.87830237 | 18.18643784 | 0.12 | 0.14 | 0.19 | 0.23 | 0.35 | 0.36 | 0.28 | 0.30 | 6.00 | 0.09 |
| -72.15704045 | 18.69362665 | 0.07 | 0.09 | 0.15 | 0.17 | 0.38 | 0.39 | 0.26 | 0.30 | 6.00 | 0.06 |
| -74.10845074 | 18.57720499 | 0.08 | 0.10 | 0.15 | 0.16 | 0.30 | 0.31 | 0.21 | 0.30 | 6.00 | 0.21 |
| -72.33814081 | 18.4823429 | 0.27 | 0.33 | 0.43 | 0.51 | 0.64 | 0.73 | 0.55 | 0.30 | 6.00 | 0.44 |
| -72.33841031 | 18.48261239 | 0.25 | 0.30 | 0.41 | 0.49 | 0.60 | 0.71 | 0.53 | 0.30 | 6.00 | 0.54 |
| -73.25819532 | 18.87661348 | 0.14 | 0.18 | 0.24 | 0.25 | 0.18 | 0.10 | 0.06 | 0.30 | 6.00 | 0.38 |
| -73.91360616 | 18.30717142 | 0.16 | 0.18 | 0.23 | 0.26 | 0.36 | 0.35 | 0.25 | 0.30 | 6.00 | 0.45 |
| -72.0597529 | 19.68482774 | 0.06 | 0.08 | 0.11 | 0.12 | 0.19 | 0.17 | 0.08 | 0.30 | 6.00 | 0.05 |
| -72.58203341 | 18.5219586 | 0.10 | 0.12 | 0.17 | 0.19 | 0.28 | 0.26 | 0.19 | 0.30 | 6.00 | 0.48 |
| -72.32709153 | 18.49851257 | 0.26 | 0.30 | 0.39 | 0.45 | 0.63 | 0.73 | 0.51 | 0.30 | 6.00 | 0.62 |
| -72.81514623 | 19.50992575 | 0.16 | 0.18 | 0.23 | 0.28 | 0.38 | 0.40 | 0.30 | 0.31 | 6.00 | 0.36 |
| -72.3734446 | 18.725966 | 0.20 | 0.24 | 0.36 | 0.41 | 0.50 | 0.59 | 0.44 | 0.30 | 6.00 | 0.16 |
| -74.10872024 | 18.57747449 | 0.02 | 0.03 | 0.07 | 0.06 | 0.34 | 0.24 | 0.13 | 0.30 | 6.00 | 0.33 |
| -72.08427691 | 19.69210409 | 0.10 | 0.12 | 0.17 | 0.19 | 0.28 | 0.31 | 0.18 | 0.30 | 6.00 | 0.07 |
| -72.33841031 | 18.4820734 | 0.24 | 0.30 | 0.42 | 0.50 | 0.65 | 0.73 | 0.53 | 0.30 | 6.00 | 0.47 |
| -72.37775651 | 18.48557683 | 0.06 | 0.07 | 0.12 | 0.12 | 0.37 | 0.26 | 0.16 | 0.30 | 6.00 | 0.12 |
| -72.08427691 | 19.69264308 | 0.08 | 0.09 | 0.14 | 0.15 | 0.24 | 0.19 | 0.08 | 0.30 | 6.00 | 0.69 |
| -73.89393305 | 18.29261871 | 0.19 | 0.22 | 0.27 | 0.30 | 0.38 | 0.37 | 0.28 | 0.30 | 6.00 | 0.51 |
| -72.702228 | 19.91335914 | 0.11 | 0.13 | 0.17 | 0.18 | 0.24 | 0.25 | 0.19 | 0.30 | 6.00 | 0.49 |
| -73.42582096 | 18.38909777 | 0.11 | 0.13 | 0.18 | 0.20 | 0.30 | 0.27 | 0.20 | 0.30 | 6.00 | 0.01 |
| -73.85270038 | 18.27375409 | 0.17 | 0.20 | 0.25 | 0.28 | 0.40 | 0.39 | 0.30 | 0.30 | 6.00 | 0.11 |
| -72.33841031 | 18.48261239 | 0.25 | 0.30 | 0.41 | 0.49 | 0.60 | 0.71 | 0.53 | 0.30 | 6.00 | 0.32 |
| -74.10845074 | 18.57774398 | 0.04 | 0.06 | 0.10 | 0.10 | 0.31 | 0.26 | 0.15 | 0.30 | 6.00 | 0.32 |
| -72.32682204 | 18.49905156 | 0.21 | 0.24 | 0.30 | 0.34 | 0.50 | 0.62 | 0.44 | 0.30 | 6.00 | 0.25 |
| -72.37317511 | 18.72569651 | 0.19 | 0.24 | 0.35 | 0.41 | 0.50 | 0.59 | 0.44 | 0.30 | 6.00 | 0.22 |
| -73.87830237 | 18.18589885 | 0.15 | 0.18 | 0.23 | 0.26 | 0.36 | 0.33 | 0.25 | 0.30 | 6.00 | 0.17 |
| -73.89366356 | 18.29234921 | 0.19 | 0.22 | 0.27 | 0.30 | 0.38 | 0.37 | 0.28 | 0.30 | 6.00 | 0.31 |
| -73.89393305 | 18.2928882 | 0.19 | 0.22 | 0.27 | 0.31 | 0.38 | 0.37 | 0.28 | 0.30 | 6.00 | 0.38 |
| -72.08454641 | 19.6918346 | 0.13 | 0.16 | 0.21 | 0.25 | 0.36 | 0.46 | 0.33 | 0.30 | 6.00 | 0.59 |
| -72.37290561 | 18.72569651 | 0.21 | 0.25 | 0.37 | 0.44 | 0.52 | 0.61 | 0.45 | 0.30 | 6.00 | 0.22 |
| -72.32709153 | 18.49932106 | 0.22 | 0.26 | 0.32 | 0.36 | 0.54 | 0.66 | 0.46 | 0.30 | 6.00 | 0.01 |
| -73.87830237 | 18.18589885 | 0.15 | 0.18 | 0.23 | 0.26 | 0.36 | 0.33 | 0.25 | 0.30 | 6.00 | 0.22 |
| -72.23842781 | 18.68203839 | 0.29 | 0.35 | 0.46 | 0.54 | 0.70 | 0.77 | 0.57 | 0.30 | 6.00 | 0.11 |
| -74.10845074 | 18.57774398 | 0.04 | 0.06 | 0.10 | 0.10 | 0.31 | 0.26 | 0.15 | 0.30 | 6.00 | 0.53 |
| -72.33841031 | 18.48261239 | 0.25 | 0.30 | 0.41 | 0.49 | 0.60 | 0.71 | 0.53 | 0.30 | 6.00 | 0.47 |
| -73.60530435 | 18.05654145 | 0.05 | 0.06 | 0.11 | 0.11 | 0.35 | 0.33 | 0.19 | 0.30 | 6.00 | 0.69 |
| -73.25873431 | 18.87715246 | 0.15 | 0.19 | 0.26 | 0.25 | 0.12 | 0.06 | 0.04 | 0.30 | 6.00 | 0.09 |
| -72.98519731 | 18.87661348 | 0.17 | 0.21 | 0.30 | 0.38 | 0.52 | 0.42 | 0.24 | 0.30 | 6.00 | 0.31 |
| -72.15757944 | 18.69308766 | 0.18 | 0.22 | 0.29 | 0.35 | 0.50 | 0.55 | 0.42 | 0.30 | 6.00 | 0.54 |
| -73.25792583 | 18.87634398 | 0.14 | 0.17 | 0.22 | 0.25 | 0.23 | 0.16 | 0.09 | 0.30 | 6.00 | 0.46 |
| -73.25819532 | 18.87688297 | 0.13 | 0.16 | 0.21 | 0.23 | 0.34 | 0.25 | 0.13 | 0.30 | 6.00 | 0.44 |
| -72.32763052 | 18.49932106 | 0.23 | 0.26 | 0.33 | 0.39 | 0.56 | 0.66 | 0.44 | 0.30 | 6.00 | 0.03 |
| -72.98492782 | 18.87688297 | 0.14 | 0.16 | 0.23 | 0.29 | 0.47 | 0.35 | 0.18 | 0.30 | 6.00 | 0.21 |
| -73.89366356 | 18.29261871 | 0.18 | 0.21 | 0.26 | 0.29 | 0.38 | 0.38 | 0.28 | 0.30 | 6.00 | 0.32 |
| -71.97162817 | 18.46078333 | 0.20 | 0.24 | 0.34 | 0.40 | 0.56 | 0.57 | 0.40 | 0.30 | 6.00 | 0.41 |
| -73.89366356 | 18.29234921 | 0.19 | 0.22 | 0.27 | 0.30 | 0.38 | 0.37 | 0.28 | 0.30 | 6.00 | 0.53 |
| -72.32763052 | 18.49905156 | 0.28 | 0.32 | 0.41 | 0.47 | 0.66 | 0.76 | 0.53 | 0.30 | 6.00 | 0.17 |
| -72.37290561 | 18.72569651 | 0.21 | 0.25 | 0.37 | 0.44 | 0.52 | 0.61 | 0.45 | 0.30 | 6.00 | 0.70 |
| -72.33841031 | 18.4820734 | 0.24 | 0.30 | 0.42 | 0.50 | 0.65 | 0.73 | 0.53 | 0.30 | 6.00 | 0.54 |
| -72.32763052 | 18.49905156 | 0.28 | 0.32 | 0.41 | 0.47 | 0.66 | 0.76 | 0.53 | 0.30 | 6.00 | 0.39 |
| -74.01924803 | 18.55268098 | 0.22 | 0.25 | 0.31 | 0.35 | 0.44 | 0.42 | 0.30 | 0.30 | 6.00 | 0.11 |
| -72.0848159 | 19.69237359 | 0.11 | 0.13 | 0.19 | 0.23 | 0.35 | 0.44 | 0.33 | 0.30 | 6.00 | 0.48 |
| -72.32682204 | 18.49878207 | 0.27 | 0.31 | 0.39 | 0.46 | 0.64 | 0.75 | 0.52 | 0.30 | 6.00 | 0.27 |
| -74.10818125 | 18.57774398 | 0.09 | 0.11 | 0.15 | 0.18 | 0.30 | 0.30 | 0.20 | 0.30 | 6.00 | 0.38 |
| -72.37317511 | 18.72569651 | 0.19 | 0.24 | 0.35 | 0.41 | 0.50 | 0.59 | 0.44 | 0.30 | 6.00 | 0.42 |
| -73.42555146 | 18.38909777 | 0.11 | 0.13 | 0.17 | 0.20 | 0.33 | 0.32 | 0.23 | 0.30 | 6.00 | 0.47 |
| -72.98492782 | 18.87661348 | 0.17 | 0.21 | 0.28 | 0.36 | 0.51 | 0.42 | 0.24 | 0.30 | 6.00 | 0.51 |
| -72.06029189 | 19.68455824 | 0.05 | 0.06 | 0.09 | 0.11 | 0.17 | 0.14 | 0.07 | 0.30 | 6.00 | 0.04 |
| -74.10845074 | 18.57747449 | 0.06 | 0.07 | 0.13 | 0.14 | 0.29 | 0.29 | 0.19 | 0.30 | 6.00 | 0.18 |
| -72.3737141 | 18.72569651 | 0.18 | 0.22 | 0.34 | 0.40 | 0.49 | 0.56 | 0.41 | 0.30 | 6.00 | 0.43 |
| -73.42555146 | 18.38909777 | 0.11 | 0.13 | 0.17 | 0.20 | 0.33 | 0.32 | 0.23 | 0.30 | 6.00 | 0.13 |
| -74.10818125 | 18.57774398 | 0.09 | 0.11 | 0.15 | 0.18 | 0.30 | 0.30 | 0.20 | 0.30 | 6.00 | 0.36 |
| -72.32709153 | 18.49878207 | 0.25 | 0.29 | 0.36 | 0.41 | 0.57 | 0.67 | 0.47 | 0.30 | 6.00 | 0.11 |
| -73.87803287 | 18.18643784 | 0.11 | 0.14 | 0.19 | 0.21 | 0.35 | 0.34 | 0.26 | 0.30 | 6.00 | 0.51 |
| -72.98519731 | 18.87688297 | 0.15 | 0.18 | 0.26 | 0.32 | 0.49 | 0.37 | 0.20 | 0.30 | 6.00 | 0.51 |
| -72.33814081 | 18.4820734 | 0.26 | 0.32 | 0.43 | 0.51 | 0.65 | 0.74 | 0.55 | 0.30 | 6.00 | 0.09 |
| -73.20833883 | 19.90204037 | 0.13 | 0.15 | 0.19 | 0.19 | 0.19 | 0.19 | 0.14 | 0.30 | 6.00 | 0.41 |
| -74.10845074 | 18.57720499 | 0.08 | 0.10 | 0.15 | 0.16 | 0.30 | 0.31 | 0.21 | 0.30 | 6.00 | 0.01 |
| -72.32736103 | 18.49959055 | 0.21 | 0.24 | 0.30 | 0.36 | 0.52 | 0.64 | 0.42 | 0.30 | 6.00 | 0.09 |
| -74.10818125 | 18.57801348 | 0.06 | 0.08 | 0.13 | 0.14 | 0.31 | 0.30 | 0.19 | 0.30 | 6.00 | 0.04 |
| -72.32709153 | 18.49878207 | 0.25 | 0.29 | 0.36 | 0.41 | 0.57 | 0.67 | 0.47 | 0.30 | 6.00 | 0.05 |
| -72.33814081 | 18.4823429 | 0.27 | 0.33 | 0.43 | 0.51 | 0.64 | 0.73 | 0.55 | 0.30 | 6.00 | 0.33 |
| -73.85270038 | 18.27375409 | 0.17 | 0.20 | 0.25 | 0.28 | 0.40 | 0.39 | 0.30 | 0.30 | 6.00 | 0.02 |
| -72.15704045 | 18.69335716 | 0.10 | 0.13 | 0.18 | 0.21 | 0.41 | 0.44 | 0.30 | 0.30 | 6.00 | 0.32 |
| -72.3734446 | 18.725966 | 0.20 | 0.24 | 0.36 | 0.41 | 0.50 | 0.59 | 0.44 | 0.30 | 6.00 | 0.48 |
| -73.42609045 | 18.38882828 | 0.12 | 0.14 | 0.19 | 0.22 | 0.34 | 0.32 | 0.24 | 0.30 | 6.00 | 0.09 |
| -72.3386798 | 18.48261239 | 0.24 | 0.29 | 0.40 | 0.47 | 0.59 | 0.70 | 0.51 | 0.30 | 6.00 | 0.51 |
| -72.3386798 | 18.48261239 | 0.24 | 0.29 | 0.40 | 0.47 | 0.59 | 0.70 | 0.51 | 0.30 | 6.00 | 0.58 |
| -72.33841031 | 18.4823429 | 0.26 | 0.31 | 0.43 | 0.50 | 0.64 | 0.74 | 0.56 | 0.30 | 6.00 | 0.31 |
| -72.25971789 | 18.67961293 | 0.30 | 0.36 | 0.46 | 0.54 | 0.65 | 0.71 | 0.54 | 0.30 | 6.00 | 0.13 |
| -73.42582096 | 18.38909777 | 0.11 | 0.13 | 0.18 | 0.20 | 0.30 | 0.27 | 0.20 | 0.30 | 6.00 | 0.35 |
| -72.0600224 | 19.68509723 | 0.06 | 0.07 | 0.11 | 0.11 | 0.18 | 0.14 | 0.07 | 0.30 | 6.00 | 0.26 |
| -74.10818125 | 18.57774398 | 0.09 | 0.11 | 0.15 | 0.18 | 0.30 | 0.30 | 0.20 | 0.30 | 6.00 | 0.42 |
| -72.08454641 | 19.6918346 | 0.13 | 0.16 | 0.21 | 0.25 | 0.36 | 0.46 | 0.33 | 0.30 | 6.00 | 0.31 |
| -72.08454641 | 19.69264308 | 0.10 | 0.13 | 0.17 | 0.20 | 0.28 | 0.27 | 0.16 | 0.30 | 6.00 | 0.33 |
| -72.98519731 | 18.87688297 | 0.15 | 0.18 | 0.26 | 0.32 | 0.49 | 0.37 | 0.20 | 0.30 | 6.00 | 0.48 |
| -72.95339695 | 19.6271559 | 0.19 | 0.23 | 0.31 | 0.36 | 0.47 | 0.58 | 0.48 | 0.31 | 6.00 | 0.54 |
| -72.702228 | 19.91362864 | 0.10 | 0.11 | 0.16 | 0.18 | 0.25 | 0.26 | 0.19 | 0.30 | 6.00 | 0.33 |
| -73.89339406 | 18.29234921 | 0.19 | 0.22 | 0.27 | 0.30 | 0.38 | 0.38 | 0.28 | 0.30 | 6.00 | 0.50 |
| -74.10845074 | 18.57747449 | 0.06 | 0.07 | 0.13 | 0.14 | 0.29 | 0.29 | 0.19 | 0.30 | 6.00 | 0.54 |
| -72.98519731 | 18.87634398 | 0.19 | 0.22 | 0.32 | 0.41 | 0.55 | 0.47 | 0.28 | 0.30 | 6.00 | 0.35 |
| -72.15757944 | 18.69308766 | 0.18 | 0.22 | 0.29 | 0.35 | 0.50 | 0.55 | 0.42 | 0.30 | 6.00 | 0.17 |
| -73.19567258 | 19.89638099 | 0.11 | 0.13 | 0.19 | 0.21 | 0.33 | 0.33 | 0.23 | 0.30 | 6.00 | 0.32 |
| -72.0848159 | 19.69237359 | 0.11 | 0.13 | 0.19 | 0.23 | 0.35 | 0.44 | 0.33 | 0.30 | 6.00 | 0.03 |
| -72.98492782 | 18.87634398 | 0.18 | 0.21 | 0.30 | 0.37 | 0.52 | 0.45 | 0.27 | 0.30 | 6.00 | 0.64 |
| -72.32709153 | 18.49905156 | 0.20 | 0.22 | 0.27 | 0.32 | 0.50 | 0.65 | 0.43 | 0.30 | 6.00 | 0.61 |
| -72.3734446 | 18.72569651 | 0.19 | 0.23 | 0.35 | 0.42 | 0.49 | 0.58 | 0.43 | 0.30 | 6.00 | 0.15 |
| -73.87479894 | 18.37346708 | 0.10 | 0.12 | 0.17 | 0.19 | 0.40 | 0.37 | 0.26 | 0.30 | 6.00 | 0.68 |
| -73.36060327 | 19.84248207 | 0.11 | 0.13 | 0.15 | 0.15 | 0.24 | 0.23 | 0.13 | 0.30 | 6.00 | 0.20 |
| -73.42635995 | 18.38882828 | 0.10 | 0.12 | 0.17 | 0.19 | 0.29 | 0.25 | 0.18 | 0.30 | 6.00 | 0.00 |
| -73.42609045 | 18.38882828 | 0.12 | 0.14 | 0.19 | 0.22 | 0.34 | 0.32 | 0.24 | 0.30 | 6.00 | 0.17 |
| -72.23842781 | 18.68230788 | 0.24 | 0.28 | 0.38 | 0.45 | 0.62 | 0.70 | 0.52 | 0.30 | 6.00 | 0.49 |
| -72.98519731 | 18.87634398 | 0.19 | 0.22 | 0.32 | 0.41 | 0.55 | 0.47 | 0.28 | 0.30 | 6.00 | 0.23 |
| -73.60530435 | 18.05627196 | 0.08 | 0.10 | 0.15 | 0.16 | 0.39 | 0.40 | 0.25 | 0.30 | 6.00 | 0.07 |
| -72.08427691 | 19.6918346 | 0.11 | 0.13 | 0.18 | 0.21 | 0.30 | 0.32 | 0.21 | 0.30 | 6.00 | 0.67 |
| -72.0600224 | 19.68482774 | 0.06 | 0.07 | 0.11 | 0.11 | 0.18 | 0.15 | 0.07 | 0.30 | 6.00 | 0.18 |
| -72.37775651 | 18.48530734 | 0.08 | 0.09 | 0.15 | 0.18 | 0.38 | 0.29 | 0.17 | 0.30 | 6.00 | 0.00 |
| -73.91333666 | 18.30717142 | 0.07 | 0.09 | 0.13 | 0.13 | 0.34 | 0.28 | 0.17 | 0.30 | 6.00 | 0.25 |
| -73.87803287 | 18.18643784 | 0.11 | 0.14 | 0.19 | 0.21 | 0.35 | 0.34 | 0.26 | 0.30 | 6.00 | 0.16 |
| -72.32709153 | 18.49932106 | 0.22 | 0.26 | 0.32 | 0.36 | 0.54 | 0.66 | 0.46 | 0.30 | 6.00 | 0.27 |
| -72.3737141 | 18.725966 | 0.19 | 0.24 | 0.35 | 0.41 | 0.49 | 0.56 | 0.40 | 0.30 | 6.00 | 0.23 |
| -71.96677727 | 18.427366 | 0.23 | 0.27 | 0.34 | 0.39 | 0.51 | 0.50 | 0.37 | 0.30 | 6.00 | 0.22 |
| -72.37748702 | 18.48557683 | 0.06 | 0.08 | 0.13 | 0.14 | 0.37 | 0.27 | 0.16 | 0.30 | 6.00 | 0.16 |
| -73.89393305 | 18.2928882 | 0.19 | 0.22 | 0.27 | 0.31 | 0.38 | 0.37 | 0.28 | 0.30 | 6.00 | 0.02 |
| -72.33841031 | 18.4820734 | 0.24 | 0.30 | 0.42 | 0.50 | 0.65 | 0.73 | 0.53 | 0.30 | 6.00 | 0.08 |
| -73.25819532 | 18.87715246 | 0.09 | 0.11 | 0.15 | 0.16 | 0.33 | 0.21 | 0.10 | 0.30 | 6.00 | 0.56 |
| -73.89339406 | 18.29207972 | 0.19 | 0.22 | 0.27 | 0.30 | 0.38 | 0.38 | 0.28 | 0.30 | 6.00 | 0.24 |
| -71.97162817 | 18.46078333 | 0.20 | 0.24 | 0.34 | 0.40 | 0.56 | 0.57 | 0.40 | 0.30 | 6.00 | 0.53 |
| -72.08454641 | 19.6915651 | 0.11 | 0.14 | 0.19 | 0.22 | 0.33 | 0.40 | 0.31 | 0.30 | 6.00 | 0.18 |
| -71.96650778 | 18.427366 | 0.24 | 0.28 | 0.35 | 0.40 | 0.51 | 0.52 | 0.38 | 0.30 | 6.00 | 0.03 |
| -73.89366356 | 18.2928882 | 0.18 | 0.21 | 0.26 | 0.30 | 0.38 | 0.38 | 0.28 | 0.30 | 6.00 | 0.11 |
| -72.32736103 | 18.49905156 | 0.25 | 0.29 | 0.37 | 0.44 | 0.63 | 0.73 | 0.50 | 0.30 | 6.00 | 0.45 |
| -72.33894929 | 18.4820734 | 0.19 | 0.23 | 0.31 | 0.36 | 0.48 | 0.56 | 0.41 | 0.30 | 6.00 | 0.54 |
| -72.0597529 | 19.68482774 | 0.06 | 0.08 | 0.11 | 0.12 | 0.19 | 0.17 | 0.08 | 0.30 | 6.00 | 0.14 |
| -72.32736103 | 18.49932106 | 0.24 | 0.28 | 0.35 | 0.39 | 0.53 | 0.67 | 0.49 | 0.30 | 6.00 | 0.12 |
| -72.37290561 | 18.72542701 | 0.20 | 0.24 | 0.36 | 0.42 | 0.50 | 0.60 | 0.44 | 0.30 | 6.00 | 0.01 |
| -72.25971789 | 18.67934344 | 0.29 | 0.35 | 0.46 | 0.53 | 0.64 | 0.68 | 0.53 | 0.30 | 6.00 | 0.15 |
| -73.25819532 | 18.87688297 | 0.13 | 0.16 | 0.21 | 0.23 | 0.34 | 0.25 | 0.13 | 0.30 | 6.00 | 0.11 |
| -72.15730994 | 18.69308766 | 0.19 | 0.23 | 0.31 | 0.36 | 0.52 | 0.55 | 0.42 | 0.30 | 6.00 | 0.56 |
| -72.25567547 | 18.6814994 | 0.14 | 0.17 | 0.25 | 0.27 | 0.39 | 0.43 | 0.31 | 0.30 | 6.00 | 0.49 |
| -74.01897854 | 18.55295048 | 0.22 | 0.25 | 0.31 | 0.33 | 0.43 | 0.42 | 0.30 | 0.30 | 6.00 | 0.44 |
| -72.33841031 | 18.4820734 | 0.24 | 0.30 | 0.42 | 0.50 | 0.65 | 0.73 | 0.53 | 0.30 | 6.00 | 0.63 |
| -74.10818125 | 18.57774398 | 0.09 | 0.11 | 0.15 | 0.18 | 0.30 | 0.30 | 0.20 | 0.30 | 6.00 | 0.37 |
| -72.08427691 | 19.69264308 | 0.08 | 0.09 | 0.14 | 0.15 | 0.24 | 0.19 | 0.08 | 0.30 | 6.00 | 0.19 |
| -71.96677727 | 18.427366 | 0.23 | 0.27 | 0.34 | 0.39 | 0.51 | 0.50 | 0.37 | 0.30 | 6.00 | 0.22 |
| -74.01897854 | 18.55295048 | 0.22 | 0.25 | 0.31 | 0.33 | 0.43 | 0.42 | 0.30 | 0.30 | 6.00 | 0.43 |
| -72.33841031 | 18.4823429 | 0.26 | 0.31 | 0.43 | 0.50 | 0.64 | 0.74 | 0.56 | 0.30 | 6.00 | 0.06 |
| -72.08454641 | 19.69237359 | 0.12 | 0.15 | 0.21 | 0.25 | 0.35 | 0.38 | 0.26 | 0.30 | 6.00 | 0.59 |
| -72.58176392 | 18.5222281 | 0.10 | 0.12 | 0.16 | 0.18 | 0.28 | 0.27 | 0.20 | 0.30 | 6.00 | 0.67 |
| -72.3386798 | 18.4820734 | 0.21 | 0.26 | 0.37 | 0.41 | 0.58 | 0.66 | 0.48 | 0.30 | 6.00 | 0.68 |
| -73.15390092 | 19.62931185 | 0.21 | 0.25 | 0.31 | 0.36 | 0.47 | 0.52 | 0.36 | 0.30 | 6.00 | 0.22 |
| -72.98492782 | 18.87634398 | 0.18 | 0.21 | 0.30 | 0.37 | 0.52 | 0.45 | 0.27 | 0.30 | 6.00 | 0.63 |
| -73.60476536 | 18.05654145 | 0.04 | 0.06 | 0.10 | 0.09 | 0.35 | 0.29 | 0.16 | 0.30 | 6.00 | 0.01 |
| -73.89366356 | 18.29234921 | 0.19 | 0.22 | 0.27 | 0.30 | 0.38 | 0.37 | 0.28 | 0.30 | 6.00 | 0.15 |
| -71.97216716 | 18.46078333 | 0.20 | 0.24 | 0.34 | 0.41 | 0.57 | 0.61 | 0.43 | 0.30 | 6.00 | 0.54 |
| -73.42528197 | 18.38936727 | 0.07 | 0.09 | 0.13 | 0.16 | 0.26 | 0.24 | 0.15 | 0.30 | 6.00 | 0.01 |
| -73.91333666 | 18.30717142 | 0.07 | 0.09 | 0.13 | 0.13 | 0.34 | 0.28 | 0.17 | 0.30 | 6.00 | 0.44 |
| -73.89339406 | 18.29234921 | 0.19 | 0.22 | 0.27 | 0.30 | 0.38 | 0.38 | 0.28 | 0.30 | 6.00 | 0.01 |
| -73.25873431 | 18.87715246 | 0.15 | 0.19 | 0.26 | 0.25 | 0.12 | 0.06 | 0.04 | 0.30 | 6.00 | 0.31 |
| -73.42582096 | 18.38909777 | 0.11 | 0.13 | 0.18 | 0.20 | 0.30 | 0.27 | 0.20 | 0.30 | 6.00 | 0.65 |
| -73.89339406 | 18.29207972 | 0.19 | 0.22 | 0.27 | 0.30 | 0.38 | 0.38 | 0.28 | 0.30 | 6.00 | 0.33 |
| -72.702228 | 19.91362864 | 0.10 | 0.11 | 0.16 | 0.18 | 0.25 | 0.26 | 0.19 | 0.30 | 6.00 | 0.10 |
| -72.08454641 | 19.69210409 | 0.13 | 0.16 | 0.21 | 0.26 | 0.37 | 0.45 | 0.32 | 0.30 | 6.00 | 0.64 |
| -72.0600224 | 19.68482774 | 0.06 | 0.07 | 0.11 | 0.11 | 0.18 | 0.15 | 0.07 | 0.30 | 6.00 | 0.55 |
| -73.89339406 | 18.29234921 | 0.19 | 0.22 | 0.27 | 0.30 | 0.38 | 0.38 | 0.28 | 0.30 | 6.00 | 0.08 |
| -72.08427691 | 19.69210409 | 0.10 | 0.12 | 0.17 | 0.19 | 0.28 | 0.31 | 0.18 | 0.30 | 6.00 | 0.59 |
| -72.81460724 | 19.50965626 | 0.12 | 0.14 | 0.19 | 0.23 | 0.31 | 0.34 | 0.27 | 0.31 | 6.00 | 0.43 |
| -72.32682204 | 18.49905156 | 0.21 | 0.24 | 0.30 | 0.34 | 0.50 | 0.62 | 0.44 | 0.30 | 6.00 | 0.35 |
| -72.32763052 | 18.49959055 | 0.25 | 0.29 | 0.36 | 0.40 | 0.55 | 0.60 | 0.41 | 0.30 | 6.00 | 0.46 |
| -72.58176392 | 18.5222281 | 0.10 | 0.12 | 0.16 | 0.18 | 0.28 | 0.27 | 0.20 | 0.30 | 6.00 | 0.44 |
| -73.25873431 | 18.87742196 | 0.15 | 0.18 | 0.25 | 0.27 | 0.29 | 0.18 | 0.09 | 0.30 | 6.00 | 0.49 |
| -73.89366356 | 18.29234921 | 0.19 | 0.22 | 0.27 | 0.30 | 0.38 | 0.37 | 0.28 | 0.30 | 6.00 | 0.58 |
| -73.91360616 | 18.30690192 | 0.18 | 0.20 | 0.26 | 0.29 | 0.37 | 0.36 | 0.26 | 0.30 | 6.00 | 0.53 |
| -72.08427691 | 19.69237359 | 0.10 | 0.11 | 0.16 | 0.17 | 0.26 | 0.24 | 0.13 | 0.30 | 6.00 | 0.41 |
| -72.49417818 | 18.5168382 | 0.10 | 0.12 | 0.18 | 0.21 | 0.40 | 0.42 | 0.28 | 0.30 | 6.00 | 0.42 |
| -73.89366356 | 18.2928882 | 0.18 | 0.21 | 0.26 | 0.30 | 0.38 | 0.38 | 0.28 | 0.30 | 6.00 | 0.61 |
| -72.08427691 | 19.69210409 | 0.10 | 0.12 | 0.17 | 0.19 | 0.28 | 0.31 | 0.18 | 0.30 | 6.00 | 0.00 |
| -72.81460724 | 19.50965626 | 0.12 | 0.14 | 0.19 | 0.23 | 0.31 | 0.34 | 0.27 | 0.31 | 6.00 | 0.63 |
| -73.89366356 | 18.29234921 | 0.19 | 0.22 | 0.27 | 0.30 | 0.38 | 0.37 | 0.28 | 0.30 | 6.00 | 0.42 |
| -72.37317511 | 18.725966 | 0.21 | 0.25 | 0.36 | 0.42 | 0.51 | 0.61 | 0.45 | 0.30 | 6.00 | 0.58 |
| -73.42635995 | 18.38855878 | 0.12 | 0.14 | 0.19 | 0.22 | 0.34 | 0.33 | 0.24 | 0.30 | 6.00 | 0.01 |
| -72.32709153 | 18.49851257 | 0.26 | 0.30 | 0.39 | 0.45 | 0.63 | 0.73 | 0.51 | 0.30 | 6.00 | 0.07 |
| -73.42528197 | 18.38936727 | 0.07 | 0.09 | 0.13 | 0.16 | 0.26 | 0.24 | 0.15 | 0.30 | 6.00 | 0.29 |
| -72.3386798 | 18.4820734 | 0.21 | 0.26 | 0.37 | 0.41 | 0.58 | 0.66 | 0.48 | 0.30 | 6.00 | 0.26 |
| -72.33894929 | 18.4820734 | 0.19 | 0.23 | 0.31 | 0.36 | 0.48 | 0.56 | 0.41 | 0.30 | 6.00 | 0.19 |
| -74.10818125 | 18.57828297 | 0.05 | 0.06 | 0.11 | 0.11 | 0.33 | 0.32 | 0.18 | 0.30 | 6.00 | 0.66 |
| -71.96650778 | 18.427366 | 0.24 | 0.28 | 0.35 | 0.40 | 0.51 | 0.52 | 0.38 | 0.30 | 6.00 | 0.44 |
| -73.85296987 | 18.27348459 | 0.22 | 0.24 | 0.31 | 0.35 | 0.42 | 0.42 | 0.30 | 0.30 | 6.00 | 0.65 |
| -72.33814081 | 18.4823429 | 0.27 | 0.33 | 0.43 | 0.51 | 0.64 | 0.73 | 0.55 | 0.30 | 6.00 | 0.22 |
| -72.32763052 | 18.49932106 | 0.23 | 0.26 | 0.33 | 0.39 | 0.56 | 0.66 | 0.44 | 0.30 | 6.00 | 0.44 |
| -72.08454641 | 19.69264308 | 0.10 | 0.13 | 0.17 | 0.20 | 0.28 | 0.27 | 0.16 | 0.30 | 6.00 | 0.36 |
| -72.25971789 | 18.67934344 | 0.29 | 0.35 | 0.46 | 0.53 | 0.64 | 0.68 | 0.53 | 0.30 | 6.00 | 0.61 |
| -72.06029189 | 19.68482774 | 0.06 | 0.07 | 0.10 | 0.10 | 0.17 | 0.13 | 0.06 | 0.30 | 6.00 | 0.31 |
| -72.3386798 | 18.4823429 | 0.23 | 0.28 | 0.39 | 0.46 | 0.59 | 0.71 | 0.52 | 0.30 | 6.00 | 0.01 |
| -72.32736103 | 18.49878207 | 0.28 | 0.33 | 0.41 | 0.48 | 0.67 | 0.78 | 0.55 | 0.30 | 6.00 | 0.64 |
| -72.98492782 | 18.87661348 | 0.17 | 0.21 | 0.28 | 0.36 | 0.51 | 0.42 | 0.24 | 0.30 | 6.00 | 0.14 |
| -72.08454641 | 19.69237359 | 0.12 | 0.15 | 0.21 | 0.25 | 0.35 | 0.38 | 0.26 | 0.30 | 6.00 | 0.56 |
| -74.01924803 | 18.55348947 | 0.18 | 0.21 | 0.26 | 0.29 | 0.40 | 0.41 | 0.30 | 0.30 | 6.00 | 0.47 |
| -74.10845074 | 18.57801348 | 0.03 | 0.04 | 0.08 | 0.08 | 0.31 | 0.26 | 0.14 | 0.30 | 6.00 | 0.13 |
| -72.33894929 | 18.4823429 | 0.22 | 0.27 | 0.37 | 0.44 | 0.56 | 0.66 | 0.48 | 0.30 | 6.00 | 0.68 |
| -72.49417818 | 18.51656871 | 0.16 | 0.19 | 0.26 | 0.32 | 0.48 | 0.51 | 0.35 | 0.30 | 6.00 | 0.12 |
| -71.97189767 | 18.46078333 | 0.19 | 0.23 | 0.33 | 0.40 | 0.56 | 0.58 | 0.40 | 0.30 | 6.00 | 0.69 |
| -72.58176392 | 18.5222281 | 0.10 | 0.12 | 0.16 | 0.18 | 0.28 | 0.27 | 0.20 | 0.30 | 6.00 | 0.12 |
| -72.7019585 | 19.91362864 | 0.09 | 0.11 | 0.15 | 0.17 | 0.25 | 0.25 | 0.19 | 0.30 | 6.00 | 0.35 |
| -71.96677727 | 18.427366 | 0.23 | 0.27 | 0.34 | 0.39 | 0.51 | 0.50 | 0.37 | 0.30 | 6.00 | 0.09 |
| -72.08454641 | 19.69210409 | 0.13 | 0.16 | 0.21 | 0.26 | 0.37 | 0.45 | 0.32 | 0.30 | 6.00 | 0.02 |
| -73.60476536 | 18.05654145 | 0.04 | 0.06 | 0.10 | 0.09 | 0.35 | 0.29 | 0.16 | 0.30 | 6.00 | 0.52 |
| -73.36006428 | 19.84221257 | 0.11 | 0.13 | 0.17 | 0.21 | 0.32 | 0.32 | 0.18 | 0.30 | 6.00 | 0.01 |
| -73.25846482 | 18.87715246 | 0.14 | 0.16 | 0.22 | 0.24 | 0.31 | 0.19 | 0.11 | 0.30 | 6.00 | 0.48 |
| -73.87479894 | 18.37346708 | 0.10 | 0.12 | 0.17 | 0.19 | 0.40 | 0.37 | 0.26 | 0.30 | 6.00 | 0.04 |
| -72.3734446 | 18.72569651 | 0.19 | 0.23 | 0.35 | 0.42 | 0.49 | 0.58 | 0.43 | 0.30 | 6.00 | 0.29 |
| -72.98492782 | 18.87607449 | 0.18 | 0.21 | 0.30 | 0.38 | 0.53 | 0.49 | 0.30 | 0.30 | 6.00 | 0.07 |
| -71.96650778 | 18.4276355 | 0.23 | 0.27 | 0.34 | 0.38 | 0.50 | 0.51 | 0.38 | 0.30 | 6.00 | 0.56 |
| -73.87803287 | 18.18643784 | 0.11 | 0.14 | 0.19 | 0.21 | 0.35 | 0.34 | 0.26 | 0.30 | 6.00 | 0.65 |
| -72.15704045 | 18.69335716 | 0.10 | 0.13 | 0.18 | 0.21 | 0.41 | 0.44 | 0.30 | 0.30 | 6.00 | 0.37 |
| -73.25846482 | 18.87742196 | 0.12 | 0.14 | 0.19 | 0.21 | 0.34 | 0.22 | 0.11 | 0.30 | 6.00 | 0.07 |
| -73.25846482 | 18.87715246 | 0.14 | 0.16 | 0.22 | 0.24 | 0.31 | 0.19 | 0.11 | 0.30 | 6.00 | 0.55 |
| -74.10845074 | 18.57774398 | 0.04 | 0.06 | 0.10 | 0.10 | 0.31 | 0.26 | 0.15 | 0.30 | 6.00 | 0.03 |
| -72.33841031 | 18.48261239 | 0.25 | 0.30 | 0.41 | 0.49 | 0.60 | 0.71 | 0.53 | 0.30 | 6.00 | 0.18 |
| -73.25873431 | 18.87742196 | 0.15 | 0.18 | 0.25 | 0.27 | 0.29 | 0.18 | 0.09 | 0.30 | 6.00 | 0.08 |
| -72.32709153 | 18.49932106 | 0.22 | 0.26 | 0.32 | 0.36 | 0.54 | 0.66 | 0.46 | 0.30 | 6.00 | 0.09 |
| -73.87830237 | 18.18589885 | 0.15 | 0.18 | 0.23 | 0.26 | 0.36 | 0.33 | 0.25 | 0.30 | 6.00 | 0.28 |
| -72.32682204 | 18.49878207 | 0.27 | 0.31 | 0.39 | 0.46 | 0.64 | 0.75 | 0.52 | 0.30 | 6.00 | 0.54 |
| -73.87803287 | 18.18616835 | 0.14 | 0.16 | 0.21 | 0.24 | 0.35 | 0.33 | 0.25 | 0.30 | 6.00 | 0.54 |
| -71.98213846 | 18.58070842 | 0.03 | 0.04 | 0.06 | 0.03 | 0.01 | 0.01 | 0.01 | 0.30 | 7.00 | 0.52 |
| -72.32304911 | 19.71716709 | 0.03 | 0.04 | 0.05 | 0.02 | 0.01 | 0.01 | 0.01 | 0.30 | 7.00 | 0.20 |
| -72.02687456 | 18.58852376 | 0.03 | 0.04 | 0.07 | 0.03 | 0.02 | 0.01 | 0.01 | 0.30 | 7.00 | 0.50 |
| -72.65937836 | 19.36547665 | 0.04 | 0.04 | 0.07 | 0.05 | 0.01 | 0.00 | 0.00 | 0.30 | 7.00 | 0.31 |
| -71.98941482 | 18.57558802 | 0.03 | 0.04 | 0.06 | 0.03 | 0.01 | 0.01 | 0.01 | 0.30 | 7.00 | 0.69 |
| -72.00854893 | 18.59418315 | 0.03 | 0.04 | 0.06 | 0.03 | 0.02 | 0.01 | 0.01 | 0.30 | 7.00 | 0.11 |
| -72.69306518 | 19.4129077 | 0.04 | 0.05 | 0.06 | 0.03 | 0.01 | 0.01 | 0.01 | 0.30 | 7.00 | 0.61 |
| -73.05688287 | 18.41335228 | 0.03 | 0.04 | 0.04 | 0.03 | 0.02 | 0.01 | 0.01 | 0.30 | 7.00 | 0.47 |
| -73.04852854 | 18.40284199 | 0.03 | 0.03 | 0.03 | 0.02 | 0.01 | 0.01 | 0.01 | 0.30 | 7.00 | 0.17 |
| -72.02040669 | 18.9051799 | 0.02 | 0.03 | 0.05 | 0.02 | 0.01 | 0.01 | 0.00 | 0.30 | 7.00 | 0.16 |
| -71.97890453 | 18.58555932 | 0.03 | 0.04 | 0.06 | 0.02 | 0.01 | 0.01 | 0.01 | 0.30 | 7.00 | 0.12 |
| -72.00774045 | 18.9103003 | 0.03 | 0.03 | 0.06 | 0.03 | 0.01 | 0.01 | 0.00 | 0.30 | 7.00 | 0.54 |
| -71.98213846 | 18.57343207 | 0.03 | 0.04 | 0.06 | 0.03 | 0.01 | 0.01 | 0.01 | 0.30 | 7.00 | 0.51 |
| -72.69576013 | 19.40940427 | 0.04 | 0.04 | 0.05 | 0.02 | 0.01 | 0.01 | 0.01 | 0.30 | 7.00 | 0.64 |
| -72.6620733 | 19.36278171 | 0.04 | 0.04 | 0.07 | 0.05 | 0.01 | 0.01 | 0.00 | 0.30 | 7.00 | 0.12 |
| -72.65910886 | 19.36008676 | 0.04 | 0.04 | 0.07 | 0.05 | 0.01 | 0.00 | 0.00 | 0.30 | 7.00 | 0.24 |
| -73.05149298 | 18.39879958 | 0.03 | 0.03 | 0.04 | 0.02 | 0.01 | 0.01 | 0.01 | 0.30 | 7.00 | 0.50 |
| -72.65776139 | 19.36493766 | 0.04 | 0.04 | 0.07 | 0.05 | 0.01 | 0.01 | 0.00 | 0.30 | 7.00 | 0.43 |
| -72.01798124 | 18.57855246 | 0.03 | 0.04 | 0.06 | 0.03 | 0.02 | 0.01 | 0.01 | 0.30 | 7.00 | 0.23 |
| -72.01798124 | 18.9000595 | 0.02 | 0.03 | 0.04 | 0.02 | 0.01 | 0.01 | 0.00 | 0.30 | 7.00 | 0.51 |
| -72.02444911 | 18.59364416 | 0.03 | 0.04 | 0.06 | 0.03 | 0.02 | 0.02 | 0.01 | 0.30 | 7.00 | 0.63 |
| -72.75424045 | 19.29594705 | 0.04 | 0.05 | 0.05 | 0.02 | 0.01 | 0.00 | 0.00 | 0.30 | 7.00 | 0.60 |
| -71.98860633 | 18.59445264 | 0.03 | 0.04 | 0.06 | 0.03 | 0.02 | 0.01 | 0.01 | 0.30 | 7.00 | 0.26 |
| -71.99291825 | 18.58744579 | 0.03 | 0.04 | 0.06 | 0.03 | 0.02 | 0.02 | 0.01 | 0.30 | 7.00 | 0.36 |
| -72.01205236 | 18.58043893 | 0.03 | 0.04 | 0.06 | 0.03 | 0.02 | 0.01 | 0.01 | 0.30 | 7.00 | 0.15 |
| -71.97998251 | 18.59256618 | 0.03 | 0.04 | 0.06 | 0.03 | 0.01 | 0.01 | 0.01 | 0.30 | 7.00 | 0.14 |
| -72.02633558 | 18.58097792 | 0.03 | 0.04 | 0.06 | 0.03 | 0.02 | 0.01 | 0.01 | 0.30 | 7.00 | 0.31 |
| -71.98752836 | 18.5869068 | 0.03 | 0.04 | 0.06 | 0.03 | 0.02 | 0.01 | 0.01 | 0.30 | 7.00 | 0.38 |
| -72.01178287 | 18.5914882 | 0.03 | 0.04 | 0.06 | 0.03 | 0.02 | 0.02 | 0.01 | 0.30 | 7.00 | 0.01 |
| -72.00908792 | 18.57451005 | 0.03 | 0.04 | 0.06 | 0.03 | 0.02 | 0.01 | 0.01 | 0.30 | 7.00 | 0.46 |
| -71.98132998 | 18.56938965 | 0.03 | 0.04 | 0.06 | 0.03 | 0.01 | 0.01 | 0.01 | 0.30 | 7.00 | 0.48 |
| -74.03676518 | 18.58259488 | 0.10 | 0.12 | 0.17 | 0.18 | 0.33 | 0.24 | 0.17 | 0.30 | 7.00 | 0.32 |
| -72.01205236 | 18.57154561 | 0.03 | 0.04 | 0.06 | 0.03 | 0.02 | 0.01 | 0.01 | 0.30 | 7.00 | 0.30 |
| -73.04745056 | 18.40688441 | 0.03 | 0.03 | 0.03 | 0.02 | 0.02 | 0.01 | 0.01 | 0.30 | 7.00 | 0.03 |
| -72.01339984 | 18.59606961 | 0.03 | 0.04 | 0.07 | 0.03 | 0.02 | 0.02 | 0.01 | 0.30 | 7.00 | 0.18 |
| -71.97620958 | 18.58340337 | 0.04 | 0.04 | 0.06 | 0.03 | 0.01 | 0.01 | 0.01 | 0.30 | 7.00 | 0.18 |
| -72.01286085 | 18.5866373 | 0.03 | 0.04 | 0.07 | 0.03 | 0.02 | 0.02 | 0.01 | 0.30 | 7.00 | 0.57 |
| -73.64492005 | 18.50929236 | 0.04 | 0.04 | 0.04 | 0.01 | 0.01 | 0.01 | 0.01 | 0.30 | 7.00 | 0.49 |
| -72.02417962 | 18.5869068 | 0.03 | 0.04 | 0.07 | 0.03 | 0.02 | 0.01 | 0.01 | 0.30 | 7.00 | 0.61 |
| -73.05445742 | 18.40068604 | 0.03 | 0.03 | 0.04 | 0.02 | 0.02 | 0.01 | 0.01 | 0.30 | 7.00 | 0.08 |
| -73.90875525 | 18.53354687 | 0.02 | 0.02 | 0.04 | 0.03 | 0.29 | 0.16 | 0.06 | 0.30 | 7.00 | 0.10 |
| -72.02444911 | 18.57882196 | 0.03 | 0.04 | 0.06 | 0.03 | 0.02 | 0.01 | 0.01 | 0.30 | 7.00 | 0.04 |
| -73.05445742 | 18.40607593 | 0.03 | 0.04 | 0.04 | 0.02 | 0.02 | 0.01 | 0.01 | 0.30 | 7.00 | 0.19 |
| -72.01690327 | 18.56588622 | 0.03 | 0.04 | 0.06 | 0.03 | 0.01 | 0.01 | 0.01 | 0.30 | 7.00 | 0.08 |
| -72.02391012 | 18.59067972 | 0.03 | 0.04 | 0.06 | 0.03 | 0.02 | 0.02 | 0.01 | 0.30 | 7.00 | 0.58 |
| -71.98887583 | 18.57289308 | 0.04 | 0.04 | 0.06 | 0.03 | 0.01 | 0.01 | 0.01 | 0.30 | 7.00 | 0.47 |
| -72.0050455 | 18.56938965 | 0.03 | 0.04 | 0.06 | 0.03 | 0.02 | 0.02 | 0.01 | 0.30 | 7.00 | 0.60 |
| -71.98132998 | 18.57127611 | 0.03 | 0.04 | 0.06 | 0.03 | 0.01 | 0.01 | 0.01 | 0.30 | 7.00 | 0.66 |
| -71.99264875 | 18.56777268 | 0.03 | 0.04 | 0.06 | 0.03 | 0.01 | 0.01 | 0.01 | 0.30 | 7.00 | 0.09 |
| -73.05122348 | 18.41443026 | 0.04 | 0.04 | 0.05 | 0.04 | 0.02 | 0.01 | 0.01 | 0.30 | 7.00 | 0.02 |
| -71.99615218 | 18.58771528 | 0.03 | 0.04 | 0.06 | 0.03 | 0.01 | 0.01 | 0.01 | 0.30 | 7.00 | 0.25 |
| -71.97944352 | 18.59364416 | 0.03 | 0.04 | 0.06 | 0.03 | 0.01 | 0.01 | 0.01 | 0.30 | 7.00 | 0.36 |
| -71.99076229 | 18.57747449 | 0.03 | 0.04 | 0.06 | 0.03 | 0.01 | 0.01 | 0.01 | 0.30 | 7.00 | 0.11 |
| -71.98159947 | 18.59067972 | 0.03 | 0.04 | 0.06 | 0.02 | 0.01 | 0.01 | 0.01 | 0.30 | 7.00 | 0.01 |
| -71.97647908 | 18.57989994 | 0.03 | 0.04 | 0.06 | 0.02 | 0.01 | 0.01 | 0.01 | 0.30 | 7.00 | 0.52 |
| -72.0150168 | 18.57316257 | 0.03 | 0.04 | 0.06 | 0.03 | 0.02 | 0.01 | 0.01 | 0.30 | 7.00 | 0.66 |
| -72.01124388 | 18.57073712 | 0.03 | 0.04 | 0.07 | 0.03 | 0.01 | 0.01 | 0.01 | 0.30 | 7.00 | 0.06 |
| -72.01744225 | 18.58421185 | 0.03 | 0.04 | 0.06 | 0.03 | 0.02 | 0.01 | 0.01 | 0.30 | 7.00 | 0.10 |
| -72.01178287 | 18.58771528 | 0.03 | 0.04 | 0.06 | 0.03 | 0.02 | 0.01 | 0.01 | 0.30 | 7.00 | 0.33 |
| -71.97486211 | 18.57451005 | 0.03 | 0.04 | 0.06 | 0.03 | 0.01 | 0.01 | 0.01 | 0.30 | 7.00 | 0.48 |
| -72.75046753 | 19.29971997 | 0.04 | 0.05 | 0.05 | 0.01 | 0.01 | 0.00 | 0.00 | 0.30 | 7.00 | 0.28 |
| -72.02121518 | 18.56373026 | 0.03 | 0.04 | 0.06 | 0.03 | 0.01 | 0.01 | 0.01 | 0.30 | 7.00 | 0.11 |
| -72.02256265 | 18.57504903 | 0.03 | 0.04 | 0.06 | 0.03 | 0.01 | 0.01 | 0.01 | 0.30 | 7.00 | 0.45 |
| -71.98159947 | 18.57424055 | 0.03 | 0.04 | 0.06 | 0.03 | 0.01 | 0.01 | 0.01 | 0.30 | 7.00 | 0.01 |
| -71.98132998 | 18.57370156 | 0.03 | 0.04 | 0.06 | 0.03 | 0.01 | 0.01 | 0.01 | 0.30 | 7.00 | 0.24 |
| -73.65300489 | 18.50902286 | 0.04 | 0.04 | 0.04 | 0.01 | 0.01 | 0.01 | 0.00 | 0.30 | 7.00 | 0.19 |
| -71.97378413 | 18.57046763 | 0.04 | 0.04 | 0.06 | 0.03 | 0.01 | 0.01 | 0.01 | 0.30 | 7.00 | 0.11 |
| -71.98348594 | 18.58879326 | 0.03 | 0.04 | 0.06 | 0.03 | 0.01 | 0.01 | 0.01 | 0.30 | 7.00 | 0.23 |
| -72.0201372 | 18.57235409 | 0.03 | 0.04 | 0.06 | 0.03 | 0.01 | 0.01 | 0.01 | 0.30 | 7.00 | 0.34 |
| -72.00127258 | 18.59580012 | 0.03 | 0.04 | 0.06 | 0.03 | 0.02 | 0.02 | 0.01 | 0.30 | 7.00 | 0.27 |
| -72.75639641 | 19.29945048 | 0.04 | 0.05 | 0.04 | 0.01 | 0.01 | 0.00 | 0.00 | 0.30 | 7.00 | 0.70 |
| -72.02040669 | 18.56453875 | 0.03 | 0.04 | 0.06 | 0.03 | 0.01 | 0.01 | 0.01 | 0.30 | 7.00 | 0.30 |
| -71.99992511 | 18.56858116 | 0.04 | 0.04 | 0.06 | 0.03 | 0.02 | 0.01 | 0.01 | 0.30 | 7.00 | 0.18 |
| -72.02121518 | 18.5914882 | 0.03 | 0.04 | 0.06 | 0.03 | 0.02 | 0.01 | 0.01 | 0.30 | 7.00 | 0.67 |
| -72.01690327 | 18.57235409 | 0.03 | 0.04 | 0.06 | 0.03 | 0.01 | 0.01 | 0.01 | 0.30 | 7.00 | 0.39 |
| -72.00720146 | 18.5869068 | 0.03 | 0.04 | 0.06 | 0.03 | 0.02 | 0.02 | 0.01 | 0.30 | 7.00 | 0.43 |
| -74.05994172 | 18.61196979 | 0.06 | 0.06 | 0.03 | 0.02 | 0.02 | 0.01 | 0.01 | 0.30 | 7.00 | 0.56 |
| -72.75397096 | 19.29244362 | 0.04 | 0.05 | 0.05 | 0.02 | 0.01 | 0.00 | 0.00 | 0.30 | 7.00 | 0.41 |
| -72.75639641 | 19.29486907 | 0.04 | 0.05 | 0.05 | 0.02 | 0.01 | 0.00 | 0.00 | 0.30 | 7.00 | 0.13 |
| -72.01986771 | 18.56750319 | 0.03 | 0.04 | 0.06 | 0.03 | 0.01 | 0.01 | 0.01 | 0.30 | 7.00 | 0.40 |
| -72.00558449 | 18.58744579 | 0.03 | 0.04 | 0.06 | 0.03 | 0.02 | 0.02 | 0.01 | 0.30 | 7.00 | 0.15 |
| -72.02202366 | 18.58367286 | 0.03 | 0.04 | 0.07 | 0.03 | 0.02 | 0.01 | 0.01 | 0.30 | 7.00 | 0.03 |
| -72.00127258 | 18.58043893 | 0.03 | 0.04 | 0.06 | 0.03 | 0.02 | 0.01 | 0.01 | 0.30 | 7.00 | 0.05 |
| -72.01097438 | 18.59067972 | 0.03 | 0.04 | 0.06 | 0.03 | 0.02 | 0.02 | 0.01 | 0.30 | 7.00 | 0.54 |
| -73.84569352 | 18.31417828 | 0.01 | 0.03 | 0.09 | 0.05 | 0.27 | 0.13 | 0.05 | 0.30 | 7.00 | 0.67 |
| -72.33032547 | 19.72606041 | 0.03 | 0.04 | 0.05 | 0.02 | 0.01 | 0.01 | 0.01 | 0.30 | 7.00 | 0.22 |
| -72.00154207 | 18.59310517 | 0.03 | 0.04 | 0.06 | 0.03 | 0.02 | 0.02 | 0.01 | 0.30 | 7.00 | 0.63 |
| -71.99615218 | 18.58070842 | 0.04 | 0.04 | 0.07 | 0.03 | 0.02 | 0.02 | 0.01 | 0.30 | 7.00 | 0.18 |
| -71.98779785 | 18.58636781 | 0.03 | 0.04 | 0.06 | 0.03 | 0.01 | 0.01 | 0.01 | 0.30 | 7.00 | 0.29 |
| -72.01636428 | 18.59121871 | 0.03 | 0.04 | 0.07 | 0.03 | 0.02 | 0.02 | 0.01 | 0.30 | 7.00 | 0.01 |
| -71.9953437 | 18.58825427 | 0.03 | 0.04 | 0.06 | 0.03 | 0.02 | 0.02 | 0.01 | 0.30 | 7.00 | 0.44 |
| -73.04987601 | 18.41227431 | 0.03 | 0.04 | 0.04 | 0.02 | 0.02 | 0.01 | 0.01 | 0.30 | 7.00 | 0.22 |
| -72.01366933 | 18.57424055 | 0.03 | 0.04 | 0.06 | 0.03 | 0.02 | 0.01 | 0.01 | 0.30 | 7.00 | 0.10 |
| -71.99588269 | 18.58340337 | 0.03 | 0.04 | 0.07 | 0.03 | 0.02 | 0.02 | 0.01 | 0.30 | 7.00 | 0.68 |
| -72.01663377 | 18.58987124 | 0.03 | 0.04 | 0.06 | 0.03 | 0.02 | 0.02 | 0.01 | 0.30 | 7.00 | 0.60 |
| -71.98240796 | 18.59337467 | 0.03 | 0.04 | 0.06 | 0.03 | 0.01 | 0.01 | 0.01 | 0.30 | 7.00 | 0.22 |
| -72.0001946 | 18.59418315 | 0.03 | 0.04 | 0.06 | 0.03 | 0.02 | 0.02 | 0.01 | 0.30 | 7.00 | 0.60 |
| -72.00612348 | 18.57504903 | 0.03 | 0.04 | 0.06 | 0.03 | 0.02 | 0.01 | 0.01 | 0.30 | 7.00 | 0.46 |
| -71.98914532 | 18.56804218 | 0.03 | 0.04 | 0.06 | 0.03 | 0.01 | 0.01 | 0.01 | 0.30 | 7.00 | 0.58 |
| -73.04745056 | 18.40014705 | 0.03 | 0.03 | 0.03 | 0.02 | 0.01 | 0.01 | 0.01 | 0.30 | 7.00 | 0.55 |
| -72.02391012 | 18.58528983 | 0.03 | 0.04 | 0.07 | 0.03 | 0.02 | 0.01 | 0.01 | 0.30 | 7.00 | 0.14 |
| -71.97809605 | 18.57558802 | 0.03 | 0.04 | 0.06 | 0.02 | 0.01 | 0.01 | 0.01 | 0.30 | 7.00 | 0.06 |
| -72.00396753 | 18.57855246 | 0.03 | 0.04 | 0.06 | 0.03 | 0.02 | 0.01 | 0.01 | 0.30 | 7.00 | 0.63 |
| -72.01393882 | 18.5666947 | 0.03 | 0.04 | 0.06 | 0.03 | 0.02 | 0.01 | 0.01 | 0.30 | 7.00 | 0.05 |
| -72.00396753 | 18.57909145 | 0.03 | 0.04 | 0.06 | 0.03 | 0.02 | 0.01 | 0.01 | 0.30 | 7.00 | 0.44 |
| -72.02444911 | 18.57801348 | 0.03 | 0.04 | 0.06 | 0.03 | 0.01 | 0.01 | 0.01 | 0.30 | 7.00 | 0.09 |
| -72.00423702 | 18.59606961 | 0.03 | 0.04 | 0.06 | 0.03 | 0.02 | 0.02 | 0.01 | 0.30 | 7.00 | 0.26 |
| -72.0150168 | 18.9054494 | 0.02 | 0.03 | 0.04 | 0.02 | 0.01 | 0.01 | 0.00 | 0.30 | 7.00 | 0.20 |
| -73.64896247 | 18.50929236 | 0.04 | 0.04 | 0.03 | 0.01 | 0.01 | 0.01 | 0.01 | 0.30 | 7.00 | 0.40 |
| -72.00262005 | 18.58070842 | 0.03 | 0.04 | 0.06 | 0.03 | 0.02 | 0.01 | 0.01 | 0.30 | 7.00 | 0.18 |
| -72.66180381 | 19.36143423 | 0.04 | 0.04 | 0.07 | 0.05 | 0.01 | 0.00 | 0.00 | 0.30 | 7.00 | 0.59 |
| -72.0150168 | 18.57262358 | 0.03 | 0.04 | 0.06 | 0.03 | 0.02 | 0.01 | 0.01 | 0.30 | 7.00 | 0.20 |
| -72.00288955 | 18.57289308 | 0.03 | 0.04 | 0.06 | 0.03 | 0.02 | 0.02 | 0.01 | 0.30 | 7.00 | 0.40 |
| -72.00908792 | 18.57639651 | 0.03 | 0.04 | 0.06 | 0.03 | 0.02 | 0.01 | 0.01 | 0.30 | 7.00 | 0.63 |
| -71.97782655 | 18.59041023 | 0.03 | 0.04 | 0.06 | 0.03 | 0.01 | 0.01 | 0.01 | 0.30 | 7.00 | 0.18 |
| -72.74858106 | 19.29136564 | 0.04 | 0.05 | 0.06 | 0.02 | 0.01 | 0.00 | 0.00 | 0.30 | 7.00 | 0.51 |
| -71.99076229 | 18.59445264 | 0.03 | 0.04 | 0.06 | 0.03 | 0.02 | 0.01 | 0.01 | 0.30 | 7.00 | 0.48 |
| -71.97863503 | 18.59094922 | 0.03 | 0.04 | 0.06 | 0.03 | 0.01 | 0.01 | 0.01 | 0.30 | 7.00 | 0.06 |
| -73.04960652 | 18.41443026 | 0.04 | 0.04 | 0.05 | 0.04 | 0.02 | 0.01 | 0.01 | 0.30 | 7.00 | 0.66 |
| -73.04960652 | 18.40930986 | 0.03 | 0.03 | 0.04 | 0.02 | 0.01 | 0.01 | 0.01 | 0.30 | 7.00 | 0.40 |
| -73.0452946 | 18.40715391 | 0.03 | 0.03 | 0.04 | 0.02 | 0.02 | 0.01 | 0.01 | 0.30 | 7.00 | 0.50 |
| -72.01663377 | 18.58097792 | 0.03 | 0.04 | 0.07 | 0.03 | 0.02 | 0.01 | 0.01 | 0.30 | 7.00 | 0.30 |
| -72.02417962 | 18.58448135 | 0.03 | 0.04 | 0.06 | 0.03 | 0.01 | 0.01 | 0.01 | 0.30 | 7.00 | 0.34 |
| -72.01636428 | 18.57289308 | 0.03 | 0.04 | 0.06 | 0.03 | 0.02 | 0.01 | 0.01 | 0.30 | 7.00 | 0.66 |
| -71.97890453 | 18.58502033 | 0.03 | 0.04 | 0.06 | 0.03 | 0.01 | 0.01 | 0.01 | 0.30 | 7.00 | 0.11 |
| -72.0152863 | 18.5914882 | 0.03 | 0.04 | 0.06 | 0.03 | 0.02 | 0.01 | 0.01 | 0.30 | 7.00 | 0.19 |
| -73.90767728 | 18.53300788 | 0.02 | 0.03 | 0.04 | 0.03 | 0.32 | 0.17 | 0.07 | 0.30 | 7.00 | 0.14 |
| -72.00720146 | 18.58394236 | 0.03 | 0.04 | 0.06 | 0.03 | 0.02 | 0.01 | 0.01 | 0.30 | 7.00 | 0.36 |
| -73.04987601 | 18.40338098 | 0.03 | 0.03 | 0.03 | 0.02 | 0.01 | 0.01 | 0.01 | 0.30 | 7.00 | 0.45 |
| -72.01366933 | 18.90706636 | 0.03 | 0.03 | 0.05 | 0.03 | 0.01 | 0.01 | 0.00 | 0.30 | 7.00 | 0.56 |
| -72.00073359 | 18.58852376 | 0.03 | 0.04 | 0.07 | 0.03 | 0.02 | 0.02 | 0.01 | 0.30 | 7.00 | 0.40 |
| -71.99157077 | 18.57936095 | 0.03 | 0.04 | 0.06 | 0.03 | 0.01 | 0.01 | 0.01 | 0.30 | 7.00 | 0.40 |
| -71.97755706 | 18.58771528 | 0.04 | 0.04 | 0.06 | 0.03 | 0.02 | 0.01 | 0.01 | 0.30 | 7.00 | 0.51 |
| -72.005315 | 18.57477954 | 0.03 | 0.04 | 0.06 | 0.03 | 0.02 | 0.01 | 0.01 | 0.30 | 7.00 | 0.17 |
| -71.99884713 | 18.58879326 | 0.03 | 0.04 | 0.07 | 0.03 | 0.02 | 0.02 | 0.02 | 0.30 | 7.00 | 0.32 |
| -72.02121518 | 18.56615571 | 0.03 | 0.04 | 0.06 | 0.02 | 0.01 | 0.01 | 0.01 | 0.30 | 7.00 | 0.40 |
| -73.05203197 | 18.40715391 | 0.03 | 0.03 | 0.03 | 0.02 | 0.01 | 0.01 | 0.01 | 0.30 | 7.00 | 0.01 |
| -72.32224063 | 19.7166281 | 0.03 | 0.04 | 0.05 | 0.02 | 0.01 | 0.01 | 0.01 | 0.30 | 7.00 | 0.54 |
| -72.02229316 | 18.59364416 | 0.03 | 0.04 | 0.06 | 0.03 | 0.02 | 0.01 | 0.01 | 0.30 | 7.00 | 0.62 |
| -71.98079099 | 18.5718151 | 0.03 | 0.04 | 0.06 | 0.02 | 0.01 | 0.01 | 0.01 | 0.30 | 7.00 | 0.36 |
| -73.04313865 | 18.40176402 | 0.03 | 0.03 | 0.04 | 0.02 | 0.01 | 0.01 | 0.01 | 0.30 | 7.00 | 0.56 |
| -71.97594009 | 18.58259488 | 0.04 | 0.04 | 0.06 | 0.03 | 0.02 | 0.01 | 0.01 | 0.30 | 7.00 | 0.03 |
| -72.01744225 | 18.90086799 | 0.02 | 0.03 | 0.04 | 0.02 | 0.01 | 0.01 | 0.00 | 0.30 | 7.00 | 0.22 |
| -73.05095399 | 18.40391997 | 0.03 | 0.03 | 0.03 | 0.02 | 0.01 | 0.01 | 0.01 | 0.30 | 7.00 | 0.12 |
| -72.00235056 | 18.57100662 | 0.04 | 0.04 | 0.06 | 0.03 | 0.02 | 0.01 | 0.01 | 0.30 | 7.00 | 0.53 |
| -71.97971301 | 18.57989994 | 0.03 | 0.04 | 0.06 | 0.03 | 0.02 | 0.02 | 0.01 | 0.30 | 7.00 | 0.24 |
| -72.69549063 | 19.41263821 | 0.03 | 0.05 | 0.06 | 0.03 | 0.01 | 0.01 | 0.01 | 0.30 | 7.00 | 0.41 |
| -72.75585742 | 19.29756402 | 0.04 | 0.05 | 0.04 | 0.01 | 0.01 | 0.00 | 0.00 | 0.30 | 7.00 | 0.12 |
| -72.00181157 | 18.57127611 | 0.04 | 0.04 | 0.06 | 0.03 | 0.02 | 0.01 | 0.01 | 0.30 | 7.00 | 0.27 |
| -72.00827944 | 18.59553062 | 0.03 | 0.04 | 0.06 | 0.03 | 0.02 | 0.02 | 0.01 | 0.30 | 7.00 | 0.59 |
| -72.00208106 | 18.5815169 | 0.03 | 0.04 | 0.06 | 0.02 | 0.02 | 0.01 | 0.01 | 0.30 | 7.00 | 0.04 |
| -72.02876103 | 18.59768658 | 0.03 | 0.04 | 0.07 | 0.03 | 0.02 | 0.02 | 0.01 | 0.30 | 7.00 | 0.17 |
| -73.04906753 | 18.40311149 | 0.03 | 0.03 | 0.03 | 0.02 | 0.01 | 0.01 | 0.01 | 0.30 | 7.00 | 0.02 |
| -72.00235056 | 18.57127611 | 0.04 | 0.04 | 0.06 | 0.03 | 0.02 | 0.02 | 0.01 | 0.30 | 7.00 | 0.62 |
| -74.05940273 | 18.61493423 | 0.04 | 0.04 | 0.03 | 0.01 | 0.02 | 0.01 | 0.01 | 0.30 | 7.00 | 0.46 |
| -71.99399623 | 18.59472214 | 0.03 | 0.04 | 0.06 | 0.03 | 0.02 | 0.01 | 0.01 | 0.30 | 7.00 | 0.21 |
| -73.05499641 | 18.40553694 | 0.03 | 0.04 | 0.04 | 0.02 | 0.02 | 0.01 | 0.01 | 0.30 | 7.00 | 0.25 |
| -71.99426572 | 18.59310517 | 0.03 | 0.04 | 0.06 | 0.03 | 0.02 | 0.02 | 0.01 | 0.30 | 7.00 | 0.54 |
| -72.75208449 | 19.29190463 | 0.04 | 0.05 | 0.05 | 0.02 | 0.01 | 0.00 | 0.00 | 0.30 | 7.00 | 0.32 |
| -72.00558449 | 18.5666947 | 0.03 | 0.04 | 0.06 | 0.03 | 0.02 | 0.01 | 0.01 | 0.30 | 7.00 | 0.69 |
| -73.04718106 | 18.41065734 | 0.03 | 0.03 | 0.03 | 0.02 | 0.01 | 0.01 | 0.01 | 0.30 | 7.00 | 0.51 |
| -72.01097438 | 18.57882196 | 0.03 | 0.04 | 0.06 | 0.03 | 0.02 | 0.01 | 0.01 | 0.30 | 7.00 | 0.42 |
| -73.05257096 | 18.40176402 | 0.03 | 0.04 | 0.04 | 0.02 | 0.02 | 0.01 | 0.01 | 0.30 | 7.00 | 0.49 |
| -71.98564189 | 18.58070842 | 0.03 | 0.04 | 0.06 | 0.03 | 0.01 | 0.01 | 0.01 | 0.30 | 7.00 | 0.38 |
| -72.01070489 | 18.58906275 | 0.03 | 0.04 | 0.06 | 0.03 | 0.02 | 0.02 | 0.01 | 0.30 | 7.00 | 0.32 |
| -72.33032547 | 19.72525192 | 0.03 | 0.04 | 0.05 | 0.02 | 0.01 | 0.01 | 0.01 | 0.30 | 7.00 | 0.44 |
| -72.01393882 | 18.59714759 | 0.03 | 0.04 | 0.06 | 0.03 | 0.02 | 0.01 | 0.01 | 0.30 | 7.00 | 0.57 |
| -72.02067619 | 18.57127611 | 0.03 | 0.04 | 0.07 | 0.03 | 0.02 | 0.01 | 0.01 | 0.30 | 7.00 | 0.67 |
| -72.01097438 | 18.58124741 | 0.03 | 0.04 | 0.06 | 0.03 | 0.02 | 0.01 | 0.01 | 0.30 | 7.00 | 0.25 |
| -71.99399623 | 18.57720499 | 0.04 | 0.04 | 0.06 | 0.03 | 0.01 | 0.01 | 0.01 | 0.30 | 7.00 | 0.15 |
| -71.98806734 | 18.5815169 | 0.03 | 0.04 | 0.06 | 0.03 | 0.01 | 0.01 | 0.01 | 0.30 | 7.00 | 0.01 |
| -71.99749966 | 18.57558802 | 0.04 | 0.04 | 0.06 | 0.03 | 0.02 | 0.02 | 0.01 | 0.30 | 7.00 | 0.11 |
| -72.00262005 | 18.5718151 | 0.03 | 0.04 | 0.06 | 0.03 | 0.02 | 0.02 | 0.01 | 0.30 | 7.00 | 0.20 |
| -73.05014551 | 18.39987755 | 0.03 | 0.03 | 0.03 | 0.02 | 0.01 | 0.01 | 0.01 | 0.30 | 7.00 | 0.51 |
| -71.96893323 | 18.57235409 | 0.04 | 0.04 | 0.07 | 0.03 | 0.02 | 0.01 | 0.01 | 0.30 | 7.00 | 0.52 |
| -72.02876103 | 18.59768658 | 0.03 | 0.04 | 0.07 | 0.03 | 0.02 | 0.02 | 0.01 | 0.30 | 7.00 | 0.35 |
| -72.00666247 | 18.58717629 | 0.03 | 0.04 | 0.06 | 0.03 | 0.02 | 0.01 | 0.01 | 0.30 | 7.00 | 0.46 |
| -71.98618088 | 18.59067972 | 0.03 | 0.04 | 0.06 | 0.03 | 0.01 | 0.01 | 0.01 | 0.30 | 7.00 | 0.14 |
| -71.98321644 | 18.57289308 | 0.03 | 0.04 | 0.06 | 0.03 | 0.01 | 0.01 | 0.01 | 0.30 | 7.00 | 0.28 |
| -73.65057944 | 18.51117882 | 0.04 | 0.04 | 0.04 | 0.01 | 0.01 | 0.01 | 0.01 | 0.30 | 7.00 | 0.10 |
| -72.01259135 | 18.57989994 | 0.03 | 0.04 | 0.06 | 0.03 | 0.02 | 0.01 | 0.01 | 0.30 | 7.00 | 0.40 |
| -72.75289298 | 19.298642 | 0.04 | 0.05 | 0.05 | 0.02 | 0.01 | 0.00 | 0.00 | 0.30 | 7.00 | 0.68 |
| -73.84704099 | 18.31175282 | 0.01 | 0.02 | 0.05 | 0.04 | 0.11 | 0.05 | 0.03 | 0.30 | 7.00 | 0.41 |
| -71.99103179 | 18.58852376 | 0.03 | 0.04 | 0.06 | 0.03 | 0.01 | 0.02 | 0.01 | 0.30 | 7.00 | 0.45 |
| -72.66180381 | 19.36628514 | 0.04 | 0.04 | 0.07 | 0.05 | 0.01 | 0.00 | 0.00 | 0.30 | 7.00 | 0.21 |
| -71.98186897 | 18.57343207 | 0.03 | 0.04 | 0.06 | 0.03 | 0.01 | 0.01 | 0.01 | 0.30 | 7.00 | 0.41 |
| -72.00639298 | 18.59256618 | 0.03 | 0.04 | 0.07 | 0.03 | 0.02 | 0.02 | 0.01 | 0.30 | 7.00 | 0.55 |
| -72.74750309 | 19.29163514 | 0.04 | 0.05 | 0.06 | 0.02 | 0.01 | 0.00 | 0.00 | 0.30 | 7.00 | 0.23 |
| -72.02606608 | 18.57909145 | 0.03 | 0.04 | 0.06 | 0.03 | 0.02 | 0.01 | 0.01 | 0.30 | 7.00 | 0.50 |
| -72.00369803 | 18.57316257 | 0.04 | 0.04 | 0.06 | 0.03 | 0.02 | 0.02 | 0.02 | 0.30 | 7.00 | 0.08 |
| -71.97755706 | 18.58259488 | 0.03 | 0.04 | 0.06 | 0.03 | 0.01 | 0.01 | 0.01 | 0.30 | 7.00 | 0.29 |
| -71.99157077 | 18.5917577 | 0.03 | 0.04 | 0.06 | 0.03 | 0.01 | 0.01 | 0.01 | 0.30 | 7.00 | 0.52 |
| -73.04987601 | 18.40957936 | 0.03 | 0.04 | 0.04 | 0.02 | 0.02 | 0.01 | 0.01 | 0.30 | 7.00 | 0.10 |
| -72.32763052 | 19.71986203 | 0.03 | 0.04 | 0.06 | 0.03 | 0.02 | 0.02 | 0.01 | 0.30 | 7.00 | 0.17 |
| -72.02876103 | 18.59606961 | 0.03 | 0.04 | 0.07 | 0.03 | 0.02 | 0.02 | 0.01 | 0.30 | 7.00 | 0.15 |
| -71.98321644 | 18.57424055 | 0.03 | 0.04 | 0.06 | 0.03 | 0.01 | 0.01 | 0.01 | 0.30 | 7.00 | 0.57 |
| -71.99615218 | 18.57585752 | 0.04 | 0.04 | 0.06 | 0.03 | 0.02 | 0.02 | 0.01 | 0.30 | 7.00 | 0.17 |
| -72.02094568 | 18.59229669 | 0.03 | 0.04 | 0.06 | 0.03 | 0.02 | 0.01 | 0.01 | 0.30 | 7.00 | 0.06 |
| -73.05391843 | 18.40311149 | 0.03 | 0.04 | 0.04 | 0.02 | 0.02 | 0.01 | 0.01 | 0.30 | 7.00 | 0.20 |
| -72.32331861 | 19.71770608 | 0.03 | 0.04 | 0.05 | 0.03 | 0.02 | 0.02 | 0.01 | 0.30 | 7.00 | 0.27 |
| -71.98995381 | 18.56992864 | 0.04 | 0.04 | 0.06 | 0.03 | 0.01 | 0.01 | 0.01 | 0.30 | 7.00 | 0.16 |
| -72.01852023 | 18.56561672 | 0.03 | 0.03 | 0.06 | 0.02 | 0.01 | 0.01 | 0.01 | 0.30 | 7.00 | 0.19 |
| -73.050415 | 18.40850138 | 0.03 | 0.04 | 0.04 | 0.02 | 0.02 | 0.01 | 0.01 | 0.30 | 7.00 | 0.10 |
| -71.9902233 | 18.58502033 | 0.03 | 0.04 | 0.06 | 0.03 | 0.01 | 0.01 | 0.01 | 0.30 | 7.00 | 0.24 |
| -72.00989641 | 18.59283568 | 0.03 | 0.04 | 0.06 | 0.03 | 0.02 | 0.01 | 0.01 | 0.30 | 7.00 | 0.05 |
| -72.02337114 | 18.56777268 | 0.03 | 0.04 | 0.06 | 0.03 | 0.01 | 0.01 | 0.01 | 0.30 | 7.00 | 0.06 |
| -72.68929226 | 19.41048225 | 0.04 | 0.05 | 0.06 | 0.03 | 0.01 | 0.01 | 0.01 | 0.30 | 7.00 | 0.16 |
| -72.02606608 | 18.59795607 | 0.03 | 0.04 | 0.07 | 0.03 | 0.02 | 0.02 | 0.01 | 0.30 | 7.00 | 0.08 |
| -72.01313034 | 18.59094922 | 0.03 | 0.04 | 0.06 | 0.03 | 0.02 | 0.01 | 0.01 | 0.30 | 7.00 | 0.50 |
| -73.0452946 | 18.40365048 | 0.03 | 0.03 | 0.03 | 0.02 | 0.01 | 0.01 | 0.01 | 0.30 | 7.00 | 0.35 |
| -72.01798124 | 18.59337467 | 0.03 | 0.04 | 0.06 | 0.03 | 0.02 | 0.01 | 0.01 | 0.30 | 7.00 | 0.70 |
| -74.05428233 | 18.61250878 | 0.05 | 0.06 | 0.05 | 0.02 | 0.02 | 0.02 | 0.02 | 0.30 | 7.00 | 0.16 |
| -73.90740778 | 18.5319299 | 0.03 | 0.03 | 0.05 | 0.04 | 0.30 | 0.18 | 0.08 | 0.30 | 7.00 | 0.67 |
| -71.98564189 | 18.58367286 | 0.03 | 0.04 | 0.06 | 0.03 | 0.01 | 0.01 | 0.01 | 0.30 | 7.00 | 0.70 |
| -71.98348594 | 18.58394236 | 0.03 | 0.04 | 0.06 | 0.03 | 0.01 | 0.01 | 0.01 | 0.30 | 7.00 | 0.29 |
| -72.00720146 | 18.58744579 | 0.03 | 0.04 | 0.06 | 0.03 | 0.02 | 0.02 | 0.01 | 0.30 | 7.00 | 0.58 |
| -72.01366933 | 18.57909145 | 0.03 | 0.04 | 0.06 | 0.03 | 0.02 | 0.01 | 0.01 | 0.30 | 7.00 | 0.49 |
| -72.01070489 | 18.59364416 | 0.03 | 0.04 | 0.07 | 0.03 | 0.02 | 0.01 | 0.01 | 0.30 | 7.00 | 0.63 |
| -72.01690327 | 18.90598839 | 0.02 | 0.03 | 0.05 | 0.02 | 0.01 | 0.01 | 0.00 | 0.30 | 7.00 | 0.18 |
| -72.01259135 | 18.59580012 | 0.03 | 0.04 | 0.07 | 0.03 | 0.02 | 0.02 | 0.01 | 0.30 | 7.00 | 0.47 |
| -71.99561319 | 18.58636781 | 0.03 | 0.04 | 0.06 | 0.03 | 0.02 | 0.02 | 0.01 | 0.30 | 7.00 | 0.52 |
| -72.00423702 | 18.58636781 | 0.03 | 0.04 | 0.06 | 0.03 | 0.02 | 0.01 | 0.01 | 0.30 | 7.00 | 0.58 |
| -71.99480471 | 18.57046763 | 0.03 | 0.04 | 0.06 | 0.03 | 0.01 | 0.01 | 0.01 | 0.30 | 7.00 | 0.54 |
| -73.050415 | 18.40850138 | 0.03 | 0.04 | 0.04 | 0.02 | 0.02 | 0.01 | 0.01 | 0.30 | 7.00 | 0.51 |
| -72.02094568 | 18.58933225 | 0.03 | 0.04 | 0.07 | 0.03 | 0.02 | 0.01 | 0.01 | 0.30 | 7.00 | 0.10 |
| -72.02849153 | 18.59256618 | 0.03 | 0.04 | 0.07 | 0.03 | 0.02 | 0.02 | 0.01 | 0.30 | 7.00 | 0.32 |
| -72.01259135 | 18.57855246 | 0.03 | 0.04 | 0.06 | 0.03 | 0.02 | 0.01 | 0.01 | 0.30 | 7.00 | 0.07 |
| -71.98213846 | 18.57504903 | 0.03 | 0.04 | 0.06 | 0.03 | 0.01 | 0.01 | 0.01 | 0.30 | 7.00 | 0.58 |
| -71.99749966 | 18.57397106 | 0.04 | 0.04 | 0.07 | 0.03 | 0.02 | 0.02 | 0.01 | 0.30 | 7.00 | 0.18 |
| -71.99803864 | 18.57477954 | 0.04 | 0.04 | 0.07 | 0.03 | 0.02 | 0.02 | 0.01 | 0.30 | 7.00 | 0.48 |
| -71.97594009 | 18.57720499 | 0.03 | 0.04 | 0.06 | 0.03 | 0.01 | 0.01 | 0.01 | 0.30 | 7.00 | 0.24 |
| -72.01959821 | 18.58475084 | 0.03 | 0.04 | 0.07 | 0.03 | 0.02 | 0.01 | 0.01 | 0.30 | 7.00 | 0.54 |
| -71.97836554 | 18.56912015 | 0.03 | 0.04 | 0.06 | 0.03 | 0.01 | 0.01 | 0.01 | 0.30 | 7.00 | 0.54 |
| -72.01905922 | 18.57073712 | 0.03 | 0.03 | 0.06 | 0.03 | 0.01 | 0.01 | 0.01 | 0.30 | 7.00 | 0.07 |
| -72.00558449 | 18.59364416 | 0.03 | 0.04 | 0.06 | 0.03 | 0.02 | 0.01 | 0.01 | 0.30 | 7.00 | 0.63 |
| -71.98995381 | 18.57100662 | 0.04 | 0.04 | 0.06 | 0.03 | 0.01 | 0.01 | 0.01 | 0.30 | 7.00 | 0.65 |
| -71.98645038 | 18.57208459 | 0.04 | 0.04 | 0.06 | 0.03 | 0.02 | 0.01 | 0.01 | 0.30 | 7.00 | 0.36 |
| -72.75397096 | 19.29082665 | 0.04 | 0.05 | 0.05 | 0.02 | 0.01 | 0.00 | 0.00 | 0.30 | 7.00 | 0.29 |
| -72.01339984 | 18.58744579 | 0.03 | 0.04 | 0.07 | 0.03 | 0.02 | 0.01 | 0.01 | 0.30 | 7.00 | 0.09 |
| -72.01582529 | 18.58798477 | 0.03 | 0.04 | 0.07 | 0.03 | 0.02 | 0.02 | 0.01 | 0.30 | 7.00 | 0.47 |
| -72.00962691 | 18.57909145 | 0.03 | 0.04 | 0.06 | 0.03 | 0.02 | 0.01 | 0.01 | 0.30 | 7.00 | 0.70 |
| -72.02175417 | 18.58340337 | 0.03 | 0.04 | 0.07 | 0.03 | 0.02 | 0.01 | 0.01 | 0.30 | 7.00 | 0.26 |
| -72.01878973 | 18.57289308 | 0.03 | 0.04 | 0.06 | 0.03 | 0.02 | 0.01 | 0.01 | 0.30 | 7.00 | 0.08 |
| -72.75262348 | 19.29406059 | 0.04 | 0.05 | 0.05 | 0.02 | 0.01 | 0.00 | 0.00 | 0.30 | 7.00 | 0.31 |
| -72.00450651 | 18.57855246 | 0.03 | 0.04 | 0.06 | 0.03 | 0.02 | 0.01 | 0.01 | 0.30 | 7.00 | 0.04 |
| -72.00477601 | 18.58448135 | 0.03 | 0.04 | 0.06 | 0.03 | 0.02 | 0.01 | 0.01 | 0.30 | 7.00 | 0.61 |
| -72.01744225 | 18.58205589 | 0.03 | 0.04 | 0.07 | 0.03 | 0.02 | 0.01 | 0.01 | 0.30 | 7.00 | 0.67 |
| -72.0104354 | 18.58016943 | 0.03 | 0.04 | 0.06 | 0.03 | 0.02 | 0.01 | 0.01 | 0.30 | 7.00 | 0.25 |
| -72.02040669 | 18.56965914 | 0.04 | 0.04 | 0.06 | 0.03 | 0.02 | 0.02 | 0.01 | 0.30 | 7.00 | 0.55 |
| -72.01582529 | 18.59714759 | 0.03 | 0.04 | 0.06 | 0.03 | 0.02 | 0.01 | 0.01 | 0.30 | 7.00 | 0.18 |
| -72.74912005 | 19.29729452 | 0.04 | 0.05 | 0.05 | 0.02 | 0.01 | 0.00 | 0.00 | 0.30 | 7.00 | 0.01 |
| -71.99103179 | 18.56992864 | 0.03 | 0.04 | 0.06 | 0.03 | 0.01 | 0.01 | 0.01 | 0.30 | 7.00 | 0.46 |
| -72.0050455 | 18.59310517 | 0.03 | 0.04 | 0.06 | 0.03 | 0.02 | 0.01 | 0.01 | 0.30 | 7.00 | 0.25 |
| -71.99884713 | 18.5669642 | 0.03 | 0.04 | 0.06 | 0.03 | 0.01 | 0.01 | 0.01 | 0.30 | 7.00 | 0.28 |
| -73.65462186 | 18.50821438 | 0.04 | 0.04 | 0.04 | 0.01 | 0.01 | 0.01 | 0.00 | 0.30 | 7.00 | 0.54 |
| -72.01744225 | 18.58232539 | 0.03 | 0.04 | 0.07 | 0.03 | 0.02 | 0.01 | 0.01 | 0.30 | 7.00 | 0.30 |
| -71.99588269 | 18.59229669 | 0.03 | 0.04 | 0.06 | 0.03 | 0.02 | 0.01 | 0.01 | 0.30 | 7.00 | 0.09 |
| -72.74831157 | 19.29594705 | 0.04 | 0.05 | 0.05 | 0.02 | 0.01 | 0.00 | 0.00 | 0.30 | 7.00 | 0.49 |
| -73.65354388 | 18.51090932 | 0.04 | 0.04 | 0.04 | 0.01 | 0.01 | 0.01 | 0.01 | 0.30 | 7.00 | 0.60 |
| -71.97998251 | 18.5917577 | 0.03 | 0.04 | 0.06 | 0.03 | 0.01 | 0.01 | 0.01 | 0.30 | 7.00 | 0.24 |
| -72.02256265 | 18.57127611 | 0.03 | 0.04 | 0.06 | 0.03 | 0.01 | 0.01 | 0.01 | 0.30 | 7.00 | 0.10 |
| -71.99345724 | 18.57585752 | 0.03 | 0.04 | 0.06 | 0.03 | 0.01 | 0.01 | 0.01 | 0.30 | 7.00 | 0.14 |
| -72.01393882 | 18.59526113 | 0.03 | 0.04 | 0.07 | 0.03 | 0.02 | 0.01 | 0.01 | 0.30 | 7.00 | 0.62 |
| -72.65937836 | 19.36574615 | 0.04 | 0.04 | 0.07 | 0.05 | 0.01 | 0.00 | 0.00 | 0.30 | 7.00 | 0.24 |
| -72.0101659 | 18.57127611 | 0.03 | 0.04 | 0.06 | 0.03 | 0.02 | 0.01 | 0.01 | 0.30 | 7.00 | 0.52 |
| -72.3235881 | 19.71743658 | 0.03 | 0.04 | 0.05 | 0.02 | 0.02 | 0.01 | 0.01 | 0.30 | 7.00 | 0.37 |
| -71.99291825 | 18.57208459 | 0.04 | 0.04 | 0.06 | 0.03 | 0.02 | 0.01 | 0.01 | 0.30 | 7.00 | 0.35 |
| -72.00181157 | 18.57936095 | 0.03 | 0.04 | 0.06 | 0.03 | 0.02 | 0.01 | 0.01 | 0.30 | 7.00 | 0.10 |
| -72.02202366 | 18.59418315 | 0.03 | 0.04 | 0.06 | 0.03 | 0.02 | 0.01 | 0.01 | 0.30 | 7.00 | 0.23 |
| -71.98806734 | 18.57316257 | 0.03 | 0.04 | 0.06 | 0.03 | 0.01 | 0.01 | 0.01 | 0.30 | 7.00 | 0.10 |
| -72.74938955 | 19.29998947 | 0.04 | 0.05 | 0.05 | 0.02 | 0.01 | 0.00 | 0.00 | 0.30 | 7.00 | 0.36 |
| -72.0104354 | 18.59014073 | 0.03 | 0.04 | 0.06 | 0.03 | 0.02 | 0.02 | 0.01 | 0.30 | 7.00 | 0.23 |
| -72.74858106 | 19.29891149 | 0.04 | 0.05 | 0.05 | 0.02 | 0.01 | 0.00 | 0.00 | 0.30 | 7.00 | 0.48 |
| -72.76609821 | 19.23584976 | 0.05 | 0.07 | 0.11 | 0.10 | 0.04 | 0.01 | 0.01 | 0.30 | 7.00 | 0.55 |
| -72.0004641 | 18.59041023 | 0.03 | 0.04 | 0.07 | 0.03 | 0.02 | 0.02 | 0.01 | 0.30 | 7.00 | 0.53 |
| -72.01124388 | 18.90841384 | 0.02 | 0.03 | 0.05 | 0.02 | 0.01 | 0.01 | 0.00 | 0.30 | 7.00 | 0.15 |
| -71.97001121 | 18.57370156 | 0.04 | 0.04 | 0.07 | 0.03 | 0.02 | 0.02 | 0.02 | 0.30 | 7.00 | 0.68 |
| -74.08177078 | 18.62086311 | 0.06 | 0.07 | 0.06 | 0.03 | 0.03 | 0.03 | 0.02 | 0.30 | 7.00 | 0.16 |
| -71.99318774 | 18.576666 | 0.04 | 0.04 | 0.06 | 0.03 | 0.02 | 0.01 | 0.01 | 0.30 | 7.00 | 0.26 |
| -72.01366933 | 18.59041023 | 0.03 | 0.04 | 0.06 | 0.03 | 0.02 | 0.01 | 0.01 | 0.30 | 7.00 | 0.29 |
| -71.98725886 | 18.58825427 | 0.03 | 0.04 | 0.06 | 0.03 | 0.02 | 0.01 | 0.01 | 0.30 | 7.00 | 0.10 |
| -71.9904928 | 18.58852376 | 0.04 | 0.04 | 0.06 | 0.03 | 0.02 | 0.02 | 0.01 | 0.30 | 7.00 | 0.04 |
| -72.74480814 | 19.2935216 | 0.04 | 0.05 | 0.06 | 0.03 | 0.01 | 0.00 | 0.00 | 0.30 | 7.00 | 0.05 |
| -71.9902233 | 18.58879326 | 0.03 | 0.04 | 0.06 | 0.03 | 0.02 | 0.01 | 0.01 | 0.30 | 7.00 | 0.14 |
| -71.98645038 | 18.57343207 | 0.03 | 0.04 | 0.06 | 0.03 | 0.01 | 0.01 | 0.01 | 0.30 | 7.00 | 0.24 |
| -72.02094568 | 18.56804218 | 0.03 | 0.04 | 0.06 | 0.02 | 0.01 | 0.01 | 0.01 | 0.30 | 7.00 | 0.50 |
| -72.6922567 | 19.40886528 | 0.04 | 0.05 | 0.06 | 0.03 | 0.01 | 0.01 | 0.01 | 0.30 | 7.00 | 0.36 |
| -72.01986771 | 18.9054494 | 0.02 | 0.03 | 0.05 | 0.02 | 0.01 | 0.01 | 0.01 | 0.30 | 7.00 | 0.05 |
| -73.65327439 | 18.50821438 | 0.04 | 0.04 | 0.04 | 0.01 | 0.01 | 0.01 | 0.01 | 0.30 | 7.00 | 0.17 |
| -72.02337114 | 18.59445264 | 0.03 | 0.04 | 0.06 | 0.03 | 0.02 | 0.01 | 0.01 | 0.30 | 7.00 | 0.51 |
| -72.00262005 | 18.58286438 | 0.03 | 0.04 | 0.06 | 0.03 | 0.02 | 0.01 | 0.01 | 0.30 | 7.00 | 0.64 |
| -73.65381338 | 18.50767539 | 0.04 | 0.04 | 0.04 | 0.01 | 0.01 | 0.01 | 0.00 | 0.30 | 7.00 | 0.28 |
| -72.01771175 | 18.59067972 | 0.03 | 0.04 | 0.07 | 0.03 | 0.02 | 0.01 | 0.01 | 0.30 | 7.00 | 0.64 |
| -71.98159947 | 18.58232539 | 0.03 | 0.04 | 0.06 | 0.03 | 0.01 | 0.01 | 0.01 | 0.30 | 7.00 | 0.32 |
| -71.99696067 | 18.56992864 | 0.04 | 0.04 | 0.06 | 0.03 | 0.02 | 0.01 | 0.01 | 0.30 | 7.00 | 0.65 |
| -72.00477601 | 18.59202719 | 0.03 | 0.04 | 0.06 | 0.03 | 0.02 | 0.02 | 0.01 | 0.30 | 7.00 | 0.40 |
| -71.99561319 | 18.57558802 | 0.04 | 0.04 | 0.06 | 0.03 | 0.02 | 0.02 | 0.01 | 0.30 | 7.00 | 0.22 |
| -72.00774045 | 18.58771528 | 0.03 | 0.04 | 0.06 | 0.03 | 0.02 | 0.01 | 0.01 | 0.30 | 7.00 | 0.49 |
| -72.00774045 | 18.58528983 | 0.03 | 0.04 | 0.06 | 0.03 | 0.02 | 0.01 | 0.01 | 0.30 | 7.00 | 0.69 |
| -71.98968431 | 18.56831167 | 0.04 | 0.04 | 0.06 | 0.03 | 0.01 | 0.01 | 0.01 | 0.30 | 7.00 | 0.58 |
| -71.9953437 | 18.57316257 | 0.04 | 0.04 | 0.06 | 0.03 | 0.02 | 0.01 | 0.01 | 0.30 | 7.00 | 0.23 |
| -72.02067619 | 18.56399976 | 0.03 | 0.04 | 0.06 | 0.03 | 0.01 | 0.01 | 0.01 | 0.30 | 7.00 | 0.48 |
| -72.00073359 | 18.59553062 | 0.03 | 0.04 | 0.07 | 0.03 | 0.02 | 0.02 | 0.01 | 0.30 | 7.00 | 0.31 |
| -71.97297565 | 18.57531853 | 0.03 | 0.04 | 0.06 | 0.03 | 0.01 | 0.01 | 0.01 | 0.30 | 7.00 | 0.31 |
| -71.97324514 | 18.57397106 | 0.03 | 0.04 | 0.06 | 0.03 | 0.01 | 0.01 | 0.01 | 0.30 | 7.00 | 0.35 |
| -71.99399623 | 18.57882196 | 0.04 | 0.04 | 0.07 | 0.03 | 0.02 | 0.02 | 0.01 | 0.30 | 7.00 | 0.39 |
| -73.05445742 | 18.40014705 | 0.03 | 0.03 | 0.04 | 0.02 | 0.02 | 0.01 | 0.01 | 0.30 | 7.00 | 0.40 |
| -72.00854893 | 18.90949181 | 0.02 | 0.03 | 0.05 | 0.03 | 0.01 | 0.01 | 0.00 | 0.30 | 7.00 | 0.10 |
| -71.98806734 | 18.57262358 | 0.03 | 0.04 | 0.06 | 0.03 | 0.01 | 0.01 | 0.01 | 0.30 | 7.00 | 0.59 |
| -71.98159947 | 18.59256618 | 0.03 | 0.04 | 0.06 | 0.03 | 0.01 | 0.01 | 0.01 | 0.30 | 7.00 | 0.35 |
| -72.00585399 | 18.57262358 | 0.03 | 0.04 | 0.07 | 0.03 | 0.02 | 0.02 | 0.01 | 0.30 | 7.00 | 0.10 |
| -71.98159947 | 18.58043893 | 0.04 | 0.04 | 0.07 | 0.03 | 0.02 | 0.02 | 0.01 | 0.30 | 7.00 | 0.64 |
| -72.6968381 | 19.41021275 | 0.04 | 0.04 | 0.05 | 0.03 | 0.01 | 0.01 | 0.01 | 0.30 | 7.00 | 0.60 |
| -73.04852854 | 18.41065734 | 0.03 | 0.03 | 0.03 | 0.02 | 0.01 | 0.01 | 0.01 | 0.30 | 7.00 | 0.41 |
| -72.32628305 | 19.7163586 | 0.03 | 0.04 | 0.06 | 0.03 | 0.02 | 0.01 | 0.01 | 0.30 | 7.00 | 0.07 |
| -71.97836554 | 18.57235409 | 0.03 | 0.04 | 0.06 | 0.03 | 0.01 | 0.01 | 0.01 | 0.30 | 7.00 | 0.04 |
| -71.98591139 | 18.58906275 | 0.03 | 0.04 | 0.06 | 0.03 | 0.02 | 0.01 | 0.01 | 0.30 | 7.00 | 0.01 |
| -71.97594009 | 18.58502033 | 0.03 | 0.04 | 0.06 | 0.03 | 0.02 | 0.01 | 0.01 | 0.30 | 7.00 | 0.50 |
| -73.05257096 | 18.41523875 | 0.03 | 0.03 | 0.04 | 0.03 | 0.02 | 0.01 | 0.01 | 0.30 | 7.00 | 0.59 |
| -72.75343197 | 19.29729452 | 0.04 | 0.05 | 0.05 | 0.02 | 0.01 | 0.00 | 0.00 | 0.30 | 7.00 | 0.43 |
| -74.05859424 | 18.61223929 | 0.04 | 0.04 | 0.04 | 0.02 | 0.02 | 0.02 | 0.01 | 0.30 | 7.00 | 0.21 |
| -71.97836554 | 18.5869068 | 0.03 | 0.04 | 0.06 | 0.03 | 0.01 | 0.01 | 0.01 | 0.30 | 7.00 | 0.00 |
| -71.97486211 | 18.58475084 | 0.03 | 0.04 | 0.06 | 0.03 | 0.02 | 0.01 | 0.01 | 0.30 | 7.00 | 0.22 |
| -72.01366933 | 18.57720499 | 0.03 | 0.04 | 0.06 | 0.03 | 0.02 | 0.01 | 0.01 | 0.30 | 7.00 | 0.09 |
| -71.98456392 | 18.57397106 | 0.03 | 0.04 | 0.06 | 0.03 | 0.01 | 0.01 | 0.01 | 0.30 | 7.00 | 0.38 |
| -71.9853724 | 18.57963044 | 0.03 | 0.04 | 0.06 | 0.03 | 0.01 | 0.01 | 0.01 | 0.30 | 7.00 | 0.27 |
| -71.98456392 | 18.59202719 | 0.03 | 0.04 | 0.06 | 0.03 | 0.01 | 0.01 | 0.01 | 0.30 | 7.00 | 0.11 |
| -71.97890453 | 18.58906275 | 0.03 | 0.04 | 0.06 | 0.03 | 0.01 | 0.01 | 0.01 | 0.30 | 7.00 | 0.47 |
| -72.01744225 | 18.58906275 | 0.03 | 0.04 | 0.07 | 0.03 | 0.02 | 0.02 | 0.01 | 0.30 | 7.00 | 0.35 |
| -72.74426915 | 19.29594705 | 0.04 | 0.05 | 0.06 | 0.03 | 0.01 | 0.00 | 0.00 | 0.30 | 7.00 | 0.06 |
| -73.05310995 | 18.40068604 | 0.04 | 0.03 | 0.04 | 0.02 | 0.02 | 0.01 | 0.01 | 0.30 | 7.00 | 0.26 |
| -71.97405363 | 18.58016943 | 0.04 | 0.04 | 0.06 | 0.03 | 0.02 | 0.01 | 0.01 | 0.30 | 7.00 | 0.53 |
| -72.00181157 | 18.59553062 | 0.03 | 0.04 | 0.06 | 0.03 | 0.02 | 0.02 | 0.01 | 0.30 | 7.00 | 0.14 |
| -72.74885056 | 19.29675553 | 0.04 | 0.05 | 0.05 | 0.02 | 0.01 | 0.00 | 0.00 | 0.30 | 7.00 | 0.03 |
| -72.01690327 | 18.59364416 | 0.03 | 0.04 | 0.06 | 0.03 | 0.02 | 0.02 | 0.01 | 0.30 | 7.00 | 0.46 |
| -72.751815 | 19.29783351 | 0.04 | 0.05 | 0.05 | 0.02 | 0.01 | 0.00 | 0.00 | 0.30 | 7.00 | 0.62 |
| -71.99237926 | 18.57558802 | 0.03 | 0.04 | 0.06 | 0.03 | 0.01 | 0.01 | 0.01 | 0.30 | 7.00 | 0.37 |
| -72.02337114 | 18.56588622 | 0.04 | 0.04 | 0.06 | 0.03 | 0.01 | 0.01 | 0.01 | 0.30 | 7.00 | 0.43 |
| -72.01986771 | 18.57127611 | 0.03 | 0.04 | 0.06 | 0.03 | 0.01 | 0.01 | 0.01 | 0.30 | 7.00 | 0.25 |
| -72.0252576 | 18.58960174 | 0.03 | 0.04 | 0.07 | 0.03 | 0.02 | 0.01 | 0.01 | 0.30 | 7.00 | 0.38 |
| -72.01663377 | 18.57316257 | 0.03 | 0.04 | 0.06 | 0.03 | 0.01 | 0.01 | 0.01 | 0.30 | 7.00 | 0.27 |
| -72.74346067 | 19.29971997 | 0.04 | 0.05 | 0.06 | 0.03 | 0.01 | 0.01 | 0.00 | 0.30 | 7.00 | 0.19 |
| -71.98618088 | 18.57774398 | 0.03 | 0.04 | 0.06 | 0.03 | 0.01 | 0.01 | 0.01 | 0.30 | 7.00 | 0.15 |
| -71.98348594 | 18.57343207 | 0.03 | 0.04 | 0.06 | 0.02 | 0.01 | 0.01 | 0.01 | 0.30 | 7.00 | 0.63 |
| -71.98159947 | 18.5817864 | 0.03 | 0.04 | 0.06 | 0.03 | 0.02 | 0.02 | 0.01 | 0.30 | 7.00 | 0.49 |
| -73.05310995 | 18.40580644 | 0.03 | 0.03 | 0.04 | 0.02 | 0.01 | 0.01 | 0.01 | 0.30 | 7.00 | 0.58 |
| -72.00181157 | 18.59041023 | 0.03 | 0.04 | 0.07 | 0.03 | 0.02 | 0.02 | 0.01 | 0.30 | 7.00 | 0.16 |
| -72.01205236 | 18.59445264 | 0.03 | 0.04 | 0.07 | 0.03 | 0.02 | 0.01 | 0.01 | 0.30 | 7.00 | 0.03 |
| -71.98213846 | 18.58717629 | 0.03 | 0.04 | 0.06 | 0.02 | 0.01 | 0.01 | 0.01 | 0.30 | 7.00 | 0.64 |
| -73.64977096 | 18.51010084 | 0.04 | 0.04 | 0.04 | 0.01 | 0.01 | 0.01 | 0.01 | 0.30 | 7.00 | 0.47 |
